# Automated cleaning of tie point clouds following USGS guidelines in Agisoft Metashape professional (ver. 2.1.0)

**DOI:** 10.1016/j.mex.2024.102679

**Published:** 2024-03-26

**Authors:** Joel Mohren, Maximilian Schulze

**Affiliations:** a Department of Geography, RWTH Aachen University, Wüllnerstraße 5b, Aachen 52062, Germany; b Institute of Geology and Mineralogy, University of Cologne, Zülpicher Str. 49b, Cologne 50674, Germany

**Keywords:** Photogrammetry, Camera model optimization, Quality measures monitoring, SfM-MVS, Automated camera model optimization

## Abstract

The U.S. Geological Survey (USGS) has published a guideline to improve the quality of digital photogrammetric reconstructions created with the widely used Agisoft Metashape Professional software. The suggested workflows aim at filtering out low-quality tie points from the tie point cloud to optimize the camera model. However, the optimization procedure relies on an iteratively performed trial-and-error approach. If manually performed, the time expenditure required from the operator can be significant and the optimization process can be affected by the degree of diligence that is applied.

To minimize the time expenditure and attentiveness required from the operator and to provide a framework for an improved reproducibility of camera model optimization workflows, we present here a python script serving as an extension for Agisoft Metashape Professional (tested on version 2.1.0) that automatizes the iterative point filtering procedure proposed by the USGS. As a result, the entire processing cycle can be performed largely unattended.

• A graphical user interface allows to individually adjust important camera model optimization parameters.
• Main tie point cloud quality measures can be directly assessed.
• The reproducibility of the automated camera model optimization as tested in this study generally is above 99%.

## Graphical abstract

**Figure:**
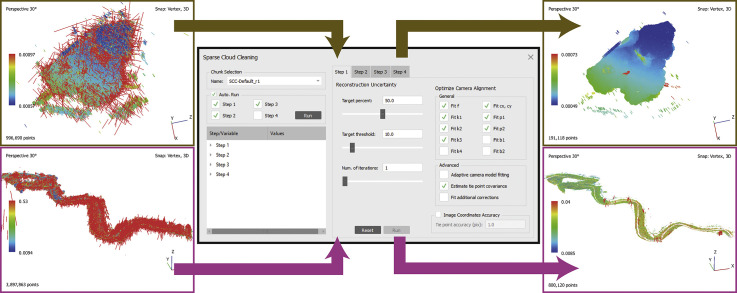

Specifications table

**Table:** 

Subject area:	Computer Science
More specific subject area:	Structure-from-Motion Multi-View Stereo Photogrammetry
Name of your method:	Automated camera model optimization
Name and reference of original method:	*Processing coastal imagery with Agisoft Metashape Professional Edition, version 1.6—Structure from motion workflow documentation.*
Resource availability:	https://github.com/MaximilianSchulze/metashape-scc
Direct submission or co-submission:	Direct submission

## Background

Since Structure-from-Motion Multi-View Stereo (SfM-MVS) techniques have emerged in geosciences (e.g. [1,2]), their application has skyrocketed and the products derived from SfM-MVS-based image processing algorithms (i.e. point clouds, meshes, digital elevation models, and/or derivations) have become a key fundament helping to address geoscientific research questions in many studies (e.g. [3,4]). Typically associated spatial scales for terrestrial geoscientific applications cover a wide range, including individual clasts and bulk sediment samples (e.g. [5], [6], [7]), small to medium-scaled topographical features and landforms such as soil pits or outcrops (e.g. [8,9]), and larger-scaled landforms (e.g. Alluvial fans, [10]) as well as landscapes (e.g. [11], [12], [13], [14], [15]).

SfM-MVS-based products can be based on a variety of optical image acquisition devices, including customer-grade smartphones for close-range photogrammetry applications (e.g. [5,8]) and unmanned aerial systems (UASs) commonly used for spatially extensive projects (e.g. [10,12]). The improvement in smartphone and UASs camera performances and the general availability of low-cost imaging devices and UASs increasingly shift the weight of factors contributing to the overall quality of SfM-MVS-based products towards the acquisition strategy applied and processing software used (cf. [16]). Today, smartphone applications provide capabilities for real-time model construction based on the smartphone's camera, and light detection and ranging (LiDAR)-equipped smartphones and other LiDAR handheld devices are increasingly considered as alternatives to SfM-MVS workflows for medium spatial scales (e.g. [17]).

However, at the time of writing, these techniques tailored for non-expert users do not yet appear to offer the spatial accuracy achieved with traditional SfM-MVS workflows. Furthermore, they are not yet suitable for capturing landscape-sized scenarios (cf. [17]). At the same time, the use of professional LiDAR equipment is still more cost-intensive than the application of SfM-MVS photogrammetry techniques. For this method, commercial photogrammetry software usually provides convenient graphical user interfaces (GUIs) to produce high-quality derivations from photogrammetric surveys, including possibilities for precise spatial referencing and error monitoring. In the field of geomorphology, Agisoft's Metashape (formally Agisoft Photoscan; current version 2.1.0.17530) is widely used to generate SfM-MVS-based products (cf. [18,19]). Such commercial photogrammetry software packages are naturally provided as black boxes (cf. [18]), providing limited insight into the algorithms the software is built on. This economic necessity limits the traceability of how products are generated and – important for most scientific applications – how accuracy estimates are derived (cf. [20]). In addition, external error sources such as the availability of georeferenced ground control points and/or cameras affect the overall accuracy of the final product (e.g. [13,19,21,22]).

An approach to assess SfM-MVS-derived product accuracies relies on empirical comparisons with internal or external benchmarks. For example, Mohren et al. [8] compared soil bulk densities obtained by using SfM-MVS photogrammetry workflows with reference densities derived using soil sampling rings and polyurethane foam. On a larger spatial scale, James et al. [22] varied the number of GCPs used to optimize the camera model of an UAS survey and compared the results to a benchmark model generated using all GCPs. Other authors aimed for providing instructions and/or software tools to assess and optimize SfM-MVS-based product generation workflows and accuracies (e.g. [16,22,23]). A very detailed instruction on accurate SfM-MVS-based product building with focus on coastal imagery processing in Agisoft Metashape Professional has been presented by the United States Geological Survey (USGS), as published by Over et al. [20]. In their publication (open file report 2021-1039), the authors detail sparse cloud cleaning steps and error monitoring based on reconstruction error, projection accuracy and reprojection error estimates as implemented in the software. Although written for an older version of the software (version 1.6), the report still provides up-to-date, detailed, and comprehensive step-by-step instructions helping software users to improve the camera model and hence the quality of SfM-MVS-based products.

The application of SfM-MVS photogrammetry techniques at the Institute of Geology and Mineralogy, University of Cologne, Germany, is based on Agisoft Metashape Professional workflows and focuses on the generation of digital elevation and volumetric data (including in situ soil density determinations, [8]) for scientific analyses as well as simple digitalization of rock and mineral specimen for teaching purposes. To achieve a standardized workflow and obtain high-quality camera alignment models among different operators, we follow the guidelines of Over et al. [20] for SfM-MVS-based product generation. However, as noted by the authors, the cleaning processes include many iterative error reduction steps and have thus been proven to be very time-consuming. Furthermore, the improvement of the camera model is based on a trial-and-error procedure to find the best cleaning thresholds. This task is often cumbersome and requires persistent attendance from the operator. To reduce the operator's efforts and time expenditures on the camera model improvements, we here present a python script to automate the sparse cloud cleaning procedure in Agisoft Metashape Professional as detailed by Over et al. [20] and test it on two datasets. We will show that our script achieves similar error reduction results as manual cleaning efforts, significantly reducing the time expenditure required from the operator and avoiding the risk of inaccuracies caused by negligence during the manual iterative cleaning workflow.

## Tie point cloud cleaning procedures as suggested by the USGS

The sparse cloud cleaning workflow suggested by Over et al. [20] includes four tie point filtering steps (for an overview, see their Figs. 1 and S1) with the overall aim to filter out low-quality tie points and to optimize the camera model. These error reduction steps generally include the iterative selection and removal of tie points by (1) reconstruction uncertainty, (2) projection accuracy, and (3) reprojection error attached to the individual points in the sparse cloud. All of these filtering criteria are unitless. In short (see [20], for a detailed description), the cleaning steps are implemented in order (1) to clean the sparse cloud from tie points that were created from images with adverse geometric relationships to each other (e.g., spatially very close camera positions), (2) to remove tie points whose corresponding key points have a low precision in their spatial location (i.e., a large key point size), and (3) to remove tie points with a large spatial offset between their original location in 3D space on an image and their position on the image as estimated by reprojection (cf. [24]). Besides their workflow suggestions, Over et al. [20] provide concrete recommendations for target values and value ranges to be achieved by removing a certain fraction of points per iteration as summarized in Table 1.

**Fig. 1:**
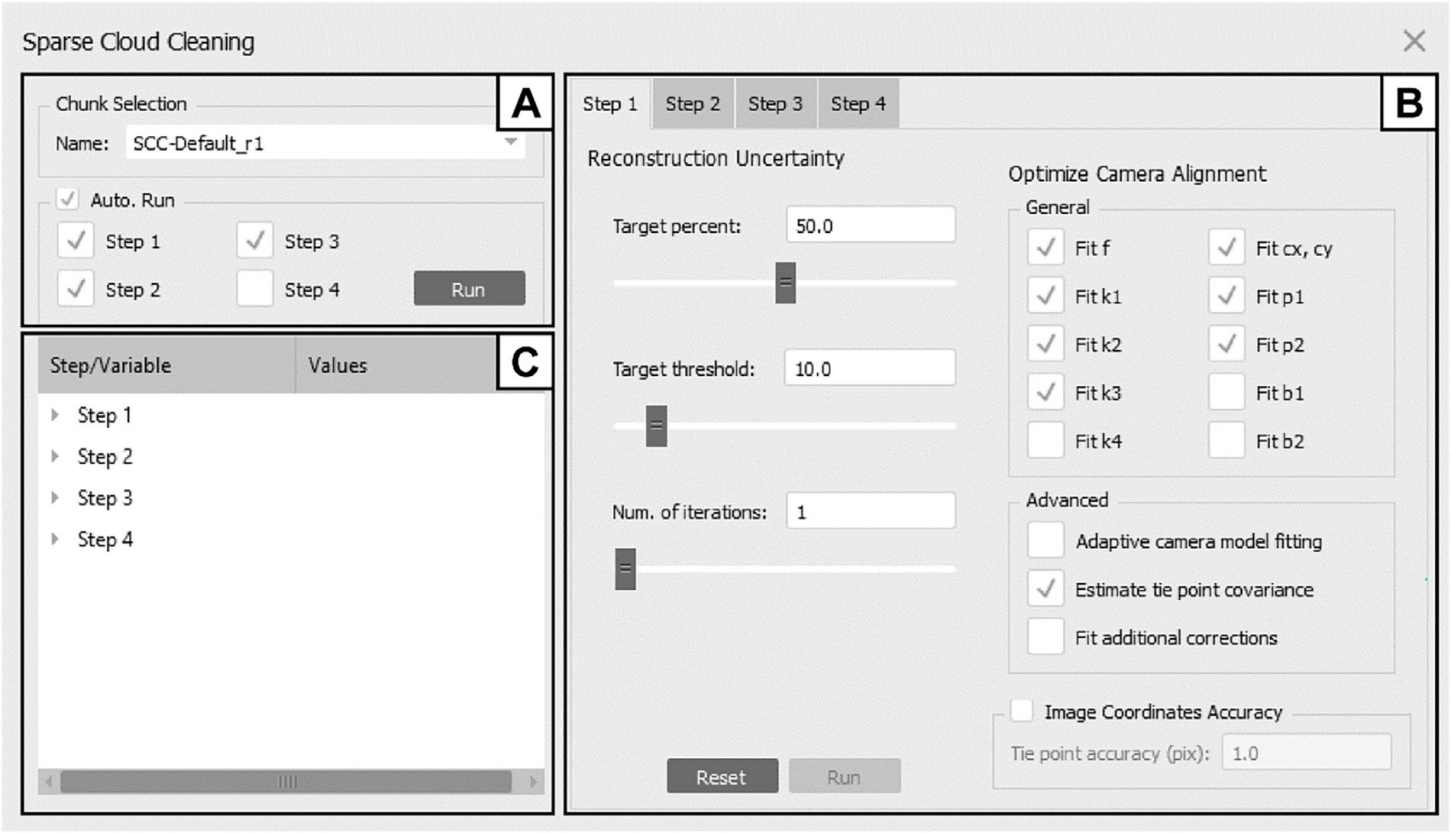
Script graphical user interface. The interface consists of three main sections allowing the processor to select the chunk, to choose which error minimization steps should be included into the cleaning procedure and whether an automatic run should be executed (A), to adjust filter and camera alignment optimizations settings of each individual filtering step (B), and to monitor quality measures (C; see also Fig. 2).

**Table 1:** Details on error reduction steps as proposed by Over, et al. [20].

Error reduction step	Point filter criterion	Maximum fraction of points to be removed per iteration (%)	Maximum threshold value	Target tie points
1	Reconstruction uncertainty	<50	10–15	Points resulting from poor camera geometry
2	Projection accuracy	<50	2–5	Points with large key point size
3	Reprojection error	10	0.3	Points with poor spatial matching of original and reprojected location in 3D space
4	Reprojection error	10	<0.3* *RMSE 0.13–0.18 px	Points with insufficient spatial matching of original and reprojected location as captured by the unweighted RMS reprojection error of the point cloud

For example, it is suggested that a maximum of 10% of points should be removed per iteration in cleaning step 3 until a maximum tie point reprojection error within the sparse cloud of 0.3 is reached (reprojection error values are normalized by the sizes of corresponding key points). As noted by Over et al. [20], a manual iteration process by repeated filtering of 10% of the tie points remains highly time consuming. Furthermore, to achieve a timely completion of a full cleaning circle, permanent attention of the processor is required. The python script we present here aims at alleviating this iterative cleaning by automating the processing steps. Over et al. [20] also provide a detailed description of the most important quality measures for the error reconstruction procedure as provided by the software (Table 2). The list of variables (see Sect. 3.2 for more details) includes simple measures such as the unweighted root mean reprojection error of the tie points and root mean square control and check point errors, but also the number of images with less than 100 projections. The latter is important as images with less than 100 valid tie points will not be used for depth maps generation in subsequent steps [24].

**Table 2:** Details on error reduction variables as implemented in the script.

Error reduction variable		GUI abbreviation	Unit	Description
Number of iterations	(*n_I_*)	Num. iterations	–	Count of iterations performed
Number of tie points	(*n_P_*)	Num. points	–	Count of tie points
Unweighted RMS reprojection error	(*RMSE*)	RMSE	px	Unweighted root mean square reprojection error of the point cloud
Standard error per unit weight	(*SEUW*)	SEUW	–	SEUW (optimum: 1) estimates the tie point accuracy
Camera error	(*e_cam_*)	Camera error	m	Root mean square error for all cameras in 3D space
Control scale error	(*e_s,co_*)	Control scale error	m	Root mean square error of all control scale bars (individual scale bar errors calculated as difference between input and estimated scale bar length)
Check scale error	(*e_s,ch_*)	Check scale error	m	Root mean square error of all check scale bars (individual scale bar errors calculated as difference between input and estimated scale bar length)
Control point error	(*e_p,co_*)	Control point error	m	Root mean square error for all control points in 3D space
Check point error	(*e_p,ch_*)	Check point error	m	Root mean square error for all check points in 3D space
Error reduction level	(*L*)	Level	–	Error reduction level approached after an error reduction step has been performed (<10 points above a pre-set threshold allowed)
Number of images with less than 100 projections	(*n_i<100_*)	Num. proj. < 100	–	Count of images that have too few valid tie points (i.e. less than 100) to be included into the generation of camera model derivations
Number of reversals	(*n_R_*)	Rev. it.	–	The number of iterations after which the number of points above the error reduction level increased (≥ 0 reversal points)
Number of reversal points	(*n_P,R_*)	Rev. pts.	–	The cumulative number of points of all reversals

## Method details

### Execution requirements

Metashape Professional allows to run codes written in Python 3.5 with its own built-in Python interpreter. The imported functionalities of the script rely on the Python standard library and the standalone Metashape module, which provides access to the main routines for processing project data. The GUI was constructed using the PySide2 module. As all these modules are readily distributed with Metashape Professional, no additional modules need to be installed for execution.

### Script functionality

To automate the iterative error reduction, all the required functionalities of the Metashape Professional software are collected in a single script. The specific settings of each step can be adjusted through a GUI. To access the script in the Agisoft Metashape Professional environment, it can either be imported from the console panel or copied to the script autorun folder as created during the installation of the software, allowing it to call the script on start-up [24]. After the script has been imported, its GUI can be accessed via the “SCC” button in the menu bar (abbreviation for “Sparse Cloud Cleaning”).

The GUI design aims to provide a clear overview on adjustment settings and quality measures and consists of three sections (Fig. 1). In section A (Fig. 1A), located in the upper left, a drop-down menu allows the user to select the chunk to be subjected to the cleaning procedure. The “Auto. Run” panel features options to execute an automated cleaning, including different cleaning steps that can be checked or unchecked individually. By default, the first three consecutive cleaning steps are selected, and hence the script will automatically clean the tie point cloud starting with filtering by tie point reconstruction uncertainty (hereafter termed “Step 1”), followed by filtering by projection accuracy (“Step 2”) and reprojection error (“Step 3”) during a run. The default cleaning does not include the unweighted RMS reprojection error optimization (“Step 4”) whose execution heavily depends on previous cleaning performance and its execution may not be regularly performed (cf. [20]; see below). Before initiating the cleaning process, a pop-up window opens, prompting the operator to double-check whether the input chunk has been correctly selected.

Section B, located on the right side of the window, allows the user to adjust the cleaning parameters (Fig. 1B). Individual tabs for each of the error reduction steps (see Table 2) allow the operator to adjust different parameters affecting the specific filtering procedure and camera alignment. The tab structure of the individual steps is similar, with the difference that the “Target threshold” parameter (towards which the final error reduction level *L* will have approached after cleaning) refers to the specific filter criterion. Each step can be run individually, provided that the “Auto Run.” option in section A is deselected.

The default values provided for the different parameters are based on the suggestions of Over et al. [20]. For Step 1, a “Target percent” value of 50.0, a “Target threshold” of 10.0, and a maximum “Num. of iterations” (iteration count, manifested as number of iterations *n_I_* after cleaning) of 1 are set by default. This combination of parameters means that a maximum of 50% of all tie points will be removed per iteration (a single run by default), until the maximum reconstruction uncertainty of the tie points in the sparse cloud has approached a value of 10. We define the approach of any target threshold to be successful if less than 10 points remain above that threshold after a full iteration is performed. To ensure that the target threshold is not undercut by excessive removal of points, it is iteratively approached in steps of 0.01%. While this method increases the overall processing time as compared to the implementation of larger-sized steps, a sufficient level of accuracy with respect to the approach of the target threshold is achieved. Note that Over et al. [20] do not recommend to execute more than one filtering iteration if a (maximum) reconstruction uncertainty value of 10 is reached without deleting more than 50% of all tie points.

Similar constrains apply to the Step 2, where the default values for maximum projection accuracy are 50.0% for “Target percent”, and 3.0 for “Target threshold”. As Over et al. [20] point out, some projects may not tolerate a maximum projection accuracy even below 6, and repeated filtering efforts after *L* = 3 is reached may lead to an overfit of camera parameters. Hence, we leave a default value of 1 for “Num. of iterations”.

Step 3 filters tie points based on point reprojection error. Over et al. [20] suggest to remove a maximum of 10% of all points with reprojection errors above *L* = 0.3, until the threshold is approached. Given this less rigorous procedure to approach the threshold, a larger number of iterations is required (200 by default). Over et al. [20] suggest to execute a further point cloud cleaning step for projects with sufficient quality measure values obtained after Step 3 has been conducted. Favoring preconditions include that default target values in Steps 1 and 2 could be reached in a single iteration each, and a sufficient number of cameras would have remained for generating the derivations based on the camera model after Step 3. Furthermore, there should be no evidence for model overfitting, and more than 15–20% of the initial tie points should be left.

Step 4 may be executed if the operators decides that the requirements mentioned above are met to a reasonable degree. In this step, default cleaning is defined as the lowering of the maximum reprojection error by a maximum of 10% of all points in the point cloud per iteration (max. number of 200 by default), until the unweighted RMS reprojection error drops below 0.18 px (cf. [20]).

In each of the error reduction steps, an optimization of the camera parameters is calculated after each iteration based on the filtered point cloud, and all measures (e.g. number of points above the threshold) are calculated after the optimizations. The operator can control which of the camera parameters should be considered in the optimization process separately for each step in the “Optimize Camera Alignment” panel on the right side of the window (Fig. 1B). By default, the standard camera parameters are selected and the point covariance is estimated during the optimization to vectorize the uncertainty of bundle adjustment transformation in the model view, indicated by the largest covariance of camera parameters for each point [24]. “Tie Point Accuracy” can also be adjusted, but it is recommended to change this value only after Step 3 has been executed [20].

**Fig. 2:**
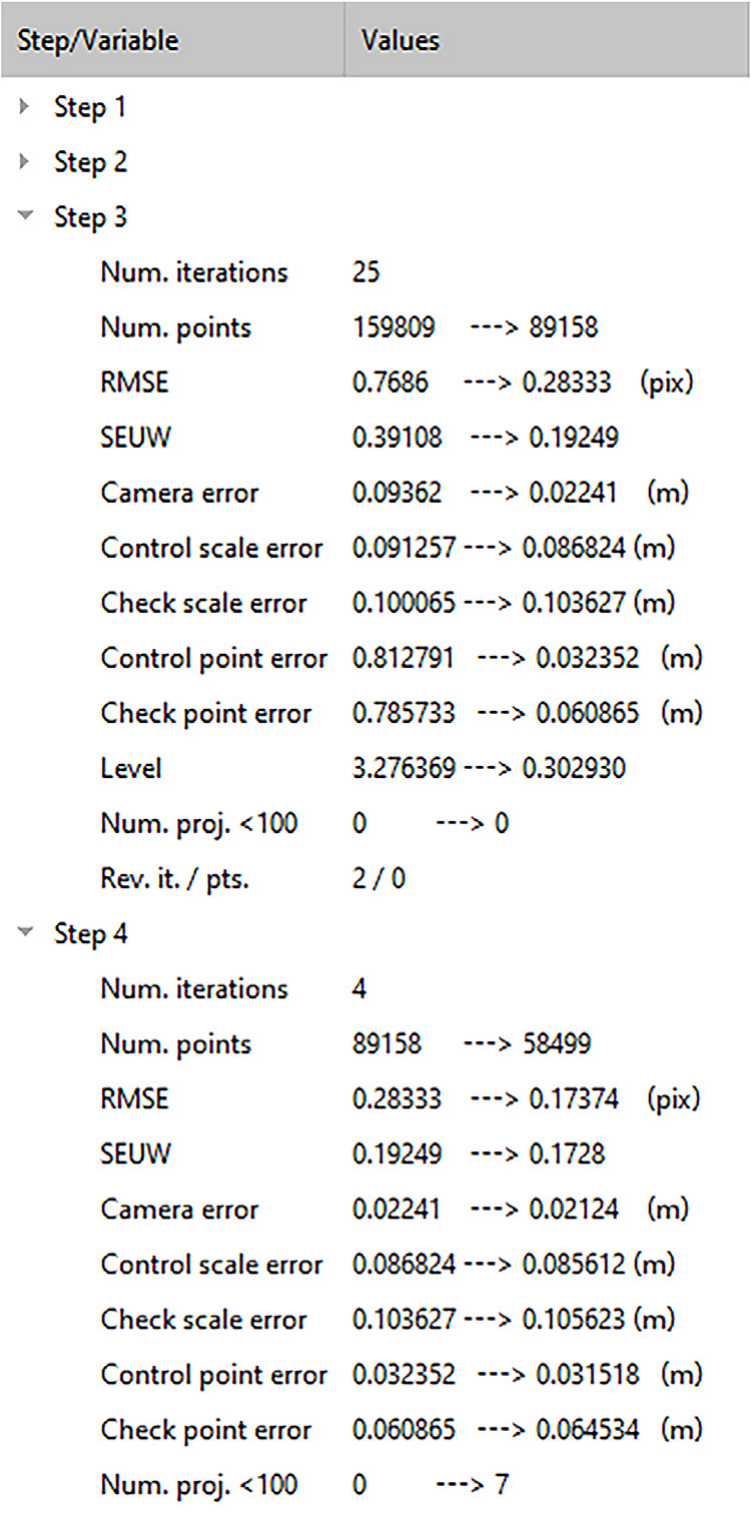
Quality measure monitoring in the third section of the top menu, i.e. (C) in Fig. 1. For more details see Table 2.

After an error reduction step has been performed, main reconstruction quality measures are presented in the third section of the GUI, which is located in the lower left corner of the window (Figs. 1C and 2). Pre- and postfiltering values calculated for the different quality measures are displayed, allowing to monitor the cleaning process (e.g. to identify indications for camera model overfitting; cf. [20]). Included are a count of iterations *n_I_* performed for the respective cleaning step (“Num. iterations”; GUI abbreviations given in brackets in the following lines of text; Table 2), the number of remaining tie points *n_P_* before and after the filtering (“Num. points”), unweighted root mean square reprojection error *RMSE* (“RMSE”), standard error per unit weight *SEUW* (“SEUW”), camera error *e_cam_* (“Camera error”), control and check scale bar errors *e_s,co_* and *e_s,ch_* (“Control scale error”, “Check scale error”), control and check marker errors *e_p,co_* and *e_p,ch_* (“Control point error”, “Check point error”), approached error reduction level *L* (“Level”), the number of images n_i<100_ that have less than 100 projections (“Num. proj. <100”), the number of iterations *n_R_* after which the number of points above the error reduction level has increased, and the corresponding cumulative number of points *n_P,R_* added during each reversal (*n_R_* and *n_P,R_* are both displayed in a single line, “Rev. it. / pts.”). Note that for Step 4, *L* equals *RMSE* and is thus not shown in the GUI (i.e., no entry for “Level”). Since in that particular step the error reduction relies on the RMSE and not on a maximum value error as valid for the previous steps, calculating *n_R_* and *n_P,R_* is not straightforward and hence also not listed in the GUI of Step 4.

**Fig. 3:**
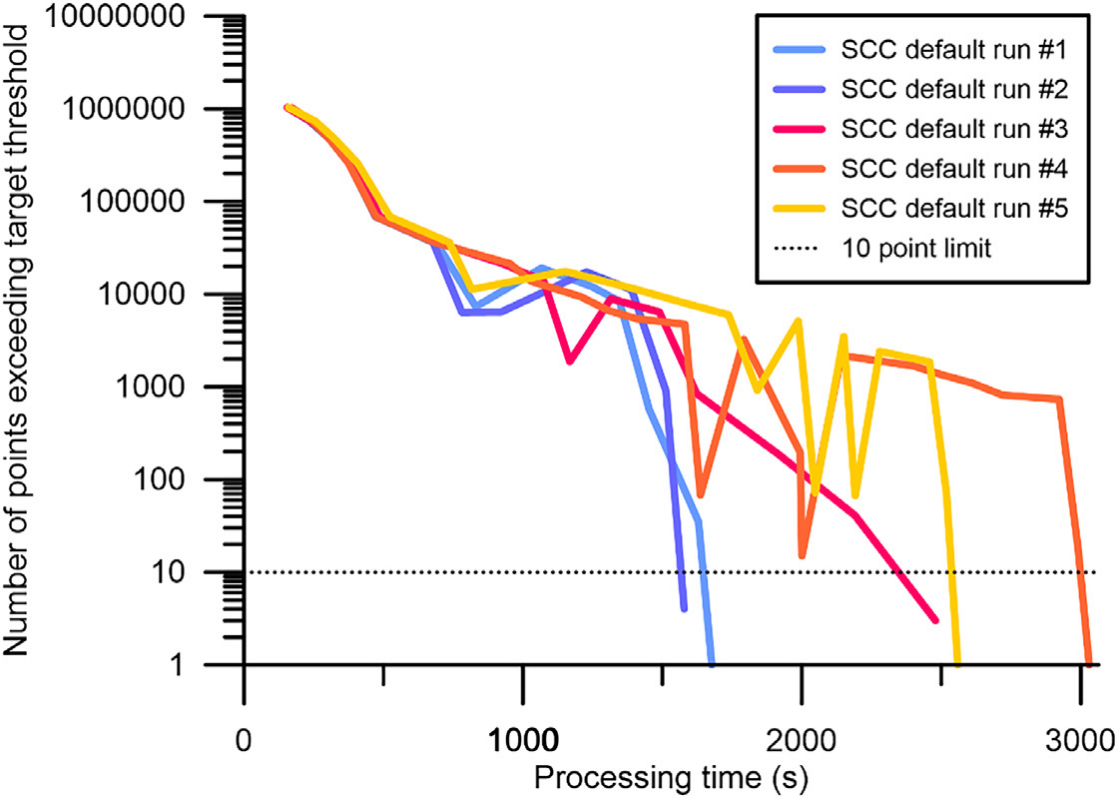
Automated cleaning pathways during Step 3, after Step 2 had yielded identical results for all repetitions providing similar initial starting positions for the individual runs (“SCC-Default” runs executed for the Lucia project of [13]). The dashed line indicates the limit of 10 points above the target threshold, marking the level below which the automated cleaning stops. Finally calculated value ranges for *n_I_, n_R_*, and *n_P,R_* are 12–24 iterations, 1–4 reversals, and 5351-16358 reversal points, respectively. Likewise, the processing time required to execute this step ranged between ∼27 min and ∼51 min. See Fig. S2 for a similar plot for the Urft project.

During manual cleaning, high values for *n_R_* and *n_P,R_* would represent a warning signal for the operator to stop or adjust further filtering efforts, as they may indicate overfitting of the camera model (cf. [20]). Hence, both *n_R_* and *n_P,R_* were included to the list of quality measures. Displaying these variables can help to evaluate how reasonable the automated cleaning may have been, complementing the information on camera model refinement iterations as natively performed by the software during the camera optimization and as displayed in the console panel (represented by a string consisting of multiple “x” and “!”; for more detail see [20]). Because the camera models are iteratively optimized by numerically solving the lens coefficients (cf. [25]), camera models and filtering pathways computed from the same image datasets can differ (Fig. 3). Although we generally do not find these differences to be significant for our projects (see method validation section), such additional information may be useful to increase comparability between optimized camera models based on the same initial point cloud within the black-box environment of Agisoft Metashape Professional. *n_P,R_* is calculated by adding up those points that contributed to the increase of points above the chosen error reduction level, when compared to the previous iteration. For example, if the number of points above the chosen error iteration level was 100 after the first iteration, but this number increased to 110 after the second and 150 after the third iteration, then *n_R_* = 2, and *n_P,R_* = 60. Note that the addition of points in the latter variable does not mean that points are added to the sparse point cloud. The optimum that can be resolved by this approach are values of zero for both the number of reversals and consequently the number of points added up after these iterations. Iterations that result in *n_P,R_* = 0 are included to the total count of reversals, because the point distance to the error reduction level is not further approached during this iteration. Such a detailed assessment of the camera model optimization performance could also play a role in those cases where a few points difference would imply the removal of images from building derivations because n_i<100_ increases. We also add supporting information characterizing the cleaning process to be displayed in the console panel of the Metashape software. During the processing, the number of tie points above the target threshold, the iteration count and the filter level approached before and after camera optimization are displayed. After a cleaning step has been completed, values obtained for *n_I_, n_R_, L* and *n_P_* are presented. Furthermore, the change in numbers of points above the threshold after each iteration is shown, which basically gives an idea of the cleaning progress (cf. Fig. 3) and from which *n_P,R_* is calculated. For Step 4, information on *RMSE* and *n_I_* are provided.

The cleaning processing preferences and quality measures for each step are stored in a JavaScript Object Notation (JSON) file in the project directory. The JSON files represent the memory of the SCC script, and a file is created whenever the operator modifies the default values in the script and/or performs the error reduction process. Each JSON filename contains the project's name and a timestamp. When calling the script in Agisoft Metashape Professional, the project directory is scanned for record files. If records exist, the operator can choose to restore a session from a drop-down menu to have the processing preferences and quality measures displayed for the individual chunks.

### Method validation

To assess the performance of the SCC script, we used real-world data obtained from three different projects, as detailed in the following section. For the initial camera models of all projects, we used camera alignment preferences as suggested by Over et al. [20], i.e. a maximum of 60,000 key points and no tie point limit assigned per image. The alignment was performed at a high accuracy with stationary tie points being excluded. The camera models relied on camera self-calibration adjusting the standard parameters (focal length, principal point position, three radial and two tangential distortion coefficients). All computations were conducted using a Dell Precision Tower 3620 (3.60 GHz processor, 64GB RAM, Intel HD Graphics 630 on board and NVIDIA Quadro M4000). We tested the script using Microsoft's Windows 10 Pro 64-bit operating system (ver. 22H2), keeping as few background programs as possible and Microsoft's Office running during the processing. Testing our script involved “Default” cleaning defined based on the recommendations Over et al. [20] and subsequent supposedly “Optimized” runs where we changed the number of iterations allowed in Step 1 and/or Step 2 (Table 3). Finally, we executed runs to minimize *RMSE* (“RMSE_m_”). To assess the reproducibility of our automated camera model optimization efforts, we repeated each automated run five times and calculated the arithmetic means and standard deviations for each step. One run per project was manually repeated serving as benchmark for quality measures and processing duration. We here do not report measures for *e_cam_* since the images were either not geotagged (BA18–008 and Lucia projects) or not used for camera model building due to large vertical offsets (Urft project; cf. [26]). Furthermore, we did not adjust the tie point accuracy in Step 4 as recommended by Over et al. [20] to more conveniently track the change of *SEUW* during the cleaning process (changing the point accuracy will affect *SEUW*).

**Table 3:** Overview on runs executed on the three projects.

Project (type)	Run	*n*	Target values Step 1			Target values Step 2			Target values Step 3			Target values Step 4		
			*n_P_*			*n_P_*			*n_P_*			*n_P_*	*RMSE*	
			(%)	*L*	*n_I_*	(%)	*L*	*n_I_*	(%)	*L*	*n_I_*	(%)	(pix)	*n_I_*
BA18–008 (close range, lightbox)	SCC-Default	5	50.00	10.00	1	50.00	3.00	1	10.00	0.30	200	10.00	–	–
	SCC—Optimized	5			2		2.00	2					–	–
	SCC-RMSE_m_	5			2		3.00	1					0.18	200
	Manual-Default	1			1		3.00	1					–	–
Lucia (drone; [13])	SCC-Default	5			1		3.00	1					–	–
	SCC—Optimized	5			2		2.00	1					–	–
	SCC-RMSE_m_	5			2		2.00	1					0.18	200
	Manual-Default	1			1		3.00	1					–	–
Urft (drone; [26])	SCC-Default	5			1		3.00	1					–	–
	SCC—Optimized	5			1		2.00	1					–	–
	SCC-RMSE_m_	5			1		2.00	1					0.18	200
	Manual-RMSE_m_	1			1		3.00	1					0.18	200

### Test project descriptions

The first test project represents the digitalization of a CaSO_4_ crust sample (sample ID: BA18–008) taken from the Atacama Desert (for more details on the study area see [11]). The sample was pictured in the GeoMuseum-PL photogrammetry laboratory of the Institute of Geology and Mineralogy, University of Cologne (Fig. S3). Image acquisition was performed on a turntable in a lightbox using a SONY NEX-7 camera (APS-C sensor) with a f/1.8 35 mm (52.5 mm in 35 mm film equivalent) fixed focal lens mounted. For further details on equipment and shooting strategy, the reader is referred to the supplement. The initial camera model of the lightbox test project had 996,690 tie points based on 196 images. Out of four 5 cm scale bars placed nearby the specimen, two were used as check scale bars.

The second and third test projects represent landscape-scaled reconstruction scenarios investigated by Sanz-Ablanedo et al. [13] and Stauch et al. [26]. The authors kindly provided their raw data (i.e., imagery and ground control points, hereafter abbreviated GCPs). Here we clarify that our intention is to test the functionality of our script and we do neither attempt to challenge any previously reported results nor do we focus on finding the optimal cleaning/model building solution based on the data we have.

Sanz-Ablanedo et al. [13] acquired a large dataset consisting of drone images without geotags and 110 GCPs measured at accuracies < ∼7 cm (hereafter referred to as “Lucia project”, Fig. S4). The imagery was captured in the highland and mining area near the Spanish village Santa Lucia de Gordón (42.9°N, 5.6°W) in 2015 using a Samsung NX500 camera with a Samsung NX 20 mm f/2.8 lens mounted, which were attached to a 2 kg fixed-wing unmanned plane [13]. According to the authors, the flight altitude was 120 m above the flight control station but the vertical distance to the ground varied significantly during the flights due to the rough topography; the mean ground sampling distance (GSD) was 6.86 cm. The images were captured both in nadir and oblique [13]. Using ArcGIS Pro-software (ver. 3.1.2), we applied a spatial filter with a search radius of 350 m to all GCPs to semi-randomly convert half of the control points to check points (*n* = 55). As suggested by Sanz-Ablanedo et al. [13], we assigned a virtual camera to each of the six flights to account for possible alterations in the internal camera geometry after each flight. Our initial image alignment efforts resulted in a tie point cloud of 11,2527,058 points (2582 images aligned).

The last project tested in this study (“Urft project”) builds on a comprehensive drone imagery and GCP dataset provided by Stauch et al. [26], who investigated spatiotemporal patterns of sediment deposition in the Urft Reservoir close to the village of Gemünd in Germany (50.6°N, 6.4°W) during the 20th and 21st century. The dataset consists raw data of their second drone survey (conducted in December 2021), including 1527 geotagged drone images and 171 GCPs (Fig. S5). The GCPs were measured at 1–2 cm horizontal and 2–3 cm vertical localization accuracies, respectively [26]. Drone images were taken using a DJI Phantom 4 drone equipped with a FC6310S camera (1” CMOS sensor, f/2.8–11 24 mm in 35 mm film equivalent; for further details see their supplement data). The authors applied an imaging strategy aiming to minimize systematic topographic errors by capturing images in a double grid pattern, with 70% frontal image overlap and 10° nominal camera angle at elevations of 90 m and 120 m above takeoff position. The GSD achieved was 2.49 cm (the final digital surface model can be obtained from [27]). We obtained the original check point configuration as used by Stauch et al. [26], implying that half of the GCPs were used as control points. Image alignment (twelve flights assigned to individual camera groups but processed in a single chunk) resulted in a tie point cloud of 3897,864 points (1512 images aligned, without considering geotagging information).

### Script performance

The overall results obtained from the in replicate performed camera model optimization runs indicate a high degree of reproducibility, with the largest standard deviations generally being less than 1% for all quality measures (Table 4 and S1; illustrated in Fig. S6). An exception made Step 1 in two of the cleaning runs (“SCC-Default” and “SCC—Optimized”) performed for the Urft project, where the standard deviation for *L* was about 100%. The high standard deviation arose from a few replicates where *L* equaled values of ∼85 and ∼40 instead of 10. These high values (representing the maximum reconstruction uncertainty within the point cloud) were carried by a single point in these replicates and had no effect on the overall error reduction (all replicate runs approached identical values for *n_P_* and *L* after Step 2; Tab. S1 and S2).

**Table 4:** Results of different camera model optimization runs. Results from automated runs are presented as arithmetic means, standard deviations are shown in brackets.

Project (type)	Run	n_P_	RMSE	SEUW	e_s||p,co_	e_s||p,ch_	n_i<100_	t
		(%)	(%)	(%)	(%)	(%)	increase	(s)
BA18–008 (close range, lightbox)	SCC-Default	19.18 (0)	39.61 (<0.01)	85.90 (<0.01)	45.00 (0)	107.30 (0)	0 (0)	211 (7)
	SCC—Optimized	4.56 (0)	36.97 (0)	87.48 (0)	20.00 (0)	125.40 (0)	1 (0)	188 (30)
	SCC-RMSE_m_	1.44 (<0.01)	20.55 (0.01)	46.51 (0.03)	34.00 (2.24)	143.81 (1.42)	22 (1)	546 (4)
	*Manual-Default*	*19.13*	*39.57*	*85.91*	*45.00*	*107.94*	*0*	*448*
Lucia (drone; Sanz-Ablanedo et al. 2018)	SCC-Default	15.69 (0.11)	9.42 (0.05)	96.31 (0.52)	2.71 (0.07)	17.11 (0.21)	67 (3)	3237 (649)
	SCC—Optimized	15.31 (0.19)	9.37 (0.08)	97.66 (0.94)	2.77 (0.11)	17.02 (0.24)	70 (4)	3164 (612)
	SCC-RMSE_m_	6.41 (0.21)	5.08 (0.07)	76.67 (0.73)	1.12 (0.05)	20.20 (0.23)	434 (5)	11,890 (1290)
	*Manual-Default*	*15.70*	*9.42*	*96.24*	*2.71*	*17.10*	*66*	*2354*
Urft (drone; Stauch et al. 2023)	SCC-Default	42.09 (<0.01)	39.24 (<0.01)	86.50 (0.01)	86.77 (0.01)	99.99 (<0.01)	51 (0)	1226 (41)
	SCC—Optimized	31.46 (<0.01)	36.52 (<0.01)	88.59 (0.01)	83.23 (0.01)	99.98 (<0.01)	72 (0)	1205 (52)
	SCC-RMSE_m_	20.64 (<0.01)	21.17 (<0.01)	61.11 (0.01)	69.69 (0.01)	99.95 (<0.01)	139 (0)	2549 (86)
	*Manual-RMSE_m_*	*21.01*	*21.39*	*61.38*	*69.93*	*99.95*	*137*	*1924*

Slightly elevated standard deviations (< 3%) were also obtained for *e_s,co_* and *e_s,ch_* as recorded during “SCC-RMSE_m_” runs performed on the turntable project. This pattern is largely related to the fact that errors reported in this project are in the lowest precision range the software can provide, i.e. at the micrometer scale. The apparent lack of precision does affect the calculations, but as a workaround we scaled the model up by a factor of 100 and obtained standard deviations reduced to about 1% and 1.6%. However, the camera parameter correlations also indicate overfitting (see supplement), likely further contributing to the slightly scattered results. Lastly, standard deviations of about 5% are evident for n_i<100_ after the automated default and optimized runs executed on the Lucia project. Such scatter is likely to be related to the variability in *n_I_, n_R_*, and *n_P,R_* (Figs. 3 and S2), governing the removal of images that are close to the limit of 100 projections.

When compared to the manually performed benchmark tests, approached quality measures generally differ by less than 1%. Slightly elevated differences of about 1% are evident for the RMSE_m_ runs performed for the Urft project, which is likely caused by two factors. Firstly, the filtering of a precisely defined number of points – e.g. 10% of all points – remains cumbersome in those cases where the chosen target percent value is insufficient to approach the target threshold when using the gradual selection tool as implemented in Agisoft Metashape. From our experience we assume that most operators will try to approach the value as close as possible by shifting the slider to a value approximating the defined number of points to be deleted. Alternatively, the exact number of points to be filtered can be calculated manually, adding up to the overall time expenditure required. For our manual testing, we hence simply used the slider. As a consequence, the threshold may not be approached as accurately as achieved by our script in Steps 1–3. Secondly, and in contrast to the other steps, the automated filtering in Step 4 is based on a removal of a chosen target percent value without considering the approach of the target threshold. In other words, in Step 4 always the chosen relative fraction of tie points will be removed, regardless of how closely the (*RMSE*) target threshold may have been approached already. For Step 4, this procedure implies that the RMSE after cleaning may be well below the defined threshold, while a more accurate cleaning requires manual adjustments. In contrast to that, the chosen target percent in Steps 1–3 will always be the maximum value, being automatically lowered when the target threshold is in its range.

Large deviations of >10 min are further observed for the total time needed to automatically optimize the camera models of the Lucia project (Fig. 3). It is striking that for this particular project, the apparent improvement in quality measure variables is much greater than for the Urft project. The differences are most likely governed by the larger density of control and check points per image (GCPs deployed for the Lucia project: 110; Urft project: 171) and a generally smaller dataset of the Urft project (area covered in the Lucia project: 12.3 km^2^; Urft project: 0.6 km^2^). The less strict spatial confinement by GCPs in the Lucia project allows the camera model optimization to have a greater effect on the quality measures, while the Urft camera models are less likely prone to overfitting issues (see also the camera parameter correlation tables provided in the supplement). Consequently, cleaning pathways of the Lucia project are characterized by larger and more scattered values for *n_I_, n_R_*, and *n_P,R_* as compared to the Urft project (Fig. S2), contributing to the scatter in recorded processing durations. In general, the computing time required using the script can be longer than if a manual cleaning is executed (Table 4), which is however outweighed by the high accuracies achieved during the cleaning process when using the SCC script. Furthermore, the time expenditure required from the operator is significantly reduced.

## Concluding remarks on script performance

Manual cleaning of tie point clouds as suggested by Over et al. [20] can yield robust camera models, generally improving the quality of their derivations and freeing up hard drive storage space by reducing tie point cloud sizes. However, the workflow is time consuming and its reproducibility can be additionally affected by the fidelity of the operator. The automatization of the cleaning steps alleviates the efforts that are required by the operator and increases comparability of error reduction outcomes. The usefulness of the presented script increases with increasing project size (i.e., by the number of tie points), when numerous iterations need to be performed to achieve a descent camera model optimization. In many cases, the pre-set default cleaning settings can yield sufficient improvements on the camera model, but further optimization efforts are easily monitored given the simple quality measures provided in the GUI of the script. Despite the automatization, the processing is still trial-and-error-based, hence we recommend to back-up original and processed clouds until a final solution is found (cf. [20]). Future programming efforts could focus on the implementation of batch processing, the inclusion of further quality measures, and/or the possibility to optimize camera models based on pre-defined values for individual quality measures.

## Limitations

Due to substantial changes made to the syntax of the standalone Metashape Python module, the SCC script will not work with software versions preceding version 2.0. Any progress in data processing with the SCC script is saved in the form of a JSON file in the current working directory, and named according to the date and time of its creation. To ensure that the working directory is set correctly, the project should be opened by clicking on the project file (.psx) and not from the Metashape software already running. Depending on the administrative rights the user possesses on the workstation, the script may lack the necessary write permissions to create the JSON file.

When the software is run, any modification of the project structure by adding, removing and/or renaming chunks after a JSON file has been created will cause a loss of recognition when re-executing the SCC script since these modifications do not appear in the previously saved JSON file. In that case, no error message will be displayed but the respective variables will not be characterized by values. Nevertheless, all information on the quality measures can be retrieved by reading the record files in a basic text editor.

## Ethics statements

Our work did neither involved human subjects, animal experiments, nor data collected from social media platforms.

## CRediT authorship contribution statement

**Joel Mohren:** Conceptualization, Investigation, Software, Writing – original draft, Writing – review & editing. **Maximilian Schulze:** Software, Writing – original draft, Writing – review & editing.

## Declaration of competing interest

The authors declare that they have no known competing financial interests or personal relationships that could have appeared to influence the work reported in this paper.

## Data Availability

I have shared the link to my code at the Attach File step.

## Appendix Supplementary materials

Image, application 1

Image, application 2

The supplementary material includes supplementary text, figures and the processing reports generated by the software. Image, application 3
