# Supplementary material for: Automated cleaning of tie point clouds following USGS guidelines in Agisoft Metashape professional (ver. 2.1.0)
Source: MethodsX. 2024 Mar 26;12:102679. doi: 10.1016/j.mex.2024.102679 (PMC10992719; doi:10.1016/j.mex.2024.102679)
Supplement: Supplementary file 2 [file mmc2.docx]

Automated cleaning of tie point clouds following USGS guidelines in Agisoft Metashape Professional (ver. 2.1.0)

Joel Mohren^a,b^, Maximilian Schulze^b^

^a^RWTH Aachen University, Department of Geography, Wüllnerstr. 5b, 52062 Aachen, Germany

^b^University of Cologne, Institute of Geology and Mineralogy, Zülpicher Str. 49b, 50674 Cologne, Germany

**Supplementary information**

**Code availability**

Name of the code/library: SCC.py

Hardware requirements: Based on the minimal configuration as recommended by Agisoft LLC [1]:

- - Windows 7 SP 1 or later (64 bit)
  - Intel Core i7 or AMD Ryzen 7 processor
  - Discrete NVIDIA or AMD GPU (4+ GB VRAM)
  - 32 GB of RAM

Program language: Python 3.5

License: GPL (3.0)

Software required: Agisoft Metashape (tested on version 2.1.0.17530).

Program size: The script is ~80 kB

The source codes are available for downloading at the link: <https://github.com/MaximilianSchulze/metashape-scc>

**Digitalization of specimen at the GeoMuseum-PL**

The GeoMuseum-PL photogrammetry laboratory at the Institute of Geology and Mineralogy, University of Cologne is equipped with a lightbox and a turntable (both ORANGEMONKIE, Inc, USA), allowing for semi-automated (horizontal) sample rotation and imaging using infrared communication between turntable and camera. The image acquisition is performed according to an internal protocol, with regularly 24-48 pictures taken from a stationary camera position at a distance of ~30-35 cm to the specimen at an angle of ~35-45° at each shooting sequence (see e.g. [2, 3] for comparable approaches). The main interest on specimen digitalization arise from generating data for scientific publications with a main focus on volume determinations. Furthermore, we aim for digitalizing large fractions of specimen used for teaching purposes and other museum pieces. Watertight and true-volume 3D modelling is achieved by manually turning the specimen after each shooting sequence, with four coded scale bars being placed close to the specimen in the first sequence. We generally aim for at least four orientations per specimen.

**Supplementary figures**


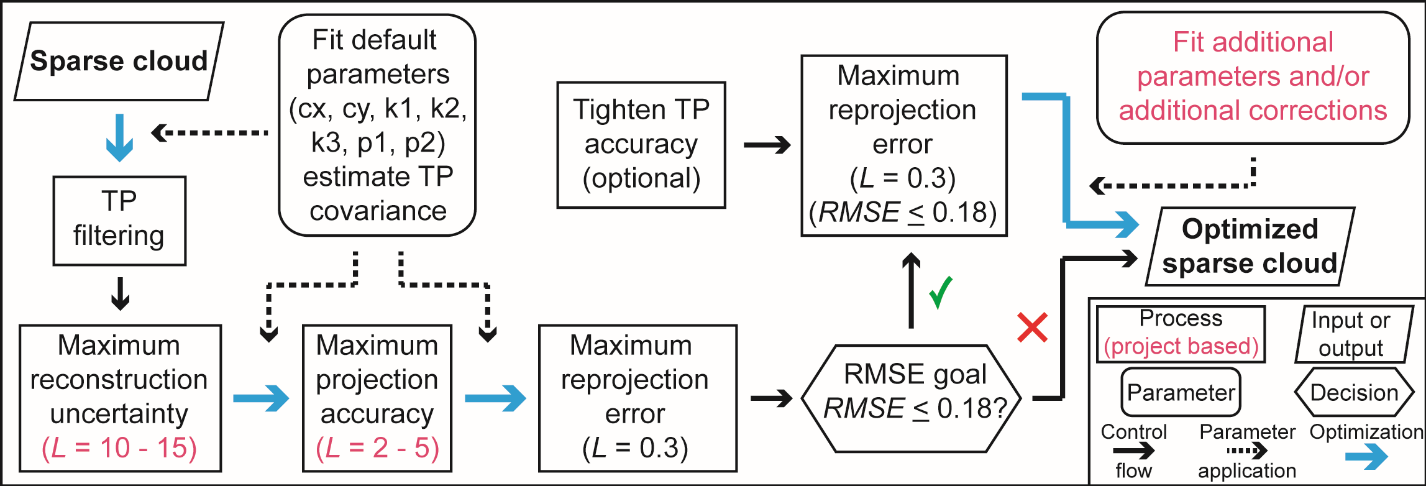


Figure S1. Sparse cloud cleaning workflow, redrawn and simplified from Over, et al. [4]. All tie point (abbreviated TP) filtering steps shown here are included in the SCC script.


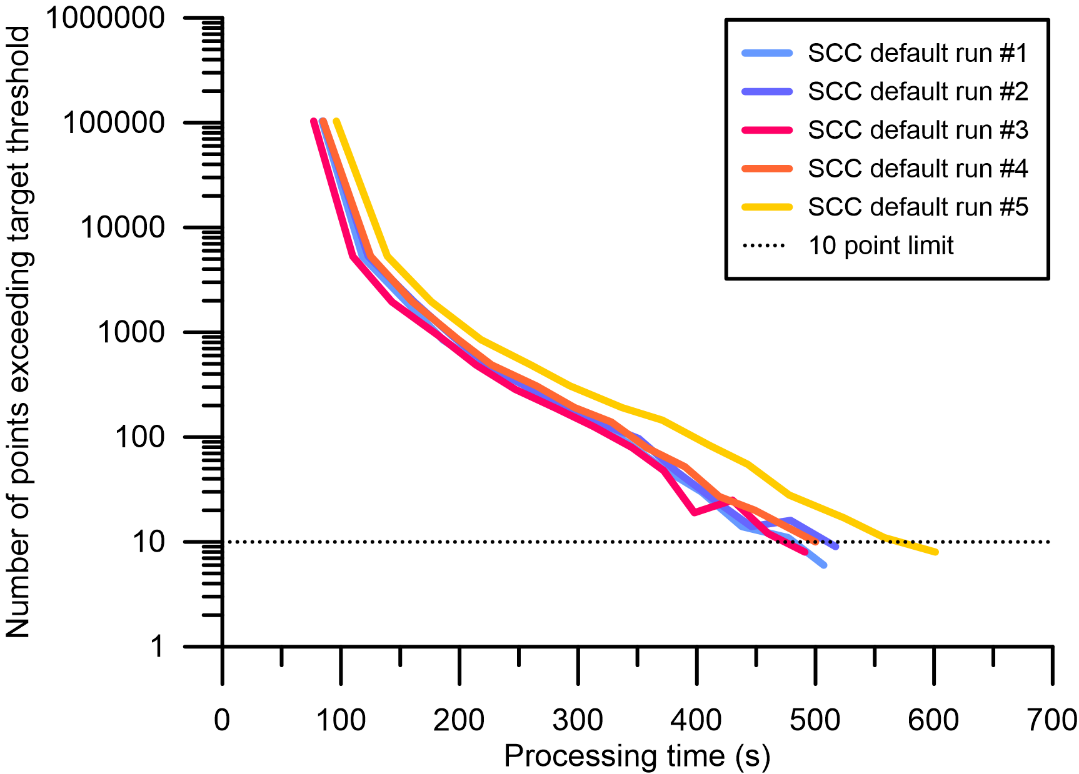


Figure S2. Automated cleaning pathways during Step 3, after Step 2 had yielded identical results for all repetitions providing similar initial starting positions for the individual runs (“SCC-Optimized” runs executed for UAS data from the Urft project of Stauch et al. [5]). Data recording as shown here began with the initial approach of the target threshold before camera optimization and ended when the threshold was approached after camera optimization. The dashed line indicates the limit of 10 points above the target threshold, marking the level below which the automated cleaning stops. Finally calculated value ranges for *n_I_*, *n_R_*, and *n_P,R_* are 13-14 iterations, 0-1 reversals, and 2-6 reversal points, respectively. Likewise, the processing time required to execute this step ranged between ~9 min and ~10 min.


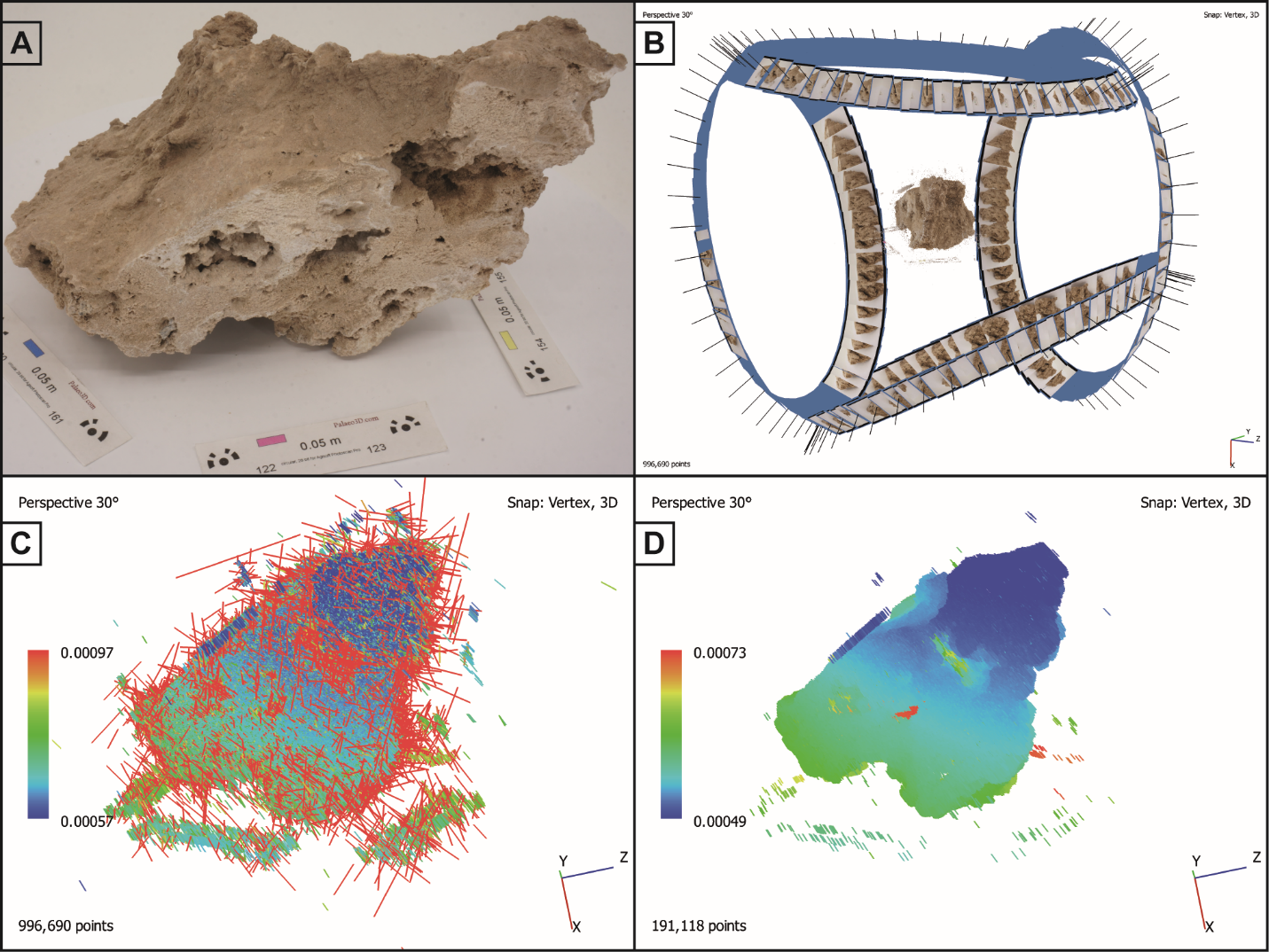
Figure S3. BA18-008 specimen digitalization and sparse point cloud cleaning. Four scale bars were placed close to the crust sample on a turntable (A). The specimen was photographed in four orientations (manually vertically turned) to obtain 196 pictures in total (B). The initial camera model (B, C) was cleaned using the SCC script (default settings), which reduced the number of tie points and generally improved the point cloud quality, as visualized by the point covariance vectors calculated for the bundle adjustment calculations (C, D). The direction and value (the color code also applies to the latter) associated to the individual vectors indicate the value and direction of the largest error for estimated tie point position in 3D space [1].

Figure S4. Camera model optimization and derivates based on the UAS dataset (“Lucia project”) provided by Sanz-Ablanedo, et al. [6]. The dataset consists of 110 GCPs distributed across the area of interest (A), and digitalization of the rough terrain based on 2582 aligned images (B). Error reduction performed on the
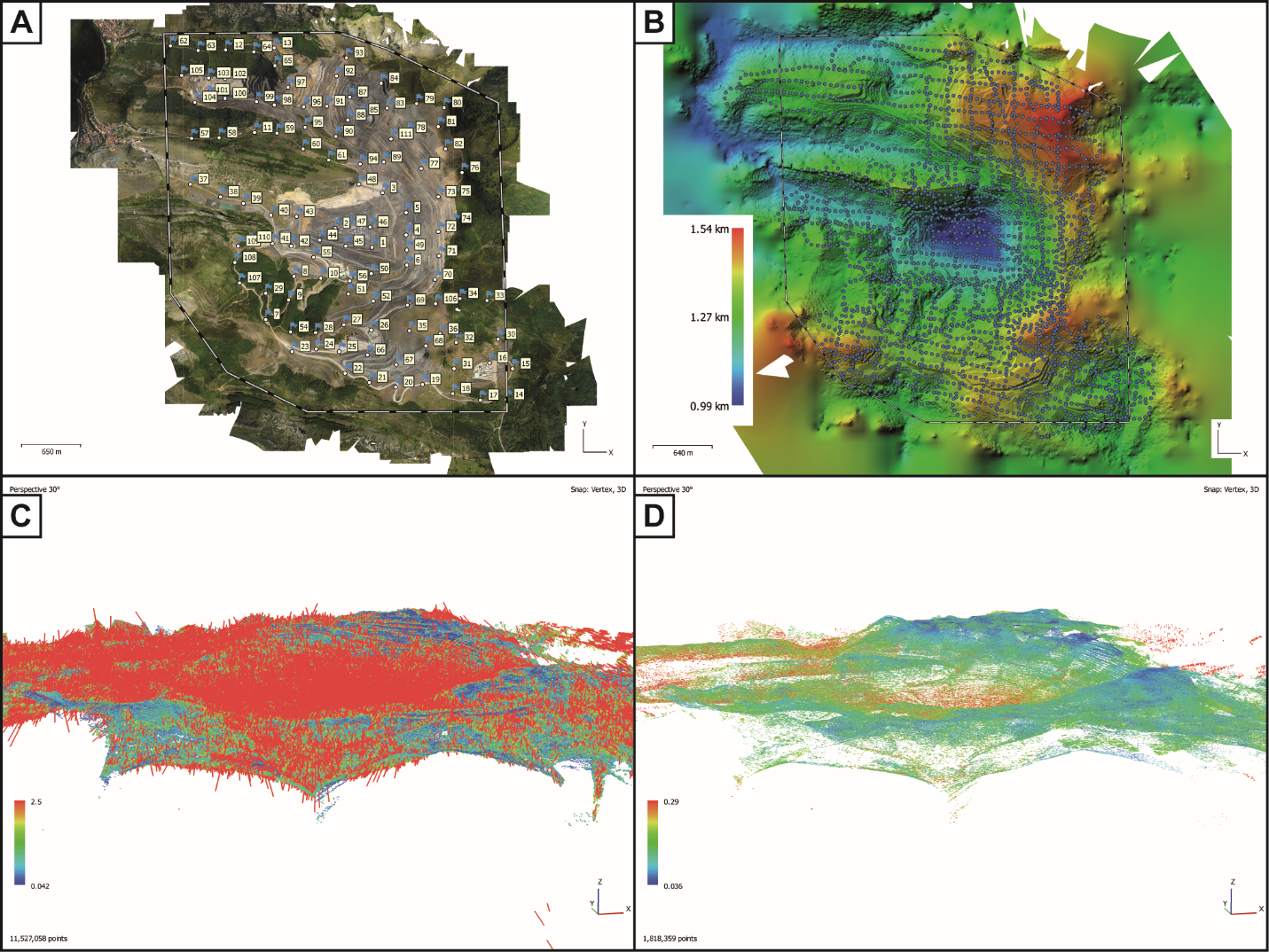
initial camera model (shown in C) using default settings reduced the number of tie points and generally improved the point cloud quality, as visualized by the point covariance vectors calculated for the bundle adjustment calculations (C, D). The direction and value (the color code also applies to the latter) of the individual vectors indicate the value and direction of the largest error for the estimated tie point position in 3D space. Both derivates (6.2 cm/px orthomosaic in A and 2 m/px digital elevation model in B) were directly obtained from the tie point cloud.


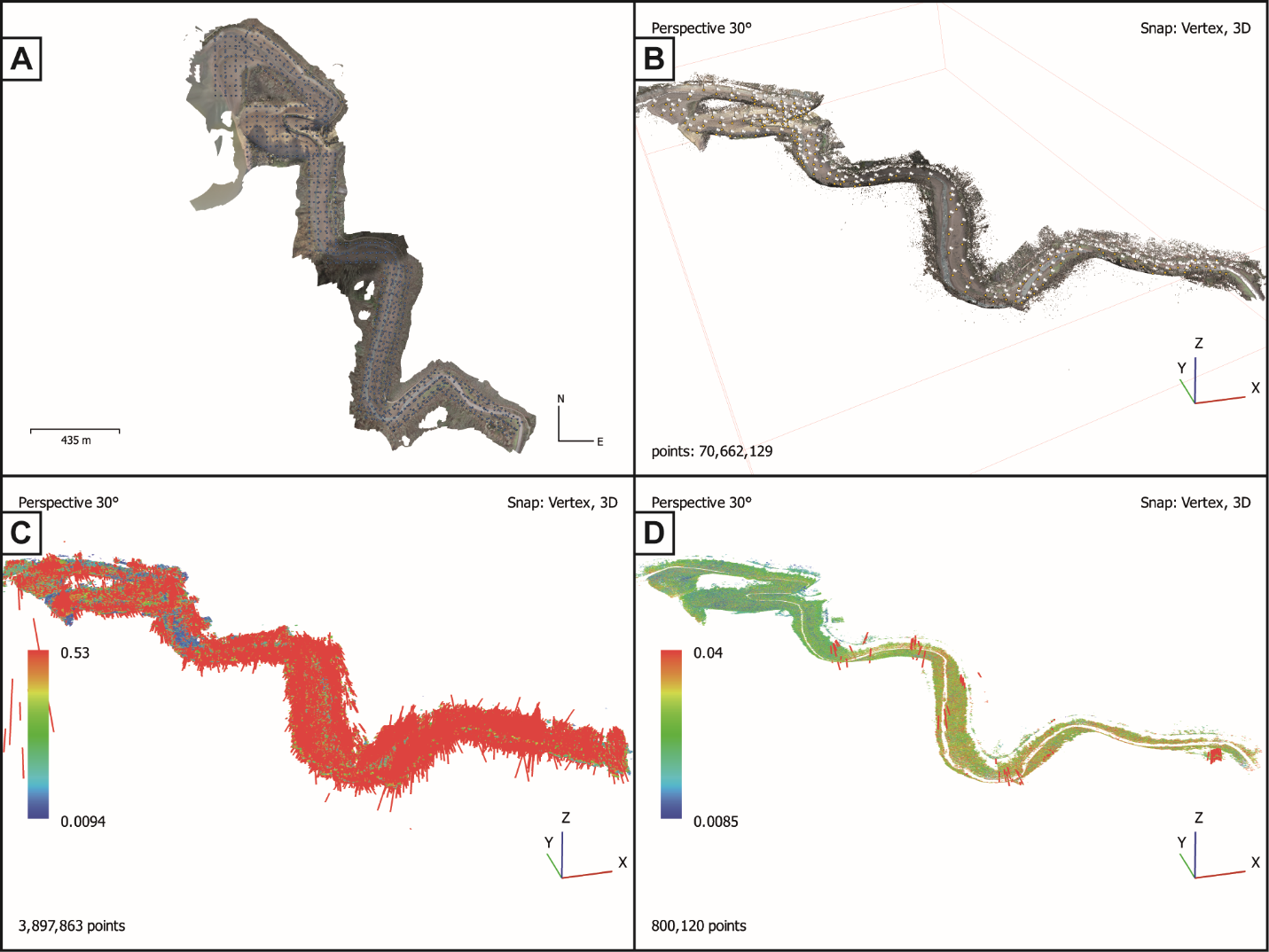
Figure S5. Camera model optimization and derivates based on the UAS dataset (“Urft project”) provided by Stauch, et al. [5]. The dataset consists of 1527 drone images captured in a double grid pattern (A). 172 GCPs were distributed along the Urft river bed (B). Error reduction performed on the initial camera model (shown in C) with the aim of minimizing the unweighted root mean square reprojection error of the point cloud. Such cleaning efforts reduced the number of tie points and generally improved the point cloud quality, as visualized by the point covariance vectors calculated for the bundle adjustment calculations (C, D). The direction and value (the latter which are also color-coded) of the individual vectors indicate the value and direction of the largest error for the estimated tie point position in 3D space. Note that in order to display tie point covariances in (D), the dataset had to be cleaned from poorly connected components (i.e. from images with non or few projections). Both derivates (25 cm/px orthomosaic in A and dense point cloud in B) were directly obtained from a cleaned tie point cloud (RMSE_m_).


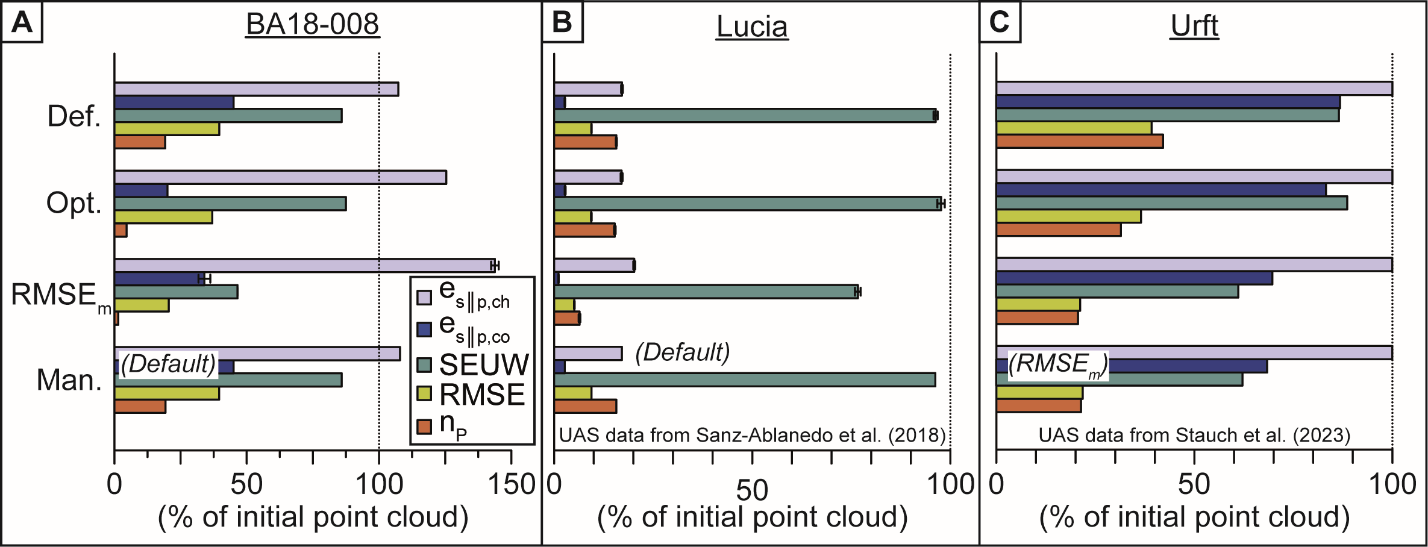
Figure S6. Graphical visualization of changes in quality measure values for different camera model optimization runs relative to the unprocessed sparse cloud (= 100%) as performed for different test projects: (A) specimen digitalization in a lightbox, (B) UAS data provided by Sanz-Ablanedo, et al. [6], and (C) UAS data provided by Stauch, et al. [5]. The results of the automated runs (Def. – default cleaning settings, Opt. – optimised, RMSE_m_ – RMSE reduction; see. Tables 3 and 4 in the main manuscript) are presented as arithmetic means of five repetitions each. Error bars represent one standard deviation from the mean. Manual error reduction was conducted to repeat one selected automated cleaning run per project (indicated in brackets). The legend and labeling on the ordinate in (A) apply to all panels.

**References**

[1] Agisoft LLC, "Agisoft Metashape User Manual Professional Edition, Version 2.0," 2023. [Online]. Available: <https://www.agisoft.com/pdf/metashape-pro_2_0_en.pdf>.

[2] G. Sorrentino *et al.*, "Close-range photogrammetry reveals morphometric changes on replicative ground stones," *PLoS One,* vol. 18, no. 8, p. e0289807, 2023, doi: 10.1371/journal.pone.0289807.

[3] M. Leménager, J. Burkiewicz, D. J. Schoen, and S. Joly, "Studying flowers in 3D using photogrammetry," *New Phytol,* vol. 237, no. 5, pp. 1922-1933, Mar 2023, doi: 10.1111/nph.18553.

[4] J.-S. R. Over *et al.*, "Processing coastal imagery with Agisoft Metashape Professional Edition, version 1.6—Structure from motion workflow documentation," US Geological Survey, 2331-1258, 2021.

[5] G. Stauch, L. Dörwald, A. Esch, and J. Walk, "115 years of sediment deposition in a reservoir in Central Europe: Topographic change detection," *Earth Surface Processes and Landforms,* 2023, doi: 10.1002/esp.5722.

[6] E. Sanz-Ablanedo, J. H. Chandler, J. R. Rodríguez-Pérez, and C. Ordóñez, "Accuracy of Unmanned Aerial Vehicle (UAV) and SfM Photogrammetry Survey as a Function of the Number and Location of Ground Control Points Used," *Remote Sensing,* vol. 10, no. 10, p. 1606, 2018, doi: 10.3390/rs10101606.
