# Supplementary material for: Automated cleaning of tie point clouds following USGS guidelines in Agisoft Metashape professional (ver. 2.1.0)
Source: MethodsX. 2024 Mar 26;12:102679. doi: 10.1016/j.mex.2024.102679 (PMC10992719; doi:10.1016/j.mex.2024.102679)
Supplement: Supplementary file 3 — The supplementary material includes supplementary text, figures and the processing reports generated by the software. [file mmc3.zip › BA18008_SCC-Optimized_r3.pdf]

BA18-008_SCC_Optimized_r3

Automatically cleaned sparse cloud using the SCC script (optimized settings). The specimen was photographed in the GeoMuseum-PL photogrammetry laboratory (turntable/lightbox setup) of the Institute of Geology and Mineralogy, University of Cologne.

29 December 2023

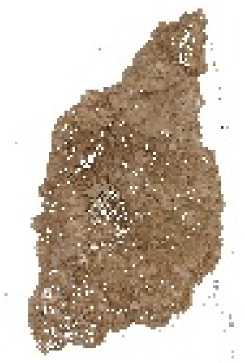

Survey Data

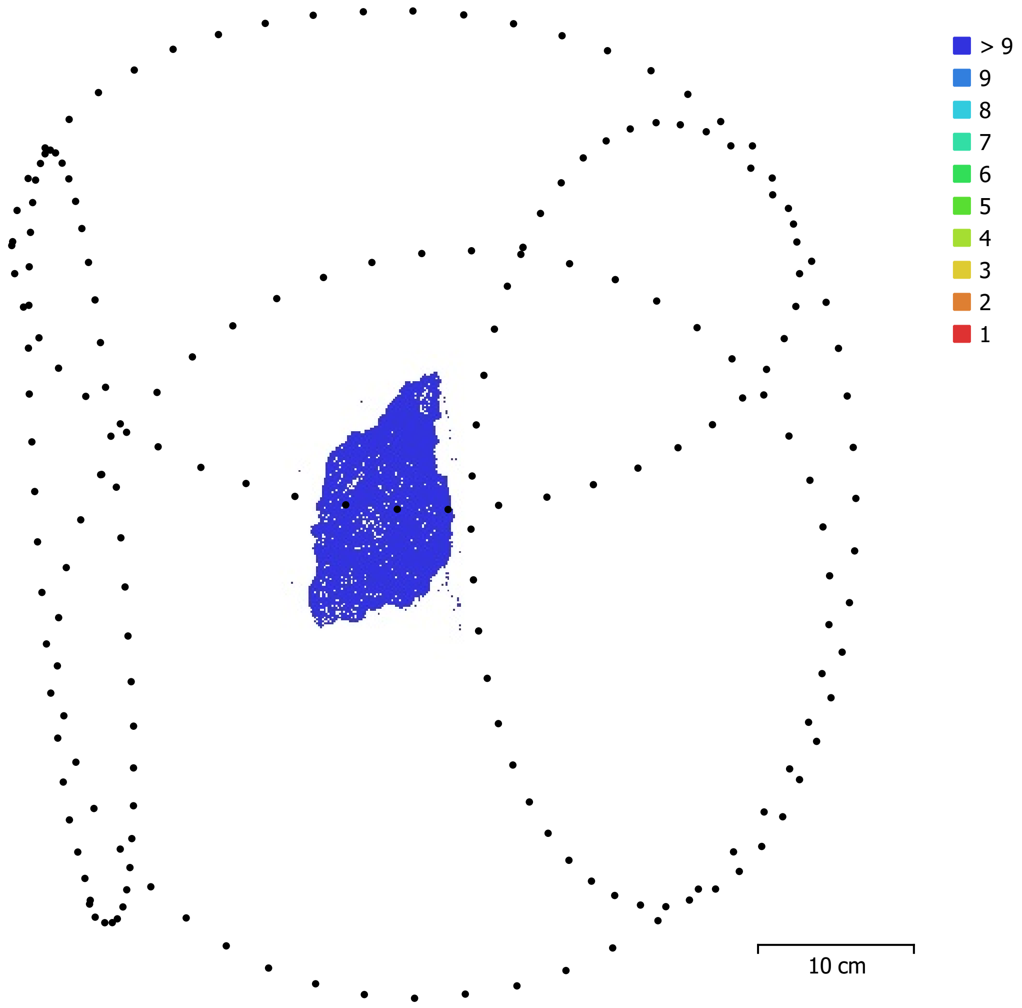

Fig. 1. Camera locations and image overlap.

Number of images:	196	Camera stations:	196
Flying altitude:	28.2 cm	Tie points:	45,475
Ground resolution:	0.0287 mm/pix	Projections:	212,157
Coverage area:	98.4 cm²	Reprojection error:	0.313 pix

Camera Model	Resolution	Focal Length	Pixel Size	Precalibrated
NEX-7, E 35mm F1.8 OS...	6000 x 4000	35 mm	4.04 x 4.04 μm	No

Table 1. Cameras.

Camera Calibration

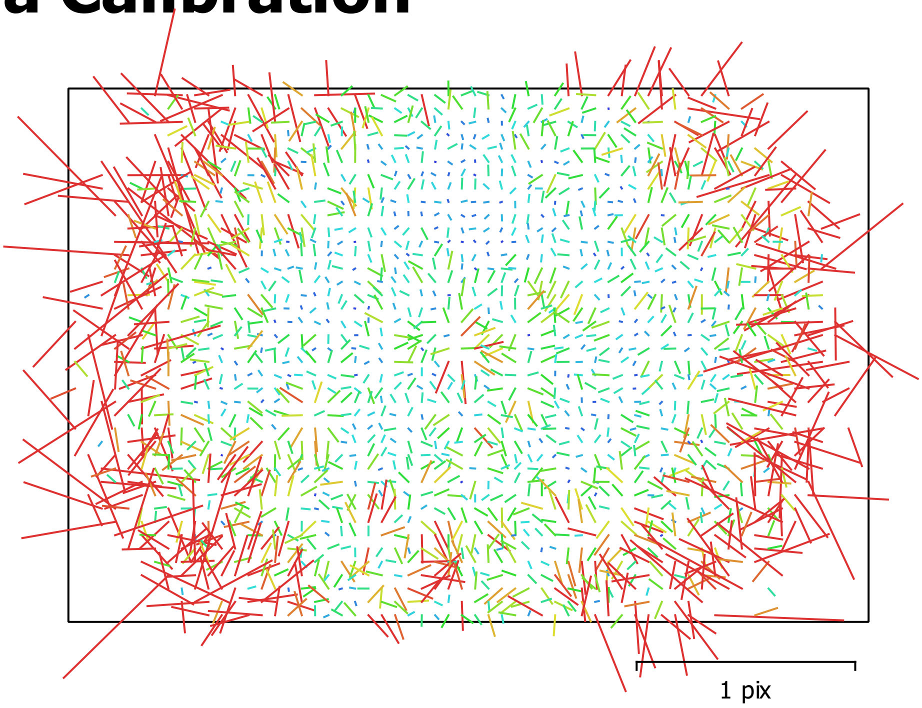

Fig. 2. Image residuals for NEX-7, E 35mm F1.8 OSS (35mm).

NEX-7, E 35mm F1.8 OSS (35mm)

196 images

Type	Resolution	Focal Length	Pixel Size
**Frame**	**6000 x 4000**	**35 mm**	**4.04 x 4.04 µm**

	Value	Error	F	Cx	Cy	K1	K2	K3	P1	P2
**F**	**9817.83**	0.47	1.00	0.09	-0.15	-0.22	0.07	0.01	0.06	-0.13
**Cx**	**-23.9581**	0.54		1.00	0.04	-0.05	0.03	-0.02	0.96	0.04
**Cy**	**-20.1988**	0.48			1.00	-0.08	0.05	-0.03	0.06	0.91
**K1**	**0.0537313**	0.00078				1.00	-0.91	0.80	-0.04	-0.02
**K2**	**-0.724404**	0.022					1.00	-0.97	0.02	0.04
**K3**	**3.69097**	0.19						1.00	-0.01	-0.03
**P1**	**0.00119756**	2.1e-05							1.00	0.04
**P2**	**0.000353459**	1.9e-05								1.00

Table 2. Calibration coefficients and correlation matrix.

Scale Bars

Label	Distance (m)	Error (m)
target 156_target 157	0.0500041	4.10046e-06
target 162_target 163	0.0499959	-4.10099e-06
**Total**		**4.10072e-06**

Table 3. Control scale bars.

Label	Distance (m)	Error (m)
target 154_target 155	0.049894	-0.000106041
target 160_target 161	0.0500347	3.46899e-05
**Total**		**7.88926e-05**

Table 4. Check scale bars.

Digital Elevation Model

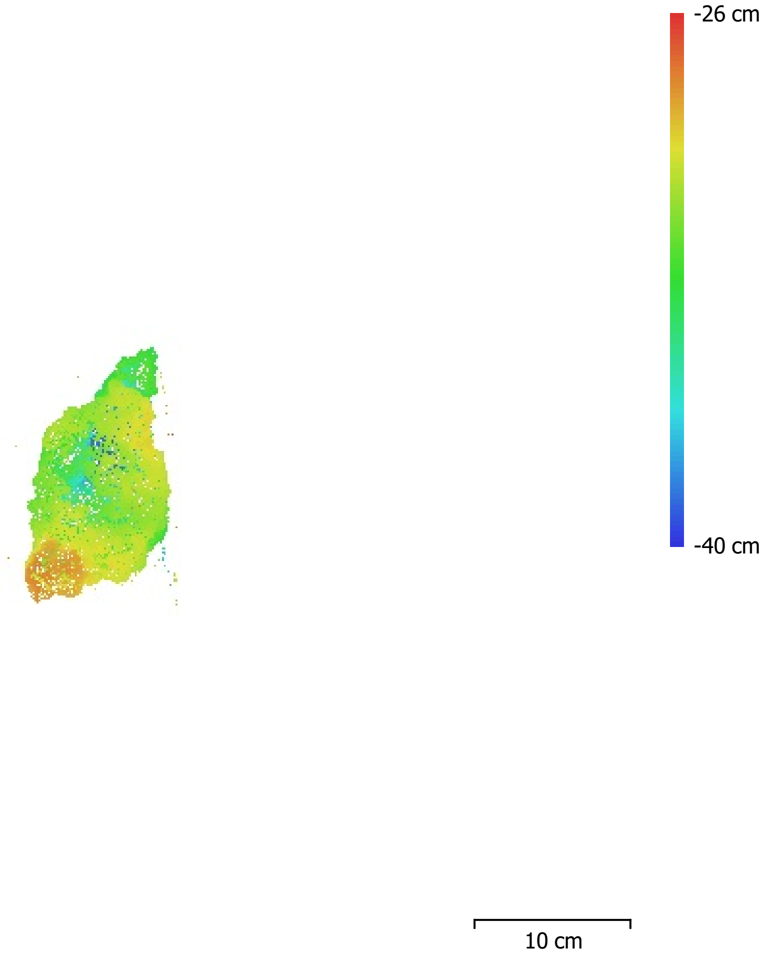

Fig. 3. Reconstructed digital elevation model.

Resolution:	unknown
Point density:	unknown

Processing Parameters

**General**	
Cameras	196
Aligned cameras	196
Markers	8
Scale bars	4
Coordinate system	Local Coordinates (m)
Rotation angles	Yaw, Pitch, Roll
**Tie Points**	
Points	45,475 of 1,100,512
RMS reprojection error	0.148688 (0.313191 pix)
Max reprojection error	0.304687 (0.865908 pix)
Mean key point size	2.10086 pix
Point colors	3 bands, uint8
Key points	No
Average tie point multiplicity	3.82927
**Alignment parameters**	
Accuracy	High
Generic preselection	Yes
Reference preselection	No
Key point limit	60,000
Key point limit per Mpx	1,000
Tie point limit	0
Filter points by mask	Yes
Mask tie points	No
Exclude stationary tie points	Yes
Guided image matching	No
Adaptive camera model fitting	No
Matching time	11 minutes 38 seconds
Matching memory usage	1.15 GB
Alignment time	5 minutes 29 seconds
Alignment memory usage	376.73 MB
**Optimization parameters**	
Parameters	f, cx, cy, k1-k3, p1, p2
Adaptive camera model fitting	No
Optimization time	2 seconds
Date created	2023:09:28 12:54:01
Software version	2.0.0.15597
File size	68.52 MB
**System**	
Software name	Agisoft Metashape Professional
Software version	2.0.3 build 16960
OS	Windows 64 bit
RAM	63.90 GB
CPU	Intel(R) Core(TM) i7-7700 CPU @ 3.60GHz
GPU(s)	Quadro M4000
