# Supplementary material for: Automated cleaning of tie point clouds following USGS guidelines in Agisoft Metashape professional (ver. 2.1.0)
Source: MethodsX. 2024 Mar 26;12:102679. doi: 10.1016/j.mex.2024.102679 (PMC10992719; doi:10.1016/j.mex.2024.102679)
Supplement: Supplementary file 3 — The supplementary material includes supplementary text, figures and the processing reports generated by the software. [file mmc3.zip › Lucia_Manual-Default.pdf]

Lucia_Manual-Default

Manually cleaned sparse cloud, following the suggestions of Over et al. (2021). Default settings applied using the gradual selection tool. UAS data provided by Sanz-Ablanedo et al. (2018).

Sanz-Ablanedo, E., Chandler, J. H., Rodríguez-Pérez, J. R., and Ordóñez, C.: Accuracy of Unmanned Aerial Vehicle (UAV) and SfM Photogrammetry Survey as a Function of the Number and Location of Ground Control Points Used, Remote Sensing, 10, 1606, 2018.

29 December 2023

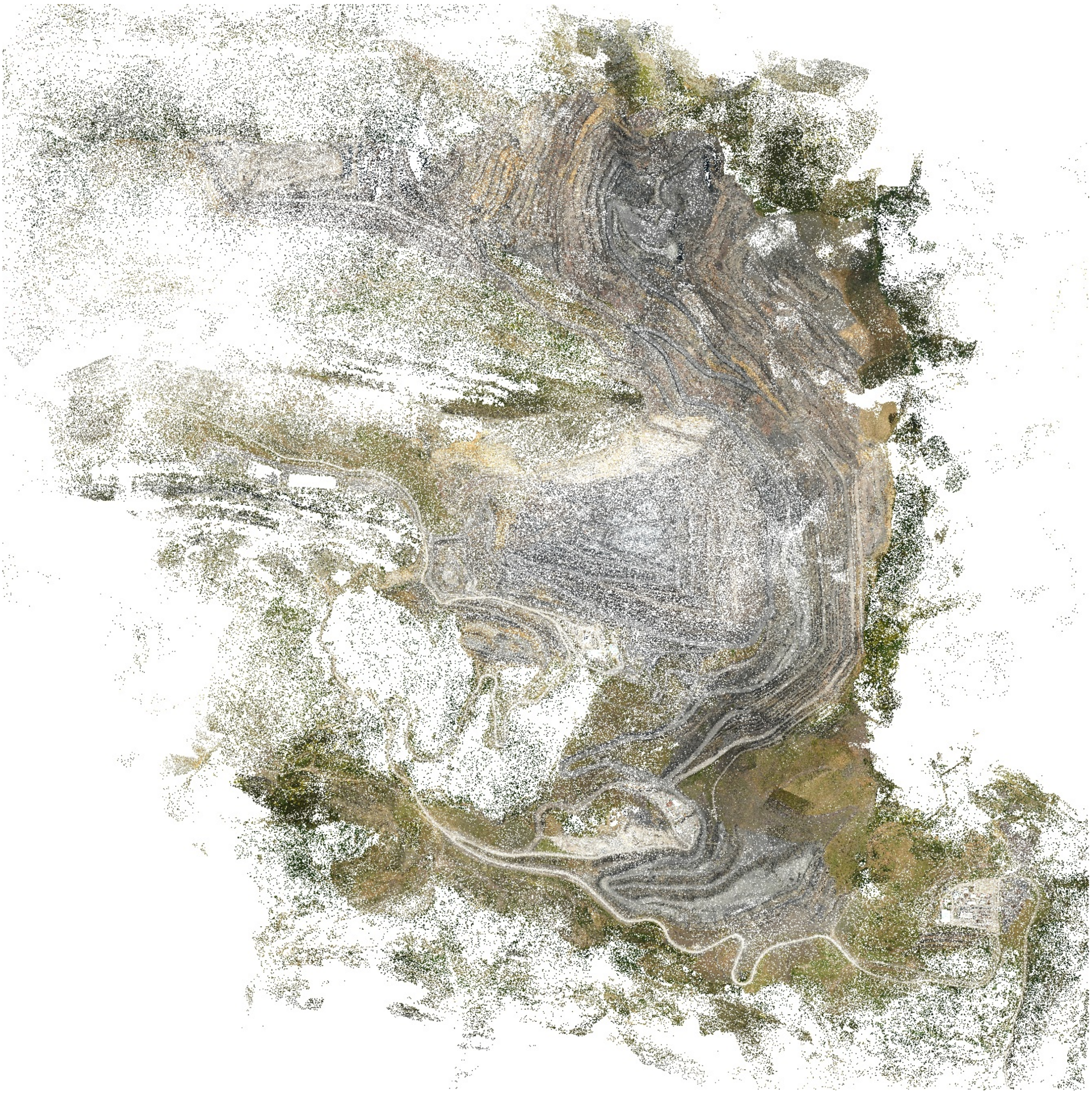

Survey Data

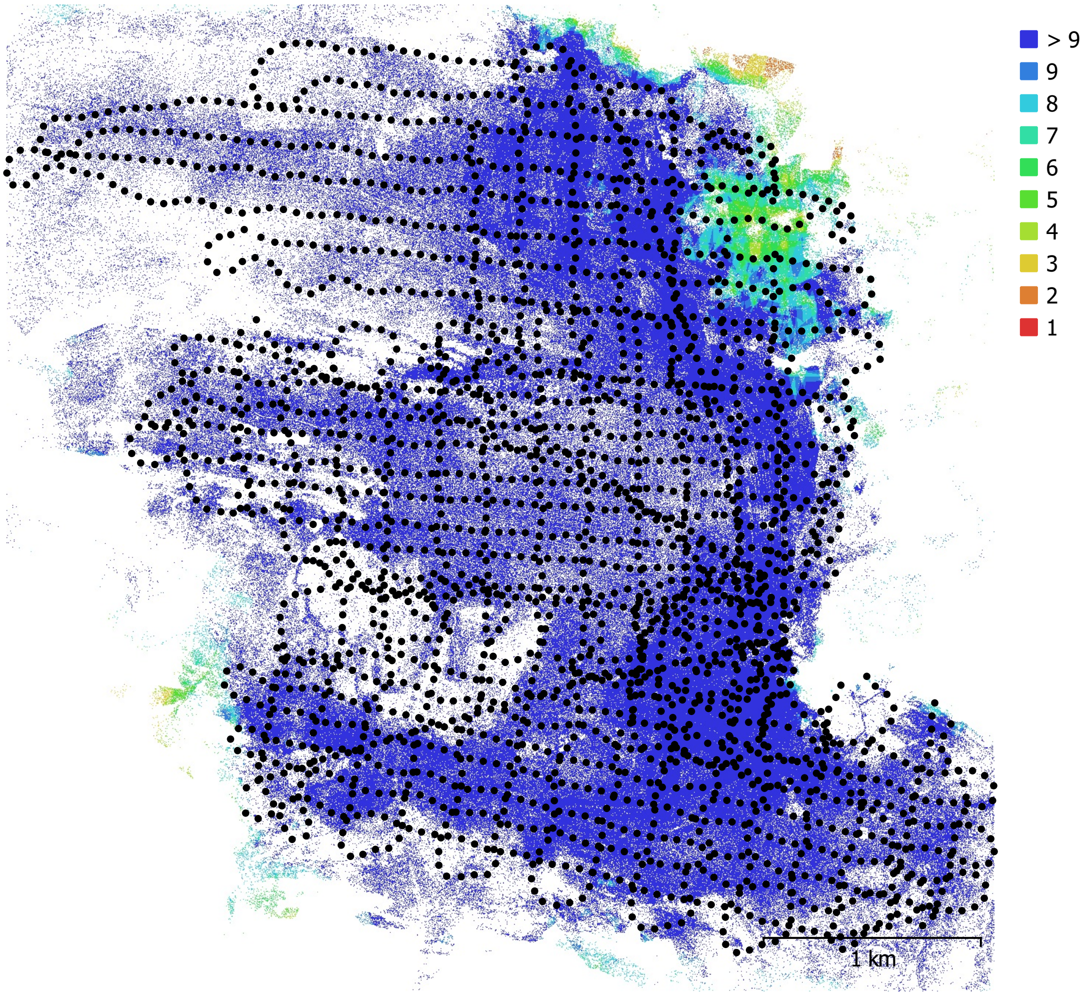

Fig. 1. Camera locations and image overlap.

Number of images:	2,595	Camera stations:	2,577
Flying altitude:	349 m	Tie points:	1,809,213
Ground resolution:	6.2 cm/pix	Projections:	4,283,125
Coverage area:	7.52 km²	Reprojection error:	0.33 pix

**Camera Model**	**Resolution**	**Focal Length**	**Pixel Size**	**Precalibrated**
NX500 (20 mm)	6480 x 4320	20 mm	3.7 x 3.7 μm	No
NX500 (20 mm)	6480 x 4320	20 mm	3.7 x 3.7 μm	No
NX500 (20 mm)	6480 x 4320	20 mm	3.7 x 3.7 μm	No
NX500 (20 mm)	6480 x 4320	20 mm	3.7 x 3.7 μm	No
NX500 (20 mm)	6480 x 4320	20 mm	3.7 x 3.7 μm	No

Camera Model	Resolution	Focal Length	Pixel Size	Precalibrated
NX500 (20 mm)	6480 x 4320	20 mm	3.7 x 3.7 µm	No

Table 1. Cameras.

Camera Calibration

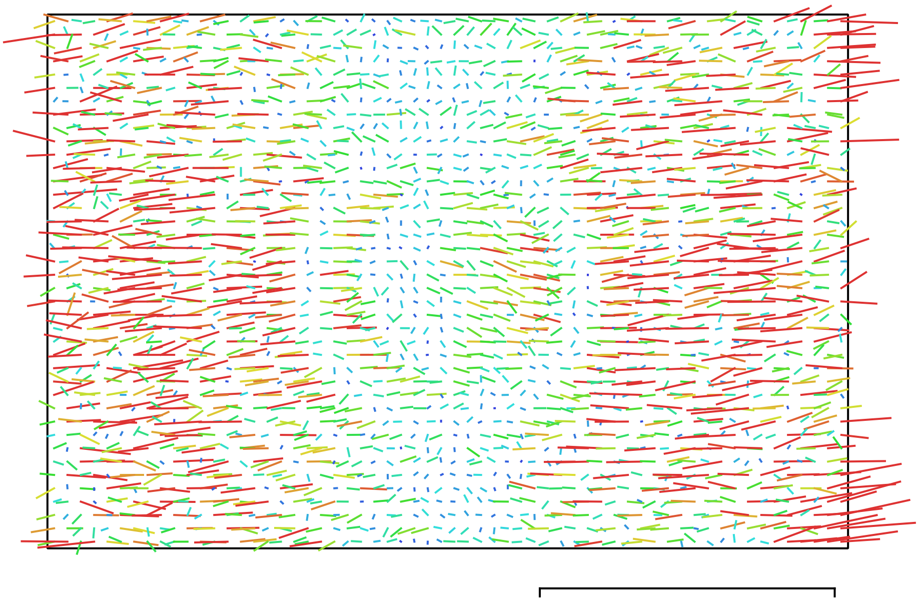

Fig. 2. Image residuals for NX500 (20 mm).

NX500 (20 mm)

200 images

Type	Resolution	Focal Length	Pixel Size
**Frame**	**6480 x 4320**	**20 mm**	**3.7 x 3.7 μm**

F:	5620.21		
Cx:	93.1786	B1:	0
Cy:	36.961	B2:	0
K1:	-0.0120386	P1:	0.00274749
K2:	0.0260299	P2:	0.000826694
K3:	-0.0223516	P3:	0
K4:	0	P4:	0

Camera Calibration

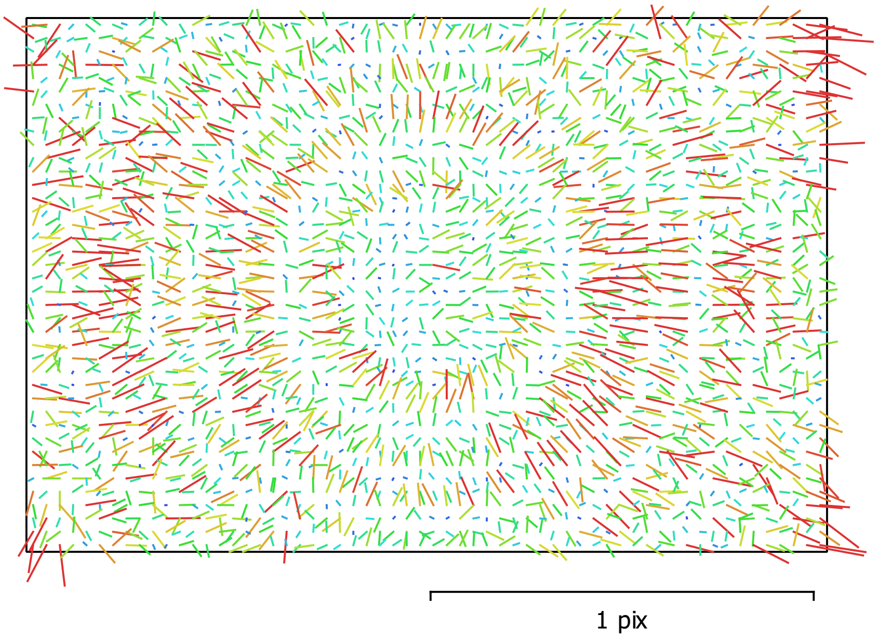

Fig. 3. Image residuals for NX500 (20 mm).

NX500 (20 mm)

462 images

Type	Resolution	Focal Length	Pixel Size
**Frame**	**6480 x 4320**	**20 mm**	**3.7 x 3.7 µm**

F:	5628.99		
Cx:	71.4744	B1:	0
Cy:	44.1822	B2:	0
K1:	-0.0117069	P1:	0.00226291
K2:	0.0266548	P2:	0.00118074
K3:	-0.0241965	P3:	0
K4:	0	P4:	0

Camera Calibration

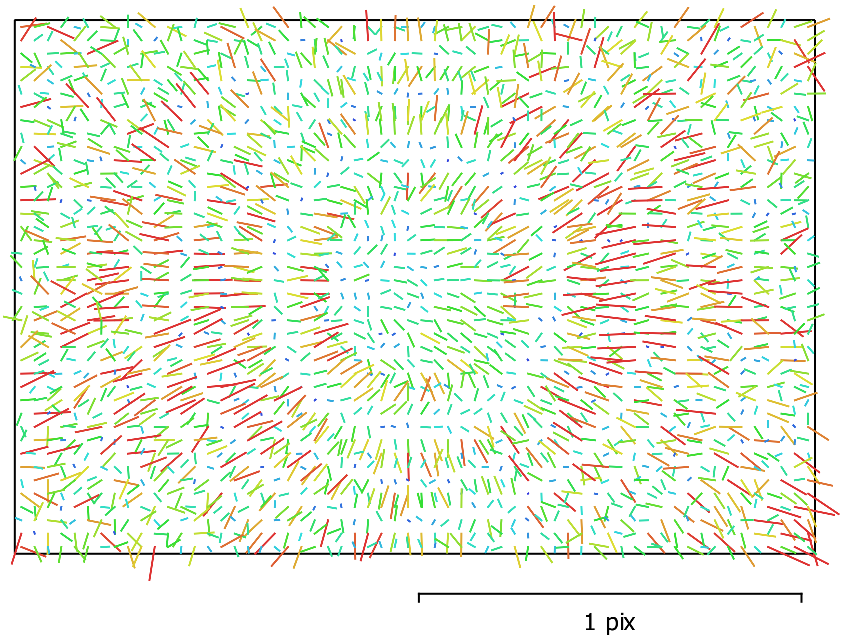

Fig. 4. Image residuals for NX500 (20 mm).

NX500 (20 mm)

530 images

Type	Resolution	Focal Length	Pixel Size
**Frame**	**6480 x 4320**	**20 mm**	**3.7 x 3.7 μm**

F:	5628.63		
Cx:	84.0755	B1:	0
Cy:	35.1679	B2:	0
K1:	-0.0120185	P1:	0.00253748
K2:	0.0305957	P2:	0.000926661
K3:	-0.0320526	P3:	0
K4:	0	P4:	0

Camera Calibration

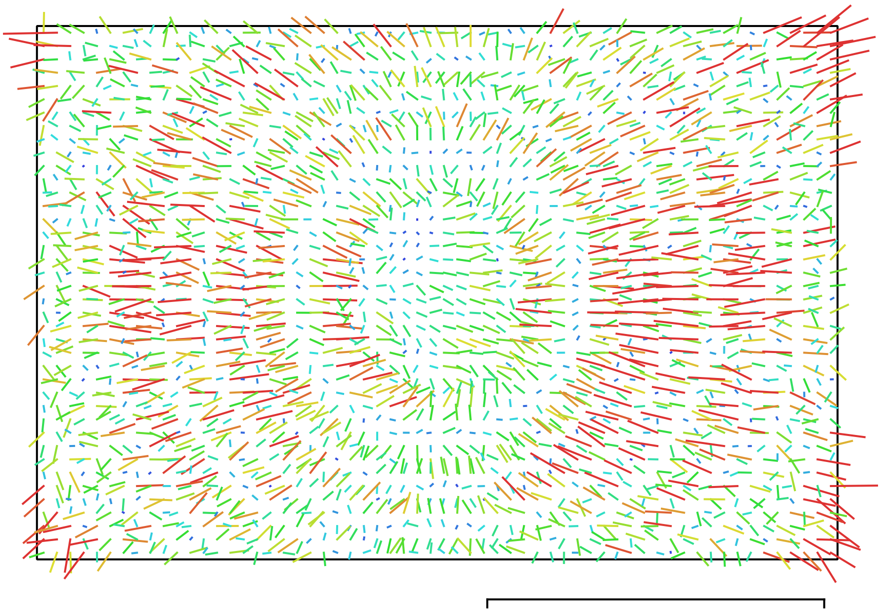

Fig. 5. Image residuals for NX500 (20 mm).

NX500 (20 mm)

513 images

Type	Resolution	Focal Length	Pixel Size
**Frame**	**6480 x 4320**	**20 mm**	**3.7 x 3.7 μm**

F:	5624.2		
Cx:	84.0068	B1:	0
Cy:	59.9958	B2:	0
K1:	-0.0106991	P1:	0.00251165
K2:	0.0220083	P2:	0.00151017
K3:	-0.0160379	P3:	0
K4:	0	P4:	0

Camera Calibration

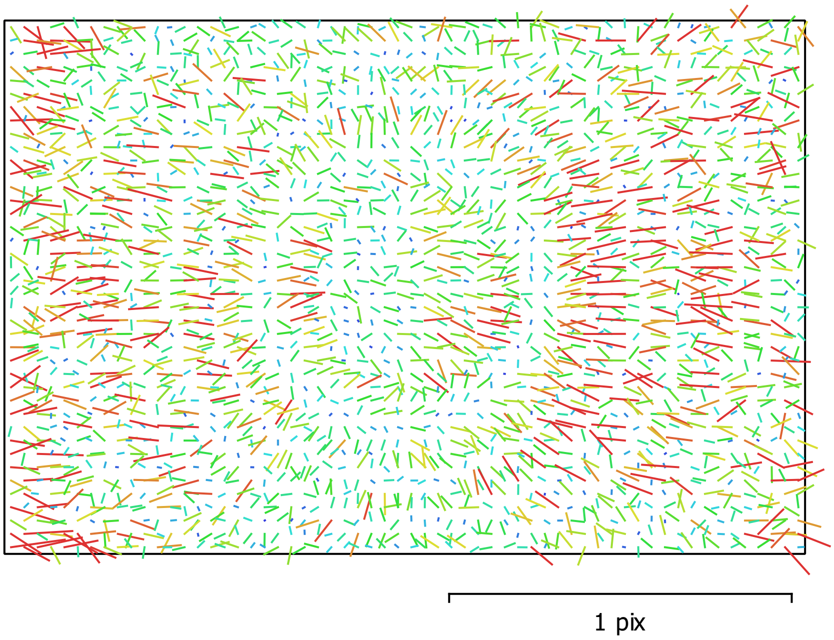

Fig. 6. Image residuals for NX500 (20 mm).

NX500 (20 mm)

412 images

Type	Resolution	Focal Length	Pixel Size
**Frame**	**6480 x 4320**	**20 mm**	**3.7 x 3.7 µm**

F:	5626.74		
Cx:	88.7521	B1:	0
Cy:	45.5222	B2:	0
K1:	-0.012702	P1:	0.00261097
K2:	0.031587	P2:	0.00113731
K3:	-0.0335598	P3:	0
K4:	0	P4:	0

Camera Calibration

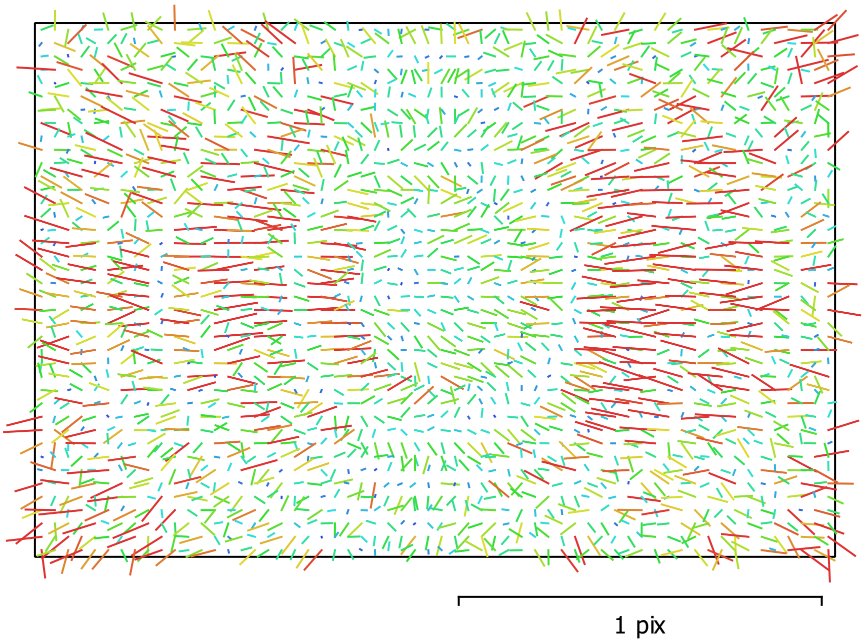

Fig. 7. Image residuals for NX500 (20 mm).

NX500 (20 mm)

478 images

Type	Resolution	Focal Length	Pixel Size
**Frame**	**6480 x 4320**	**20 mm**	**3.7 x 3.7 μm**

F:	5627.26		
Cx:	68.9622	B1:	0
Cy:	48.1364	B2:	0
K1:	-0.0125348	P1:	0.002021
K2:	0.0352762	P2:	0.00124887
K3:	-0.0391567	P3:	0
K4:	0	P4:	0

Ground Control Points

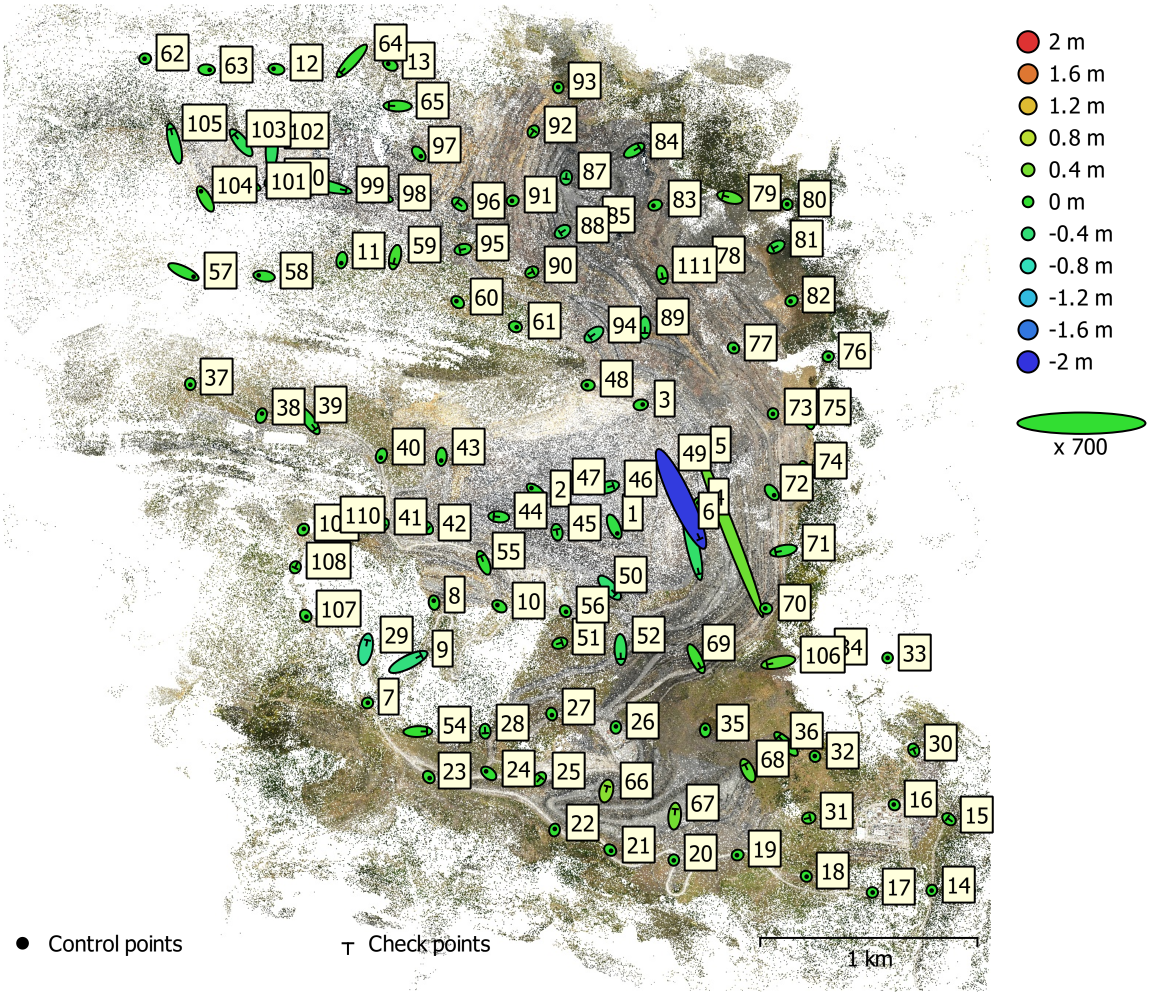

Fig. 8. GCP locations and error estimates.
Z error is represented by ellipse color. X,Y errors are represented by ellipse shape.
Estimated GCP locations are marked with a dot or crossing.

Count	X error (cm)	Y error (cm)	Z error (cm)	XY error (cm)	Total (cm)
55	2.79525	2.84686	2.69256	3.98974	4.8133

Table 2. Control points RMSE.
X - Easting, Y - Northing, Z - Altitude.

Count	X error (cm)	Y error (cm)	Z error (cm)	XY error (cm)	Total (cm)
54	10.3148	18.2747	32.4699	20.9848	38.6608

Table 3. Check points RMSE.
X - Easting, Y - Northing, Z - Altitude.

Label	X error (cm)	Y error (cm)	Z error (cm)	Total (cm)	Image (pix)
1	4.04994	-8.96932	-10.6602	14.5083	0.502 (104)
2	-5.92548	4.64388	-0.0981973	7.52905	0.483 (109)
3	2.53959	0.429761	-0.189097	2.58263	0.166 (51)
4	2.14634	8.9156	14.683	17.3115	0.625 (50)
7	0.456706	0.21664	0.00697465	0.505531	0.083 (24)
8	-0.365794	2.53752	-0.139868	2.56756	0.349 (32)
10	-2.66772	1.64566	2.26318	3.86612	0.498 (42)
11	-0.657849	-3.82945	-0.710228	3.94993	0.261 (36)
12	-3.68477	0.560151	0.375953	3.74602	0.406 (26)
13	-2.78498	1.40223	0.444118	3.14954	0.180 (20)
14	-0.176847	-0.373148	0.0572646	0.416885	0.073 (23)
16	-0.554616	0.449698	0.108846	0.722271	0.089 (34)
17	0.437657	-0.0160262	-0.0487402	0.440655	0.071 (23)
18	0.459565	-0.455832	-0.0461635	0.648933	0.113 (25)
19	-0.88845	-0.220882	-0.0915914	0.920066	0.104 (20)
20	-0.0280974	-0.479991	-0.0307439	0.481795	0.124 (16)
21	1.16365	-0.954966	0.177403	1.51576	0.136 (15)
22	0.33794	1.42732	-0.0161672	1.46687	0.120 (13)
23	1.1241	-1.3571	0.128564	1.76687	0.133 (18)
24	-3.48311	2.65624	-0.530569	4.41239	0.330 (27)
26	0.0466577	1.27845	-0.556286	1.39501	0.126 (33)
27	0.402789	-1.35967	0.0579017	1.41926	0.192 (27)
32	-0.228516	0.381687	-0.0166412	0.445176	0.068 (18)
33	0.012718	0.00620355	-0.00051338	0.0141596	0.001 (3)
34	-0.0141669	0.0687252	-0.0192008	0.0727497	0.014 (4)
35	0.0738018	2.05801	-0.443059	2.10645	0.258 (11)
37	0.0252773	-0.921276	0.269766	0.960293	0.120 (46)
38	0.889666	2.71462	-0.553796	2.90987	0.180 (57)
40	-0.864909	-2.61335	-1.30779	3.04762	0.272 (66)
43	-0.249895	-4.96421	0.245568	4.97656	0.489 (69)
48	-1.78194	0.0686227	-0.520899	1.85778	0.186 (44)

Label	X error (cm)	Y error (cm)	Z error (cm)	Total (cm)	Image (pix)
56	0.728549	-0.931884	-0.0814574	1.18568	0.308 (50)
57	13.3635	-6.39242	-1.56004	14.8957	1.481 (14)
58	-7.09607	1.03329	-0.901453	7.22734	0.856 (30)
60	-1.67056	1.37394	0.137255	2.16732	0.158 (32)
61	1.60217	-0.571265	0.563321	1.79182	0.131 (20)
62	-0.813034	-0.111404	0.205042	0.845859	0.146 (17)
63	3.95468	-0.220702	-0.284282	3.97102	0.309 (16)
70	-1.06679	0.191513	-0.786181	1.33895	0.099 (30)
72	3.09554	-3.73221	-3.19651	5.8077	0.264 (18)
73	-0.0490188	-0.251445	0.166252	0.305396	0.064 (15)
76	0.588271	0.137928	-0.0157637	0.604429	0.094 (9)
77	0.71676	-0.51775	0.027345	0.884623	0.088 (11)
78	0.380504	-1.58336	-0.237478	1.64566	0.121 (11)
80	-0.0143309	0.329591	0.0276702	0.331061	0.092 (7)
82	-1.24435	-0.658492	-0.275934	1.43463	0.212 (6)
83	-2.18227	-0.647894	-0.169805	2.28274	0.203 (13)
85	0.377635	0.0558817	-0.353052	0.519977	0.205 (10)
91	-0.979748	-0.251866	-0.165125	1.02499	0.188 (20)
93	-0.0434712	0.613778	-0.0918198	0.622128	0.169 (16)
97	2.7008	-3.19501	0.245429	4.19078	0.362 (40)
100	1.98122	0.566288	-3.24447	3.8435	0.821 (28)
104	-5.80269	10.0815	5.46163	12.8505	1.071 (21)
107	0.852937	-1.17724	-0.468133	1.52727	0.102 (21)
109	0.810559	0.913187	2.15844	2.47988	0.263 (22)
**Total**	**2.79525**	**2.84686**	**2.69256**	**4.8133**	**0.394**

Table 4. Control points.

X - Easting, Y - Northing, Z - Altitude.

Label	X error (cm)	Y error (cm)	Z error (cm)	Total (cm)	Image (pix)
5	-40.9808	105.633	34.1331	118.333	0.545 (38)
6	8.4594	-37.8032	-31.693	50.0509	0.259 (66)
9	17.2869	8.60566	-43.2694	47.3829	0.244 (29)

Label	X error (cm)	Y error (cm)	Z error (cm)	Total (cm)	Image (pix)
15	-2.03025	1.4773	2.08089	3.26105	0.117 (27)
25	2.10418	2.40712	7.15063	7.83284	0.102 (22)
28	0.12236	-2.56933	-7.48508	7.91472	0.163 (29)
29	2.35546	11.9165	-54.0267	55.3754	0.029 (18)
30	-0.744043	1.68447	0.0354319	1.84182	0.103 (20)
31	1.60329	0.378066	8.17872	8.34295	0.134 (26)
36	-9.64985	9.77214	3.89661	14.2758	0.135 (14)
39	8.56166	-11.7007	-6.33029	15.8203	0.206 (40)
41	1.31017	1.90432	-10.1571	10.4168	0.395 (56)
42	2.60799	-2.35422	-11.2167	11.754	0.371 (48)
44	-6.58826	0.635057	-5.53757	8.62978	0.603 (73)
45	-0.651555	3.63152	-8.97272	9.70166	0.556 (94)
46	6.49713	1.96392	-17.5606	18.8267	0.408 (63)
47	2.86785	5.79216	-15.25	16.5631	0.478 (107)
49	24.8395	-52.8372	-194.703	203.268	0.769 (59)
50	8.16355	-8.78581	-36.7235	38.6322	0.426 (56)
51	3.00691	0.968463	-2.30252	3.9091	0.351 (42)
52	0.360123	-12.4656	-24.7979	27.7571	0.308 (56)
54	11.9821	0.351153	1.50796	12.0817	0.119 (31)
55	-3.78237	8.93546	-1.05551	9.76027	0.341 (51)
59	-2.12255	-8.86966	9.81969	13.4016	0.254 (9)
64	-14.3757	-15.6267	4.21013	21.6467	0.289 (13)
65	-11.4419	0.300888	3.04238	11.8433	0.348 (31)
66	1.49285	6.25513	50.4769	50.8849	0.180 (22)
67	0.748604	9.38918	36.9175	38.1001	0.162 (12)
68	-3.91064	7.96661	17.3688	19.5047	0.137 (16)
69	6.24731	-12.2901	7.40418	15.6492	0.215 (28)
71	-10.5057	-2.15159	-4.24771	11.5344	0.222 (24)
74	3.11464	-6.3302	6.24271	9.42039	0.105 (10)
75	-1.68658	7.11364	1.44937	7.45313	0.024 (6)
79	-9.22957	2.44334	18.2917	20.6335	0.142 (8)
81	-4.39106	-2.25181	-9.80062	10.9729	0.238 (6)

Label	X error (cm)	Y error (cm)	Z error (cm)	Total (cm)	Image (pix)
84	6.8282	4.01507	-2.97651	8.46196	0.174 (12)
87	-0.177154	-1.78948	-19.4292	19.5122	0.240 (10)
88	-2.94208	-1.88921	-22.0107	22.2867	0.241 (13)
89	-0.096775	-7.04142	-17.1817	18.5689	0.161 (27)
90	1.70221	0.852998	3.80144	4.25159	0.153 (28)
92	0.839743	1.18259	2.44282	2.84096	0.223 (18)
94	-5.1454	-3.65078	-28.1643	28.8623	0.148 (35)
95	-4.07767	-0.834441	-2.4063	4.80769	0.248 (38)
96	-3.28546	2.59166	-4.67346	6.27313	0.193 (23)
98	16.6515	-3.77259	-1.19238	17.1151	0.573 (32)
99	30.4289	-6.97942	-14.0057	34.2168	0.572 (30)
101	-16.849	7.81449	-2.49681	18.7401	0.670 (26)
102	0.829733	17.0442	-26.3447	31.3885	0.364 (21)
103	-8.92904	11.1239	-20.5835	25.0429	0.478 (20)
105	-4.84985	19.0915	-17.1355	26.1081	0.199 (24)
106	-14.8764	-2.91684	20.0211	25.1129	0.087 (13)
108	-0.428106	-0.907386	3.82903	3.95829	0.179 (19)
110	-2.25429	3.24822	-7.04633	8.07982	0.262 (28)
111	1.17994	-5.1267	2.68092	5.90446	0.141 (14)
**Total**	**10.3148**	**18.2747**	**32.4699**	**38.6608**	**0.386**

Table 5. Check points.

X - Easting, Y - Northing, Z - Altitude.

Digital Elevation Model

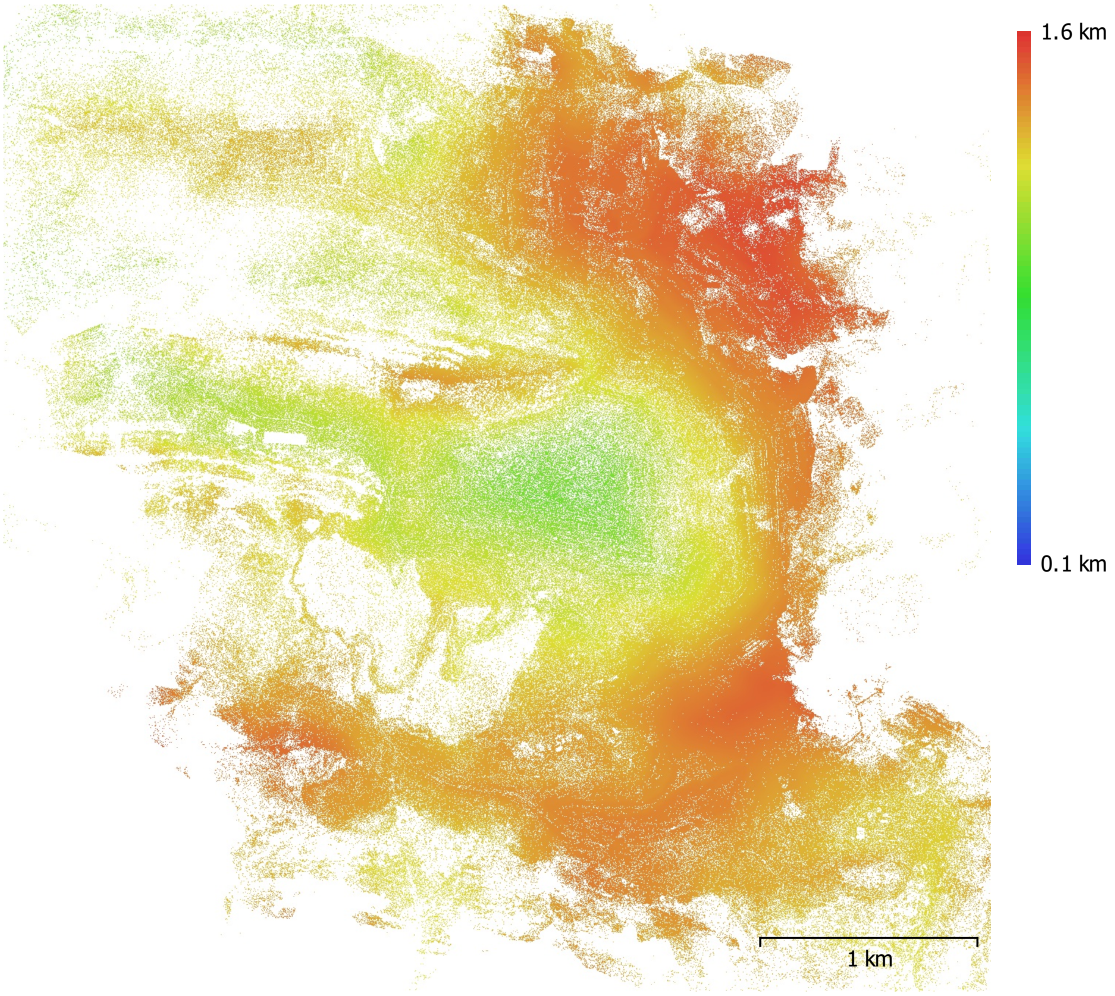

Fig. 9. Reconstructed digital elevation model.

Resolution:	unknown
Point density:	unknown

Processing Parameters

**General**	
Cameras	2595
Aligned cameras	2577
Markers	110
**Shapes**	
Polygon	1
Coordinate system	ETRS89 / UTM zone 30N (EPSG::25830)
Rotation angles	Yaw, Pitch, Roll
**Tie Points**	
Points	1,809,213 of 12,529,745
RMS reprojection error	0.138903 (0.330138 pix)
Max reprojection error	0.299998 (1.79095 pix)
Mean key point size	2.33432 pix
Point colors	3 bands, uint8
Key points	No
Average tie point multiplicity	3.65511
**Alignment parameters**	
Accuracy	High
Generic preselection	Yes
Reference preselection	No
Key point limit	60,000
Key point limit per Mpx	1,000
Tie point limit	0
Exclude stationary tie points	Yes
Guided image matching	No
Adaptive camera model fitting	No
Matching time	4 hours 7 minutes
Matching memory usage	3.73 GB
Alignment time	2 hours 17 minutes
Alignment memory usage	4.82 GB
**Optimization parameters**	
Parameters	f, cx, cy, k1-k3, p1, p2
Adaptive camera model fitting	No
Optimization time	28 seconds
Date created	2023:11:13 15:04:46
Software version	2.0.0.15597
File size	776.41 MB
**System**	
Software name	Agisoft Metashape Professional
Software version	2.0.3 build 16960
OS	Windows 64 bit
RAM	63.90 GB
CPU	Intel(R) Core(TM) i7-7700 CPU @ 3.60GHz
GPU(s)	Quadro M4000
