# Supplementary material for: Automated cleaning of tie point clouds following USGS guidelines in Agisoft Metashape professional (ver. 2.1.0)
Source: MethodsX. 2024 Mar 26;12:102679. doi: 10.1016/j.mex.2024.102679 (PMC10992719; doi:10.1016/j.mex.2024.102679)
Supplement: Supplementary file 3 — The supplementary material includes supplementary text, figures and the processing reports generated by the software. [file mmc3.zip › Lucia_SCC-Default_r1.pdf]

Lucia_SCC-Default_r1

Automatically cleaned sparse cloud using the SCC script (default settings). UAS data provided by Sanz-Ablanedo et al. (2018).

Sanz-Ablanedo, E., Chandler, J. H., Rodríguez-Pérez, J. R., and Ordóñez, C.: Accuracy of Unmanned Aerial Vehicle (UAV) and SfM Photogrammetry Survey as a Function of the Number and Location of Ground Control Points Used, Remote Sensing, 10, 1606, 2018.

28 December 2023

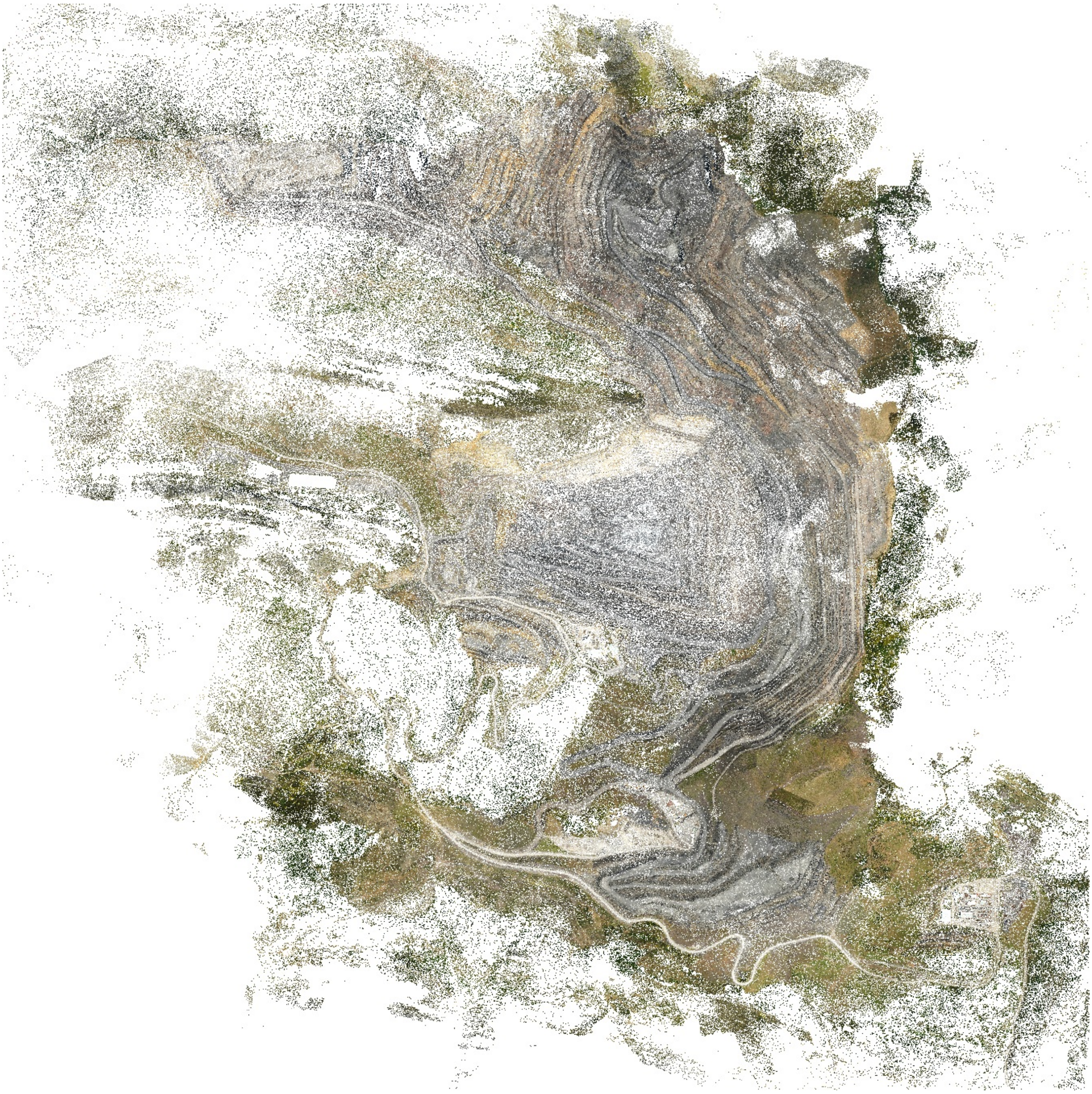

Survey Data

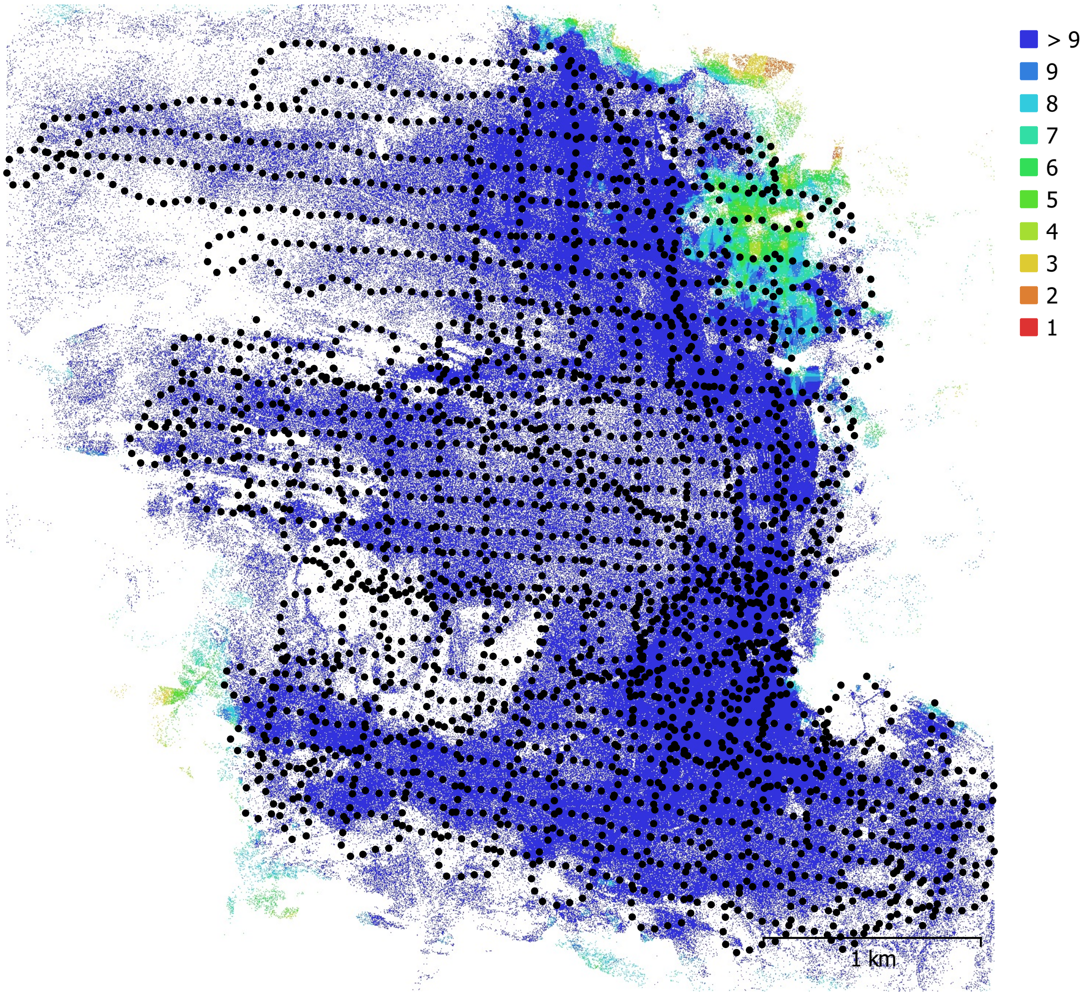

Fig. 1. Camera locations and image overlap.

Number of images:	2,595	Camera stations:	2,577
Flying altitude:	349 m	Tie points:	1,818,359
Ground resolution:	6.2 cm/pix	Projections:	4,310,173
Coverage area:	7.52 km²	Reprojection error:	0.331 pix

Camera Model	Resolution	Focal Length	Pixel Size	Precalibrated
NX500 (20 mm)	6480 x 4320	20 mm	3.7 x 3.7 μm	No
NX500 (20 mm)	6480 x 4320	20 mm	3.7 x 3.7 μm	No
NX500 (20 mm)	6480 x 4320	20 mm	3.7 x 3.7 μm	No
NX500 (20 mm)	6480 x 4320	20 mm	3.7 x 3.7 μm	No
NX500 (20 mm)	6480 x 4320	20 mm	3.7 x 3.7 μm	No

Camera Model	Resolution	Focal Length	Pixel Size	Precalibrated
NX500 (20 mm)	6480 x 4320	20 mm	3.7 x 3.7 µm	No

Table 1. Cameras.

Camera Calibration

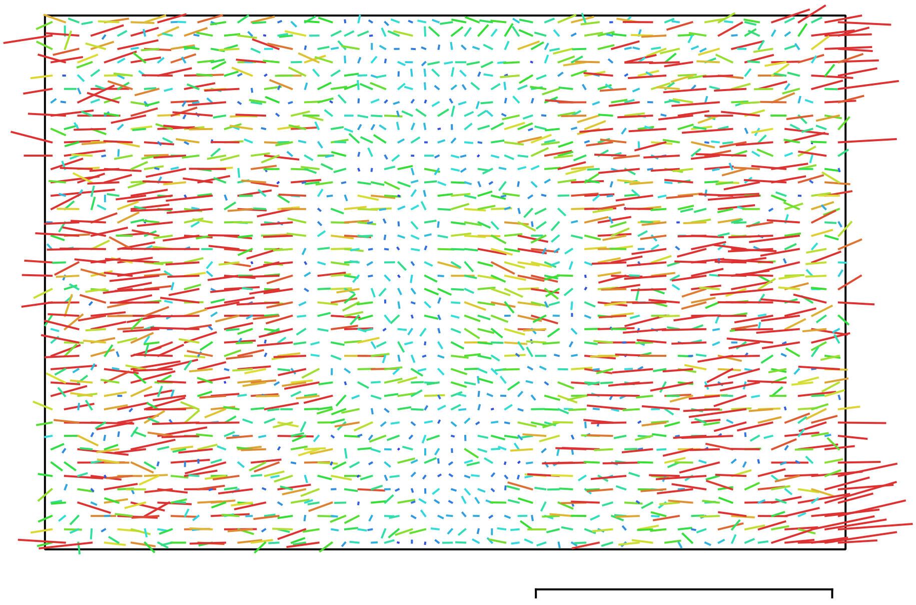

Fig. 2. Image residuals for NX500 (20 mm).

NX500 (20 mm)

200 images

Type	Resolution	Focal Length	Pixel Size
**Frame**	**6480 x 4320**	**20 mm**	**3.7 x 3.7 μm**

	Value	Error	F	Cx	Cy	K1	K2	K3	P1	P2
**F**	**5620.52**	0.05	1.00	0.02	0.01	-0.39	0.33	-0.30	-0.00	0.07
**Cx**	**93.3594**	0.059		1.00	-0.04	0.03	-0.01	0.01	0.82	0.06
**Cy**	**36.8005**	0.068			1.00	-0.00	0.00	-0.01	-0.02	0.78
**K1**	**-0.0120382**	6.3e-05				1.00	-0.96	0.91	0.05	0.00
**K2**	**0.0263152**	0.00031					1.00	-0.98	-0.04	-0.01
**K3**	**-0.0229198**	0.00046						1.00	0.05	0.01
**P1**	**0.00274893**	3.6e-06							1.00	0.04
**P2**	**0.000821608**	4.2e-06								1.00

Table 2. Calibration coefficients and correlation matrix.

Camera Calibration

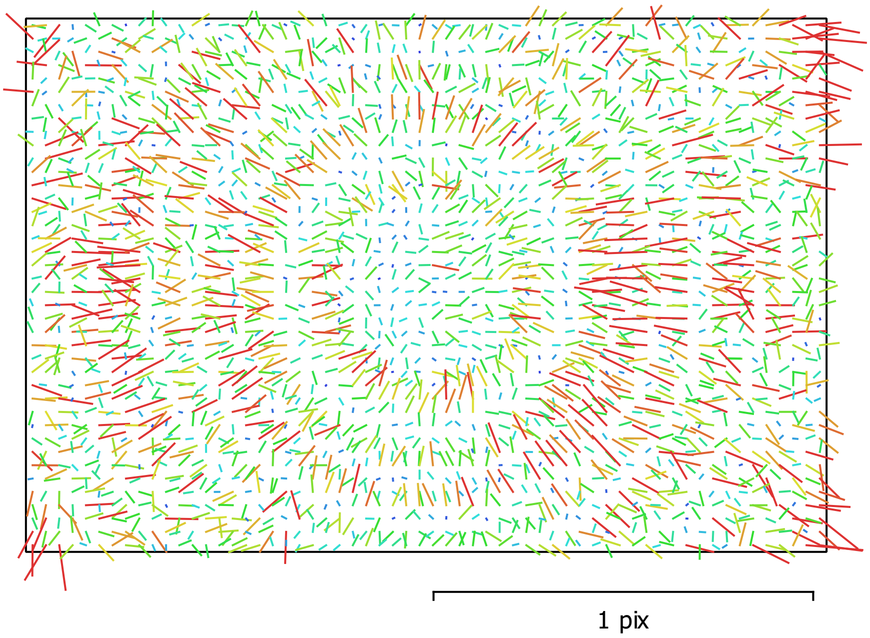

Fig. 3. Image residuals for NX500 (20 mm).

NX500 (20 mm)

462 images

Type	Resolution	Focal Length	Pixel Size
**Frame**	**6480 x 4320**	**20 mm**	**3.7 x 3.7 μm**

	Value	Error	F	Cx	Cy	K1	K2	K3	P1	P2
**F**	**5629.07**	0.041	1.00	-0.16	-0.12	-0.34	0.32	-0.28	-0.04	-0.02
**Cx**	**71.6231**	0.041		1.00	0.06	0.03	-0.03	0.03	0.88	0.02
**Cy**	**44.2577**	0.035			1.00	-0.00	-0.02	0.02	0.05	0.78
**K1**	**-0.0117591**	4.6e-05				1.00	-0.97	0.91	0.03	0.01
**K2**	**0.0270775**	0.00023					1.00	-0.98	-0.03	-0.03
**K3**	**-0.0249374**	0.00034						1.00	0.04	0.03
**P1**	**0.00227125**	2.5e-06							1.00	0.02
**P2**	**0.00118461**	2e-06								1.00

Table 3. Calibration coefficients and correlation matrix.

Camera Calibration

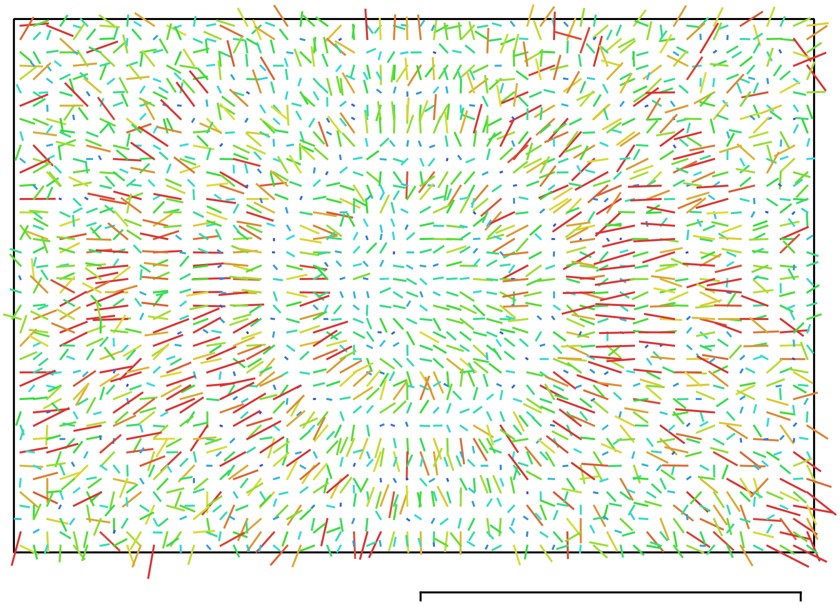

Fig. 4. Image residuals for NX500 (20 mm).

NX500 (20 mm)

530 images

Type	Resolution	Focal Length	Pixel Size
**Frame**	**6480 x 4320**	**20 mm**	**3.7 x 3.7 µm**

	Value	Error	F	Cx	Cy	K1	K2	K3	P1	P2
**F**	**5628.59**	0.044	1.00	-0.03	-0.13	-0.26	0.25	-0.22	-0.00	-0.02
**Cx**	**84.112**	0.038		1.00	-0.02	0.01	-0.01	0.01	0.83	0.00
**Cy**	**35.1988**	0.029			1.00	0.01	-0.02	0.01	-0.01	0.68
**K1**	**-0.0119976**	4e-05				1.00	-0.96	0.91	0.02	0.01
**K2**	**0.0304653**	0.00021					1.00	-0.98	-0.02	-0.02
**K3**	**-0.0318246**	0.00032						1.00	0.03	0.02
**P1**	**0.00253824**	2.2e-06							1.00	0.02
**P2**	**0.000928026**	1.6e-06								1.00

Table 4. Calibration coefficients and correlation matrix.

Camera Calibration

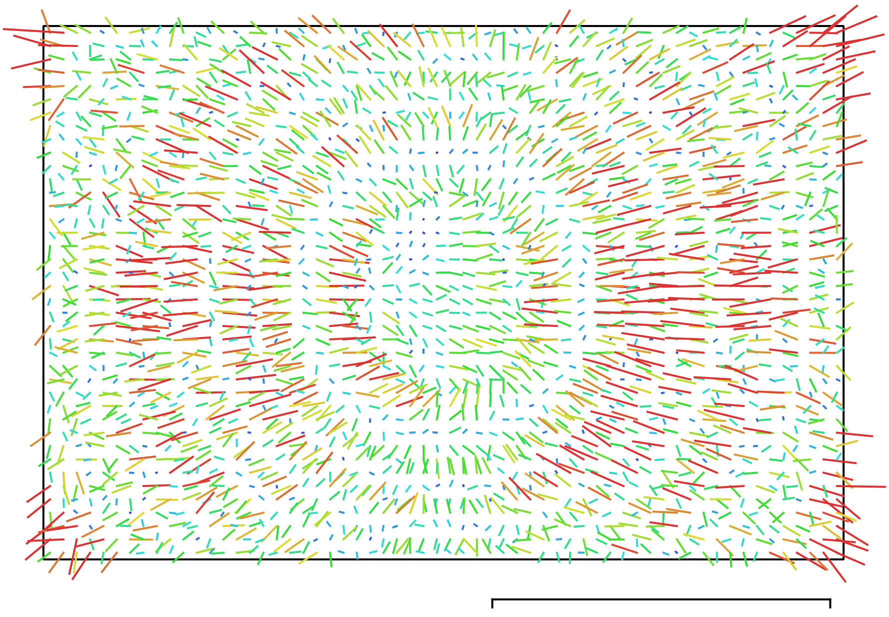

Fig. 5. Image residuals for NX500 (20 mm).

NX500 (20 mm)

513 images

Type	Resolution	Focal Length	Pixel Size
**Frame**	**6480 x 4320**	**20 mm**	**3.7 x 3.7 μm**

	Value	Error	F	Cx	Cy	K1	K2	K3	P1	P2
**F**	**5624.36**	0.052	1.00	-0.08	-0.08	-0.20	0.20	-0.18	0.00	-0.02
**Cx**	**83.9902**	0.034		1.00	-0.01	0.01	-0.01	0.02	0.80	-0.01
**Cy**	**59.8876**	0.028			1.00	0.01	-0.02	0.02	-0.02	0.72
**K1**	**-0.0106129**	3.6e-05				1.00	-0.96	0.90	0.03	0.01
**K2**	**0.0214698**	0.00018					1.00	-0.98	-0.03	-0.01
**K3**	**-0.0150281**	0.00028						1.00	0.04	0.01
**P1**	**0.00251085**	2.1e-06							1.00	-0.02
**P2**	**0.00151164**	1.7e-06								1.00

Table 5. Calibration coefficients and correlation matrix.

Camera Calibration

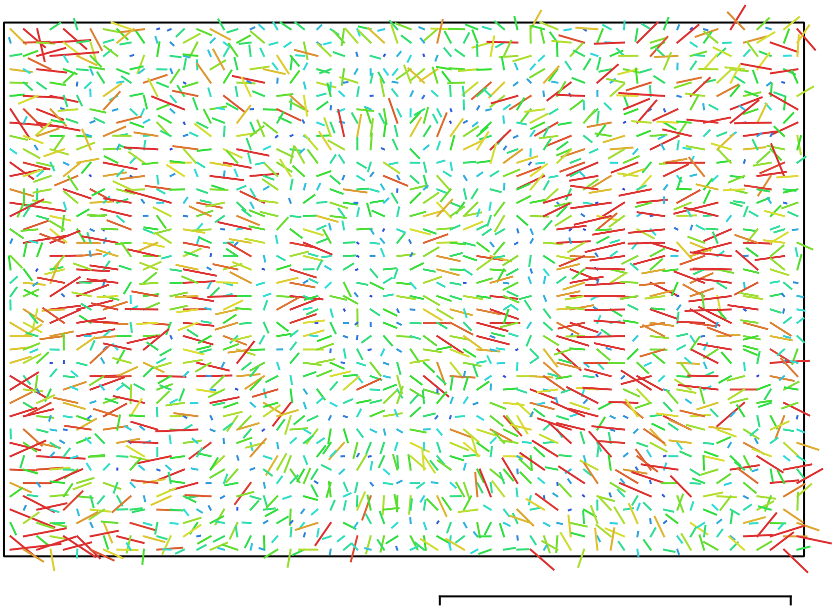

Fig. 6. Image residuals for NX500 (20 mm).

NX500 (20 mm)

412 images

Type	Resolution	Focal Length	Pixel Size
**Frame**	**6480 x 4320**	**20 mm**	**3.7 x 3.7 µm**

	Value	Error	F	Cx	Cy	K1	K2	K3	P1	P2
**F**	**5626.6**	0.045	1.00	0.05	-0.10	-0.36	0.34	-0.31	0.04	-0.01
**Cx**	**89.0109**	0.048		1.00	0.05	-0.01	0.00	0.00	0.87	0.04
**Cy**	**45.377**	0.038			1.00	-0.02	0.03	-0.04	0.05	0.74
**K1**	**-0.012629**	5.3e-05				1.00	-0.97	0.91	0.01	-0.01
**K2**	**0.0308073**	0.00027					1.00	-0.98	-0.01	0.00
**K3**	**-0.0320683**	0.0004						1.00	0.02	-0.00
**P1**	**0.00262101**	2.9e-06							1.00	0.05
**P2**	**0.00113058**	2.1e-06								1.00

Table 6. Calibration coefficients and correlation matrix.

Camera Calibration

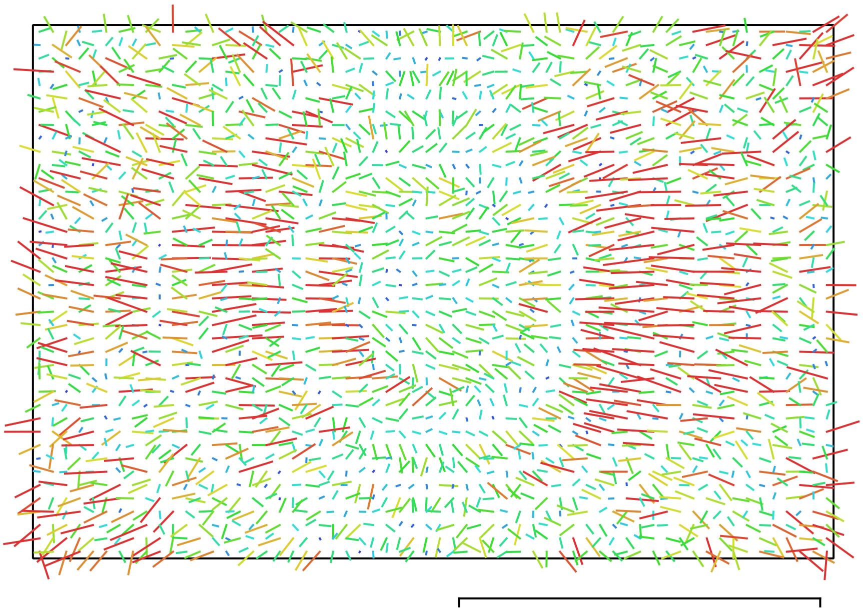

Fig. 7. Image residuals for NX500 (20 mm).

NX500 (20 mm)

478 images

Type	Resolution	Focal Length	Pixel Size
**Frame**	**6480 x 4320**	**20 mm**	**3.7 x 3.7 μm**

	Value	Error	F	Cx	Cy	K1	K2	K3	P1	P2
**F**	**5627.25**	0.032	1.00	-0.00	-0.00	-0.44	0.39	-0.34	-0.01	0.02
**Cx**	**68.8877**	0.04		1.00	-0.01	0.02	-0.01	0.01	0.87	-0.03
**Cy**	**48.2091**	0.036			1.00	0.02	-0.03	0.03	-0.02	0.74
**K1**	**-0.0125589**	4.7e-05				1.00	-0.97	0.91	0.01	0.01
**K2**	**0.0353533**	0.00024					1.00	-0.98	-0.01	-0.02
**K3**	**-0.0392415**	0.00037						1.00	0.01	0.02
**P1**	**0.00201834**	2.6e-06							1.00	-0.03
**P2**	**0.00125496**	2.1e-06								1.00

Table 7. Calibration coefficients and correlation matrix.

Ground Control Points

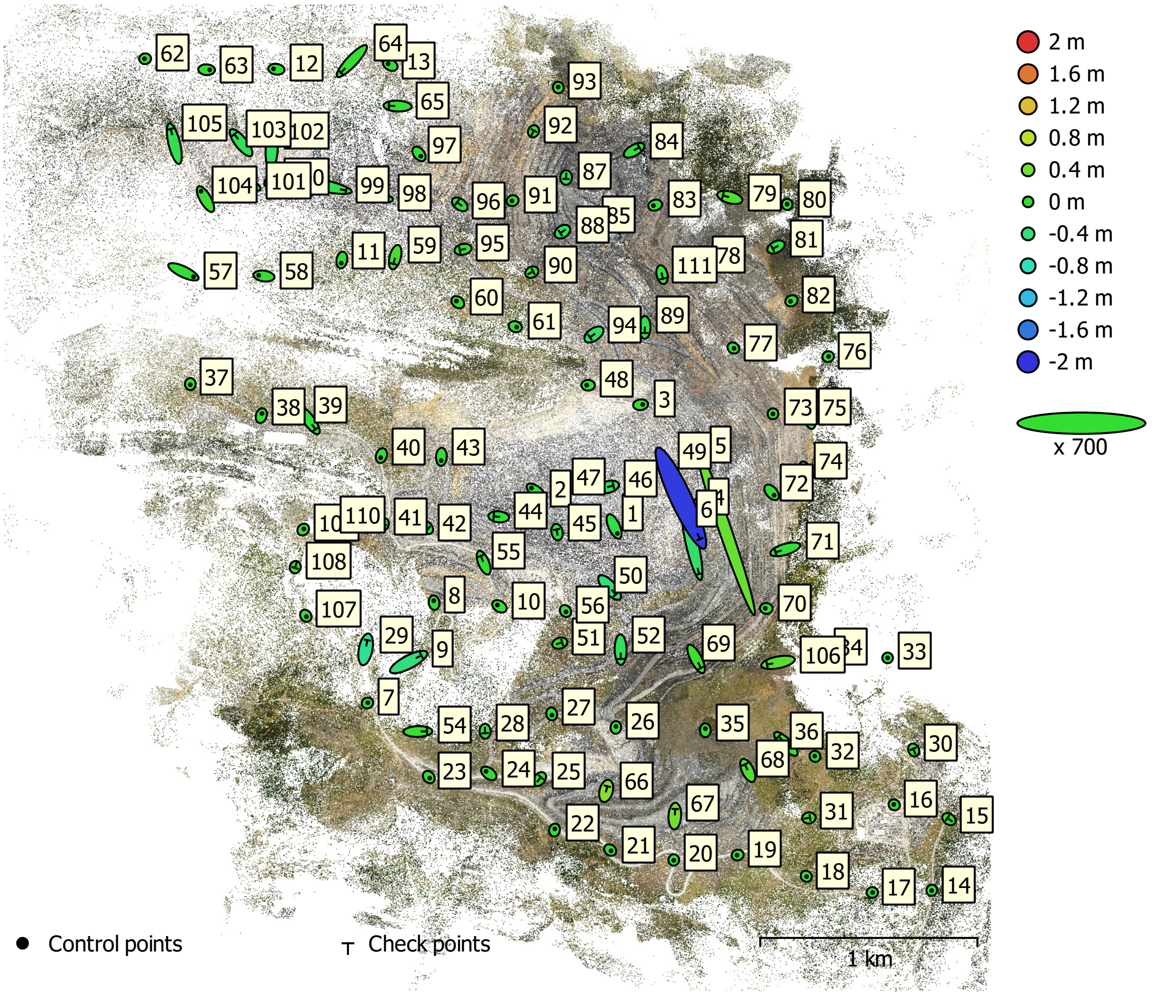

Fig. 8. GCP locations and error estimates.

Z error is represented by ellipse color. X,Y errors are represented by ellipse shape.

Estimated GCP locations are marked with a dot or crossing.

Count	X error (cm)	Y error (cm)	Z error (cm)	XY error (cm)	Total (cm)
55	2.80876	2.91905	2.75573	4.05092	4.89939

Table 8. Control points RMSE.

X - Easting, Y - Northing, Z - Altitude.

Count	X error (cm)	Y error (cm)	Z error (cm)	XY error (cm)	Total (cm)
54	9.93067	18.2507	32.2167	20.7775	38.3356

Table 9. Check points RMSE.

X - Easting, Y - Northing, Z - Altitude.

Label	X error (cm)	Y error (cm)	Z error (cm)	Total (cm)	Image (pix)
1	4.18488	-9.16933	-10.8981	14.8444	0.504 (104)
2	-5.94406	4.49174	0.00230073	7.45035	0.486 (109)
3	2.75812	0.312923	-0.416243	2.80685	0.194 (51)
4	1.96117	9.61486	15.037	17.9556	0.646 (50)
7	0.528131	0.30157	0.0337115	0.609101	0.088 (24)
8	-0.414701	2.60907	-0.142511	2.64566	0.351 (32)
10	-2.70843	1.74396	2.24511	3.92652	0.503 (42)
11	-0.700544	-4.02011	-0.756298	4.15019	0.265 (36)
12	-3.6957	0.551092	0.391104	3.75697	0.411 (26)
13	-2.74135	1.44816	0.451432	3.13305	0.180 (20)
14	-0.185226	-0.374391	0.0549566	0.421304	0.074 (23)
16	-0.55495	0.428881	0.0974445	0.708099	0.090 (34)
17	0.455727	-0.0176847	-0.0555067	0.459436	0.071 (23)
18	0.472376	-0.443135	-0.0558608	0.650099	0.115 (25)
19	-0.931117	-0.245463	-0.0830817	0.966505	0.106 (20)
20	-0.0421483	-0.540815	0.00656871	0.542494	0.125 (16)
21	1.19968	-0.941047	0.142488	1.53138	0.141 (15)
22	0.36031	1.50733	-0.0464942	1.55049	0.123 (13)
23	1.15315	-1.39279	0.139272	1.81356	0.135 (18)
24	-3.56639	2.6563	-0.510476	4.47613	0.334 (27)
26	0.0984313	1.32894	-0.572877	1.4505	0.129 (33)
27	0.344441	-1.47454	0.0594553	1.51541	0.208 (27)
32	-0.244282	0.376235	-0.0107193	0.448711	0.069 (18)
33	0.0118462	0.00444837	-0.00430551	0.0133663	0.001 (3)
34	-0.0115763	0.0739798	-0.0214783	0.0778996	0.015 (4)
35	0.0605019	2.10075	-0.429617	2.14508	0.261 (11)
37	0.00473868	-0.922455	0.27881	0.96368	0.121 (46)
38	0.907163	2.75067	-0.528794	2.94427	0.180 (57)
40	-0.854182	-2.72514	-1.32129	3.14671	0.273 (66)
43	-0.244005	-4.9127	0.292499	4.92744	0.492 (69)
48	-1.89466	0.0581445	-0.522792	1.96632	0.190 (44)

Label	X error (cm)	Y error (cm)	Z error (cm)	Total (cm)	Image (pix)
56	0.747516	-1.05398	-0.0461429	1.29297	0.308 (50)
57	13.378	-6.39558	-1.55944	14.9099	1.484 (14)
58	-6.89235	0.928987	-1.01605	7.0285	0.866 (30)
60	-1.7824	1.42726	0.152788	2.28853	0.164 (32)
61	1.74455	-0.638257	0.59925	1.95191	0.138 (20)
62	-0.812978	-0.103681	0.219171	0.848363	0.147 (17)
63	3.97628	-0.21384	-0.28342	3.9921	0.312 (16)
70	-1.31596	0.0871365	-0.895049	1.59388	0.105 (30)
72	3.27819	-3.7872	-3.26769	5.98057	0.275 (18)
73	-0.0835886	-0.186709	0.225773	0.304665	0.064 (15)
76	0.59384	0.144849	-0.0225702	0.611667	0.095 (9)
77	0.827722	-0.562553	0.0687229	1.00315	0.090 (11)
78	0.419818	-1.5986	-0.195804	1.66436	0.121 (11)
80	-0.00999886	0.341622	0.0267045	0.34281	0.092 (7)
82	-1.26239	-0.659162	-0.2787	1.45114	0.214 (6)
83	-2.19704	-0.662158	-0.186365	2.30221	0.205 (13)
85	0.378305	0.0602178	-0.335161	0.508993	0.207 (10)
91	-0.856795	-0.313038	-0.0962754	0.917256	0.202 (20)
93	-0.0999299	0.594321	-0.0979188	0.610567	0.173 (16)
97	2.41257	-3.05458	0.227513	3.89907	0.363 (40)
100	2.07732	0.461269	-3.37823	3.99255	0.826 (28)
104	-5.93866	10.2965	5.57953	13.1308	1.070 (21)
107	0.807021	-1.17928	-0.467615	1.50355	0.103 (21)
109	0.843719	0.886679	2.17095	2.4922	0.265 (22)
**Total**	**2.80876**	**2.91905**	**2.75573**	**4.89939**	**0.397**

Table 10. Control points.

X - Easting, Y - Northing, Z - Altitude.

Label	X error (cm)	Y error (cm)	Z error (cm)	Total (cm)	Image (pix)
5	-35.6943	104.414	31.5498	114.768	0.609 (38)
6	8.99132	-39.1983	-31.6531	51.1788	0.280 (66)
9	16.7526	8.77323	-41.9712	46.0348	0.246 (29)

Label	X error (cm)	Y error (cm)	Z error (cm)	Total (cm)	Image (pix)
15	-1.98715	1.41356	2.05875	3.19145	0.118 (27)
25	2.06332	2.39508	5.84344	6.64376	0.105 (22)
28	0.115363	-2.54013	-7.10368	7.54505	0.163 (29)
29	2.31628	12.0622	-53.8146	55.1985	0.030 (18)
30	-0.7065	1.60581	0.139461	1.75989	0.104 (20)
31	1.53131	0.224854	8.10732	8.25374	0.135 (26)
36	-9.56423	9.70252	4.09457	14.226	0.136 (14)
39	8.46712	-11.7678	-6.26796	15.7943	0.207 (40)
41	1.26955	1.94601	-9.99242	10.259	0.400 (56)
42	2.64213	-2.34632	-11.0151	11.568	0.375 (48)
44	-6.49002	0.62131	-5.67498	8.6436	0.605 (73)
45	-0.709341	3.47948	-8.93118	9.61124	0.558 (94)
46	6.57202	1.70909	-17.5602	18.8275	0.412 (63)
47	2.77702	5.54188	-15.0602	16.286	0.481 (107)
49	25.0442	-53.8849	-194.517	203.39	0.776 (59)
50	8.09373	-9.04094	-36.4227	38.3909	0.427 (56)
51	3.00529	0.927867	-2.0213	3.73876	0.352 (42)
52	0.227911	-12.3103	-24.045	27.014	0.311 (56)
54	11.9128	0.308948	1.52838	12.0144	0.121 (31)
55	-3.70557	8.90007	-1.04092	9.6967	0.345 (51)
59	-2.28506	-8.87155	10.3681	13.8356	0.262 (9)
64	-14.3746	-15.2914	2.64227	21.1527	0.291 (13)
65	-11.453	0.438032	2.65705	11.7653	0.346 (31)
66	1.65859	6.10488	47.4125	47.8327	0.175 (22)
67	0.535039	9.26357	37.3907	38.5249	0.166 (12)
68	-3.82898	7.95476	17.797	19.8663	0.139 (16)
69	6.2566	-12.0248	7.79232	15.6353	0.216 (28)
71	-12.7806	-3.80563	-4.59048	14.1032	0.230 (24)
74	2.76667	-5.99857	7.09878	9.69691	0.100 (10)
75	-2.10081	6.91863	2.71629	7.72393	0.025 (6)
79	-9.13638	2.53468	18.4582	20.7509	0.142 (8)
81	-4.34362	-2.21553	-9.50826	10.6856	0.241 (6)

Label	X error (cm)	Y error (cm)	Z error (cm)	Total (cm)	Image (pix)
84	6.87576	3.91445	-3.63257	8.706	0.178 (12)
87	-0.0995463	-1.87194	-19.4756	19.5657	0.244 (10)
88	-2.82754	-1.87679	-22.012	22.2721	0.247 (13)
89	-0.0654613	-7.36294	-15.3139	16.9921	0.167 (27)
90	1.71176	0.895349	2.85854	3.45007	0.155 (28)
92	0.513072	1.00902	2.30925	2.57177	0.226 (18)
94	-5.22951	-3.68672	-26.9435	27.6928	0.153 (35)
95	-4.23577	-0.890014	-1.80036	4.68777	0.254 (38)
96	-3.57805	2.66506	-3.94561	5.9559	0.205 (23)
98	16.3567	-3.2961	-1.48114	16.7511	0.578 (32)
99	29.7537	-6.31744	-14.344	33.6295	0.579 (30)
101	-16.8722	7.8634	-2.41987	18.7713	0.669 (26)
102	0.949608	17.1042	-26.0515	31.1791	0.365 (21)
103	-8.86584	11.1921	-20.3051	24.8227	0.478 (20)
105	-4.67526	19.2961	-16.376	25.7366	0.198 (24)
106	-14.5231	-2.86351	19.8306	24.7461	0.088 (13)
108	-0.327424	-1.07367	3.87332	4.03269	0.178 (19)
110	-2.23909	3.20474	-6.68952	7.74813	0.264 (28)
111	1.24766	-5.28503	3.70632	6.57457	0.142 (14)
**Total**	**9.93067**	**18.2507**	**32.2167**	**38.3356**	**0.391**

Table 11. Check points.

X - Easting, Y - Northing, Z - Altitude.

Digital Elevation Model

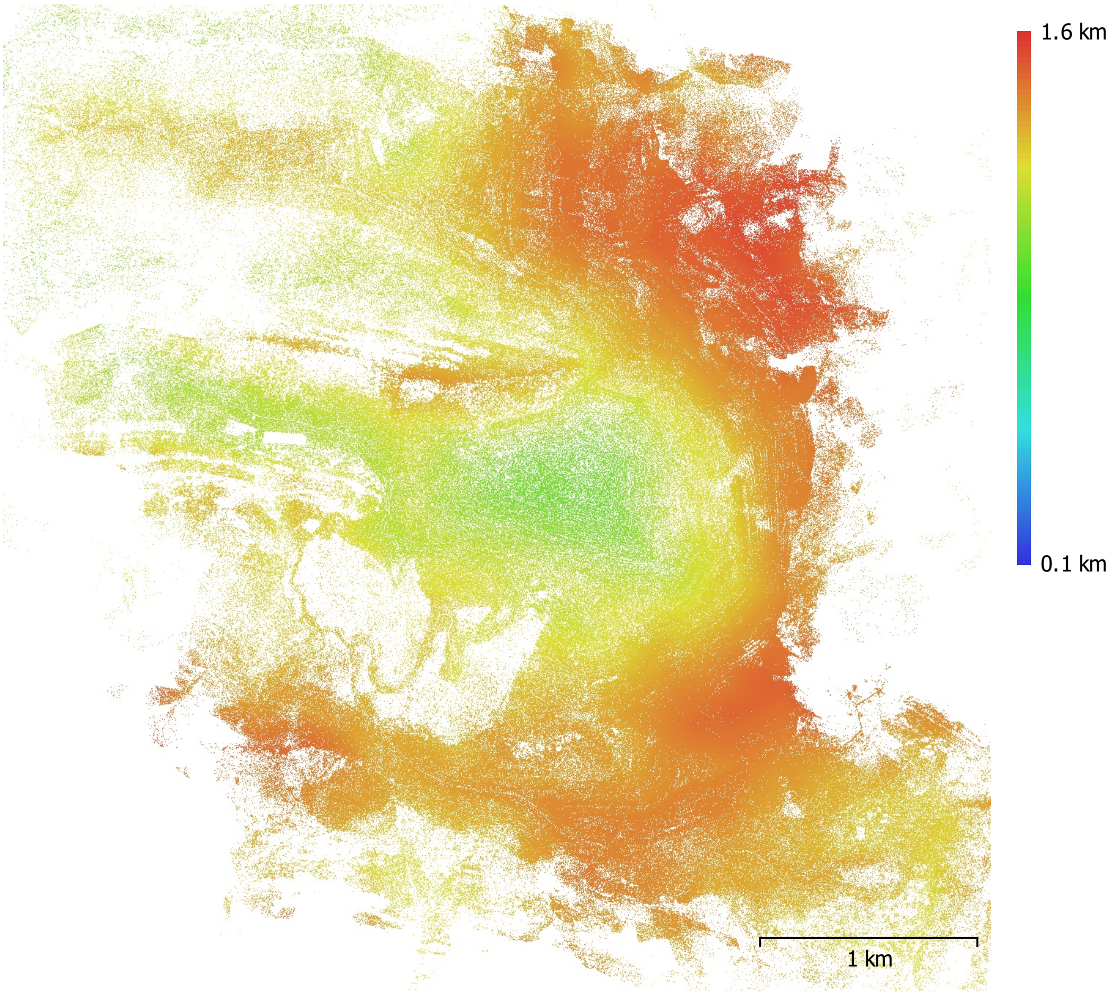

Fig. 9. Reconstructed digital elevation model.

Resolution:	unknown
Point density:	unknown

Processing Parameters

**General**	
Cameras	2595
Aligned cameras	2577
Markers	110
**Shapes**	
Polygon	1
Coordinate system	ETRS89 / UTM zone 30N (EPSG::25830)
Rotation angles	Yaw, Pitch, Roll
**Tie Points**	
Points	1,818,359 of 12,529,745
RMS reprojection error	0.139386 (0.331307 pix)
Max reprojection error	0.299898 (1.81779 pix)
Mean key point size	2.33453 pix
Point colors	3 bands, uint8
Key points	No
Average tie point multiplicity	3.65511
**Alignment parameters**	
Accuracy	High
Generic preselection	Yes
Reference preselection	No
Key point limit	60,000
Key point limit per Mpx	1,000
Tie point limit	0
Exclude stationary tie points	Yes
Guided image matching	No
Adaptive camera model fitting	No
Matching time	4 hours 7 minutes
Matching memory usage	3.73 GB
Alignment time	2 hours 17 minutes
Alignment memory usage	4.82 GB
**Optimization parameters**	
Parameters	f, cx, cy, k1-k3, p1, p2
Adaptive camera model fitting	No
Optimization time	36 seconds
Date created	2023:11:13 15:04:46
Software version	2.0.0.15597
File size	776.62 MB
**System**	
Software name	Agisoft Metashape Professional
Software version	2.0.3 build 16960
OS	Windows 64 bit
RAM	63.90 GB
CPU	Intel(R) Core(TM) i7-7700 CPU @ 3.60GHz
GPU(s)	Quadro M4000
