# Supplementary material for: Automated cleaning of tie point clouds following USGS guidelines in Agisoft Metashape professional (ver. 2.1.0)
Source: MethodsX. 2024 Mar 26;12:102679. doi: 10.1016/j.mex.2024.102679 (PMC10992719; doi:10.1016/j.mex.2024.102679)
Supplement: Supplementary file 3 — The supplementary material includes supplementary text, figures and the processing reports generated by the software. [file mmc3.zip › Lucia_SCC-Default_r2.pdf]

Lucia_SCC-Default_r2

Automatically cleaned sparse cloud using the SCC script (default settings). UAS data provided by Sanz-Ablanedo et al. (2018).

Sanz-Ablanedo, E., Chandler, J. H., Rodríguez-Pérez, J. R., and Ordóñez, C.: Accuracy of Unmanned Aerial Vehicle (UAV) and SfM Photogrammetry Survey as a Function of the Number and Location of Ground Control Points Used, Remote Sensing, 10, 1606, 2018.

28 December 2023

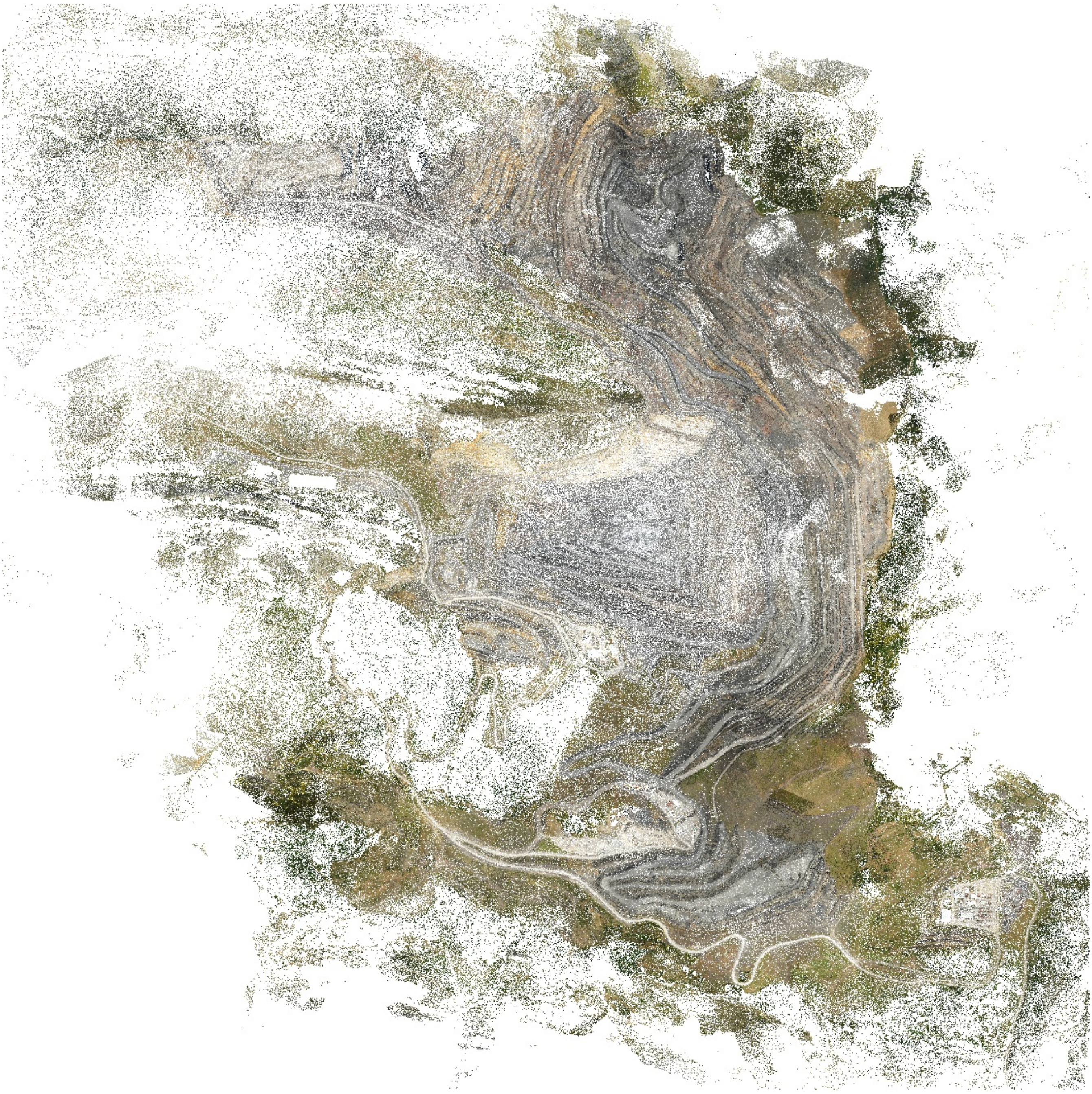

Survey Data

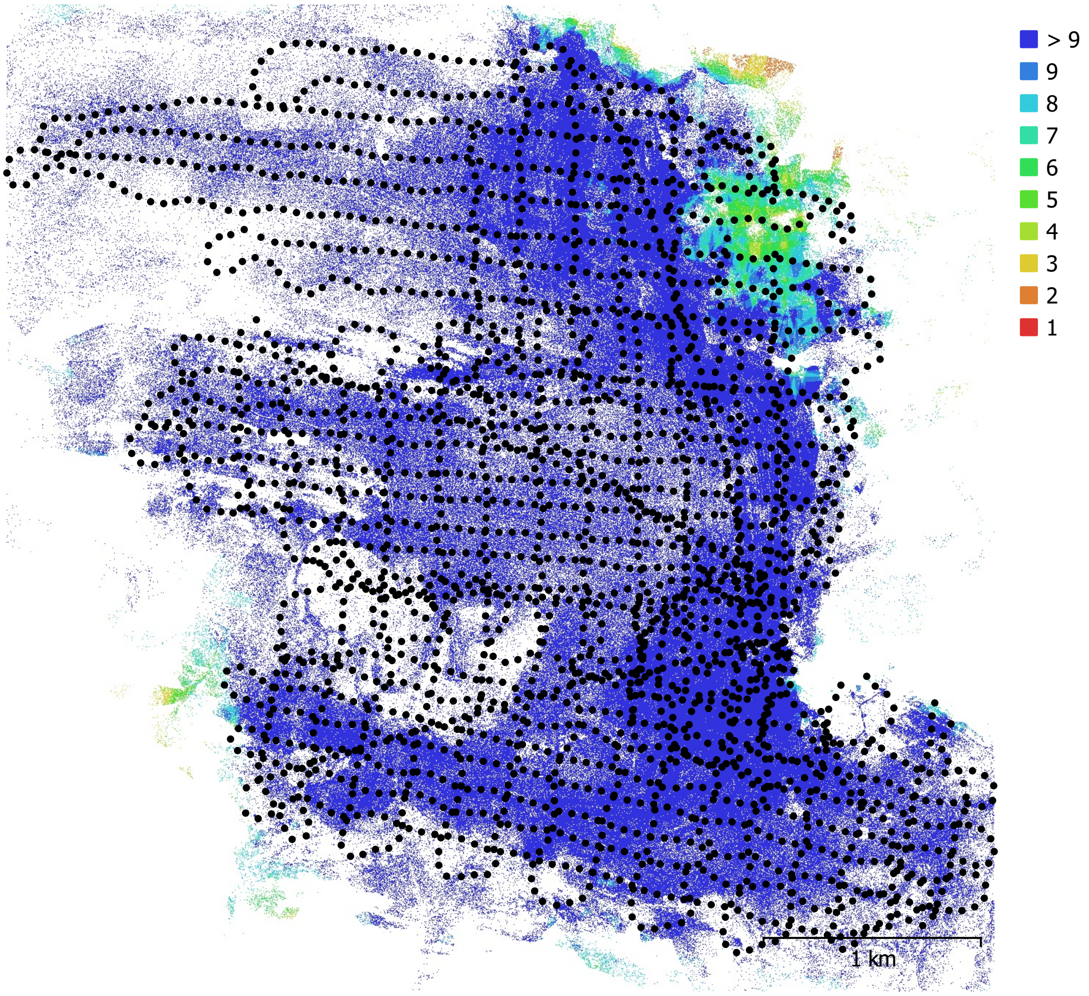

Fig. 1. Camera locations and image overlap.

Number of images:	2,595	Camera stations:	2,577
Flying altitude:	349 m	Tie points:	1,824,039
Ground resolution:	6.2 cm/pix	Projections:	4,327,104
Coverage area:	7.52 km²	Reprojection error:	0.332 pix

Camera Model	Resolution	Focal Length	Pixel Size	Precalibrated
NX500 (20 mm)	6480 x 4320	20 mm	3.7 x 3.7 μm	No
NX500 (20 mm)	6480 x 4320	20 mm	3.7 x 3.7 μm	No
NX500 (20 mm)	6480 x 4320	20 mm	3.7 x 3.7 μm	No
NX500 (20 mm)	6480 x 4320	20 mm	3.7 x 3.7 μm	No
NX500 (20 mm)	6480 x 4320	20 mm	3.7 x 3.7 μm	No

Camera Model	Resolution	Focal Length	Pixel Size	Precalibrated
NX500 (20 mm)	6480 x 4320	20 mm	3.7 x 3.7 μm	No

Table 1. Cameras.

Camera Calibration

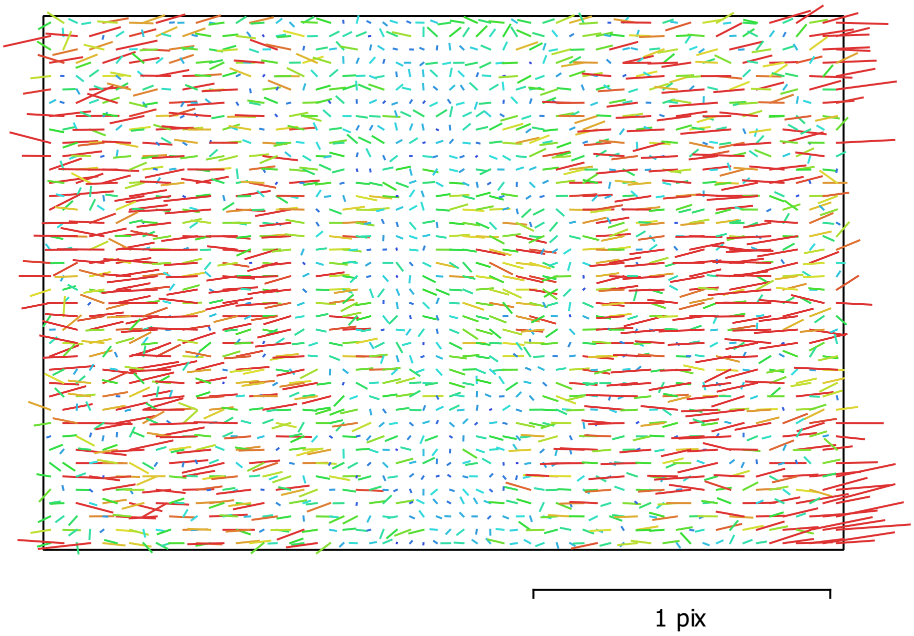

Fig. 2. Image residuals for NX500 (20 mm).

NX500 (20 mm)

200 images

Type	Resolution	Focal Length	Pixel Size
**Frame**	**6480 x 4320**	**20 mm**	**3.7 x 3.7 μm**

	Value	Error	F	Cx	Cy	K1	K2	K3	P1	P2
**F**	**5620.69**	0.049	1.00	0.02	0.01	-0.39	0.33	-0.30	-0.00	0.07
**Cx**	**93.4699**	0.059		1.00	-0.04	0.03	-0.02	0.01	0.82	0.06
**Cy**	**36.6425**	0.068			1.00	-0.00	0.00	-0.01	-0.02	0.78
**K1**	**-0.0120615**	6.3e-05				1.00	-0.96	0.91	0.05	0.00
**K2**	**0.0265161**	0.00031					1.00	-0.98	-0.04	-0.01
**K3**	**-0.0232523**	0.00046						1.00	0.05	0.01
**P1**	**0.00275328**	3.6e-06							1.00	0.04
**P2**	**0.000815696**	4.2e-06								1.00

Table 2. Calibration coefficients and correlation matrix.

Camera Calibration

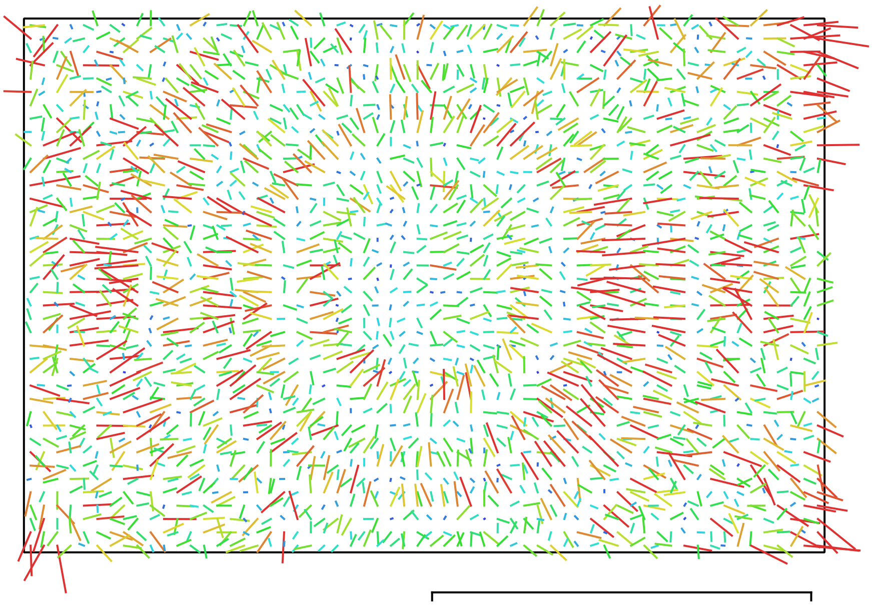

Fig. 3. Image residuals for NX500 (20 mm).

NX500 (20 mm)

462 images

Type	Resolution	Focal Length	Pixel Size
**Frame**	**6480 x 4320**	**20 mm**	**3.7 x 3.7 µm**

	Value	Error	F	Cx	Cy	K1	K2	K3	P1	P2
**F**	**5629.11**	0.041	1.00	-0.16	-0.12	-0.35	0.32	-0.28	-0.04	-0.02
**Cx**	**71.6845**	0.041		1.00	0.06	0.03	-0.03	0.03	0.88	0.02
**Cy**	**44.2637**	0.034			1.00	-0.00	-0.02	0.02	0.05	0.78
**K1**	**-0.0117762**	4.6e-05				1.00	-0.97	0.91	0.03	0.01
**K2**	**0.0272394**	0.00023					1.00	-0.98	-0.04	-0.03
**K3**	**-0.0252547**	0.00034						1.00	0.04	0.03
**P1**	**0.00227438**	2.5e-06							1.00	0.02
**P2**	**0.00118498**	2e-06								1.00

Table 3. Calibration coefficients and correlation matrix.

Camera Calibration

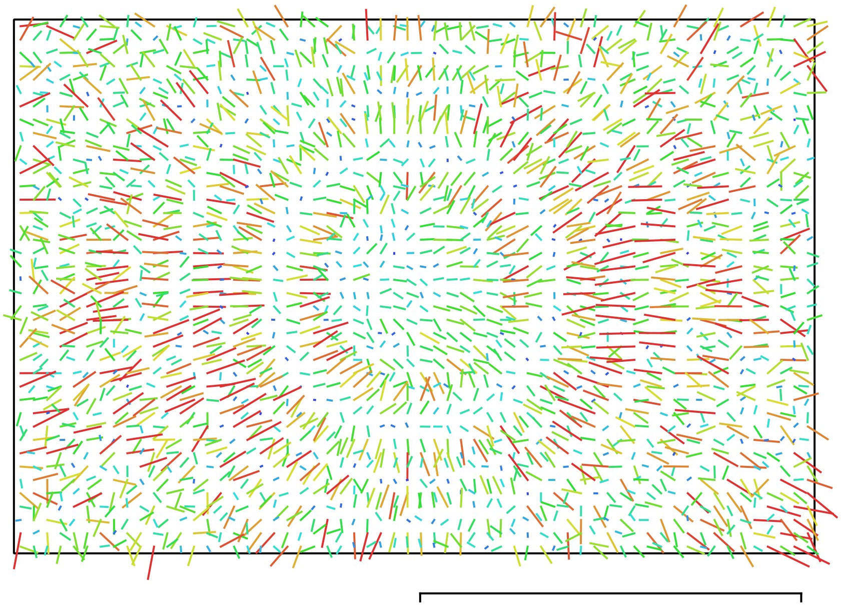

Fig. 4. Image residuals for NX500 (20 mm).

NX500 (20 mm)

530 images

Type	Resolution	Focal Length	Pixel Size
**Frame**	**6480 x 4320**	**20 mm**	**3.7 x 3.7 µm**

	**Value**	**Error**	**F**	**Cx**	**Cy**	**K1**	**K2**	**K3**	**P1**	**P2**
**F**	**5628.56**	0.044	1.00	-0.03	-0.13	-0.26	0.25	-0.22	-0.00	-0.02
**Cx**	**84.1402**	0.038		1.00	-0.02	0.01	-0.01	0.01	0.83	0.00
**Cy**	**35.207**	0.029			1.00	0.01	-0.02	0.01	-0.01	0.68
**K1**	**-0.0120012**	4e-05				1.00	-0.96	0.91	0.02	0.01
**K2**	**0.0304752**	0.00021					1.00	-0.98	-0.02	-0.02
**K3**	**-0.031821**	0.00032						1.00	0.03	0.02
**P1**	**0.00253836**	2.2e-06							1.00	0.02
**P2**	**0.000928506**	1.6e-06								1.00

Table 4. Calibration coefficients and correlation matrix.

Camera Calibration

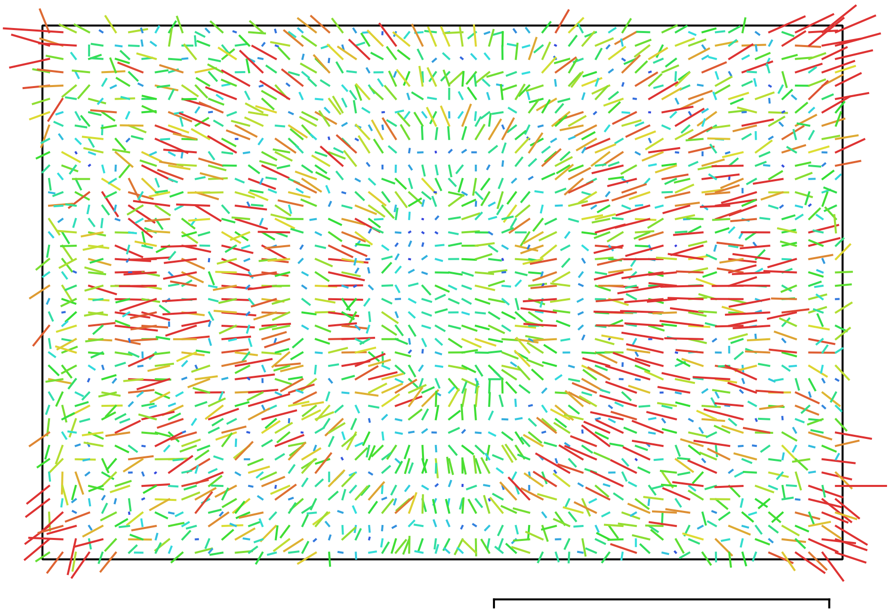

Fig. 5. Image residuals for NX500 (20 mm).

NX500 (20 mm)

513 images

Type	Resolution	Focal Length	Pixel Size
**Frame**	**6480 x 4320**	**20 mm**	**3.7 x 3.7 µm**

	Value	Error	F	Cx	Cy	K1	K2	K3	P1	P2
**F**	**5624.53**	0.052	1.00	-0.08	-0.08	-0.20	0.20	-0.18	0.01	-0.02
**Cx**	**83.9923**	0.034		1.00	-0.01	0.01	-0.01	0.02	0.80	-0.01
**Cy**	**59.8007**	0.027			1.00	0.01	-0.02	0.03	-0.02	0.73
**K1**	**-0.0106019**	3.5e-05				1.00	-0.96	0.90	0.03	0.01
**K2**	**0.0214289**	0.00018					1.00	-0.98	-0.03	-0.01
**K3**	**-0.0149487**	0.00028						1.00	0.04	0.01
**P1**	**0.00251115**	2.1e-06							1.00	-0.02
**P2**	**0.00151097**	1.7e-06								1.00

Table 5. Calibration coefficients and correlation matrix.

Camera Calibration

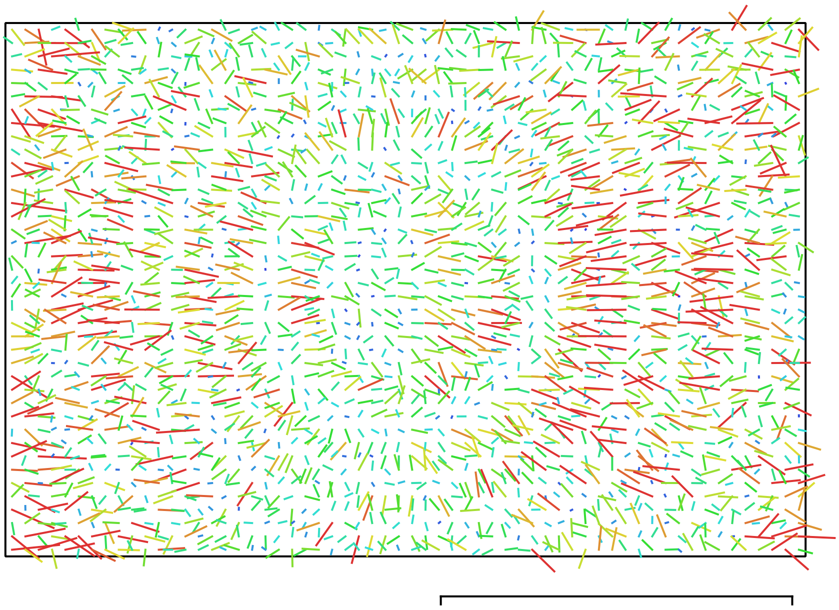

Fig. 6. Image residuals for NX500 (20 mm).

NX500 (20 mm)

412 images

Type	Resolution	Focal Length	Pixel Size
**Frame**	**6480 x 4320**	**20 mm**	**3.7 x 3.7 μm**

	Value	Error	F	Cx	Cy	K1	K2	K3	P1	P2
**F**	**5626.55**	0.045	1.00	0.05	-0.10	-0.37	0.34	-0.31	0.04	-0.01
**Cx**	**89.0702**	0.047		1.00	0.05	-0.01	0.00	0.00	0.88	0.04
**Cy**	**45.3216**	0.038			1.00	-0.02	0.03	-0.04	0.05	0.74
**K1**	**-0.0126198**	5.3e-05				1.00	-0.97	0.91	0.01	-0.01
**K2**	**0.0306704**	0.00027					1.00	-0.98	-0.01	0.00
**K3**	**-0.0317432**	0.0004						1.00	0.02	-0.00
**P1**	**0.00262245**	2.9e-06							1.00	0.05
**P2**	**0.00112835**	2.1e-06								1.00

Table 6. Calibration coefficients and correlation matrix.

Camera Calibration

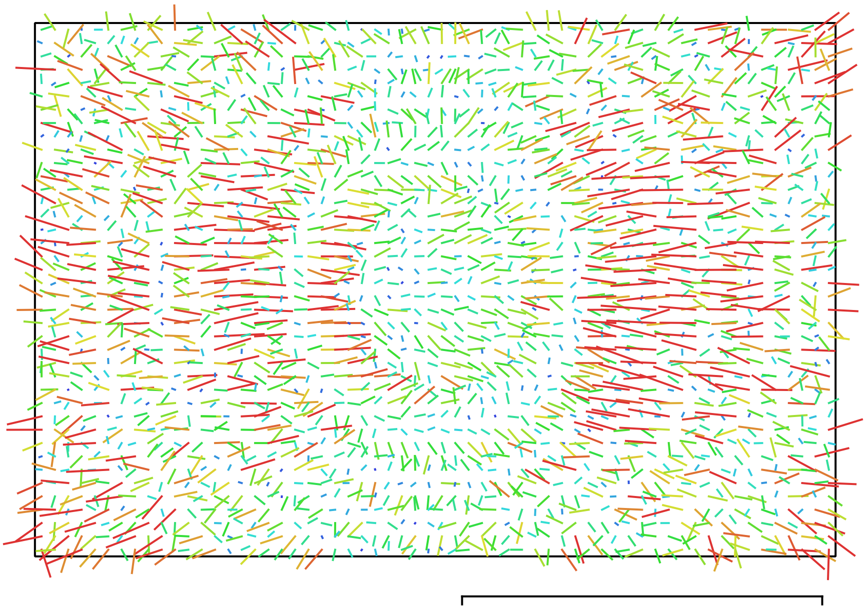

Fig. 7. Image residuals for NX500 (20 mm).

NX500 (20 mm)

478 images

Type	Resolution	Focal Length	Pixel Size
**Frame**	**6480 x 4320**	**20 mm**	**3.7 x 3.7 µm**

	Value	Error	F	Cx	Cy	K1	K2	K3	P1	P2
**F**	**5627.24**	0.032	1.00	-0.00	-0.00	-0.44	0.39	-0.34	-0.01	0.02
**Cx**	**68.8587**	0.04		1.00	-0.01	0.02	-0.01	0.01	0.87	-0.03
**Cy**	**48.253**	0.036			1.00	0.02	-0.03	0.03	-0.02	0.74
**K1**	**-0.0125765**	4.7e-05				1.00	-0.97	0.91	0.01	0.01
**K2**	**0.0354089**	0.00024					1.00	-0.98	-0.01	-0.02
**K3**	**-0.0393068**	0.00037						1.00	0.01	0.02
**P1**	**0.00201776**	2.6e-06							1.00	-0.03
**P2**	**0.00125835**	2.1e-06								1.00

Table 7. Calibration coefficients and correlation matrix.

Ground Control Points

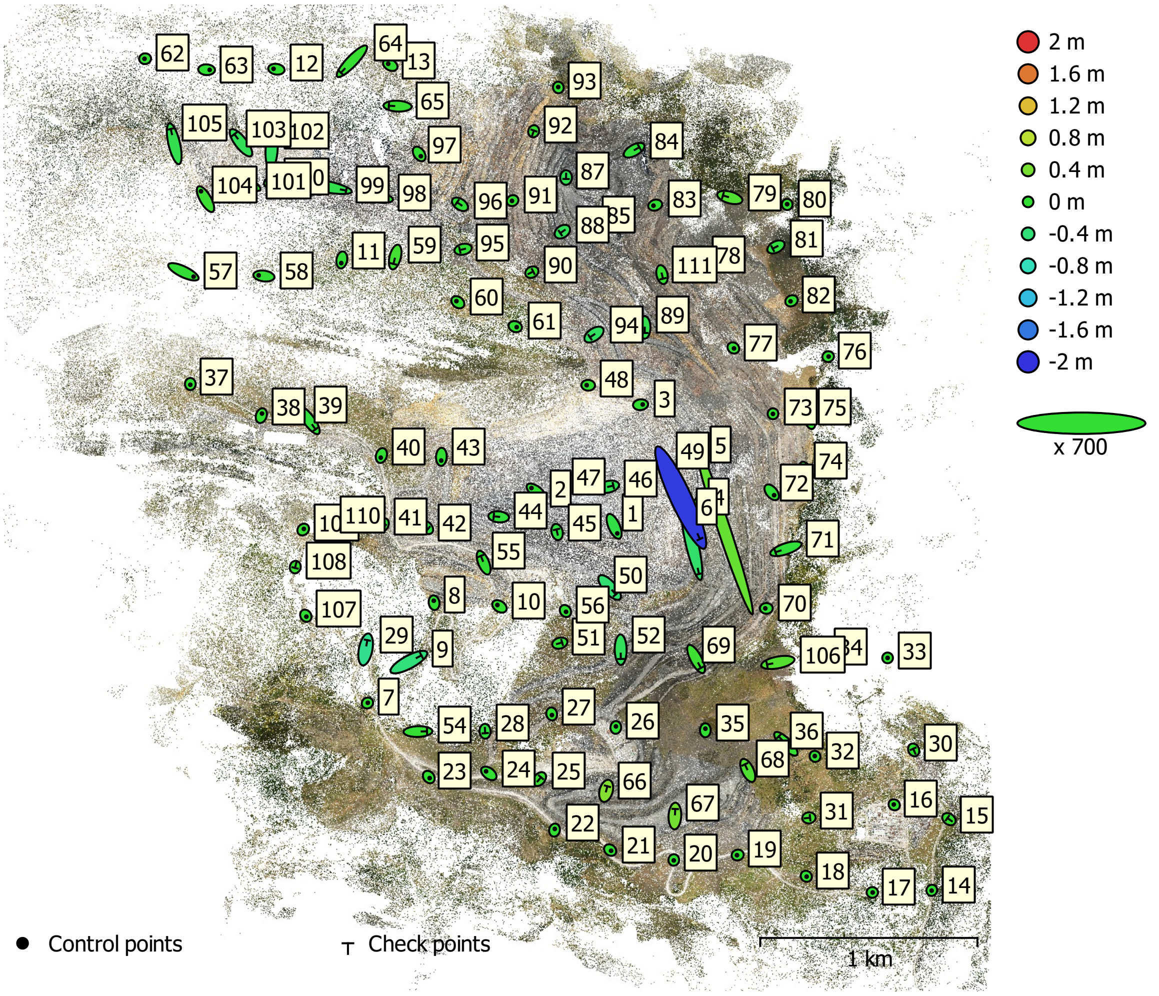

Fig. 8. GCP locations and error estimates.
Z error is represented by ellipse color. X,Y errors are represented by ellipse shape.
Estimated GCP locations are marked with a dot or crossing.

Count	X error (cm)	Y error (cm)	Z error (cm)	XY error (cm)	Total (cm)
55	2.81571	2.95595	2.78072	4.08239	4.93946

Table 8. Control points RMSE.
X - Easting, Y - Northing, Z - Altitude.

Count	X error (cm)	Y error (cm)	Z error (cm)	XY error (cm)	Total (cm)
54	9.84078	18.2461	32.1237	20.7307	38.2321

Table 9. Check points RMSE.
X - Easting, Y - Northing, Z - Altitude.

Label	X error (cm)	Y error (cm)	Z error (cm)	Total (cm)	Image (pix)
1	4.20919	-9.33183	-10.9828	15.014	0.506 (104)
2	-5.94341	4.44297	0.0416448	7.42064	0.488 (109)
3	2.8411	0.305302	-0.465594	2.89514	0.199 (51)
4	1.97048	9.90692	15.1871	18.2395	0.647 (50)
7	0.545942	0.336024	0.0456783	0.64269	0.091 (24)
8	-0.422547	2.64478	-0.143769	2.68218	0.351 (32)
10	-2.73625	1.80225	2.24768	3.97332	0.505 (42)
11	-0.717284	-4.16368	-0.808291	4.30164	0.268 (36)
12	-3.71158	0.564081	0.395399	3.77497	0.414 (26)
13	-2.71567	1.49297	0.458571	3.13275	0.182 (20)
14	-0.186658	-0.372074	0.0539878	0.419756	0.074 (23)
16	-0.556991	0.42619	0.0934393	0.707536	0.091 (34)
17	0.454449	-0.0261807	-0.0577265	0.458849	0.072 (23)
18	0.485275	-0.452221	-0.056182	0.665696	0.116 (25)
19	-0.934037	-0.240438	-0.0873192	0.968432	0.107 (20)
20	-0.0549322	-0.572074	0.0204737	0.57507	0.126 (16)
21	1.21936	-0.942318	0.133773	1.54684	0.144 (15)
22	0.357412	1.55105	-0.0601003	1.59283	0.124 (13)
23	1.17613	-1.43036	0.147604	1.85769	0.137 (18)
24	-3.62521	2.66224	-0.507565	4.52629	0.336 (27)
26	0.126247	1.35371	-0.582005	1.47892	0.131 (33)
27	0.333631	-1.50277	0.0581645	1.54046	0.214 (27)
32	-0.255678	0.371545	-0.0103075	0.451136	0.070 (18)
33	0.0118872	0.00380193	-0.00555069	0.013659	0.001 (3)
34	-0.0114293	0.0760915	-0.023158	0.0803545	0.015 (4)
35	0.051112	2.14887	-0.422542	2.19062	0.263 (11)
37	-0.00405754	-0.93264	0.277394	0.973027	0.121 (46)
38	0.911183	2.76705	-0.511865	2.95784	0.180 (57)
40	-0.848475	-2.78395	-1.3244	3.19755	0.274 (66)
43	-0.231416	-4.88111	0.300196	4.89581	0.493 (69)
48	-1.9403	0.0877212	-0.533893	2.01432	0.192 (44)

Label	X error (cm)	Y error (cm)	Z error (cm)	Total (cm)	Image (pix)
56	0.769034	-1.09822	-0.0503888	1.34165	0.309 (50)
57	13.4018	-6.40795	-1.55683	14.9363	1.487 (14)
58	-6.86186	0.902772	-1.05019	7.00021	0.870 (30)
60	-1.8633	1.4626	0.168745	2.37478	0.168 (32)
61	1.8433	-0.671359	0.616892	2.05646	0.142 (20)
62	-0.808335	-0.103407	0.220601	0.844253	0.148 (17)
63	3.99724	-0.198372	-0.282114	4.01209	0.316 (16)
70	-1.36879	0.0235685	-0.937878	1.65944	0.107 (30)
72	3.22172	-3.82985	-3.30007	5.99481	0.277 (18)
73	-0.0369533	-0.166579	0.234251	0.289807	0.065 (15)
76	0.597672	0.153953	-0.0263736	0.617745	0.096 (9)
77	0.847168	-0.598191	0.0788072	1.04007	0.090 (11)
78	0.42985	-1.61738	-0.18085	1.68327	0.120 (11)
80	-0.0100574	0.342588	0.0248236	0.343633	0.092 (7)
82	-1.27156	-0.664545	-0.279666	1.46175	0.214 (6)
83	-2.21287	-0.656244	-0.193841	2.31626	0.205 (13)
85	0.402298	0.0752082	-0.330569	0.526095	0.208 (10)
91	-0.764576	-0.379042	-0.040674	0.854344	0.215 (20)
93	-0.148566	0.580723	-0.0981958	0.607416	0.175 (16)
97	2.15265	-2.9816	0.196076	3.6827	0.365 (40)
100	2.21456	0.495848	-3.41436	4.09976	0.830 (28)
104	-5.96645	10.3356	5.61585	13.1895	1.072 (21)
107	0.788297	-1.17254	-0.473234	1.49004	0.104 (21)
109	0.850397	0.860122	2.18086	2.49382	0.266 (22)
**Total**	**2.81571**	**2.95595**	**2.78072**	**4.93946**	**0.399**

Table 10. Control points.
X - Easting, Y - Northing, Z - Altitude.

Label	X error (cm)	Y error (cm)	Z error (cm)	Total (cm)	Image (pix)
5	-34.34	103.803	30.5371	113.52	0.620 (38)
6	9.31336	-39.7985	-31.8325	51.8071	0.290 (66)
9	16.4334	8.81553	-41.153	45.1812	0.247 (29)

Label	X error (cm)	Y error (cm)	Z error (cm)	Total (cm)	Image (pix)
15	-2.00865	1.42395	2.24519	3.33215	0.118 (27)
25	2.04438	2.39115	5.42536	6.27149	0.106 (22)
28	0.148577	-2.49	-7.0087	7.43936	0.164 (29)
29	2.32829	12.1938	-53.6958	55.1122	0.030 (18)
30	-0.672223	1.46788	0.518846	1.69581	0.105 (20)
31	1.48476	0.160217	7.99386	8.13215	0.136 (26)
36	-9.49694	9.64712	4.11424	14.1487	0.137 (14)
39	8.50801	-11.8391	-6.14906	15.8228	0.208 (40)
41	1.22441	2.01742	-9.94748	10.2236	0.403 (56)
42	2.65678	-2.31832	-10.8569	11.4151	0.377 (48)
44	-6.46037	0.617692	-5.64747	8.60302	0.607 (73)
45	-0.79581	3.39178	-8.88779	9.54622	0.559 (94)
46	6.6037	1.52803	-17.5865	18.8476	0.413 (63)
47	2.72549	5.38412	-14.9795	16.1494	0.484 (107)
49	25.2896	-54.5466	-194.6	203.676	0.783 (59)
50	8.03238	-9.10846	-36.1629	38.1476	0.429 (56)
51	3.00256	0.905771	-1.85001	3.6412	0.353 (42)
52	0.16927	-12.2317	-23.6682	26.6425	0.312 (56)
54	11.8956	0.341727	1.57325	12.0041	0.123 (31)
55	-3.67796	8.88837	-1.0164	9.67283	0.346 (51)
59	-2.28701	-9.03088	10.8274	14.2836	0.265 (9)
64	-14.4521	-15.0983	1.94398	20.9905	0.292 (13)
65	-11.453	0.522494	2.46516	11.7269	0.345 (31)
66	1.66859	6.02579	46.3318	46.7518	0.174 (22)
67	0.426672	9.2263	37.6094	38.7269	0.168 (12)
68	-3.79808	7.92754	17.94	19.9778	0.140 (16)
69	6.35432	-11.8759	7.6121	15.4712	0.219 (28)
71	-13.6469	-4.57603	-4.68847	15.138	0.234 (24)
74	2.7758	-5.8385	7.23118	9.69965	0.099 (10)
75	-2.10239	6.92575	2.74634	7.74135	0.026 (6)
79	-9.0857	2.58374	18.5443	20.8114	0.142 (8)
81	-4.31689	-2.23226	-9.28132	10.4767	0.242 (6)

Label	X error (cm)	Y error (cm)	Z error (cm)	Total (cm)	Image (pix)
84	6.93556	3.88165	-4.06922	8.92904	0.181 (12)
87	-0.0426903	-1.9004	-19.5101	19.6025	0.247 (10)
88	-2.73653	-1.87648	-21.9858	22.2347	0.250 (13)
89	0.0169438	-7.33789	-14.8295	16.5456	0.170 (27)
90	1.72205	0.897849	2.30407	3.01336	0.157 (28)
92	0.319455	0.915851	2.174	2.38057	0.228 (18)
94	-5.29078	-3.59715	-26.5767	27.3359	0.157 (35)
95	-4.35341	-0.993472	-1.34138	4.66246	0.257 (38)
96	-3.83528	2.62682	-3.25227	5.67334	0.220 (23)
98	16.2961	-2.84037	-1.62874	16.6218	0.581 (32)
99	29.423	-5.81715	-14.4251	33.2812	0.587 (30)
101	-16.7523	7.94178	-2.31492	18.6834	0.670 (26)
102	1.05144	17.1963	-25.6974	30.9383	0.366 (21)
103	-8.74199	11.2616	-19.9751	24.5408	0.479 (20)
105	-4.58648	19.4708	-15.9685	25.5957	0.197 (24)
106	-14.2802	-2.76785	19.5245	24.3473	0.089 (13)
108	-0.273393	-1.10068	3.79125	3.95725	0.178 (19)
110	-2.30543	3.15078	-6.46482	7.55224	0.266 (28)
111	1.29315	-5.36035	4.09676	6.86943	0.143 (14)
**Total**	**9.84078**	**18.2461**	**32.1237**	**38.2321**	**0.394**

Table 11. Check points.

X - Easting, Y - Northing, Z - Altitude.

Digital Elevation Model

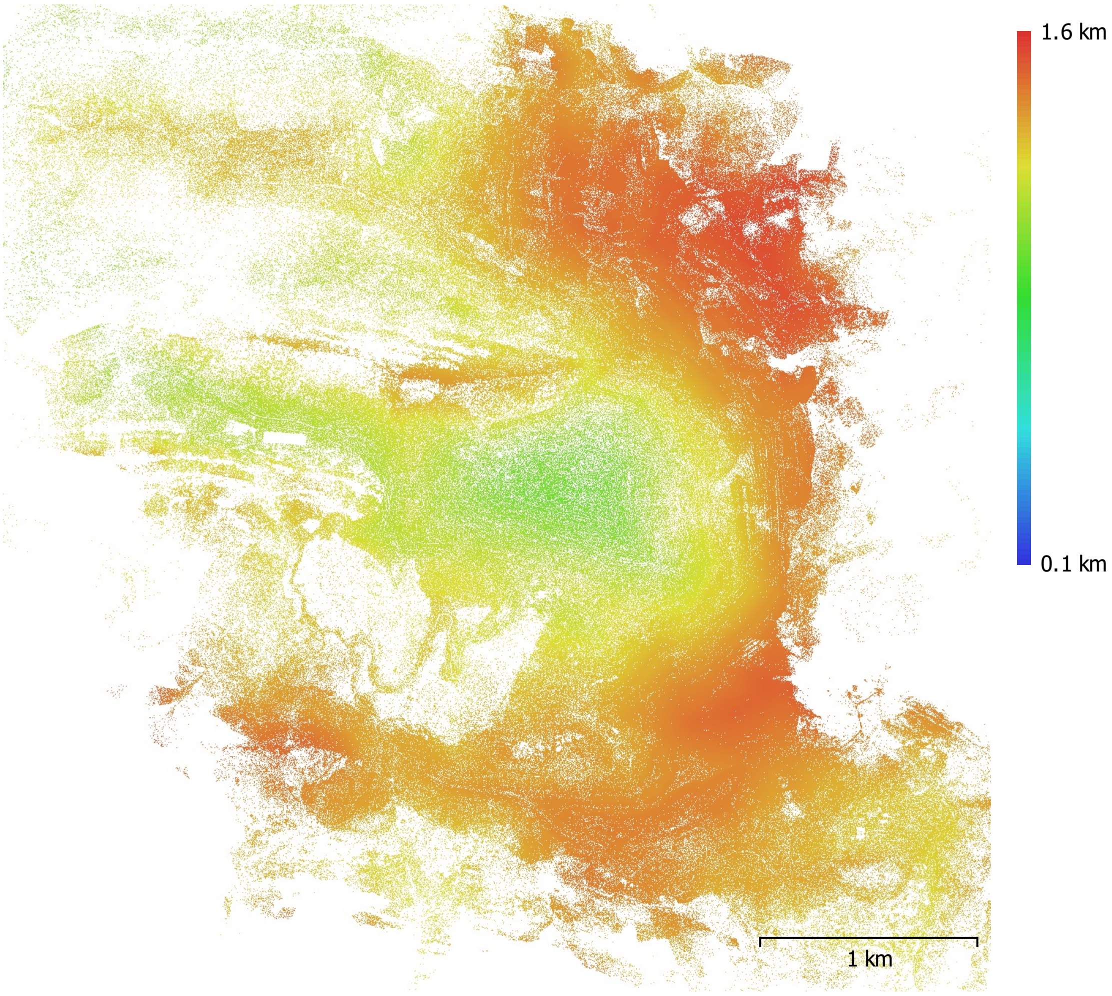

Fig. 9. Reconstructed digital elevation model.

Resolution:	unknown
Point density:	unknown

Processing Parameters

**General**	
Cameras	2595
Aligned cameras	2577
Markers	110
**Shapes**	
Polygon	1
Coordinate system	ETRS89 / UTM zone 30N (EPSG::25830)
Rotation angles	Yaw, Pitch, Roll
**Tie Points**	
Points	1,824,039 of 12,529,745
RMS reprojection error	0.139655 (0.331941 pix)
Max reprojection error	0.300001 (1.81849 pix)
Mean key point size	2.3346 pix
Point colors	3 bands, uint8
Key points	No
Average tie point multiplicity	3.65511
**Alignment parameters**	
Accuracy	High
Generic preselection	Yes
Reference preselection	No
Key point limit	60,000
Key point limit per Mpx	1,000
Tie point limit	0
Exclude stationary tie points	Yes
Guided image matching	No
Adaptive camera model fitting	No
Matching time	4 hours 7 minutes
Matching memory usage	3.73 GB
Alignment time	2 hours 17 minutes
Alignment memory usage	4.82 GB
**Optimization parameters**	
Parameters	f, cx, cy, k1-k3, p1, p2
Adaptive camera model fitting	No
Optimization time	53 seconds
Date created	2023:11:13 15:04:46
Software version	2.0.0.15597
File size	776.75 MB
**System**	
Software name	Agisoft Metashape Professional
Software version	2.0.3 build 16960
OS	Windows 64 bit
RAM	63.90 GB
CPU	Intel(R) Core(TM) i7-7700 CPU @ 3.60GHz
GPU(s)	Quadro M4000
