# Supplementary material for: Automated cleaning of tie point clouds following USGS guidelines in Agisoft Metashape professional (ver. 2.1.0)
Source: MethodsX. 2024 Mar 26;12:102679. doi: 10.1016/j.mex.2024.102679 (PMC10992719; doi:10.1016/j.mex.2024.102679)
Supplement: Supplementary file 3 — The supplementary material includes supplementary text, figures and the processing reports generated by the software. [file mmc3.zip › Lucia_SCC-Default_r3.pdf]

Lucia_SCC-Default_r3

Automatically cleaned sparse cloud using the SCC script (default settings). UAS data provided by Sanz-Ablanedo et al. (2018).

Sanz-Ablanedo, E., Chandler, J. H., Rodríguez-Pérez, J. R., and Ordóñez, C.: Accuracy of Unmanned Aerial Vehicle (UAV) and SfM Photogrammetry Survey as a Function of the Number and Location of Ground Control Points Used, Remote Sensing, 10, 1606, 2018.

28 December 2023

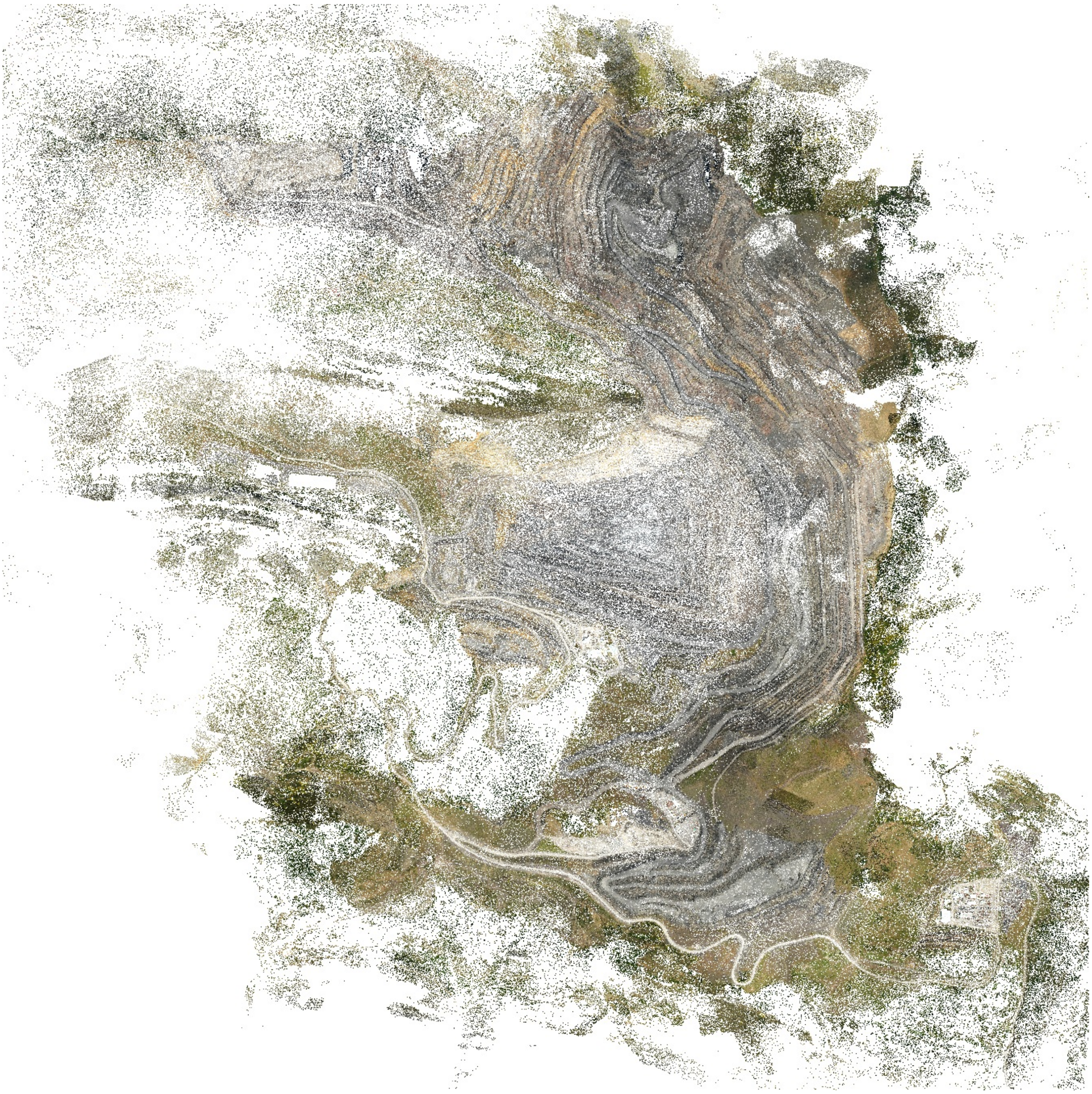

Survey Data

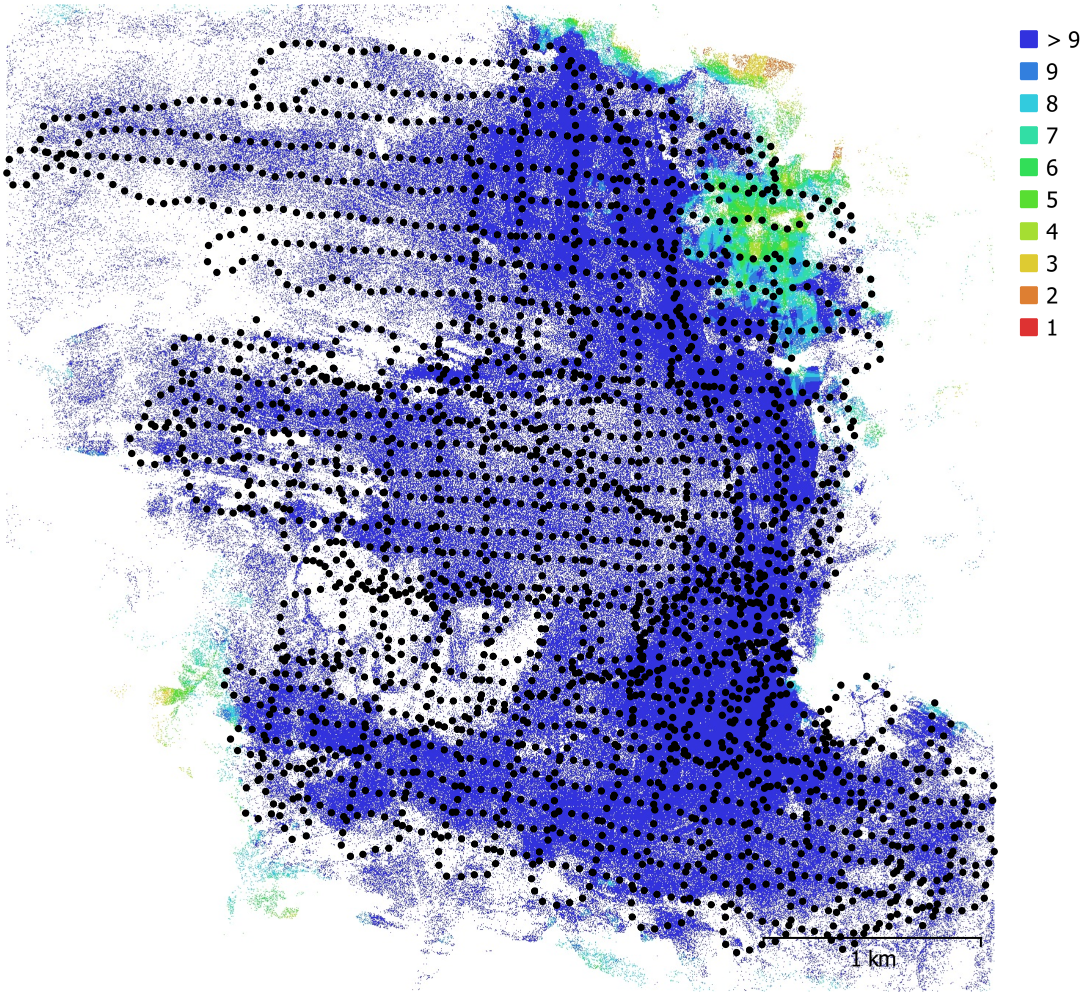

Fig. 1. Camera locations and image overlap.

Number of images:	2,595	Camera stations:	2,577
Flying altitude:	349 m	Tie points:	1,812,511
Ground resolution:	6.2 cm/pix	Projections:	4,292,869
Coverage area:	7.52 km²	Reprojection error:	0.331 pix

Camera Model	Resolution	Focal Length	Pixel Size	Precalibrated
NX500 (20 mm)	6480 x 4320	20 mm	3.7 x 3.7 μm	No
NX500 (20 mm)	6480 x 4320	20 mm	3.7 x 3.7 μm	No
NX500 (20 mm)	6480 x 4320	20 mm	3.7 x 3.7 μm	No
NX500 (20 mm)	6480 x 4320	20 mm	3.7 x 3.7 μm	No
NX500 (20 mm)	6480 x 4320	20 mm	3.7 x 3.7 μm	No

Camera Model	Resolution	Focal Length	Pixel Size	Precalibrated
NX500 (20 mm)	6480 x 4320	20 mm	3.7 x 3.7 μm	No

Table 1. Cameras.

Camera Calibration

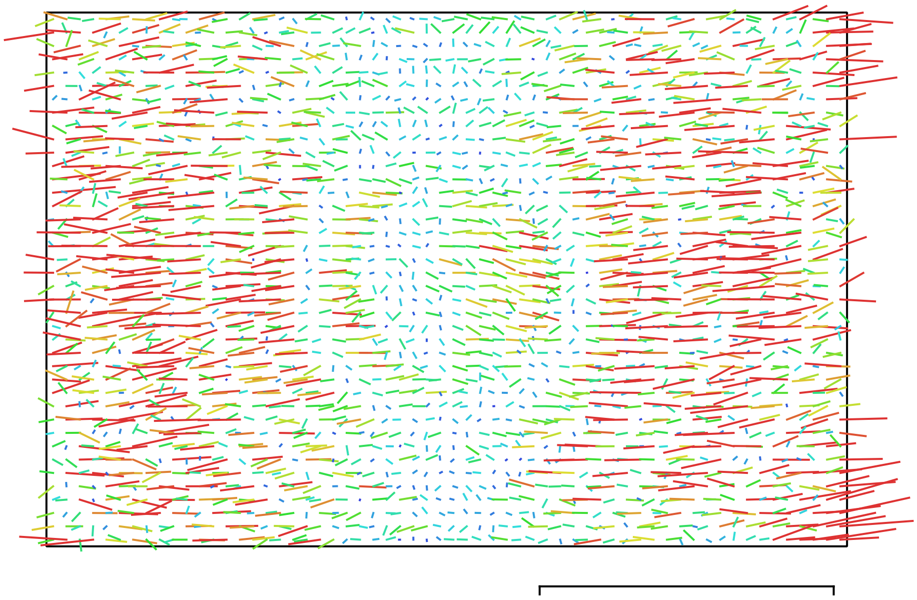

Fig. 2. Image residuals for NX500 (20 mm).

NX500 (20 mm)

200 images

Type	Resolution	Focal Length	Pixel Size
**Frame**	**6480 x 4320**	**20 mm**	**3.7 x 3.7 μm**

	**Value**	**Error**	**F**	**Cx**	**Cy**	**K1**	**K2**	**K3**	**P1**	**P2**
**F**	**5620.37**	0.05	1.00	0.02	0.01	-0.38	0.33	-0.30	-0.00	0.07
**Cx**	**93.2572**	0.06		1.00	-0.04	0.03	-0.02	0.01	0.82	0.06
**Cy**	**36.911**	0.068			1.00	-0.00	0.00	-0.00	-0.02	0.78
**K1**	**-0.0120472**	6.3e-05				1.00	-0.96	0.91	0.05	0.00
**K2**	**0.0262535**	0.00031					1.00	-0.98	-0.05	-0.01
**K3**	**-0.0227762**	0.00046						1.00	0.05	0.01
**P1**	**0.00274542**	3.6e-06							1.00	0.04
**P2**	**0.000824831**	4.2e-06								1.00

Table 2. Calibration coefficients and correlation matrix.

Camera Calibration

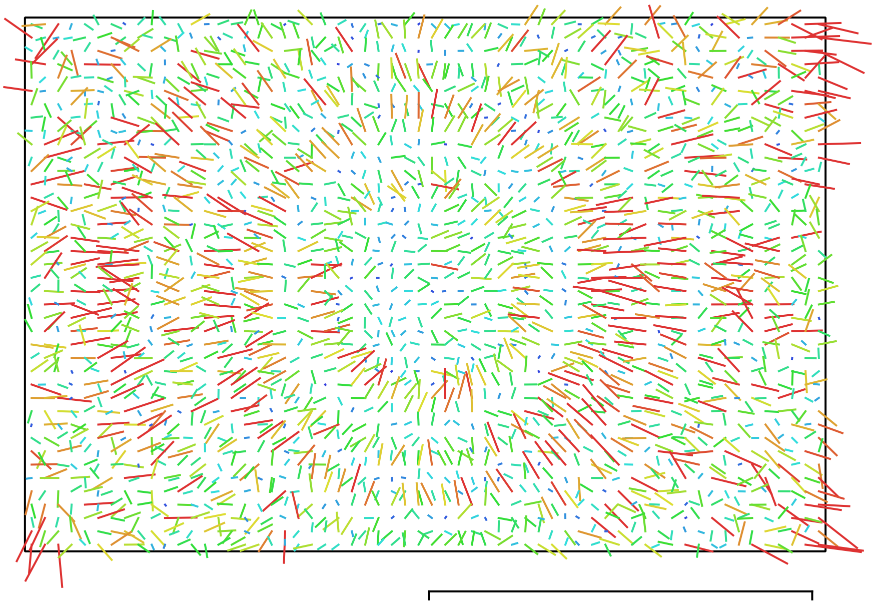

Fig. 3. Image residuals for NX500 (20 mm).

NX500 (20 mm)

462 images

Type	Resolution	Focal Length	Pixel Size
**Frame**	**6480 x 4320**	**20 mm**	**3.7 x 3.7 µm**

	**Value**	**Error**	**F**	**Cx**	**Cy**	**K1**	**K2**	**K3**	**P1**	**P2**
**F**	**5629.02**	0.041	1.00	-0.16	-0.12	-0.34	0.32	-0.28	-0.03	-0.02
**Cx**	**71.5582**	0.041		1.00	0.06	0.03	-0.03	0.03	0.88	0.02
**Cy**	**44.2164**	0.035			1.00	-0.00	-0.02	0.02	0.05	0.78
**K1**	**-0.0117338**	4.7e-05				1.00	-0.97	0.91	0.03	0.01
**K2**	**0.0268661**	0.00023					1.00	-0.98	-0.03	-0.03
**K3**	**-0.0245587**	0.00034						1.00	0.04	0.03
**P1**	**0.00226746**	2.6e-06							1.00	0.03
**P2**	**0.00118287**	2e-06								1.00

Table 3. Calibration coefficients and correlation matrix.

Camera Calibration

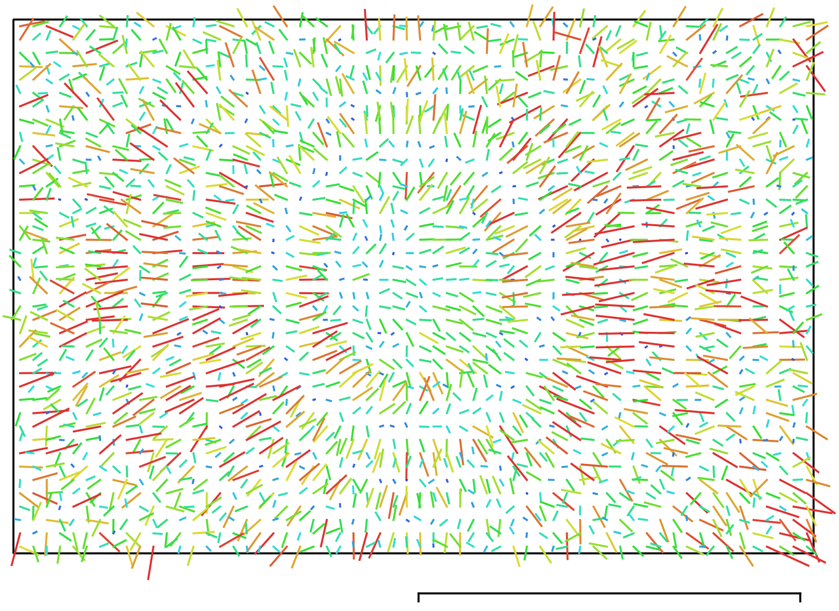

Fig. 4. Image residuals for NX500 (20 mm).

NX500 (20 mm)

530 images

Type	Resolution	Focal Length	Pixel Size
**Frame**	**6480 x 4320**	**20 mm**	**3.7 x 3.7 µm**

	**Value**	**Error**	**F**	**Cx**	**Cy**	**K1**	**K2**	**K3**	**P1**	**P2**
**F**	**5628.61**	0.044	1.00	-0.03	-0.13	-0.26	0.25	-0.22	-0.00	-0.02
**Cx**	**84.0861**	0.038		1.00	-0.02	0.01	-0.01	0.01	0.83	0.00
**Cy**	**35.1836**	0.029			1.00	0.01	-0.02	0.01	-0.01	0.68
**K1**	**-0.0120066**	4e-05				1.00	-0.96	0.91	0.02	0.01
**K2**	**0.0305185**	0.00021					1.00	-0.98	-0.02	-0.02
**K3**	**-0.0319222**	0.00032						1.00	0.03	0.02
**P1**	**0.00253771**	2.2e-06							1.00	0.02
**P2**	**0.000927044**	1.6e-06								1.00

Table 4. Calibration coefficients and correlation matrix.

Camera Calibration

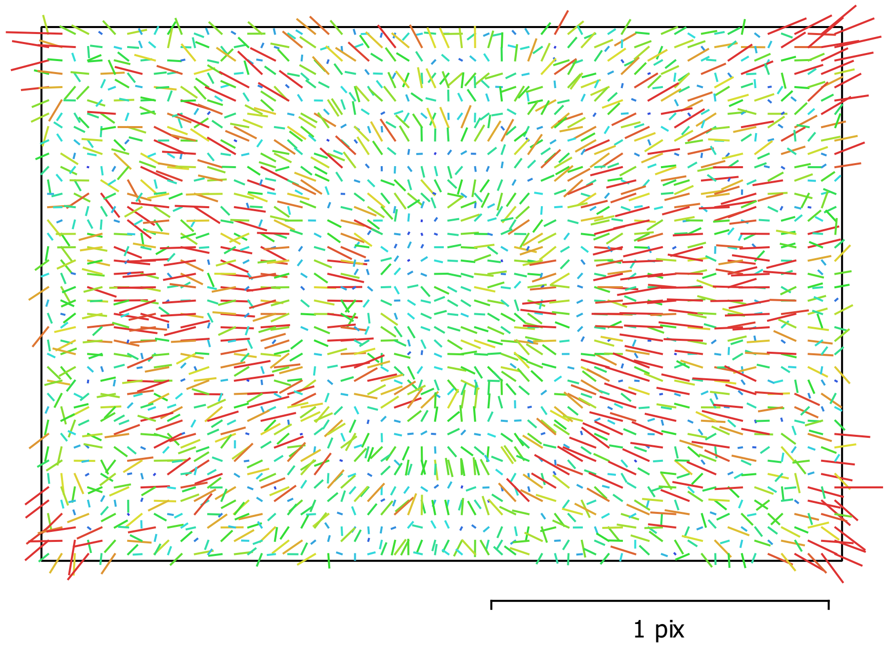

Fig. 5. Image residuals for NX500 (20 mm).

NX500 (20 mm)

513 images

Type	Resolution	Focal Length	Pixel Size
**Frame**	**6480 x 4320**	**20 mm**	**3.7 x 3.7 μm**

	Value	Error	F	Cx	Cy	K1	K2	K3	P1	P2
**F**	**5624.25**	0.052	1.00	-0.08	-0.08	-0.21	0.21	-0.19	0.00	-0.03
**Cx**	**83.9967**	0.034		1.00	-0.01	0.01	-0.01	0.02	0.80	-0.01
**Cy**	**59.966**	0.028			1.00	0.01	-0.01	0.01	-0.02	0.72
**K1**	**-0.0106685**	3.6e-05				1.00	-0.96	0.90	0.03	0.01
**K2**	**0.0217859**	0.00019					1.00	-0.98	-0.03	-0.01
**K3**	**-0.0155825**	0.00028						1.00	0.04	0.01
**P1**	**0.00251119**	2.1e-06							1.00	-0.02
**P2**	**0.00151148**	1.7e-06								1.00

Table 5. Calibration coefficients and correlation matrix.

Camera Calibration

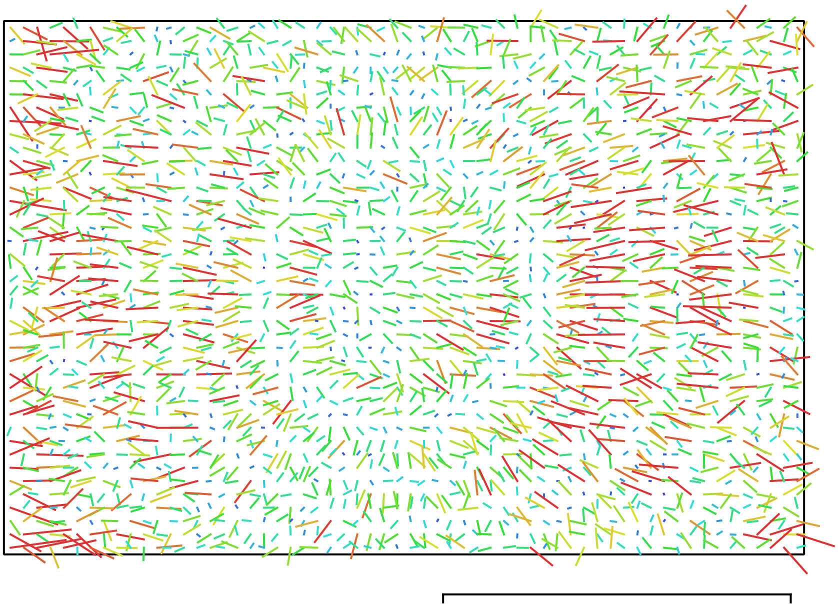

Fig. 6. Image residuals for NX500 (20 mm).

NX500 (20 mm)

412 images

Type	Resolution	Focal Length	Pixel Size
**Frame**	**6480 x 4320**	**20 mm**	**3.7 x 3.7 µm**

	**Value**	**Error**	**F**	**Cx**	**Cy**	**K1**	**K2**	**K3**	**P1**	**P2**
**F**	**5626.66**	0.045	1.00	0.04	-0.10	-0.37	0.34	-0.32	0.03	-0.01
**Cx**	**88.8911**	0.048		1.00	0.05	-0.01	0.01	-0.00	0.88	0.04
**Cy**	**45.4622**	0.038			1.00	-0.02	0.03	-0.04	0.05	0.74
**K1**	**-0.012676**	5.3e-05				1.00	-0.97	0.91	0.00	-0.01
**K2**	**0.0312779**	0.00027					1.00	-0.98	-0.01	0.00
**K3**	**-0.0329823**	0.0004						1.00	0.02	-0.01
**P1**	**0.00261617**	2.9e-06							1.00	0.05
**P2**	**0.00113462**	2.2e-06								1.00

Table 6. Calibration coefficients and correlation matrix.

Camera Calibration

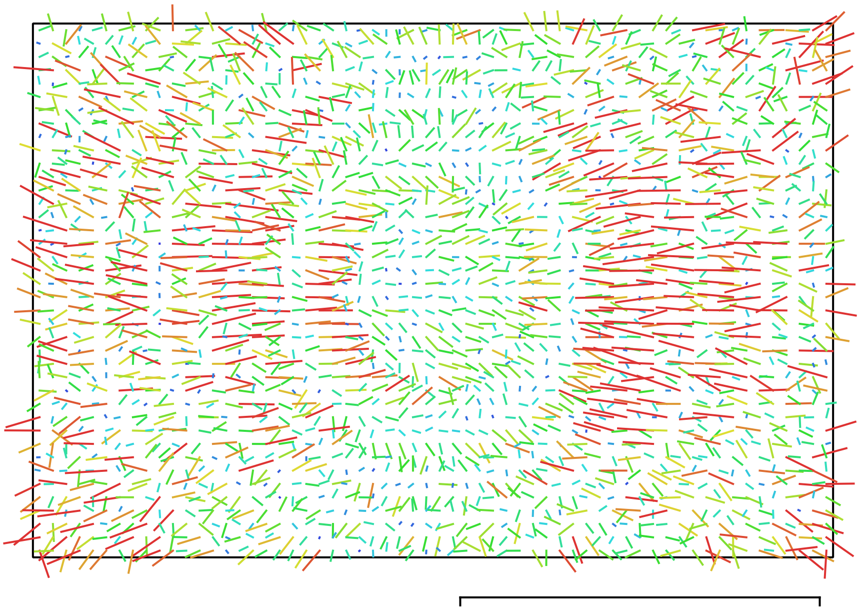

Fig. 7. Image residuals for NX500 (20 mm).

NX500 (20 mm)

478 images

Type	Resolution	Focal Length	Pixel Size
**Frame**	**6480 x 4320**	**20 mm**	**3.7 x 3.7 μm**

	Value	Error	F	Cx	Cy	K1	K2	K3	P1	P2
**F**	**5627.25**	0.032	1.00	-0.00	-0.00	-0.44	0.39	-0.34	-0.01	0.02
**Cx**	**68.9327**	0.04		1.00	-0.01	0.02	-0.01	0.01	0.87	-0.03
**Cy**	**48.155**	0.036			1.00	0.02	-0.03	0.03	-0.02	0.74
**K1**	**-0.0125406**	4.7e-05				1.00	-0.97	0.91	0.02	0.01
**K2**	**0.0352767**	0.00024					1.00	-0.98	-0.01	-0.01
**K3**	**-0.0391368**	0.00037						1.00	0.01	0.02
**P1**	**0.00201997**	2.6e-06							1.00	-0.03
**P2**	**0.00125072**	2.1e-06								1.00

Table 7. Calibration coefficients and correlation matrix.

Ground Control Points

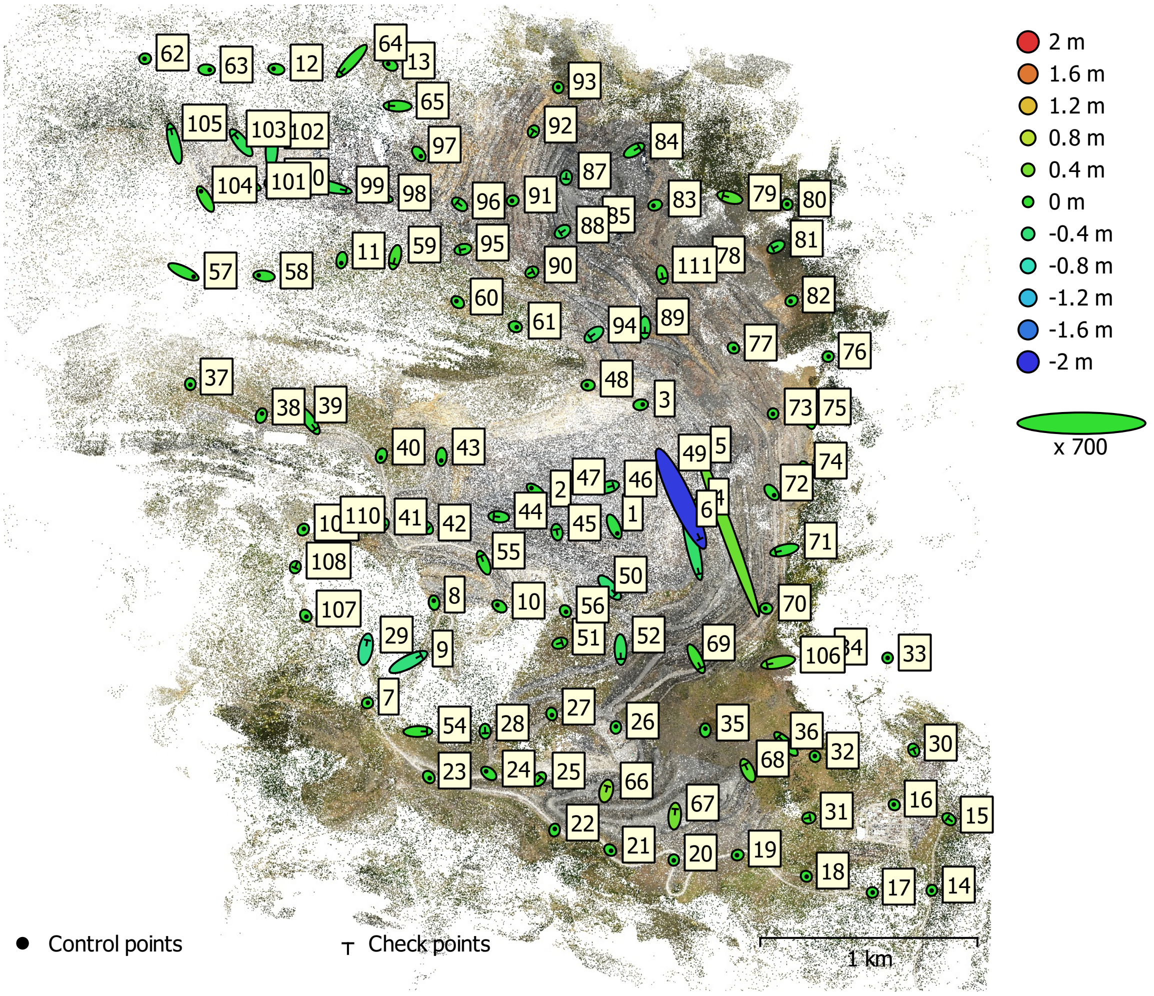

Fig. 8. GCP locations and error estimates.
Z error is represented by ellipse color. X,Y errors are represented by ellipse shape.
Estimated GCP locations are marked with a dot or crossing.

Count	X error (cm)	Y error (cm)	Z error (cm)	XY error (cm)	Total (cm)
55	2.80614	2.87393	2.72167	4.0167	4.85195

Table 8. Control points RMSE.
X - Easting, Y - Northing, Z - Altitude.

Count	X error (cm)	Y error (cm)	Z error (cm)	XY error (cm)	Total (cm)
54	10.1337	18.2778	32.3392	20.8991	38.5045

Table 9. Check points RMSE.
X - Easting, Y - Northing, Z - Altitude.

Label	X error (cm)	Y error (cm)	Z error (cm)	Total (cm)	Image (pix)
1	4.1236	-9.01941	-10.7503	14.6261	0.503 (104)
2	-5.93548	4.56413	-0.0457726	7.48755	0.485 (109)
3	2.65827	0.377634	-0.308716	2.70265	0.180 (51)
4	2.02176	9.22405	14.8465	17.5951	0.637 (50)
7	0.501077	0.261658	0.0280129	0.565975	0.085 (24)
8	-0.388368	2.57931	-0.138992	2.61209	0.350 (32)
10	-2.68748	1.68706	2.24488	3.88693	0.500 (42)
11	-0.649293	-3.87353	-0.715585	3.99223	0.263 (36)
12	-3.68286	0.556057	0.393698	3.74535	0.409 (26)
13	-2.76199	1.42261	0.455177	3.14	0.179 (20)
14	-0.180699	-0.376666	0.0492855	0.420664	0.073 (23)
16	-0.557352	0.431544	0.0952309	0.711295	0.090 (34)
17	0.443331	-0.0141205	-0.0564633	0.447135	0.071 (23)
18	0.459378	-0.445775	-0.0578671	0.642723	0.114 (25)
19	-0.910253	-0.240528	-0.0876634	0.945568	0.105 (20)
20	-0.0326274	-0.503946	-0.0246455	0.505602	0.124 (16)
21	1.17858	-0.947873	0.168426	1.5218	0.137 (15)
22	0.347731	1.4585	-0.0262054	1.49961	0.121 (13)
23	1.13055	-1.36686	0.134707	1.77893	0.134 (18)
24	-3.52331	2.65463	-0.514931	4.44139	0.331 (27)
26	0.0716841	1.32018	-0.556325	1.4344	0.128 (33)
27	0.373543	-1.41474	0.0646983	1.46466	0.199 (27)
32	-0.242279	0.38142	-0.014289	0.452089	0.069 (18)
33	0.0122861	0.00353754	-0.00668827	0.014429	0.002 (3)
34	-0.0115964	0.0703207	-0.0222295	0.0746568	0.014 (4)
35	0.0631794	2.07324	-0.439987	2.12036	0.259 (11)
37	0.0113253	-0.904668	0.28565	0.948762	0.120 (46)
38	0.899759	2.7332	-0.524011	2.92481	0.180 (57)
40	-0.858977	-2.64859	-1.30515	3.0751	0.273 (66)
43	-0.261915	-4.93825	0.289193	4.95364	0.490 (69)
48	-1.82677	0.0475625	-0.523648	1.90093	0.187 (44)

Label	X error (cm)	Y error (cm)	Z error (cm)	Total (cm)	Image (pix)
56	0.739305	-0.982904	-0.0594551	1.23134	0.309 (50)
57	13.3641	-6.38408	-1.5549	14.892	1.482 (14)
58	-7.06247	0.810913	-1.13914	7.19957	0.867 (30)
60	-1.69953	1.40301	0.152463	2.20909	0.160 (32)
61	1.65262	-0.615679	0.584762	1.858	0.135 (20)
62	-0.816152	-0.09953	0.219345	0.850954	0.147 (17)
63	3.96067	-0.211341	-0.277278	3.97598	0.310 (16)
70	-1.21935	0.152804	-0.844678	1.49119	0.101 (30)
72	3.27945	-3.7512	-3.23618	5.9413	0.270 (18)
73	-0.118933	-0.220183	0.204586	0.323236	0.064 (15)
76	0.598256	0.141012	-0.0192661	0.614951	0.095 (9)
77	0.784	-0.522848	0.0501635	0.943686	0.089 (11)
78	0.40615	-1.58726	-0.215848	1.65256	0.121 (11)
80	-0.0074238	0.333767	0.0272648	0.334961	0.092 (7)
82	-1.24407	-0.665084	-0.277328	1.43769	0.212 (6)
83	-2.1864	-0.653107	-0.173334	2.28843	0.204 (13)
85	0.374918	0.0602314	-0.340271	0.509878	0.206 (10)
91	-0.935761	-0.276732	-0.137101	0.985406	0.193 (20)
93	-0.0604171	0.610346	-0.0895177	0.619827	0.171 (16)
97	2.62384	-3.12629	0.253238	4.08929	0.362 (40)
100	2.00051	0.494675	-3.30287	3.89303	0.822 (28)
104	-5.87587	10.1928	5.5231	12.9971	1.070 (21)
107	0.839521	-1.1728	-0.457699	1.51319	0.102 (21)
109	0.817335	0.913995	2.16963	2.49213	0.264 (22)
**Total**	**2.80614**	**2.87393**	**2.72167**	**4.85195**	**0.396**

Table 10. Control points.

X - Easting, Y - Northing, Z - Altitude.

Label	X error (cm)	Y error (cm)	Z error (cm)	Total (cm)	Image (pix)
5	-38.413	105.317	33.3422	116.957	0.576 (38)
6	8.72542	-38.4118	-31.6374	50.5225	0.270 (66)
9	17.1101	8.67679	-42.9204	47.0128	0.245 (29)

Label	X error (cm)	Y error (cm)	Z error (cm)	Total (cm)	Image (pix)
15	-2.03168	1.45582	1.87527	3.1247	0.117 (27)
25	2.08082	2.4156	6.5471	7.28214	0.103 (22)
28	0.0899507	-2.57057	-7.27869	7.7198	0.163 (29)
29	2.27808	11.8959	-53.4212	54.777	0.029 (18)
30	-0.757266	1.67192	-0.152978	1.84178	0.104 (20)
31	1.55741	0.29718	8.13716	8.29019	0.135 (26)
36	-9.61585	9.75309	3.93114	14.2493	0.135 (14)
39	8.50128	-11.7352	-6.24954	15.7811	0.207 (40)
41	1.30704	1.92305	-10.065	10.3301	0.398 (56)
42	2.62342	-2.33979	-11.123	11.6653	0.373 (48)
44	-6.53145	0.642799	-5.6512	8.66078	0.604 (73)
45	-0.660894	3.58457	-8.94778	9.66172	0.557 (94)
46	6.52148	1.88891	-17.4853	18.7572	0.409 (63)
47	2.83172	5.691	-15.1491	16.4287	0.479 (107)
49	24.9098	-53.1415	-194.528	203.189	0.772 (59)
50	8.14115	-8.89508	-36.5821	38.5182	0.426 (56)
51	3.01356	0.944139	-2.18763	3.8417	0.351 (42)
52	0.322917	-12.3883	-24.4707	27.4297	0.309 (56)
54	11.9346	0.285161	1.52662	12.0353	0.119 (31)
55	-3.74356	8.92332	-1.05674	9.7343	0.343 (51)
59	-2.20581	-8.8315	10.0371	13.5501	0.258 (9)
64	-14.3291	-15.4808	3.6689	21.4112	0.289 (13)
65	-11.4532	0.358142	2.85579	11.8093	0.348 (31)
66	1.59517	6.22009	49.0822	49.5005	0.178 (22)
67	0.681768	9.34342	36.9895	38.1574	0.164 (12)
68	-3.8621	7.9737	17.508	19.6221	0.138 (16)
69	6.28438	-12.2023	7.66347	15.72	0.215 (28)
71	-11.7285	-2.88152	-4.49859	12.8879	0.226 (24)
74	2.83654	-6.19457	6.70876	9.5617	0.102 (10)
75	-2.13221	6.93228	2.52553	7.67992	0.025 (6)
79	-9.15199	2.48023	18.3282	20.6357	0.142 (8)
81	-4.34324	-2.23673	-9.7802	10.9325	0.238 (6)

Label	X error (cm)	Y error (cm)	Z error (cm)	Total (cm)	Image (pix)
84	6.83321	3.94881	-3.30392	8.5558	0.176 (12)
87	-0.138108	-1.84664	-19.47	19.5579	0.241 (10)
88	-2.90209	-1.8822	-21.9795	22.25	0.244 (13)
89	-0.0892739	-7.23419	-16.3154	17.8476	0.163 (27)
90	1.70975	0.876042	3.39237	3.89857	0.154 (28)
92	0.685951	1.0946	2.37954	2.70756	0.225 (18)
94	-5.16675	-3.71797	-27.6354	28.359	0.149 (35)
95	-4.13219	-0.861919	-2.16282	4.74296	0.251 (38)
96	-3.37997	2.61137	-4.41792	6.14504	0.197 (23)
98	16.5423	-3.64262	-1.30085	16.9885	0.576 (32)
99	30.2008	-6.78962	-14.1385	34.0307	0.574 (30)
101	-16.9507	7.80438	-2.44955	18.8211	0.669 (26)
102	0.86173	17.0477	-26.24	31.3034	0.364 (21)
103	-8.93727	11.1335	-20.4879	24.9717	0.478 (20)
105	-4.79108	19.1797	-16.8139	25.9523	0.199 (24)
106	-14.7088	-2.93439	20.0941	25.0745	0.087 (13)
108	-0.397947	-1.02435	3.8732	4.02609	0.179 (19)
110	-2.22619	3.24526	-6.86472	7.91277	0.263 (28)
111	1.21539	-5.21346	3.19844	6.23598	0.142 (14)
**Total**	**10.1337**	**18.2778**	**32.3392**	**38.5045**	**0.388**

Table 11. Check points.

X - Easting, Y - Northing, Z - Altitude.

Digital Elevation Model

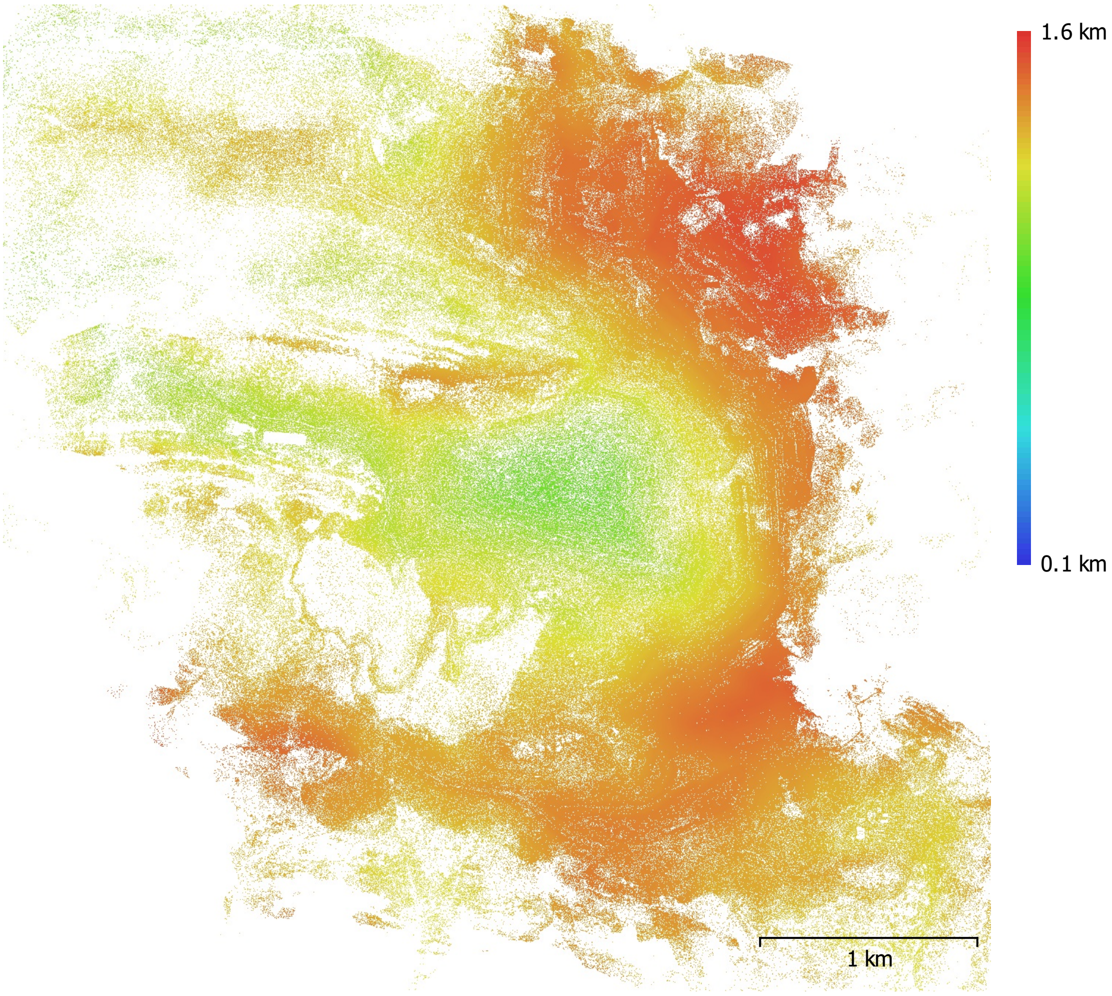

Fig. 9. Reconstructed digital elevation model.

Resolution:	unknown
Point density:	unknown

Processing Parameters

**General**	
Cameras	2595
Aligned cameras	2577
Markers	110
**Shapes**	
Polygon	1
Coordinate system	ETRS89 / UTM zone 30N (EPSG::25830)
Rotation angles	Yaw, Pitch, Roll
**Tie Points**	
Points	1,812,511 of 12,529,745
RMS reprojection error	0.13909 (0.330593 pix)
Max reprojection error	0.300168 (1.80171 pix)
Mean key point size	2.33442 pix
Point colors	3 bands, uint8
Key points	No
Average tie point multiplicity	3.65511
**Alignment parameters**	
Accuracy	High
Generic preselection	Yes
Reference preselection	No
Key point limit	60,000
Key point limit per Mpx	1,000
Tie point limit	0
Exclude stationary tie points	Yes
Guided image matching	No
Adaptive camera model fitting	No
Matching time	4 hours 7 minutes
Matching memory usage	3.73 GB
Alignment time	2 hours 17 minutes
Alignment memory usage	4.82 GB
**Optimization parameters**	
Parameters	f, cx, cy, k1-k3, p1, p2
Adaptive camera model fitting	No
Optimization time	4 minutes 36 seconds
Date created	2023:11:13 15:04:46
Software version	2.0.0.15597
File size	776.49 MB
**System**	
Software name	Agisoft Metashape Professional
Software version	2.0.3 build 16960
OS	Windows 64 bit
RAM	63.90 GB
CPU	Intel(R) Core(TM) i7-7700 CPU @ 3.60GHz
GPU(s)	Quadro M4000
