# Supplementary material for: Automated cleaning of tie point clouds following USGS guidelines in Agisoft Metashape professional (ver. 2.1.0)
Source: MethodsX. 2024 Mar 26;12:102679. doi: 10.1016/j.mex.2024.102679 (PMC10992719; doi:10.1016/j.mex.2024.102679)
Supplement: Supplementary file 3 — The supplementary material includes supplementary text, figures and the processing reports generated by the software. [file mmc3.zip › Lucia_SCC-Default_r4.pdf]

Lucia_SCC-Default_r4

Automatically cleaned sparse cloud using the SCC script (default settings). UAS data provided by Sanz-Ablanedo et al. (2018).

Sanz-Ablanedo, E., Chandler, J. H., Rodríguez-Pérez, J. R., and Ordóñez, C.: Accuracy of Unmanned Aerial Vehicle (UAV) and SfM Photogrammetry Survey as a Function of the Number and Location of Ground Control Points Used, Remote Sensing, 10, 1606, 2018.

28 December 2023

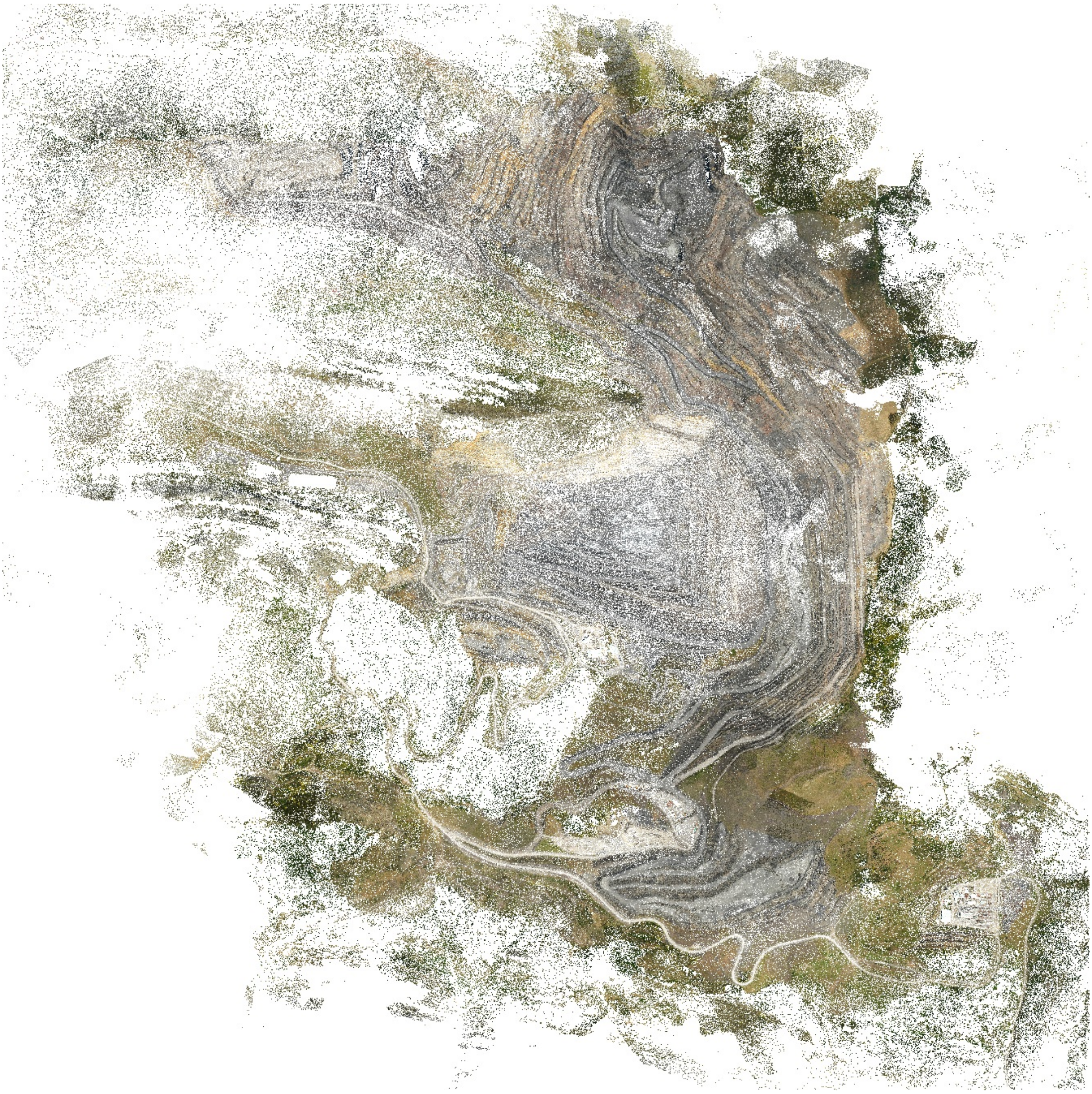

Survey Data

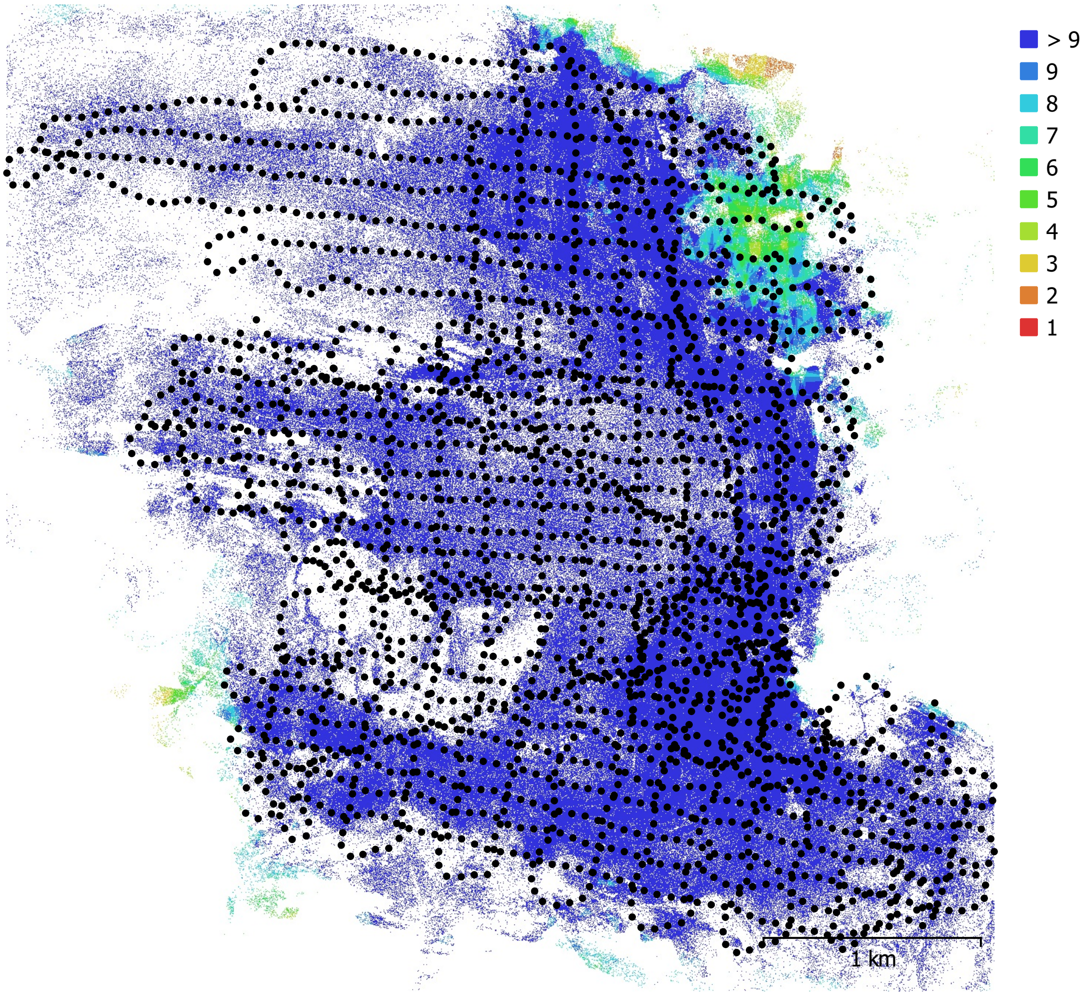

Fig. 1. Camera locations and image overlap.

Number of images:	2,595	Camera stations:	2,577
Flying altitude:	349 m	Tie points:	1,793,201
Ground resolution:	6.2 cm/pix	Projections:	4,236,413
Coverage area:	7.51 km²	Reprojection error:	0.328 pix

Camera Model	Resolution	Focal Length	Pixel Size	Precalibrated
NX500 (20 mm)	6480 x 4320	20 mm	3.7 x 3.7 μm	No
NX500 (20 mm)	6480 x 4320	20 mm	3.7 x 3.7 μm	No
NX500 (20 mm)	6480 x 4320	20 mm	3.7 x 3.7 μm	No
NX500 (20 mm)	6480 x 4320	20 mm	3.7 x 3.7 μm	No
NX500 (20 mm)	6480 x 4320	20 mm	3.7 x 3.7 μm	No

Camera Model	Resolution	Focal Length	Pixel Size	Precalibrated
NX500 (20 mm)	6480 x 4320	20 mm	3.7 x 3.7 μm	No

Table 1. Cameras.

Camera Calibration

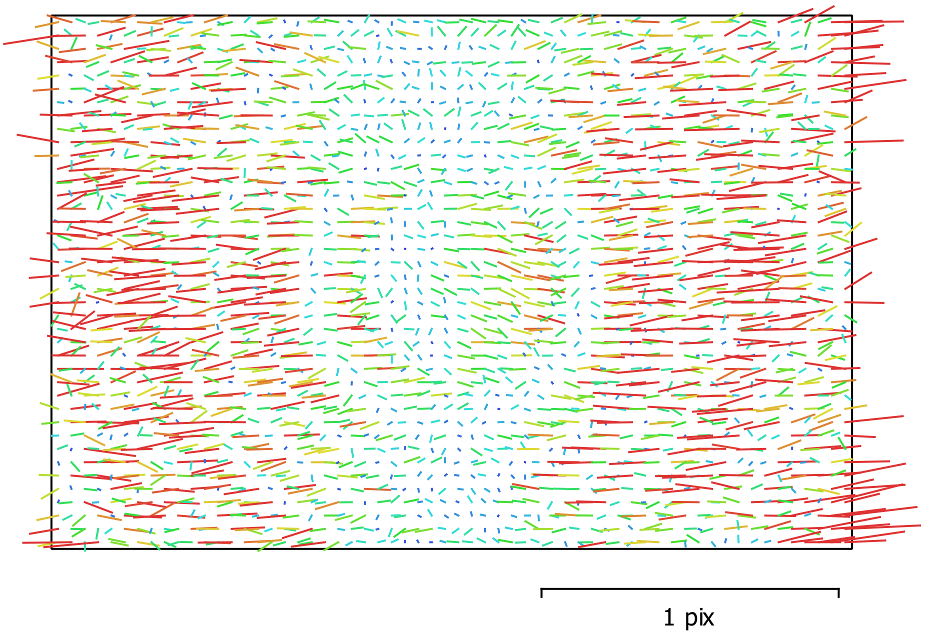

Fig. 2. Image residuals for NX500 (20 mm).

NX500 (20 mm)

200 images

Type	Resolution	Focal Length	Pixel Size
**Frame**	**6480 x 4320**	**20 mm**	**3.7 x 3.7 µm**

	Value	Error	F	Cx	Cy	K1	K2	K3	P1	P2
**F**	**5619.54**	0.051	1.00	0.02	-0.00	-0.38	0.32	-0.29	-0.01	0.08
**Cx**	**93.0206**	0.06		1.00	-0.03	0.03	-0.02	0.01	0.82	0.06
**Cy**	**37.2762**	0.069			1.00	-0.00	-0.00	-0.00	-0.02	0.78
**K1**	**-0.0119427**	6.3e-05				1.00	-0.96	0.90	0.05	0.00
**K2**	**0.0252285**	0.00031					1.00	-0.98	-0.05	-0.01
**K3**	**-0.0210794**	0.00046						1.00	0.05	0.01
**P1**	**0.0027531**	3.6e-06							1.00	0.04
**P2**	**0.000831181**	4.3e-06								1.00

Table 2. Calibration coefficients and correlation matrix.

Camera Calibration

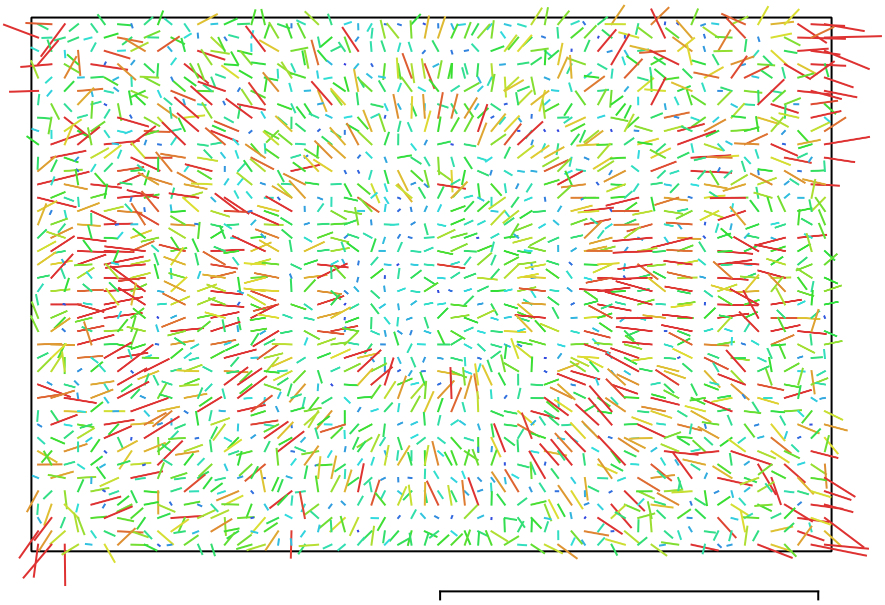

Fig. 3. Image residuals for NX500 (20 mm).

NX500 (20 mm)

462 images

Type	Resolution	Focal Length	Pixel Size
**Frame**	**6480 x 4320**	**20 mm**	**3.7 x 3.7 μm**

	Value	Error	F	Cx	Cy	K1	K2	K3	P1	P2
**F**	**5628.81**	0.042	1.00	-0.16	-0.14	-0.34	0.31	-0.28	-0.03	-0.03
**Cx**	**71.1535**	0.041		1.00	0.07	0.03	-0.03	0.03	0.88	0.02
**Cy**	**43.975**	0.035			1.00	-0.00	-0.02	0.02	0.06	0.79
**K1**	**-0.0115172**	4.7e-05				1.00	-0.97	0.91	0.03	0.01
**K2**	**0.0254331**	0.00023					1.00	-0.98	-0.04	-0.03
**K3**	**-0.0222704**	0.00035						1.00	0.04	0.03
**P1**	**0.00224904**	2.6e-06							1.00	0.03
**P2**	**0.00117005**	2e-06								1.00

Table 3. Calibration coefficients and correlation matrix.

Camera Calibration

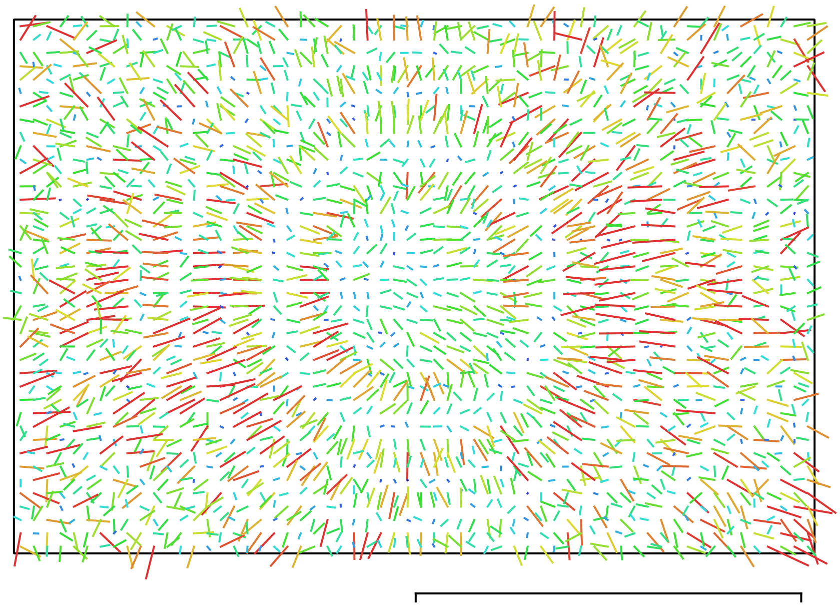

Fig. 4. Image residuals for NX500 (20 mm).

NX500 (20 mm)

530 images

Type	Resolution	Focal Length	Pixel Size
**Frame**	**6480 x 4320**	**20 mm**	**3.7 x 3.7 μm**

	Value	Error	F	Cx	Cy	K1	K2	K3	P1	P2
F	**5628.64**	0.044	1.00	-0.03	-0.13	-0.26	0.25	-0.22	-0.00	-0.02
Cx	**84.0261**	0.038		1.00	-0.02	0.01	-0.01	0.01	0.83	0.00
Cy	**35.1599**	0.029			1.00	0.01	-0.02	0.01	-0.01	0.68
K1	**-0.0120495**	4e-05				1.00	-0.96	0.91	0.02	0.01
K2	**0.0307728**	0.00021					1.00	-0.98	-0.02	-0.02
K3	**-0.0323838**	0.00032						1.00	0.03	0.02
P1	**0.00253638**	2.2e-06							1.00	0.02
P2	**0.000926536**	1.6e-06								1.00

Table 4. Calibration coefficients and correlation matrix.

Camera Calibration

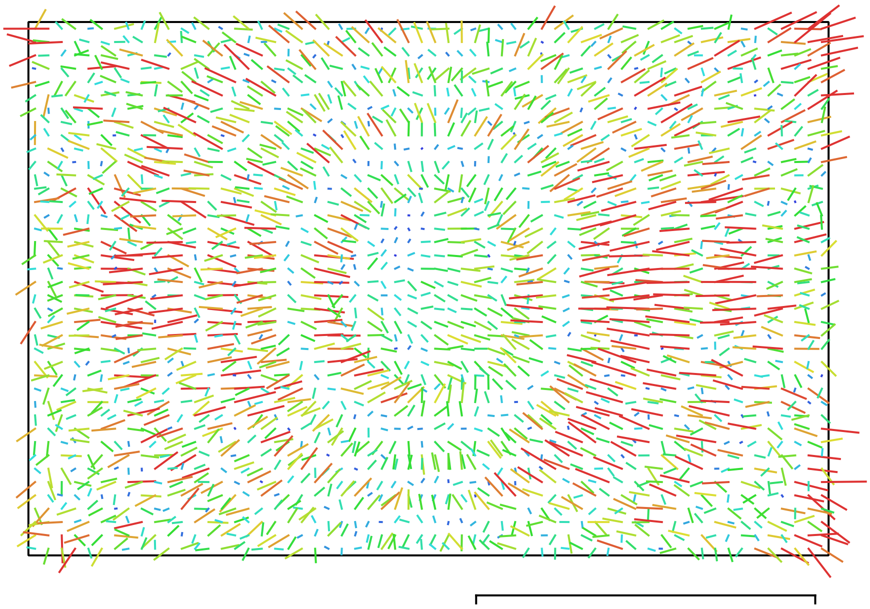

Fig. 5. Image residuals for NX500 (20 mm).

NX500 (20 mm)

513 images

Type	Resolution	Focal Length	Pixel Size
**Frame**	**6480 x 4320**	**20 mm**	**3.7 x 3.7 μm**

	Value	Error	F	Cx	Cy	K1	K2	K3	P1	P2
**F**	**5624.04**	0.053	1.00	-0.08	-0.08	-0.21	0.21	-0.19	0.00	-0.03
**Cx**	**83.9814**	0.034		1.00	-0.01	0.01	-0.01	0.02	0.80	-0.01
**Cy**	**60.21**	0.028			1.00	0.00	-0.01	0.01	-0.02	0.72
**K1**	**-0.0108241**	3.6e-05				1.00	-0.96	0.90	0.03	0.02
**K2**	**0.0228096**	0.00019					1.00	-0.98	-0.03	-0.02
**K3**	**-0.017545**	0.00029						1.00	0.04	0.02
**P1**	**0.00251328**	2.1e-06							1.00	-0.02
**P2**	**0.00150719**	1.7e-06								1.00

Table 5. Calibration coefficients and correlation matrix.

Camera Calibration

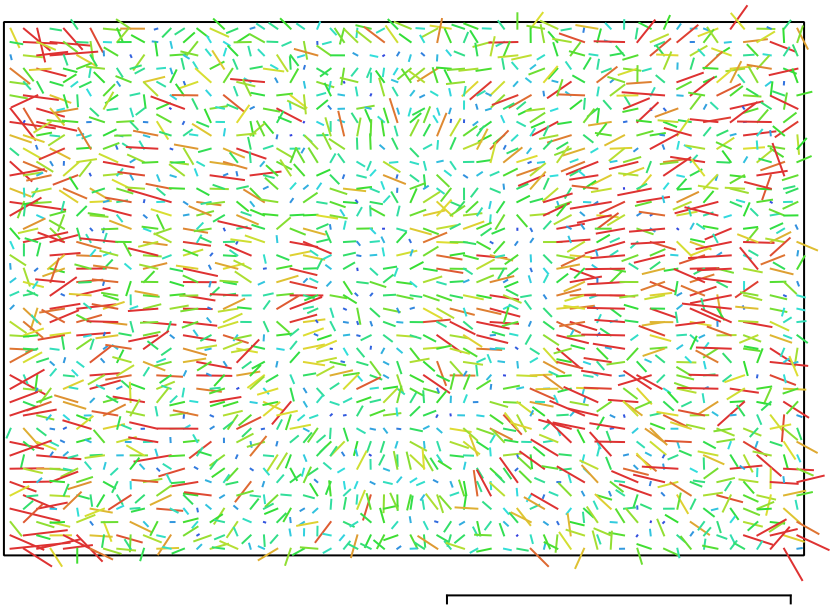

Fig. 6. Image residuals for NX500 (20 mm).

NX500 (20 mm)

412 images

Type	Resolution	Focal Length	Pixel Size
**Frame**	**6480 x 4320**	**20 mm**	**3.7 x 3.7 µm**

	Value	Error	F	Cx	Cy	K1	K2	K3	P1	P2
**F**	**5626.9**	0.046	1.00	0.04	-0.10	-0.37	0.34	-0.32	0.03	-0.01
**Cx**	**88.3983**	0.048		1.00	0.05	-0.01	0.01	0.00	0.88	0.03
**Cy**	**45.8036**	0.038			1.00	-0.03	0.03	-0.04	0.04	0.74
**K1**	**-0.01283**	5.4e-05				1.00	-0.97	0.91	0.00	-0.01
**K2**	**0.0327738**	0.00027					1.00	-0.98	-0.01	0.00
**K3**	**-0.0356751**	0.00041						1.00	0.02	-0.01
**P1**	**0.0025952**	3e-06							1.00	0.04
**P2**	**0.00115364**	2.2e-06								1.00

Table 6. Calibration coefficients and correlation matrix.

Camera Calibration

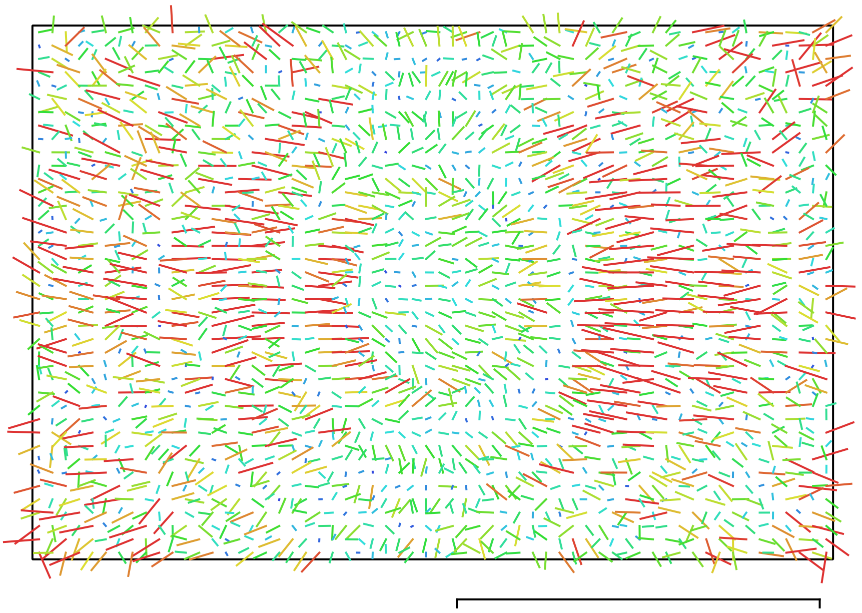

Fig. 7. Image residuals for NX500 (20 mm).

NX500 (20 mm)

478 images

Type	Resolution	Focal Length	Pixel Size
**Frame**	**6480 x 4320**	**20 mm**	**3.7 x 3.7 μm**

	Value	Error	F	Cx	Cy	K1	K2	K3	P1	P2
**F**	**5627.14**	0.032	1.00	0.01	0.00	-0.44	0.38	-0.34	-0.01	0.02
**Cx**	**68.8867**	0.04		1.00	-0.01	0.01	-0.01	0.01	0.87	-0.03
**Cy**	**47.9723**	0.036			1.00	0.02	-0.02	0.03	-0.02	0.74
**K1**	**-0.0124258**	4.7e-05				1.00	-0.97	0.91	0.02	0.01
**K2**	**0.0347767**	0.00024					1.00	-0.98	-0.01	-0.01
**K3**	**-0.03865**	0.00037						1.00	0.01	0.01
**P1**	**0.00201926**	2.6e-06							1.00	-0.03
**P2**	**0.0012388**	2.1e-06								1.00

Table 7. Calibration coefficients and correlation matrix.

Ground Control Points

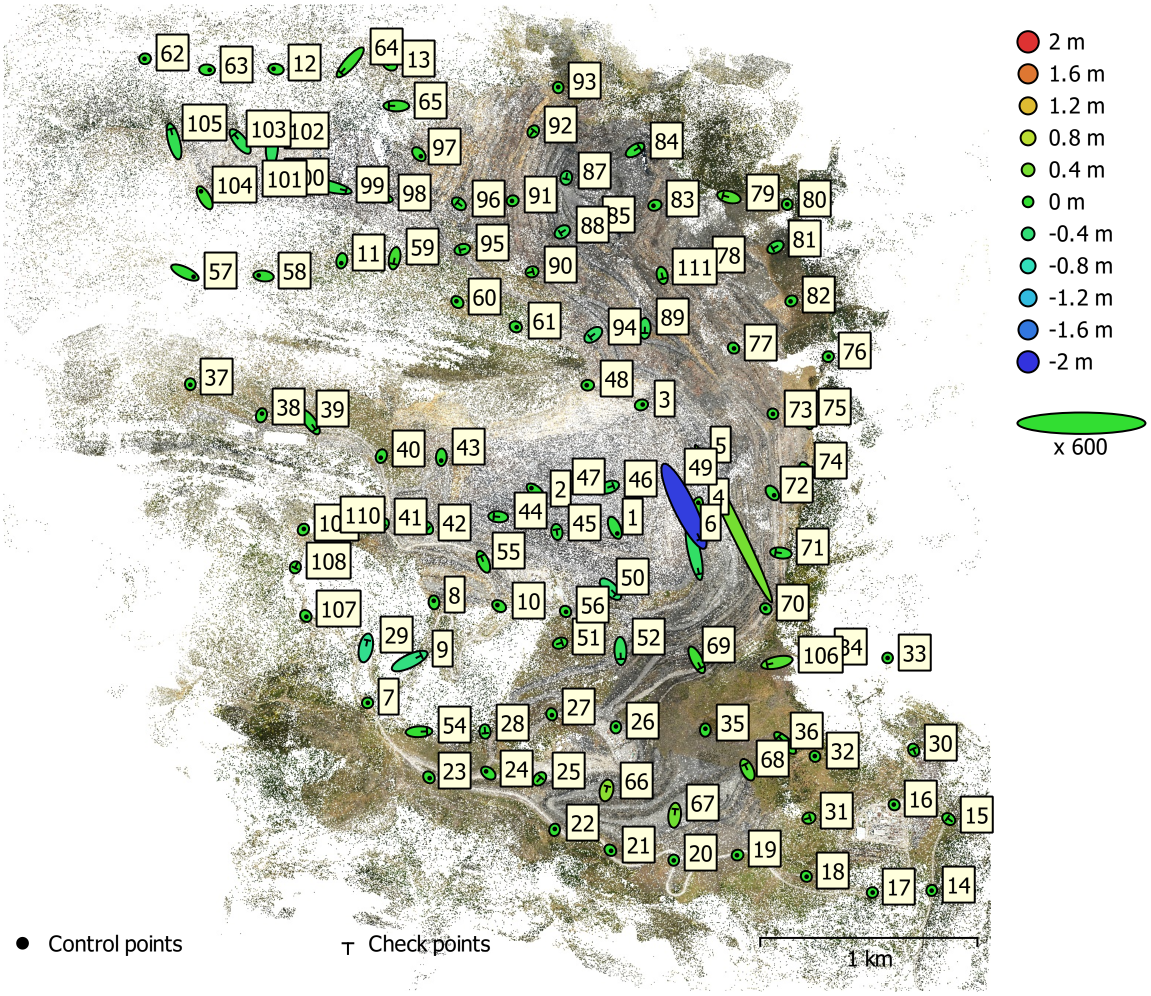

Fig. 8. GCP locations and error estimates.
Z error is represented by ellipse color. X,Y errors are represented by ellipse shape.
Estimated GCP locations are marked with a dot or crossing.

Count	X error (cm)	Y error (cm)	Z error (cm)	XY error (cm)	Total (cm)
55	2.77804	2.74946	2.55118	3.90858	4.6675

Table 8. Control points RMSE.
X - Easting, Y - Northing, Z - Altitude.

Count	X error (cm)	Y error (cm)	Z error (cm)	XY error (cm)	Total (cm)
54	11.4501	18.7323	32.5044	21.9546	39.2242

Table 9. Check points RMSE.
X - Easting, Y - Northing, Z - Altitude.

Label	X error (cm)	Y error (cm)	Z error (cm)	Total (cm)	Image (pix)
1	3.85579	-8.73471	-10.0878	13.8898	0.497 (104)
2	-5.93776	4.91177	-0.535181	7.72456	0.480 (109)
3	2.02077	0.56606	0.156957	2.10442	0.141 (51)
4	2.15442	7.78908	13.7789	15.974	0.612 (50)
7	0.410193	0.0845135	0.014519	0.41906	0.078 (24)
8	-0.285915	2.45738	-0.117809	2.47676	0.346 (32)
10	-2.59297	1.55596	2.30793	3.80408	0.491 (42)
11	-0.716248	-3.64368	-0.643614	3.76877	0.257 (36)
12	-3.63545	0.601921	0.372262	3.7037	0.398 (26)
13	-2.81488	1.35734	0.439726	3.15583	0.178 (20)
14	-0.174626	-0.380785	0.0591958	0.423078	0.073 (23)
16	-0.546182	0.451623	0.109623	0.717144	0.089 (34)
17	0.432122	0.007402	-0.0471225	0.434747	0.069 (23)
18	0.446785	-0.453557	-0.0522673	0.638797	0.113 (25)
19	-0.90298	-0.254063	-0.0750068	0.941035	0.103 (20)
20	-0.0221303	-0.428013	-0.0525788	0.431798	0.123 (16)
21	1.14722	-0.949013	0.184339	1.50024	0.130 (15)
22	0.327822	1.33463	-0.00326014	1.37431	0.116 (13)
23	1.07801	-1.25798	0.122555	1.66122	0.131 (18)
24	-3.3997	2.62293	-0.53376	4.32697	0.325 (27)
26	0.0602651	1.26392	-0.550149	1.37978	0.121 (33)
27	0.417761	-1.26193	0.0773464	1.33153	0.180 (27)
32	-0.243039	0.38161	-0.0164639	0.452731	0.068 (18)
33	0.0116587	0.0062943	-0.000100979	0.0132496	0.001 (3)
34	-0.0147969	0.0567708	-0.021551	0.0625006	0.012 (4)
35	0.129377	2.02519	-0.486557	2.08683	0.255 (11)
37	0.041317	-0.927637	0.247053	0.960861	0.120 (46)
38	0.847192	2.70969	-0.521329	2.88651	0.179 (57)
40	-0.90963	-2.36313	-1.36934	2.8787	0.269 (66)
43	-0.208024	-5.17716	0.270151	5.18837	0.486 (69)
48	-1.54714	-0.0286615	-0.494246	1.62442	0.180 (44)

Label	X error (cm)	Y error (cm)	Z error (cm)	Total (cm)	Image (pix)
56	0.716779	-0.696983	-0.0618388	1.00169	0.309 (50)
57	13.3425	-6.37914	-1.56875	14.872	1.477 (14)
58	-7.40322	0.940933	-0.949446	7.52293	0.848 (30)
60	-1.43598	1.36045	0.14422	1.98334	0.150 (32)
61	1.34709	-0.612861	0.543263	1.57651	0.123 (20)
62	-0.834705	-0.124485	0.193123	0.865752	0.145 (17)
63	3.9445	-0.21369	-0.279106	3.96013	0.305 (16)
70	-0.810282	0.470944	-0.654274	1.14299	0.092 (30)
72	2.82942	-3.48299	-2.97078	5.38167	0.256 (18)
73	0.184536	-0.156343	0.218352	0.325844	0.059 (15)
76	0.598485	0.152769	-0.0164491	0.617894	0.094 (9)
77	0.726122	-0.585276	0.0173894	0.932794	0.085 (11)
78	0.448108	-1.56712	-0.289109	1.65537	0.122 (11)
80	-0.00899533	0.325802	0.0314936	0.327444	0.091 (7)
82	-1.20734	-0.636561	-0.270809	1.39148	0.205 (6)
83	-2.15691	-0.635399	-0.14311	2.25311	0.201 (13)
85	0.395329	0.0591676	-0.354896	0.534544	0.202 (10)
91	-1.04313	-0.294292	-0.206788	1.1034	0.175 (20)
93	-0.0464385	0.654988	-0.098629	0.663998	0.163 (16)
97	2.96353	-3.20886	0.268046	4.3762	0.360 (40)
100	2.04007	0.695817	-3.10161	3.77704	0.817 (28)
104	-5.6692	9.82915	5.35208	12.5458	1.072 (21)
107	0.907541	-1.11836	-0.458861	1.51159	0.100 (21)
109	0.743047	0.898263	2.12391	2.4228	0.261 (22)
**Total**	**2.77804**	**2.74946**	**2.55118**	**4.6675**	**0.390**

Table 10. Control points.
X - Easting, Y - Northing, Z - Altitude.

Label	X error (cm)	Y error (cm)	Z error (cm)	Total (cm)	Image (pix)
5	-54.405	111.971	38.6768	130.358	0.473 (38)
6	7.49809	-34.3393	-29.6733	45.9991	0.237 (66)
9	18.0155	8.6025	-44.8985	49.137	0.242 (29)

Label	X error (cm)	Y error (cm)	Z error (cm)	Total (cm)	Image (pix)
15	-2.01856	1.45444	1.85133	3.10119	0.115 (27)
25	2.17506	2.35696	7.49278	8.15033	0.100 (22)
28	0.226772	-2.62867	-7.42133	7.87639	0.162 (29)
29	2.43831	11.8326	-53.5623	54.9079	0.028 (18)
30	-0.765729	1.75112	-0.0506007	1.91189	0.103 (20)
31	1.64434	0.484578	8.44534	8.61757	0.133 (26)
36	-9.76767	9.83702	3.47894	14.2926	0.135 (14)
39	8.24456	-11.4386	-6.01833	15.3308	0.202 (40)
41	1.37523	1.74377	-10.0112	10.2546	0.389 (56)
42	2.75185	-2.54587	-11.1201	11.7351	0.367 (48)
44	-6.7877	0.553546	-5.58626	8.80826	0.601 (73)
45	-0.638704	3.81182	-9.08307	9.87117	0.554 (94)
46	6.27819	2.26673	-17.6598	18.8791	0.392 (63)
47	3.00643	6.16278	-15.5916	17.0328	0.473 (107)
49	24.4643	-50.4427	-193.484	201.442	0.758 (59)
50	8.13773	-8.22901	-36.5724	38.3598	0.423 (56)
51	3.05209	1.03956	-2.55703	4.11513	0.345 (42)
52	0.515082	-12.3993	-25.2443	28.1298	0.308 (56)
54	12.2557	0.58418	1.74014	12.3924	0.118 (31)
55	-3.89112	8.87475	-1.27563	9.7739	0.337 (51)
59	-1.77706	-8.71983	9.32507	12.8899	0.245 (9)
64	-14.2677	-15.9779	6.12012	22.2782	0.288 (13)
65	-11.3773	0.12903	3.61411	11.9382	0.348 (31)
66	1.45036	6.37211	51.7351	52.1462	0.180 (22)
67	0.895245	9.58307	36.059	37.3214	0.159 (12)
68	-3.89528	8.09311	16.7995	19.0498	0.137 (16)
69	6.44766	-12.5663	6.77645	15.6654	0.211 (28)
71	-8.29957	1.08677	-4.29817	9.40947	0.213 (24)
74	3.42611	-6.58875	4.02368	8.4463	0.105 (10)
75	-1.29238	8.1573	-3.37989	8.92387	0.024 (6)
79	-9.25271	2.34674	18.4611	20.7829	0.142 (8)
81	-4.23829	-2.32288	-10.0771	11.1762	0.236 (6)

Label	X error (cm)	Y error (cm)	Z error (cm)	Total (cm)	Image (pix)
84	6.6157	4.18111	-1.86626	8.04563	0.168 (12)
87	-0.336571	-1.76103	-19.0829	19.1669	0.230 (10)
88	-3.07024	-1.89994	-22.0661	22.3595	0.229 (13)
89	-0.168823	-7.09935	-19.5219	20.7734	0.154 (27)
90	1.66197	0.443701	5.67633	5.93125	0.147 (28)
92	1.05557	1.37212	2.40215	2.96096	0.221 (18)
94	-5.0295	-3.82621	-29.6601	30.3258	0.141 (35)
95	-3.7974	-0.93881	-2.59037	4.69166	0.242 (38)
96	-2.82124	2.1438	-4.63688	5.83574	0.185 (23)
98	17.4246	-3.8532	-0.681248	17.8585	0.562 (32)
99	31.5636	-7.70039	-13.7685	35.2863	0.564 (30)
101	-16.7842	7.71884	-2.54897	18.6491	0.671 (26)
102	0.748437	17.0314	-26.5508	31.5527	0.361 (21)
103	-8.93854	11.0483	-20.8374	25.2222	0.476 (20)
105	-5.10979	18.9512	-17.7598	26.4701	0.200 (24)
106	-15.2115	-3.28821	20.2546	25.5431	0.084 (13)
108	-0.460556	-0.964976	3.92113	4.0643	0.178 (19)
110	-2.3349	3.23779	-7.23474	8.26296	0.261 (28)
111	1.27171	-5.05539	1.16808	5.34216	0.139 (14)
**Total**	**11.4501**	**18.7323**	**32.5044**	**39.2242**	**0.379**

Table 11. Check points.

X - Easting, Y - Northing, Z - Altitude.

Digital Elevation Model

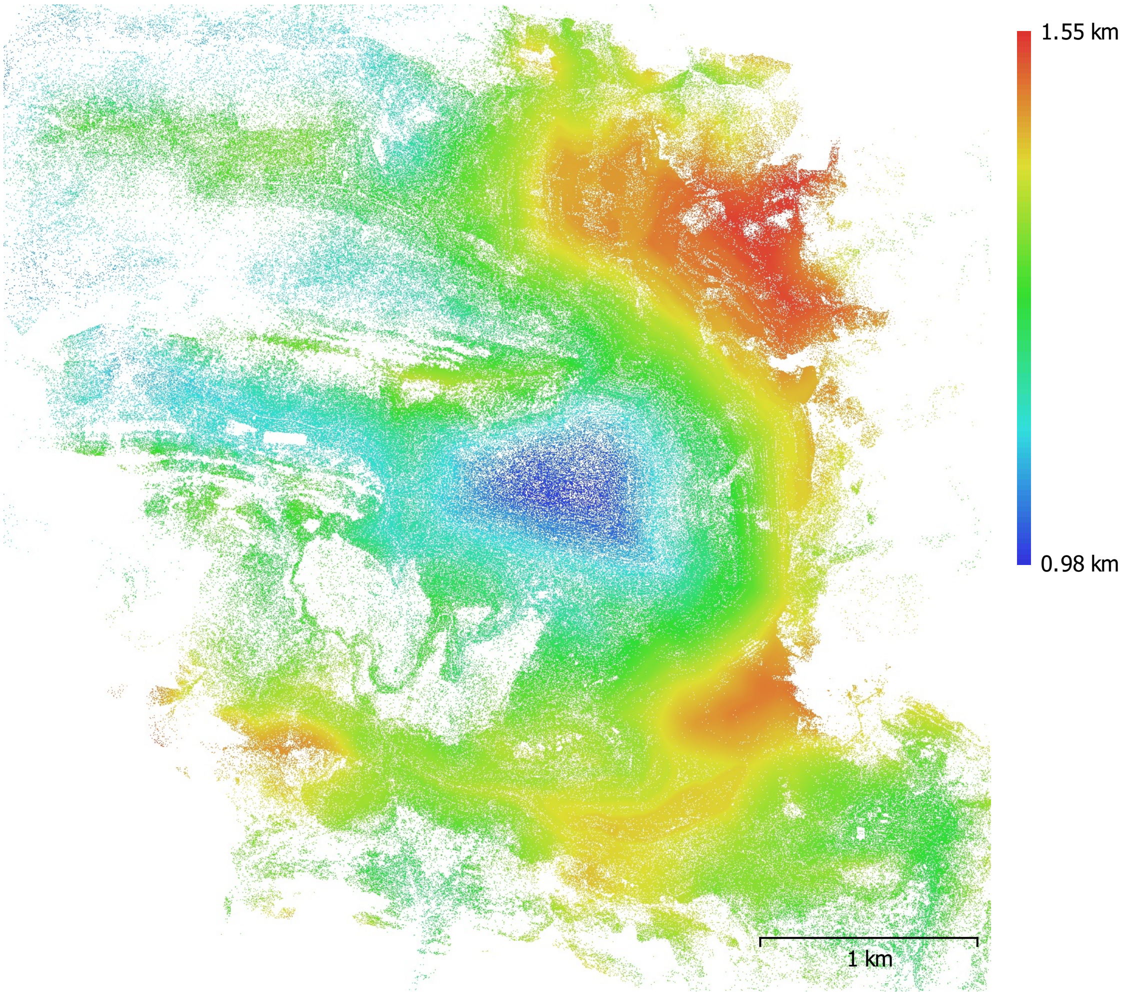

Fig. 9. Reconstructed digital elevation model.

Resolution:	unknown
Point density:	unknown

Processing Parameters

**General**	
Cameras	2595
Aligned cameras	2577
Markers	110
**Shapes**	
Polygon	1
Coordinate system	ETRS89 / UTM zone 30N (EPSG::25830)
Rotation angles	Yaw, Pitch, Roll
**Tie Points**	
Points	1,793,201 of 12,529,745
RMS reprojection error	0.138122 (0.328243 pix)
Max reprojection error	0.299804 (1.64331 pix)
Mean key point size	2.33405 pix
Point colors	3 bands, uint8
Key points	No
Average tie point multiplicity	3.65511
**Alignment parameters**	
Accuracy	High
Generic preselection	Yes
Reference preselection	No
Key point limit	60,000
Key point limit per Mpx	1,000
Tie point limit	0
Exclude stationary tie points	Yes
Guided image matching	No
Adaptive camera model fitting	No
Matching time	4 hours 7 minutes
Matching memory usage	3.73 GB
Alignment time	2 hours 17 minutes
Alignment memory usage	4.82 GB
**Optimization parameters**	
Parameters	f, cx, cy, k1-k3, p1, p2
Adaptive camera model fitting	No
Optimization time	27 seconds
Date created	2023:11:13 15:04:46
Software version	2.0.0.15597
File size	776.05 MB
**System**	
Software name	Agisoft Metashape Professional
Software version	2.0.3 build 16960
OS	Windows 64 bit
RAM	63.90 GB
CPU	Intel(R) Core(TM) i7-7700 CPU @ 3.60GHz
GPU(s)	Quadro M4000
