# Supplementary material for: Automated cleaning of tie point clouds following USGS guidelines in Agisoft Metashape professional (ver. 2.1.0)
Source: MethodsX. 2024 Mar 26;12:102679. doi: 10.1016/j.mex.2024.102679 (PMC10992719; doi:10.1016/j.mex.2024.102679)
Supplement: Supplementary file 3 — The supplementary material includes supplementary text, figures and the processing reports generated by the software. [file mmc3.zip › Lucia_SCC-Default_r5.pdf]

Lucia_SCC-Default_r5

Automatically cleaned sparse cloud using the SCC script (default settings). UAS data provided by Sanz-Ablanedo et al. (2018).

Sanz-Ablanedo, E., Chandler, J. H., Rodríguez-Pérez, J. R., and Ordóñez, C.: Accuracy of Unmanned Aerial Vehicle (UAV) and SfM Photogrammetry Survey as a Function of the Number and Location of Ground Control Points Used, Remote Sensing, 10, 1606, 2018.

28 December 2023

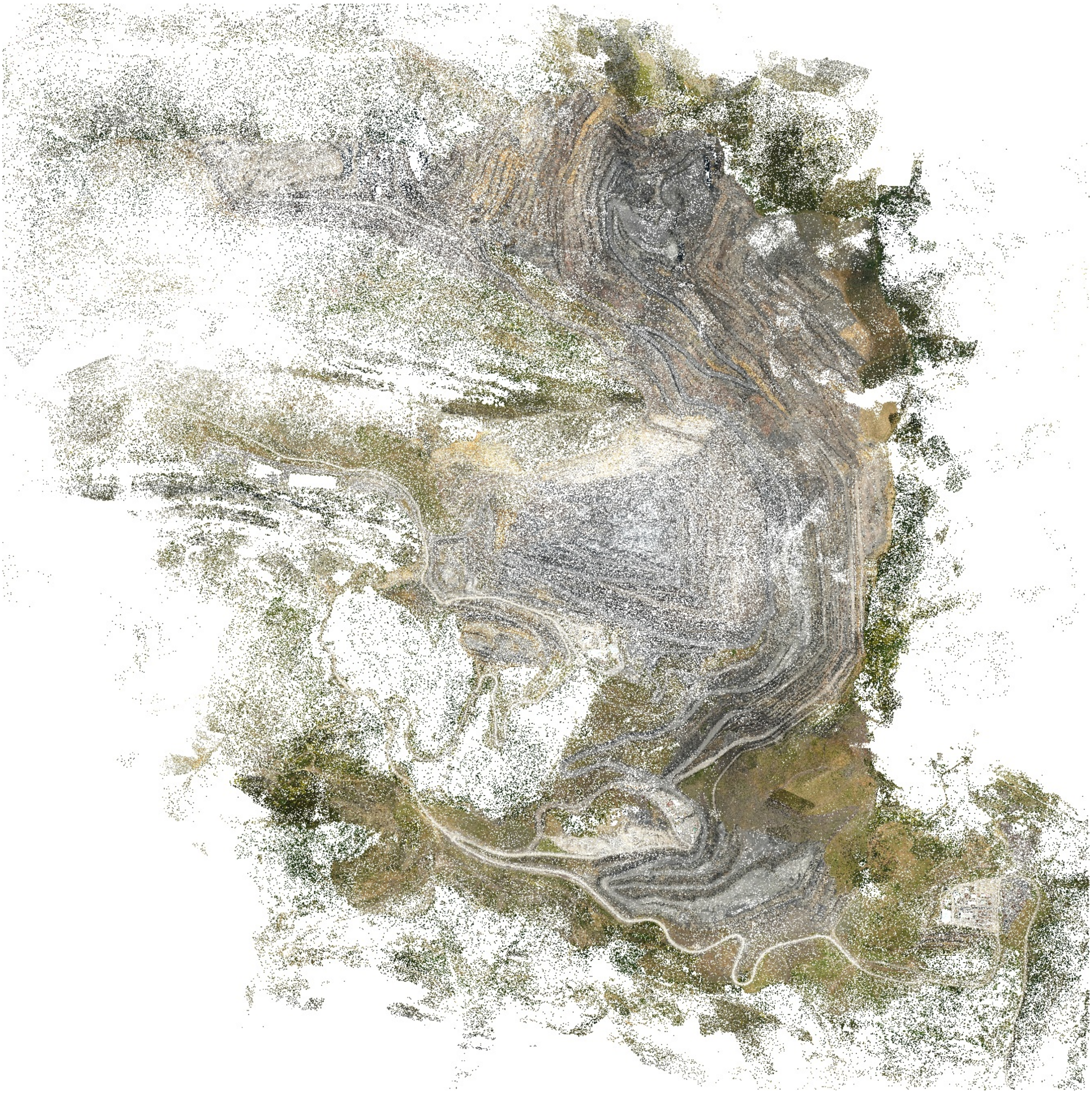

Survey Data

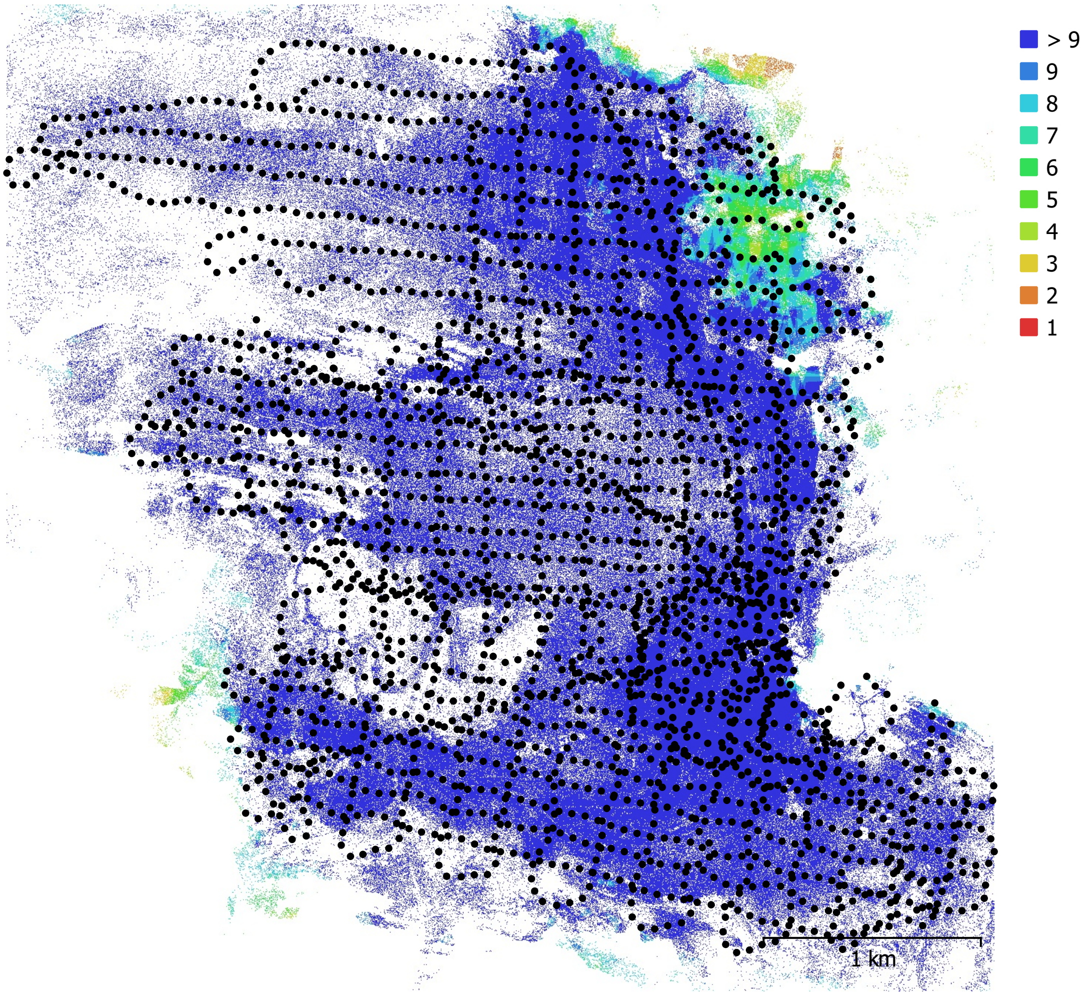

Fig. 1. Camera locations and image overlap.

Number of images:	2,595	Camera stations:	2,577
Flying altitude:	349 m	Tie points:	1,797,730
Ground resolution:	6.2 cm/pix	Projections:	4,249,639
Coverage area:	7.51 km²	Reprojection error:	0.329 pix

Camera Model	Resolution	Focal Length	Pixel Size	Precalibrated
NX500 (20 mm)	6480 x 4320	20 mm	3.7 x 3.7 μm	No
NX500 (20 mm)	6480 x 4320	20 mm	3.7 x 3.7 μm	No
NX500 (20 mm)	6480 x 4320	20 mm	3.7 x 3.7 μm	No
NX500 (20 mm)	6480 x 4320	20 mm	3.7 x 3.7 μm	No
NX500 (20 mm)	6480 x 4320	20 mm	3.7 x 3.7 μm	No

Camera Model	Resolution	Focal Length	Pixel Size	Precalibrated
NX500 (20 mm)	6480 x 4320	20 mm	3.7 x 3.7 μm	No

Table 1. Cameras.

Camera Calibration

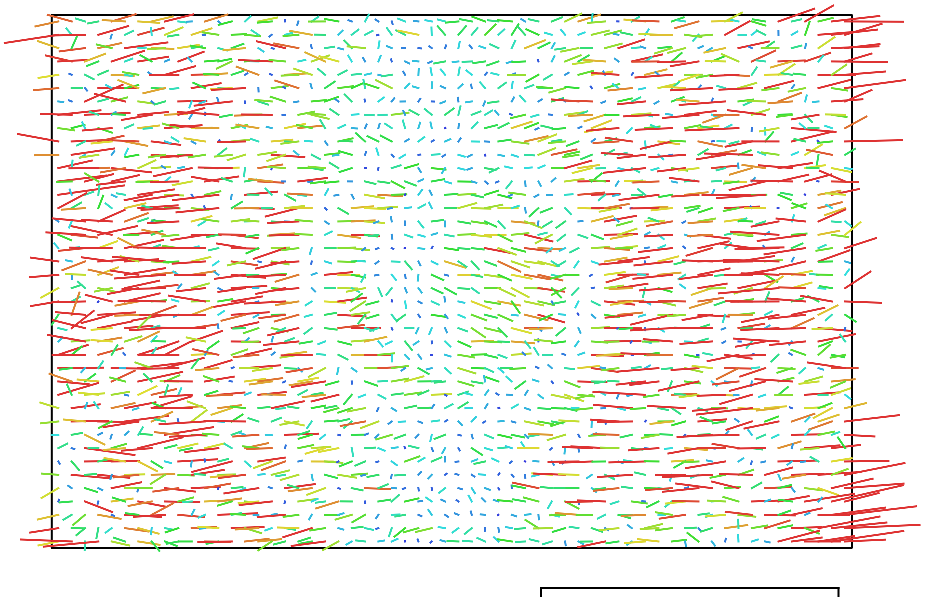

Fig. 2. Image residuals for NX500 (20 mm).

NX500 (20 mm)

200 images

Type	Resolution	Focal Length	Pixel Size
**Frame**	**6480 x 4320**	**20 mm**	**3.7 x 3.7 µm**

	Value	Error	F	Cx	Cy	K1	K2	K3	P1	P2
**F**	**5619.69**	0.05	1.00	0.02	0.00	-0.38	0.33	-0.29	-0.01	0.08
**Cx**	**93.0205**	0.06		1.00	-0.03	0.03	-0.02	0.01	0.82	0.06
**Cy**	**37.1704**	0.069			1.00	-0.00	-0.00	-0.00	-0.02	0.78
**K1**	**-0.0119445**	6.3e-05				1.00	-0.96	0.90	0.05	0.00
**K2**	**0.025263**	0.00031					1.00	-0.98	-0.05	-0.01
**K3**	**-0.0211274**	0.00046						1.00	0.05	0.01
**P1**	**0.00275189**	3.6e-06							1.00	0.04
**P2**	**0.00083253**	4.3e-06								1.00

Table 2. Calibration coefficients and correlation matrix.

Camera Calibration

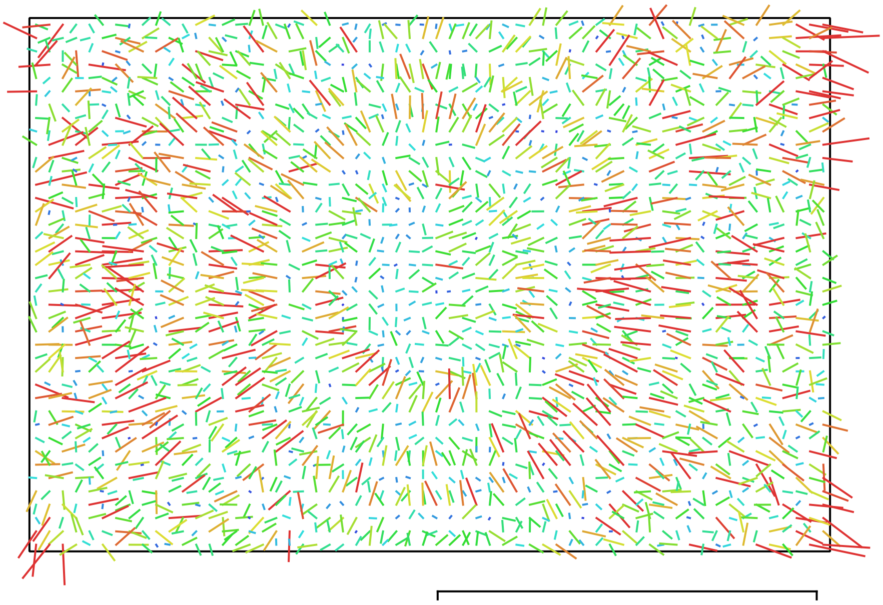

Fig. 3. Image residuals for NX500 (20 mm).

NX500 (20 mm)

462 images

Type	Resolution	Focal Length	Pixel Size
**Frame**	**6480 x 4320**	**20 mm**	**3.7 x 3.7 µm**

	Value	Error	F	Cx	Cy	K1	K2	K3	P1	P2
**F**	**5628.92**	0.041	1.00	-0.16	-0.13	-0.34	0.31	-0.28	-0.03	-0.03
**Cx**	**71.1604**	0.041		1.00	0.07	0.03	-0.03	0.03	0.88	0.02
**Cy**	**43.9633**	0.035			1.00	-0.00	-0.02	0.02	0.06	0.79
**K1**	**-0.0115588**	4.7e-05				1.00	-0.97	0.91	0.03	0.01
**K2**	**0.0257279**	0.00023					1.00	-0.98	-0.04	-0.03
**K3**	**-0.0227214**	0.00035						1.00	0.04	0.03
**P1**	**0.00224822**	2.6e-06							1.00	0.03
**P2**	**0.00117016**	2e-06								1.00

Table 3. Calibration coefficients and correlation matrix.

Camera Calibration

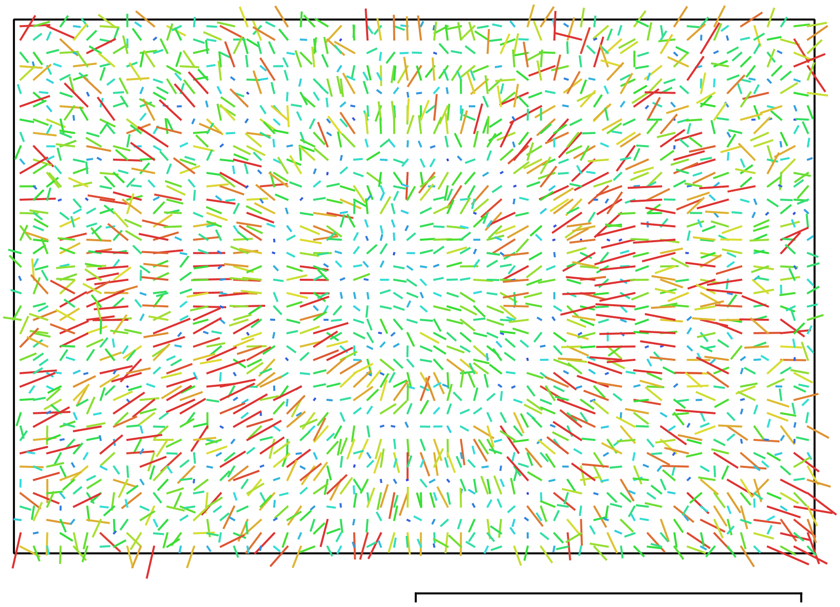

Fig. 4. Image residuals for NX500 (20 mm).

NX500 (20 mm)

530 images

Type	Resolution	Focal Length	Pixel Size
**Frame**	**6480 x 4320**	**20 mm**	**3.7 x 3.7 µm**

	**Value**	**Error**	**F**	**Cx**	**Cy**	**K1**	**K2**	**K3**	**P1**	**P2**
**F**	**5628.65**	0.044	1.00	-0.03	-0.13	-0.26	0.25	-0.22	-0.00	-0.02
**Cx**	**84.0377**	0.038		1.00	-0.02	0.01	-0.01	0.01	0.83	0.00
**Cy**	**35.1515**	0.029			1.00	0.01	-0.02	0.01	-0.01	0.68
**K1**	**-0.0120485**	4e-05				1.00	-0.96	0.91	0.02	0.01
**K2**	**0.0307658**	0.00021					1.00	-0.98	-0.02	-0.02
**K3**	**-0.032354**	0.00032						1.00	0.03	0.02
**P1**	**0.0025365**	2.2e-06							1.00	0.02
**P2**	**0.000926072**	1.6e-06								1.00

Table 4. Calibration coefficients and correlation matrix.

Camera Calibration

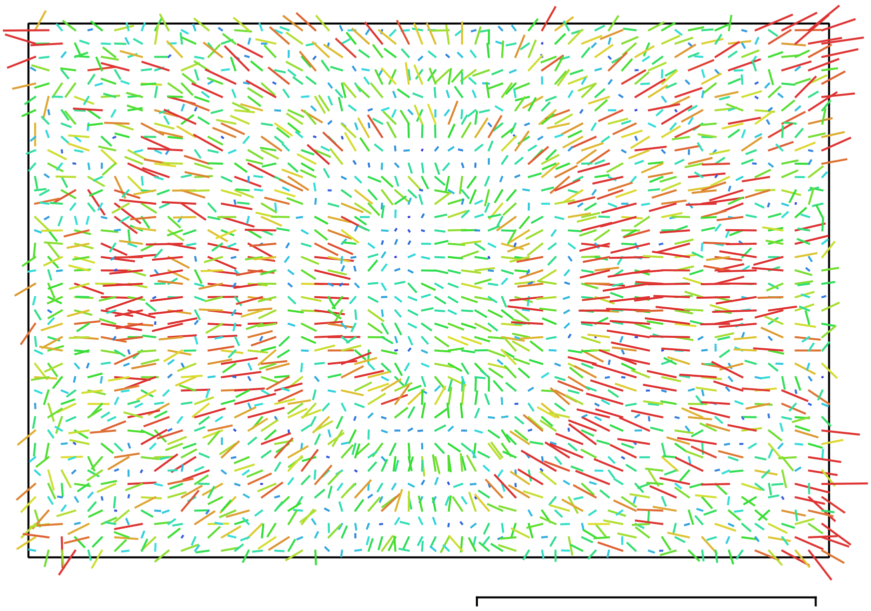

Fig. 5. Image residuals for NX500 (20 mm).

NX500 (20 mm)

513 images

Type	Resolution	Focal Length	Pixel Size
**Frame**	**6480 x 4320**	**20 mm**	**3.7 x 3.7 µm**

	Value	Error	F	Cx	Cy	K1	K2	K3	P1	P2
**F**	**5624.18**	0.052	1.00	-0.08	-0.08	-0.21	0.21	-0.19	0.00	-0.03
**Cx**	**83.9912**	0.034		1.00	-0.01	0.01	-0.01	0.02	0.80	-0.01
**Cy**	**60.1507**	0.028			1.00	0.00	-0.01	0.01	-0.02	0.72
**K1**	**-0.010838**	3.6e-05				1.00	-0.96	0.90	0.03	0.02
**K2**	**0.0228721**	0.00019					1.00	-0.98	-0.03	-0.02
**K3**	**-0.017615**	0.00029						1.00	0.04	0.02
**P1**	**0.00251282**	2.1e-06							1.00	-0.02
**P2**	**0.00150787**	1.7e-06								1.00

Table 5. Calibration coefficients and correlation matrix.

Camera Calibration

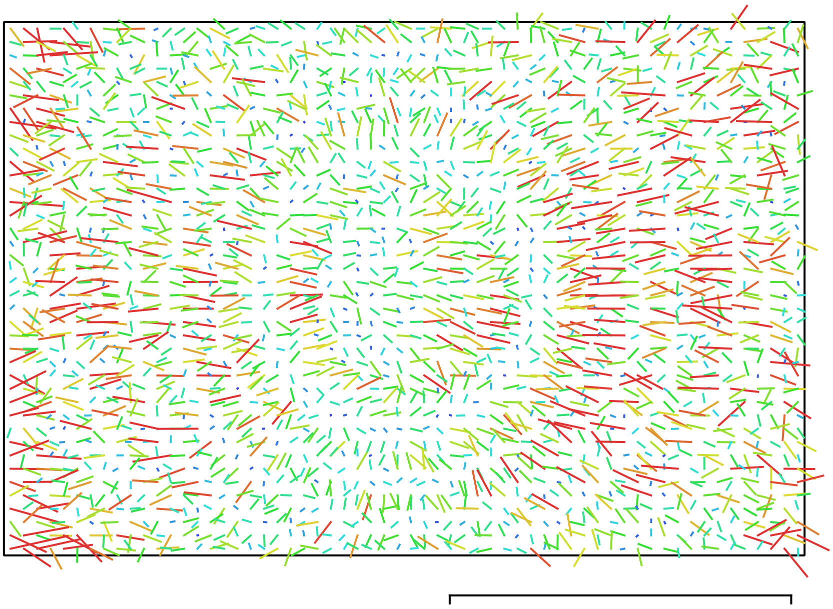

Fig. 6. Image residuals for NX500 (20 mm).

NX500 (20 mm)

412 images

Type	Resolution	Focal Length	Pixel Size
**Frame**	**6480 x 4320**	**20 mm**	**3.7 x 3.7 µm**

	Value	Error	F	Cx	Cy	K1	K2	K3	P1	P2
**F**	**5626.9**	0.046	1.00	0.04	-0.09	-0.37	0.34	-0.32	0.03	-0.01
**Cx**	**88.3845**	0.048		1.00	0.05	-0.01	0.01	-0.00	0.88	0.03
**Cy**	**45.7815**	0.038			1.00	-0.02	0.03	-0.04	0.04	0.74
**K1**	**-0.0127974**	5.3e-05				1.00	-0.97	0.91	0.00	-0.01
**K2**	**0.0325567**	0.00027					1.00	-0.98	-0.01	0.01
**K3**	**-0.0353261**	0.0004						1.00	0.02	-0.01
**P1**	**0.00259312**	3e-06							1.00	0.04
**P2**	**0.0011534**	2.2e-06								1.00

Table 6. Calibration coefficients and correlation matrix.

Camera Calibration

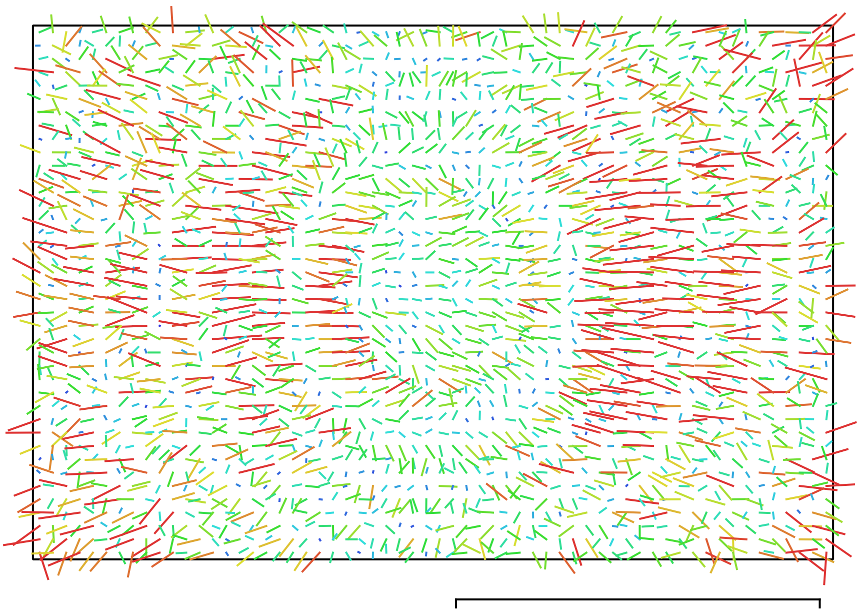

Fig. 7. Image residuals for NX500 (20 mm).

NX500 (20 mm)

478 images

Type	Resolution	Focal Length	Pixel Size
**Frame**	**6480 x 4320**	**20 mm**	**3.7 x 3.7 μm**

	Value	Error	F	Cx	Cy	K1	K2	K3	P1	P2
**F**	**5627.22**	0.032	1.00	0.00	-0.00	-0.44	0.38	-0.34	-0.01	0.02
**Cx**	**68.9471**	0.04		1.00	-0.01	0.02	-0.01	0.01	0.87	-0.03
**Cy**	**48.0259**	0.036			1.00	0.02	-0.03	0.03	-0.02	0.74
**K1**	**-0.01248**	4.7e-05				1.00	-0.97	0.91	0.02	0.01
**K2**	**0.0350133**	0.00024					1.00	-0.98	-0.01	-0.01
**K3**	**-0.0388208**	0.00037						1.00	0.01	0.02
**P1**	**0.00202042**	2.6e-06							1.00	-0.03
**P2**	**0.00124025**	2.1e-06								1.00

Table 7. Calibration coefficients and correlation matrix.

Ground Control Points

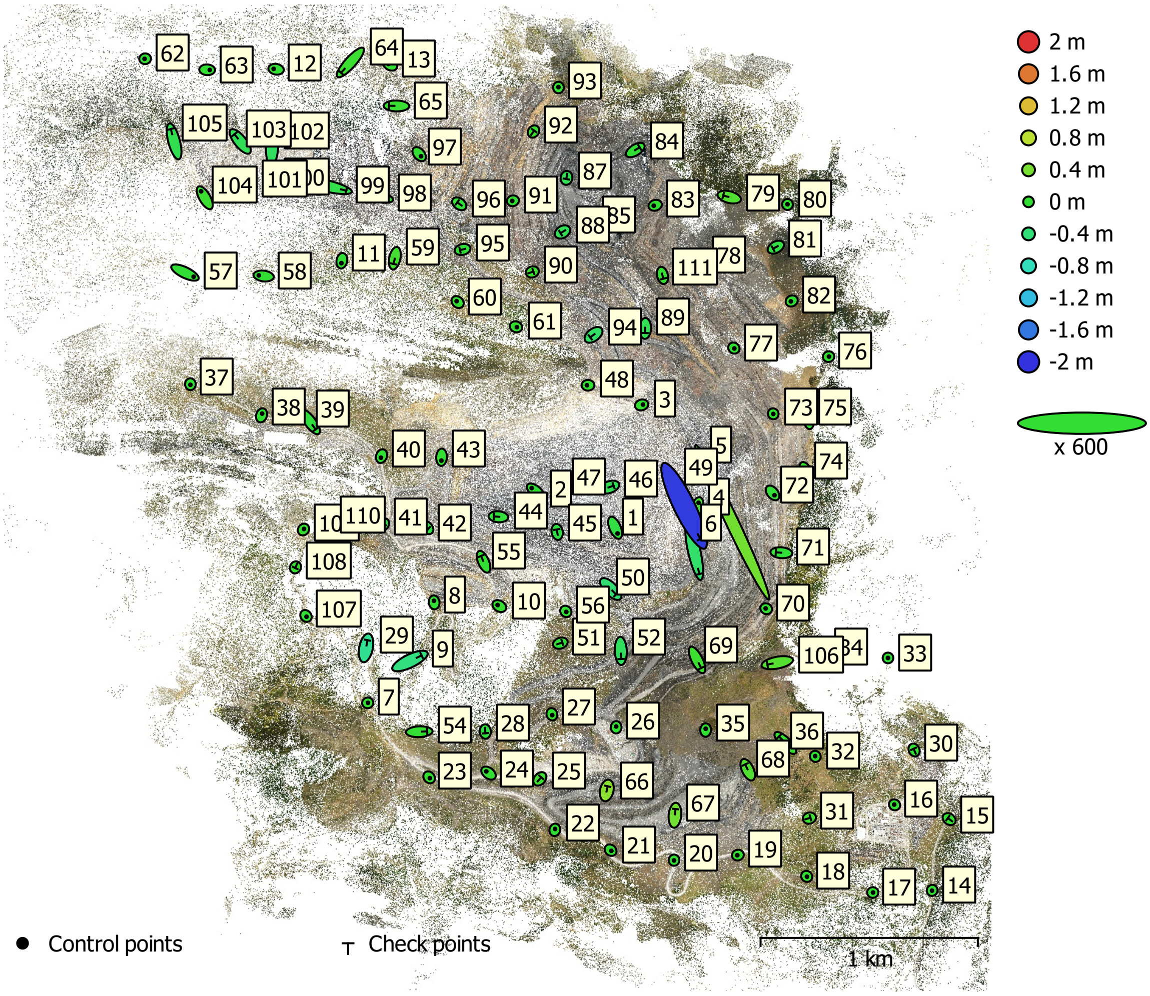

Fig. 8. GCP locations and error estimates.
Z error is represented by ellipse color. X,Y errors are represented by ellipse shape.
Estimated GCP locations are marked with a dot or crossing.

Count	X error (cm)	Y error (cm)	Z error (cm)	XY error (cm)	Total (cm)
55	2.7826	2.76526	2.58547	3.92295	4.69832

Table 8. Control points RMSE.
X - Easting, Y - Northing, Z - Altitude.

Count	X error (cm)	Y error (cm)	Z error (cm)	XY error (cm)	Total (cm)
54	11.2113	18.5581	32.6152	21.6817	39.1643

Table 9. Check points RMSE.
X - Easting, Y - Northing, Z - Altitude.

Label	X error (cm)	Y error (cm)	Z error (cm)	Total (cm)	Image (pix)
1	3.78866	-8.76907	-10.1916	13.9685	0.499 (104)
2	-5.91027	4.80955	-0.444328	7.63286	0.482 (109)
3	2.09479	0.595399	0.116341	2.18086	0.142 (51)
4	2.29548	7.98671	14.0399	16.3149	0.607 (50)
7	0.416219	0.092894	0.0115514	0.426616	0.078 (24)
8	-0.3073	2.46835	-0.111346	2.4899	0.347 (32)
10	-2.60411	1.56688	2.28429	3.80191	0.494 (42)
11	-0.666076	-3.69656	-0.663427	3.81423	0.259 (36)
12	-3.65673	0.603388	0.374409	3.72504	0.400 (26)
13	-2.78941	1.39113	0.446165	3.14883	0.178 (20)
14	-0.179536	-0.381523	0.0584432	0.425686	0.073 (23)
16	-0.550115	0.455018	0.109426	0.722248	0.089 (34)
17	0.437663	-0.000256625	-0.0469606	0.440175	0.069 (23)
18	0.448662	-0.449421	-0.0519567	0.637163	0.114 (25)
19	-0.903185	-0.250065	-0.0774148	0.940355	0.104 (20)
20	-0.0218026	-0.4344	-0.0533766	0.43821	0.123 (16)
21	1.15418	-0.960329	0.186492	1.51299	0.131 (15)
22	0.324846	1.36421	-0.0019595	1.40235	0.117 (13)
23	1.10061	-1.26464	0.122203	1.68095	0.132 (18)
24	-3.42984	2.62381	-0.536126	4.35151	0.327 (27)
26	0.0337295	1.27918	-0.560527	1.39701	0.124 (33)
27	0.427594	-1.29439	0.0738973	1.36519	0.183 (27)
32	-0.226818	0.396883	-0.0144288	0.457352	0.068 (18)
33	0.0115804	0.00615273	-0.000368017	0.0131186	0.001 (3)
34	-0.0126197	0.0624268	-0.0167464	0.0658544	0.013 (4)
35	0.0941131	2.07347	-0.484385	2.13138	0.257 (11)
37	0.0318019	-0.900236	0.262505	0.938268	0.119 (46)
38	0.882542	2.68675	-0.540106	2.87911	0.179 (57)
40	-0.892317	-2.4045	-1.37164	2.90848	0.269 (66)
43	-0.230644	-5.13752	0.258173	5.14917	0.486 (69)
48	-1.60113	0.00272092	-0.499378	1.6772	0.181 (44)

Label	X error (cm)	Y error (cm)	Z error (cm)	Total (cm)	Image (pix)
56	0.686939	-0.760389	-0.0954373	1.02917	0.308 (50)
57	13.3637	-6.38949	-1.56647	14.8952	1.479 (14)
58	-7.40254	0.955845	-0.94009	7.52296	0.850 (30)
60	-1.51754	1.36174	0.14772	2.04428	0.153 (32)
61	1.42898	-0.583253	0.543492	1.63633	0.126 (20)
62	-0.827572	-0.124699	0.195691	0.859489	0.145 (17)
63	3.94945	-0.208728	-0.284855	3.96521	0.306 (16)
70	-0.821547	0.420332	-0.666688	1.13846	0.094 (30)
72	2.92794	-3.54686	-3.04598	5.51643	0.260 (18)
73	0.112365	-0.224394	0.182095	0.31006	0.060 (15)
76	0.598904	0.155723	-0.0194495	0.619123	0.095 (9)
77	0.680775	-0.576368	0.00729593	0.892025	0.086 (11)
78	0.436929	-1.57634	-0.281789	1.65986	0.122 (11)
80	-0.00417001	0.329615	0.0288539	0.330902	0.091 (7)
82	-1.23084	-0.645693	-0.272305	1.41635	0.206 (6)
83	-2.14649	-0.633722	-0.147667	2.24296	0.202 (13)
85	0.392853	0.0771914	-0.34627	0.529334	0.202 (10)
91	-1.05548	-0.278253	-0.212519	1.11204	0.178 (20)
93	-0.026863	0.647333	-0.0882452	0.653872	0.165 (16)
97	2.85294	-3.26553	0.253411	4.34364	0.361 (40)
100	2.04658	0.73005	-3.0964	3.78274	0.818 (28)
104	-5.65876	9.84327	5.35301	12.5525	1.073 (21)
107	0.896016	-1.13076	-0.461047	1.5146	0.100 (21)
109	0.756959	0.901035	2.13564	2.4384	0.262 (22)
**Total**	**2.7826**	**2.76526**	**2.58547**	**4.69832**	**0.390**

Table 10. Control points.
X - Easting, Y - Northing, Z - Altitude.

Label	X error (cm)	Y error (cm)	Z error (cm)	Total (cm)	Image (pix)
5	-51.9779	109.862	37.7706	127.271	0.475 (38)
6	7.89992	-35.2258	-31.5894	47.9703	0.244 (66)
9	17.8596	8.53701	-44.6925	48.8802	0.243 (29)

Label	X error (cm)	Y error (cm)	Z error (cm)	Total (cm)	Image (pix)
15	-2.03578	1.48097	1.71823	3.04795	0.115 (27)
25	2.13799	2.37073	7.6222	8.26373	0.101 (22)
28	0.190171	-2.63331	-7.48252	7.93465	0.163 (29)
29	2.33491	11.8006	-53.8759	55.2025	0.028 (18)
30	-0.807976	1.78724	-0.192848	1.97085	0.103 (20)
31	1.63423	0.483852	8.49726	8.6665	0.133 (26)
36	-9.72509	9.87671	3.61017	14.3234	0.135 (14)
39	8.4478	-11.602	-6.07204	15.5834	0.204 (40)
41	1.39248	1.77904	-10.01	10.2618	0.390 (56)
42	2.75846	-2.52595	-11.0612	11.6765	0.368 (48)
44	-6.72761	0.554351	-5.49646	8.70512	0.601 (73)
45	-0.668747	3.76114	-9.02986	9.80468	0.554 (94)
46	6.29539	2.23225	-17.5818	18.8078	0.397 (63)
47	2.92782	6.00466	-15.5277	16.9038	0.474 (107)
49	24.7559	-51.0557	-194.388	202.5	0.764 (59)
50	8.1244	-8.39493	-36.8248	38.6335	0.424 (56)
51	3.02149	1.0147	-2.59192	4.10817	0.348 (42)
52	0.471954	-12.4707	-25.4244	28.3221	0.309 (56)
54	12.2131	0.548322	1.617	12.3318	0.118 (31)
55	-3.85815	8.87265	-1.17096	9.74579	0.338 (51)
59	-1.87898	-8.83511	9.38167	13.0233	0.244 (9)
64	-14.2994	-15.8295	5.33583	21.989	0.288 (13)
65	-11.396	0.18539	3.38317	11.889	0.348 (31)
66	1.42709	6.38834	51.7948	52.2067	0.181 (22)
67	0.87314	9.5528	36.1644	37.415	0.160 (12)
68	-3.90769	8.09458	17.0152	19.2434	0.137 (16)
69	6.34432	-12.4873	6.70943	15.5306	0.213 (28)
71	-8.43637	0.637322	-4.31841	9.4988	0.215 (24)
74	3.48499	-6.72187	3.95807	8.54371	0.106 (10)
75	-0.892538	8.06716	-3.66351	8.90488	0.024 (6)
79	-9.23374	2.3614	18.4708	20.7848	0.142 (8)
81	-4.25851	-2.34563	-9.96972	11.092	0.236 (6)

Label	X error (cm)	Y error (cm)	Z error (cm)	Total (cm)	Image (pix)
84	6.6741	4.13144	-2.18375	8.14746	0.169 (12)
87	-0.30421	-1.73522	-19.185	19.2657	0.232 (10)
88	-3.05403	-1.88698	-21.8796	22.1721	0.233 (13)
89	-0.231378	-7.10655	-19.0532	20.3367	0.156 (27)
90	1.64377	0.573092	5.24934	5.53046	0.149 (28)
92	0.988389	1.38371	2.28704	2.84993	0.222 (18)
94	-5.05188	-3.80745	-29.4069	30.0796	0.142 (35)
95	-3.91247	-0.929921	-2.54246	4.75775	0.244 (38)
96	-3.02039	2.21278	-4.70511	6.01309	0.186 (23)
98	17.0132	-4.00761	-1.0674	17.5114	0.567 (32)
99	30.9835	-7.37588	-13.6856	34.6652	0.567 (30)
101	-16.7346	7.7378	-2.46353	18.6008	0.671 (26)
102	0.772009	17.0044	-26.3843	31.3987	0.362 (21)
103	-8.89695	11.0324	-20.6508	25.0465	0.476 (20)
105	-5.027	18.9537	-17.4771	26.2671	0.199 (24)
106	-15.2421	-2.97977	20.5064	25.7237	0.085 (13)
108	-0.468202	-0.995826	3.94396	4.0946	0.178 (19)
110	-2.29123	3.22333	-7.17365	8.19151	0.261 (28)
111	1.23457	-5.07295	1.46444	5.4225	0.140 (14)
**Total**	**11.2113**	**18.5581**	**32.6152**	**39.1643**	**0.381**

Table 11. Check points.

X - Easting, Y - Northing, Z - Altitude.

Digital Elevation Model

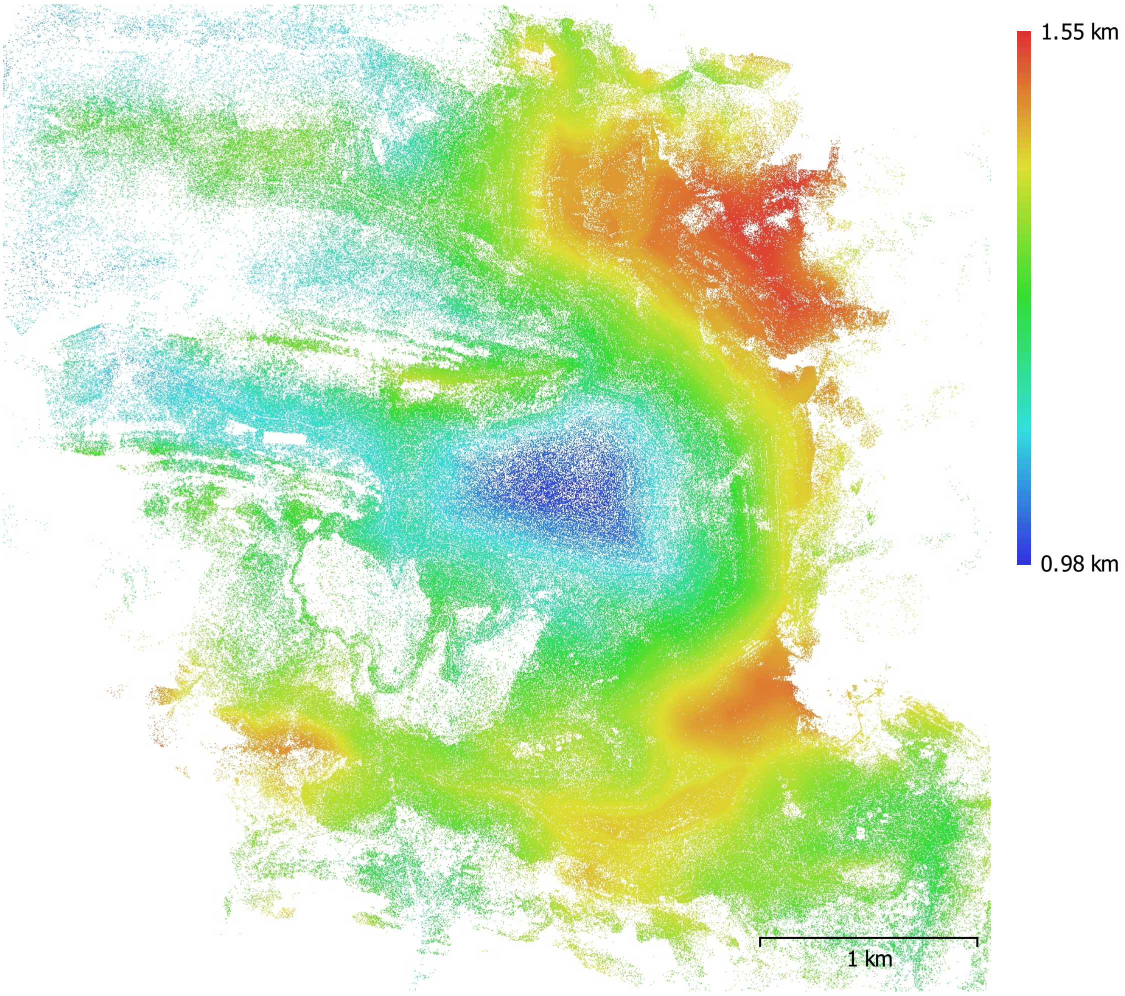

Fig. 9. Reconstructed digital elevation model.

Resolution:	unknown
Point density:	unknown

Processing Parameters

**General**	
Cameras	2595
Aligned cameras	2577
Markers	110
**Shapes**	
Polygon	1
Coordinate system	ETRS89 / UTM zone 30N (EPSG::25830)
Rotation angles	Yaw, Pitch, Roll
**Tie Points**	
Points	1,797,730 of 12,529,745
RMS reprojection error	0.138349 (0.3288 pix)
Max reprojection error	0.299902 (1.807 pix)
Mean key point size	2.33415 pix
Point colors	3 bands, uint8
Key points	No
Average tie point multiplicity	3.65511
**Alignment parameters**	
Accuracy	High
Generic preselection	Yes
Reference preselection	No
Key point limit	60,000
Key point limit per Mpx	1,000
Tie point limit	0
Exclude stationary tie points	Yes
Guided image matching	No
Adaptive camera model fitting	No
Matching time	4 hours 7 minutes
Matching memory usage	3.73 GB
Alignment time	2 hours 17 minutes
Alignment memory usage	4.82 GB
**Optimization parameters**	
Parameters	f, cx, cy, k1-k3, p1, p2
Adaptive camera model fitting	No
Optimization time	29 seconds
Date created	2023:11:13 15:04:46
Software version	2.0.0.15597
File size	776.15 MB
**System**	
Software name	Agisoft Metashape Professional
Software version	2.0.3 build 16960
OS	Windows 64 bit
RAM	63.90 GB
CPU	Intel(R) Core(TM) i7-7700 CPU @ 3.60GHz
GPU(s)	Quadro M4000
