# Supplementary material for: Automated cleaning of tie point clouds following USGS guidelines in Agisoft Metashape professional (ver. 2.1.0)
Source: MethodsX. 2024 Mar 26;12:102679. doi: 10.1016/j.mex.2024.102679 (PMC10992719; doi:10.1016/j.mex.2024.102679)
Supplement: Supplementary file 3 — The supplementary material includes supplementary text, figures and the processing reports generated by the software. [file mmc3.zip › Lucia_SCC-Optimized_r1.pdf]

Lucia_SCC-Optimized_r1

Automatically cleaned sparse cloud using the SCC script (optimized settings). UAS data provided by Sanz-Ablanedo et al. (2018).

Sanz-Ablanedo, E., Chandler, J. H., Rodríguez-Pérez, J. R., and Ordóñez, C.: Accuracy of Unmanned Aerial Vehicle (UAV) and SfM Photogrammetry Survey as a Function of the Number and Location of Ground Control Points Used, Remote Sensing, 10, 1606, 2018.

28 December 2023

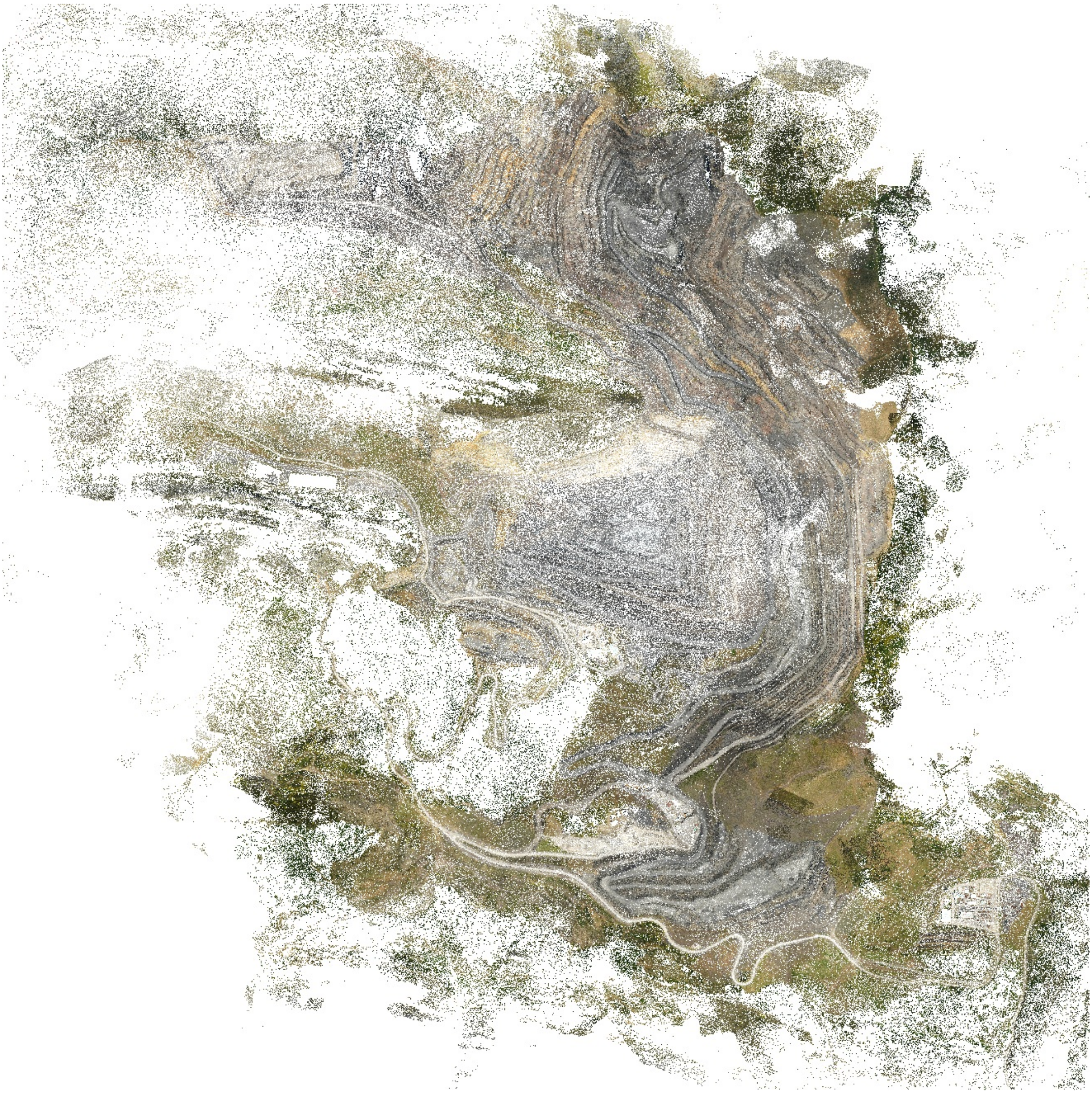

Survey Data

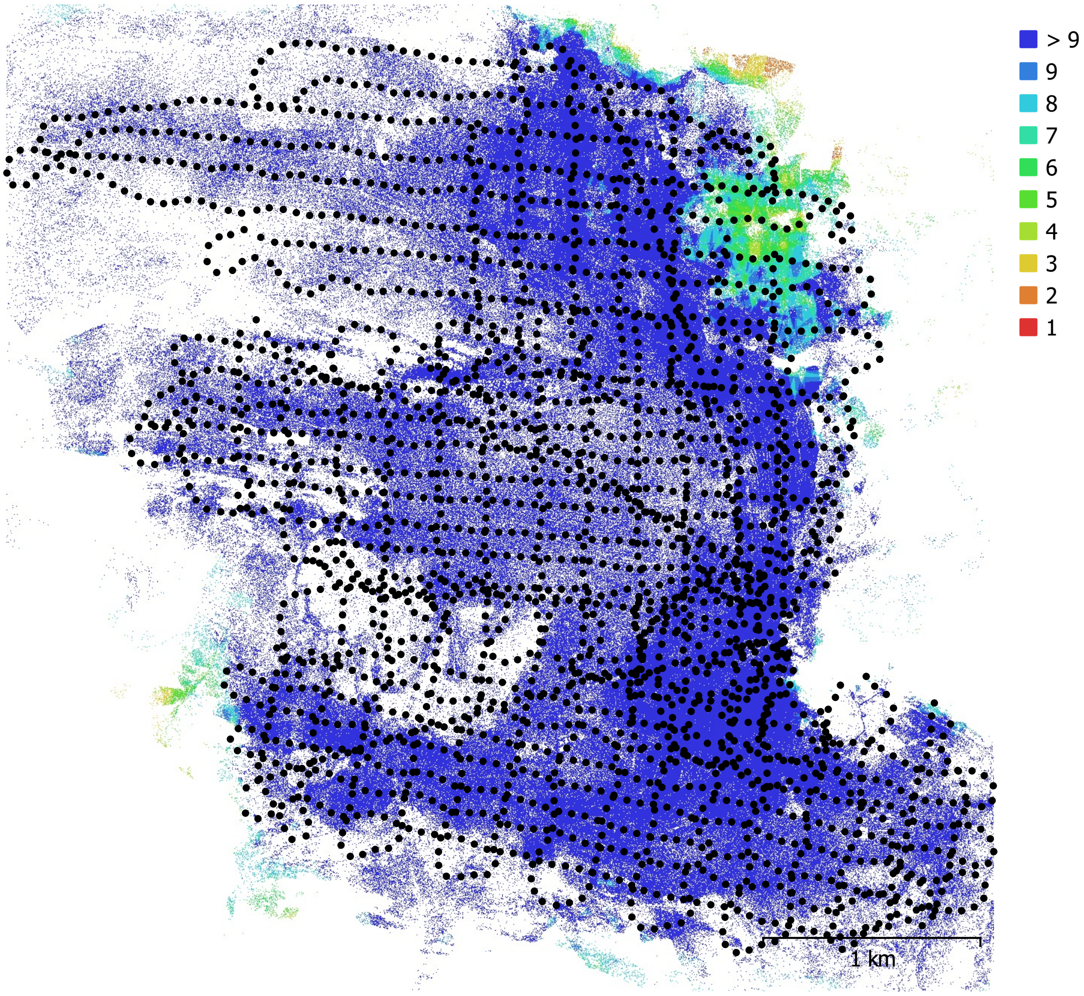

Fig. 1. Camera locations and image overlap.

Number of images:	2,595	Camera stations:	2,577
Flying altitude:	349 m	Tie points:	1,736,575
Ground resolution:	6.2 cm/pix	Projections:	4,093,683
Coverage area:	7.49 km²	Reprojection error:	0.325 pix

Camera Model	Resolution	Focal Length	Pixel Size	Precalibrated
NX500 (20 mm)	6480 x 4320	20 mm	3.7 x 3.7 μm	No
NX500 (20 mm)	6480 x 4320	20 mm	3.7 x 3.7 μm	No
NX500 (20 mm)	6480 x 4320	20 mm	3.7 x 3.7 μm	No
NX500 (20 mm)	6480 x 4320	20 mm	3.7 x 3.7 μm	No
NX500 (20 mm)	6480 x 4320	20 mm	3.7 x 3.7 μm	No

Camera Model	Resolution	Focal Length	Pixel Size	Precalibrated
NX500 (20 mm)	6480 x 4320	20 mm	3.7 x 3.7 μm	No

Table 1. Cameras.

Camera Calibration

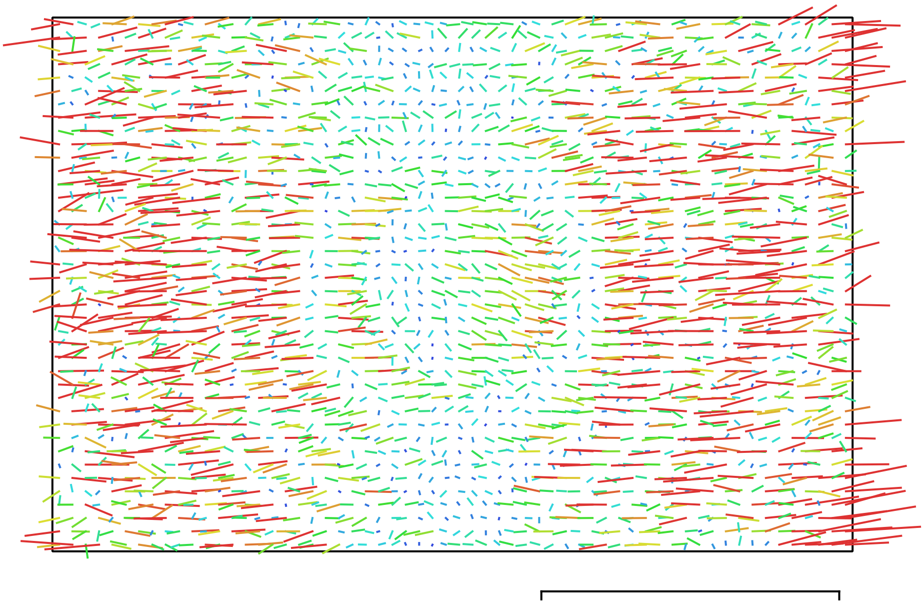

Fig. 2. Image residuals for NX500 (20 mm).

NX500 (20 mm)

200 images

Type	Resolution	Focal Length	Pixel Size
**Frame**	**6480 x 4320**	**20 mm**	**3.7 x 3.7 μm**

F:	5619.45		
Cx:	93.0166	B1:	0
Cy:	37.2805	B2:	0
K1:	-0.0119393	P1:	0.00275128
K2:	0.0251053	P2:	0.000825825
K3:	-0.0208381	P3:	0
K4:	0	P4:	0

Camera Calibration

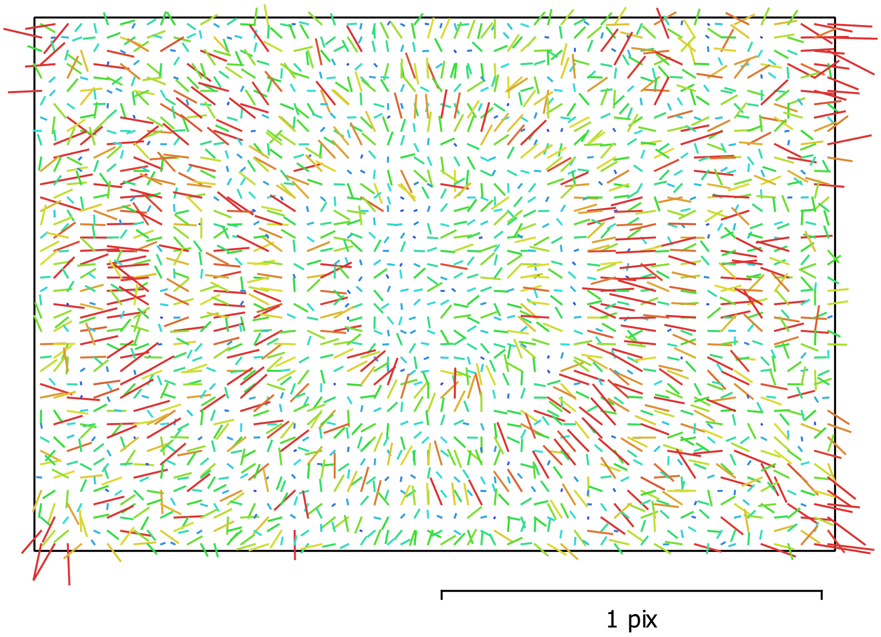

Fig. 3. Image residuals for NX500 (20 mm).

NX500 (20 mm)

462 images

Type	Resolution	Focal Length	Pixel Size
**Frame**	**6480 x 4320**	**20 mm**	**3.7 x 3.7 μm**

F:	5628.79		
Cx:	71.1376	B1:	0
Cy:	44.0059	B2:	0
K1:	-0.0115019	P1:	0.00224893
K2:	0.0253464	P2:	0.00117141
K3:	-0.0220892	P3:	0
K4:	0	P4:	0

Camera Calibration

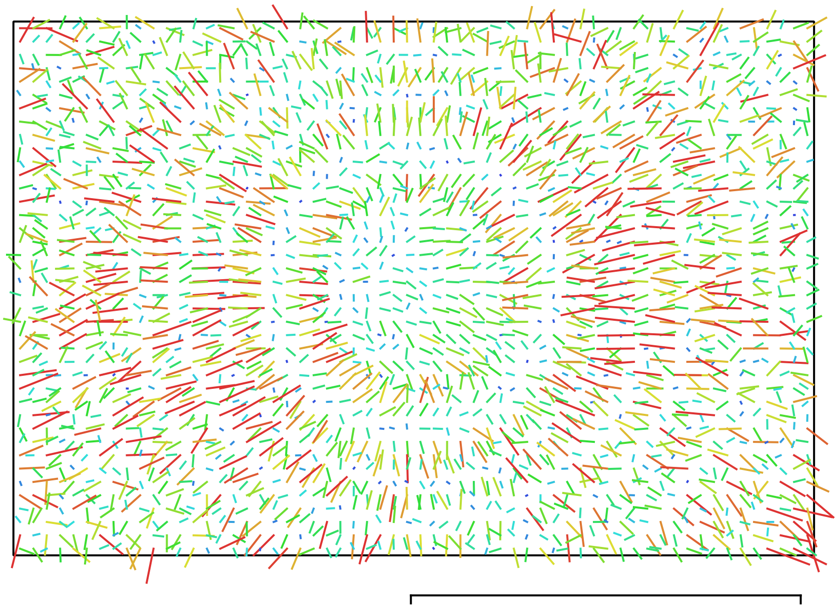

Fig. 4. Image residuals for NX500 (20 mm).

NX500 (20 mm)

530 images

Type	Resolution	Focal Length	Pixel Size
**Frame**	**6480 x 4320**	**20 mm**	**3.7 x 3.7 µm**

F:	5628.65		
Cx:	84.0445	B1:	0
Cy:	35.1545	B2:	0
K1:	-0.0120429	P1:	0.00253817
K2:	0.0307375	P2:	0.000926613
K3:	-0.0323232	P3:	0
K4:	0	P4:	0

Camera Calibration

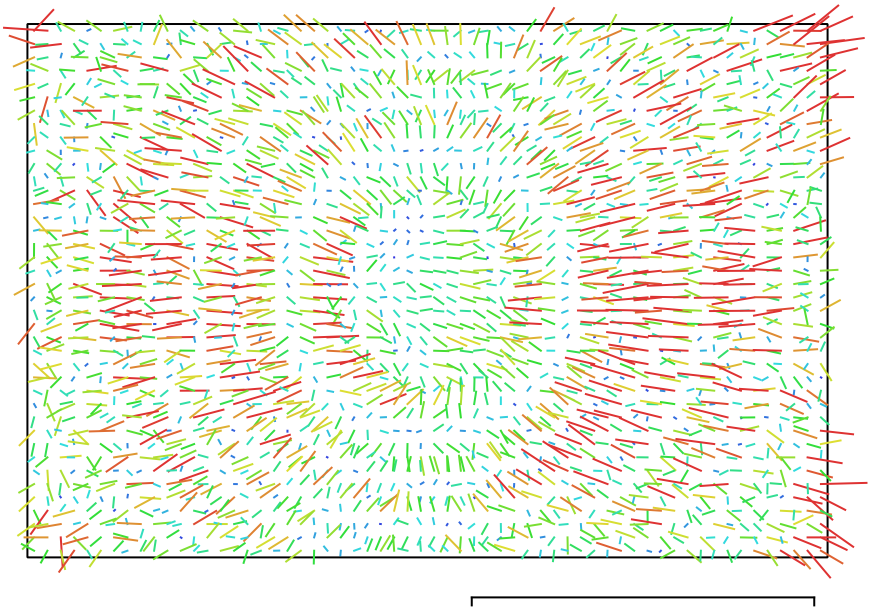

Fig. 5. Image residuals for NX500 (20 mm).

NX500 (20 mm)

513 images

Type	Resolution	Focal Length	Pixel Size
**Frame**	**6480 x 4320**	**20 mm**	**3.7 x 3.7 μm**

F:	5623.89		
Cx:	83.9675	B1:	0
Cy:	60.2792	B2:	0
K1:	-0.0107957	P1:	0.00251211
K2:	0.0226508	P2:	0.00150877
K3:	-0.0172228	P3:	0
K4:	0	P4:	0

Camera Calibration

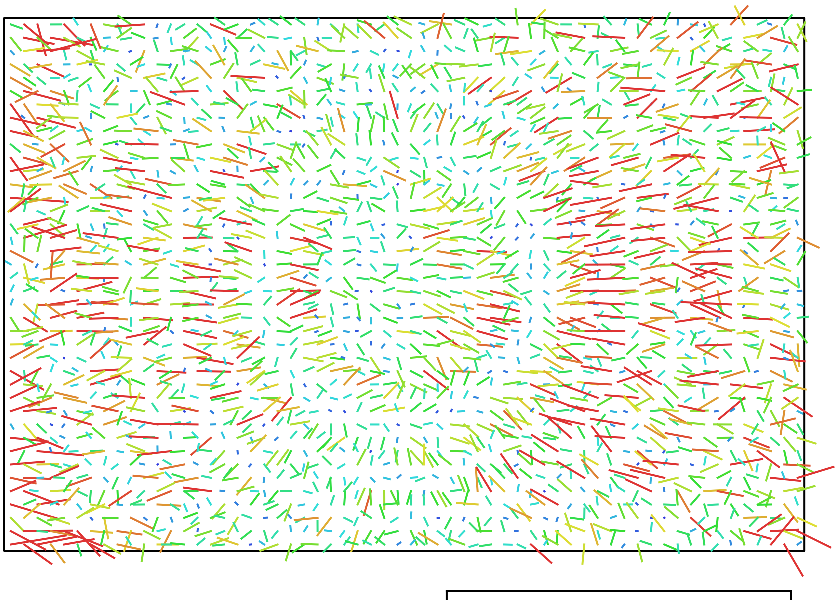

Fig. 6. Image residuals for NX500 (20 mm).

NX500 (20 mm)

412 images

Type	Resolution	Focal Length	Pixel Size
**Frame**	**6480 x 4320**	**20 mm**	**3.7 x 3.7 μm**

F:	5626.93		
Cx:	88.3981	B1:	0
Cy:	45.8618	B2:	0
K1:	-0.0128455	P1:	0.00259516
K2:	0.0329102	P2:	0.00115416
K3:	-0.0359777	P3:	0
K4:	0	P4:	0

Camera Calibration

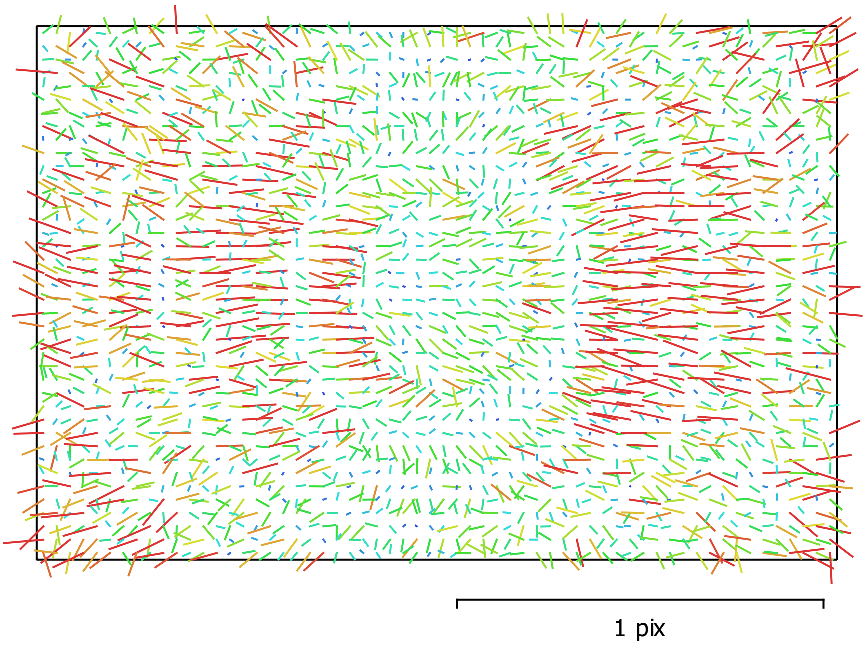

Fig. 7. Image residuals for NX500 (20 mm).

NX500 (20 mm)

478 images

Type	Resolution	Focal Length	Pixel Size
**Frame**	**6480 x 4320**	**20 mm**	**3.7 x 3.7 μm**

F:	5627.2		
Cx:	68.9021	B1:	0
Cy:	47.9746	B2:	0
K1:	-0.0124372	P1:	0.00201639
K2:	0.0348936	P2:	0.00123732
K3:	-0.0387337	P3:	0
K4:	0	P4:	0

Ground Control Points

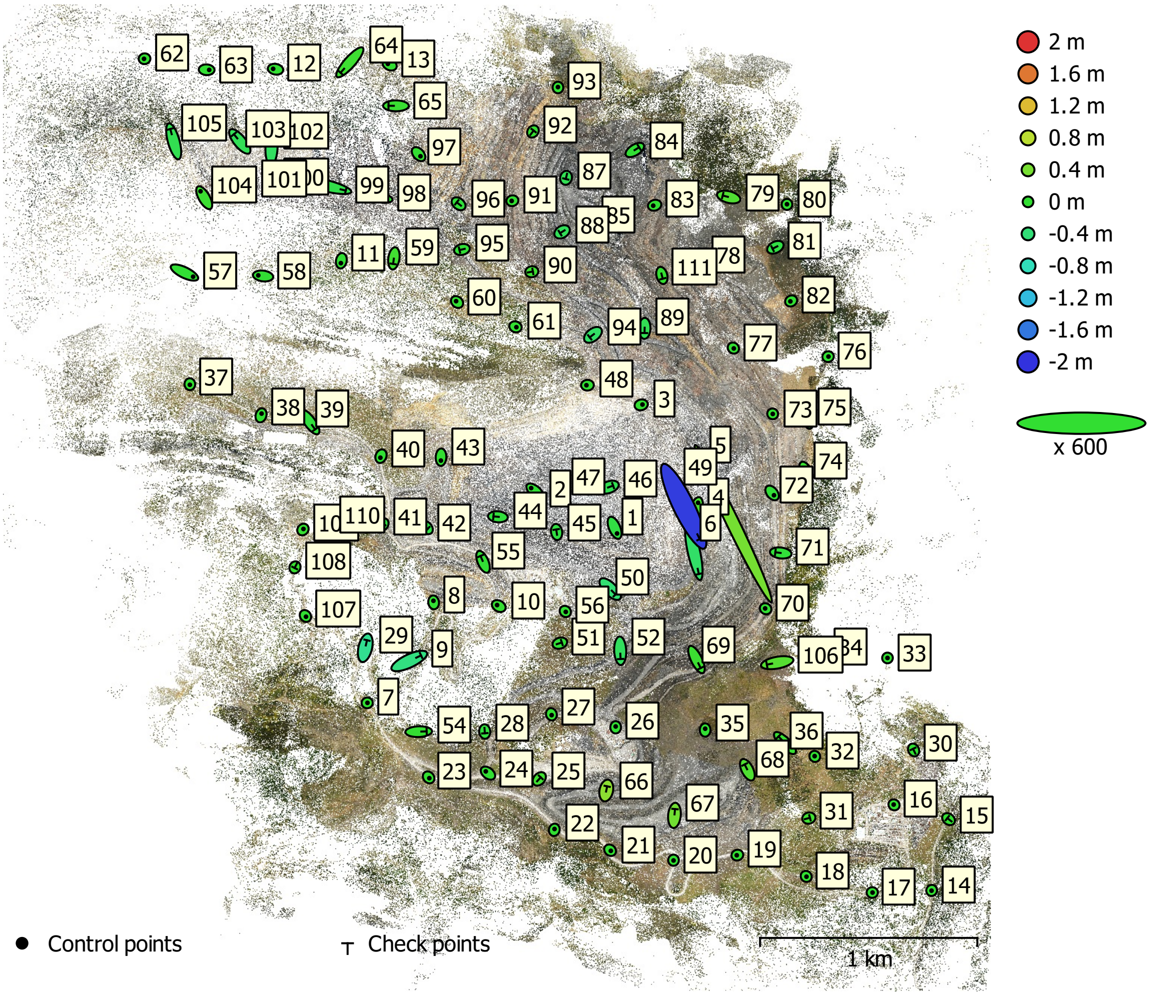

Fig. 8. GCP locations and error estimates.
Z error is represented by ellipse color. X,Y errors are represented by ellipse shape.
Estimated GCP locations are marked with a dot or crossing.

Count	X error (cm)	Y error (cm)	Z error (cm)	XY error (cm)	Total (cm)
55	2.7666	2.74892	2.54884	3.90008	4.6591

Table 2. Control points RMSE.
X - Easting, Y - Northing, Z - Altitude.

Count	X error (cm)	Y error (cm)	Z error (cm)	XY error (cm)	Total (cm)
54	11.4821	18.7439	32.6612	21.9812	39.3691

Table 3. Check points RMSE.
X - Easting, Y - Northing, Z - Altitude.

Label	X error (cm)	Y error (cm)	Z error (cm)	Total (cm)	Image (pix)
1	3.79059	-8.72828	-10.0469	13.8381	0.496 (104)
2	-5.90164	4.94991	-0.542976	7.72177	0.480 (109)
3	2.00057	0.514843	0.161853	2.07208	0.140 (51)
4	2.18243	7.82781	13.7791	15.9969	0.610 (50)
7	0.411962	0.0839586	-0.00446184	0.420454	0.076 (24)
8	-0.309119	2.42367	-0.119236	2.44621	0.346 (32)
10	-2.54321	1.50097	2.30516	3.74627	0.490 (42)
11	-0.694627	-3.64812	-0.622139	3.76542	0.256 (36)
12	-3.62776	0.617611	0.370897	3.6986	0.396 (26)
13	-2.81555	1.34203	0.438521	3.14971	0.177 (20)
14	-0.178435	-0.381574	0.0620505	0.425779	0.070 (23)
16	-0.532431	0.44034	0.096796	0.697676	0.087 (34)
17	0.43325	-0.00300769	-0.0484146	0.435958	0.069 (23)
18	0.439942	-0.424938	-0.0464096	0.613413	0.110 (25)
19	-0.860961	-0.259177	-0.0822363	0.902879	0.101 (20)
20	-0.00422268	-0.395055	-0.0497473	0.398198	0.117 (16)
21	1.12082	-0.948316	0.190594	1.4805	0.126 (15)
22	0.338225	1.30648	-0.0103195	1.34959	0.114 (13)
23	1.07909	-1.24635	0.127139	1.65348	0.129 (18)
24	-3.36654	2.65475	-0.530428	4.32003	0.325 (27)
26	-0.00110881	1.25749	-0.556901	1.37529	0.120 (33)
27	0.403041	-1.29149	0.0870033	1.35571	0.180 (27)
32	-0.226639	0.403652	-0.00691293	0.462977	0.065 (18)
33	0.0102179	0.00431302	-0.00119412	0.011155	0.001 (3)
34	-0.0151269	0.0628672	-0.02342	0.0687721	0.013 (4)
35	0.0451091	2.0017	-0.484759	2.06005	0.252 (11)
37	0.0558693	-0.911651	0.249373	0.946792	0.118 (46)
38	0.837448	2.70289	-0.53905	2.88054	0.178 (57)
40	-0.910931	-2.32512	-1.36901	2.84783	0.268 (66)
43	-0.225882	-5.17946	0.279412	5.19191	0.485 (69)
48	-1.55922	-0.0555804	-0.525264	1.64625	0.178 (44)

Label	X error (cm)	Y error (cm)	Z error (cm)	Total (cm)	Image (pix)
56	0.697091	-0.684037	-0.0768175	0.979665	0.308 (50)
57	13.3184	-6.36509	-1.56819	14.8443	1.474 (14)
58	-7.35687	0.942224	-0.956254	7.47835	0.847 (30)
60	-1.45522	1.30816	0.125562	1.96079	0.147 (32)
61	1.37744	-0.562526	0.549381	1.58606	0.119 (20)
62	-0.821214	-0.123684	0.196185	0.853334	0.145 (17)
63	3.91677	-0.20953	-0.282444	3.93252	0.303 (16)
70	-0.787038	0.480638	-0.639944	1.12248	0.093 (30)
72	2.85576	-3.50057	-2.99719	5.42149	0.256 (18)
73	0.197398	-0.142394	0.217407	0.326355	0.058 (15)
76	0.593813	0.156999	-0.0134601	0.614365	0.089 (9)
77	0.663539	-0.564769	0.016757	0.871509	0.083 (11)
78	0.424532	-1.48247	-0.29453	1.56994	0.118 (11)
80	0.0130291	0.303135	0.0309337	0.304987	0.086 (7)
82	-1.15927	-0.636562	-0.266183	1.34906	0.198 (6)
83	-2.12251	-0.644325	-0.119915	2.22139	0.196 (13)
85	0.396421	0.0443869	-0.358122	0.53607	0.198 (10)
91	-1.04626	-0.293477	-0.195587	1.1041	0.172 (20)
93	-0.0324131	0.642254	-0.108415	0.652146	0.159 (16)
97	2.95273	-3.22475	0.269696	4.38068	0.360 (40)
100	2.01586	0.669016	-3.09807	3.75623	0.816 (28)
104	-5.66658	9.8332	5.34572	12.5451	1.071 (21)
107	0.880834	-1.12891	-0.455666	1.50264	0.100 (21)
109	0.768643	0.885625	2.14078	2.44092	0.260 (22)
**Total**	**2.7666**	**2.74892**	**2.54884**	**4.6591**	**0.389**

Table 4. Control points.

X - Easting, Y - Northing, Z - Altitude.

Label	X error (cm)	Y error (cm)	Z error (cm)	Total (cm)	Image (pix)
5	-54.3051	111.991	38.678	130.334	0.469 (38)
6	7.66803	-34.4878	-30.5808	46.7268	0.240 (66)
9	18.0803	8.50382	-45.5534	49.7426	0.241 (29)

Label	X error (cm)	Y error (cm)	Z error (cm)	Total (cm)	Image (pix)
15	-1.90968	1.49657	0.912346	2.5921	0.112 (27)
25	2.21932	2.39973	7.97636	8.62012	0.099 (22)
28	0.200922	-2.63557	-7.63962	8.08396	0.162 (29)
29	2.68563	11.618	-54.5303	55.8188	0.027 (18)
30	-0.849537	1.7518	-0.30416	1.97054	0.101 (20)
31	1.64401	0.441496	8.32738	8.49959	0.129 (26)
36	-9.90628	9.9837	3.67988	14.5379	0.132 (14)
39	8.16597	-11.3699	-6.17688	15.3007	0.202 (40)
41	1.36434	1.73711	-10.118	10.3563	0.388 (56)
42	2.68643	-2.49568	-11.2394	11.8224	0.366 (48)
44	-6.77839	0.599476	-5.59755	8.81127	0.600 (73)
45	-0.602889	3.8528	-9.0031	9.81139	0.553 (94)
46	6.28088	2.28252	-17.6677	18.8893	0.392 (63)
47	3.03305	6.18952	-15.5729	17.0301	0.472 (107)
49	24.585	-50.3388	-193.829	201.763	0.760 (59)
50	8.17082	-8.28426	-36.785	38.5815	0.421 (56)
51	3.02203	1.00702	-2.72385	4.19119	0.345 (42)
52	0.51992	-12.6742	-25.5729	28.5461	0.306 (56)
54	12.2466	0.45276	1.18347	12.312	0.116 (31)
55	-3.89406	8.94834	-1.28045	9.84256	0.336 (51)
59	-1.21964	-8.57985	9.16361	12.6124	0.241 (9)
64	-14.2692	-16.0342	6.47947	22.4207	0.287 (13)
65	-11.4115	0.066792	3.82388	12.0353	0.348 (31)
66	1.34339	6.43654	52.4731	52.8835	0.177 (22)
67	0.886971	9.71576	36.2734	37.5626	0.156 (12)
68	-4.10536	8.14641	16.7274	19.0532	0.133 (16)
69	6.45608	-13.1115	5.76931	15.7123	0.208 (28)
71	-8.26631	1.00907	-4.5335	9.4817	0.213 (24)
74	3.3402	-6.93801	3.85491	8.61123	0.104 (10)
75	-1.10578	7.8356	-3.26391	8.55993	0.023 (6)
79	-9.20945	2.34638	17.7204	20.108	0.139 (8)
81	-4.32923	-2.15585	-10.5694	11.6234	0.231 (6)

Label	X error (cm)	Y error (cm)	Z error (cm)	Total (cm)	Image (pix)
84	6.5631	4.0622	-1.72706	7.90939	0.163 (12)
87	-0.435193	-1.79235	-19.1668	19.2553	0.224 (10)
88	-3.14967	-1.92535	-22.0353	22.3424	0.225 (13)
89	-0.162741	-7.15687	-19.8669	21.1174	0.151 (27)
90	1.6625	0.307916	6.15518	6.38318	0.143 (28)
92	1.04873	1.35851	2.23485	2.81779	0.220 (18)
94	-5.01102	-3.84929	-29.7791	30.4421	0.139 (35)
95	-3.83835	-0.9006	-2.57022	4.70638	0.240 (38)
96	-2.78189	2.1841	-5.11681	6.22021	0.183 (23)
98	17.5241	-3.90403	-0.736719	17.9689	0.560 (32)
99	31.6384	-7.72478	-13.767	35.3581	0.564 (30)
101	-16.9173	7.67256	-2.5731	18.7532	0.670 (26)
102	0.741162	17.0431	-26.8168	31.783	0.361 (21)
103	-9.05002	11.0546	-21.0475	25.4383	0.476 (20)
105	-5.25906	18.9498	-18.1476	26.7598	0.201 (24)
106	-15.9199	-2.97121	20.7537	26.3247	0.082 (13)
108	-0.497019	-0.982977	4.05988	4.20664	0.176 (19)
110	-2.3486	3.23087	-7.34357	8.35957	0.259 (28)
111	1.34105	-5.04047	0.970835	5.3054	0.138 (14)
**Total**	**11.4821**	**18.7439**	**32.6612**	**39.3691**	**0.378**

Table 5. Check points.

X - Easting, Y - Northing, Z - Altitude.

Digital Elevation Model

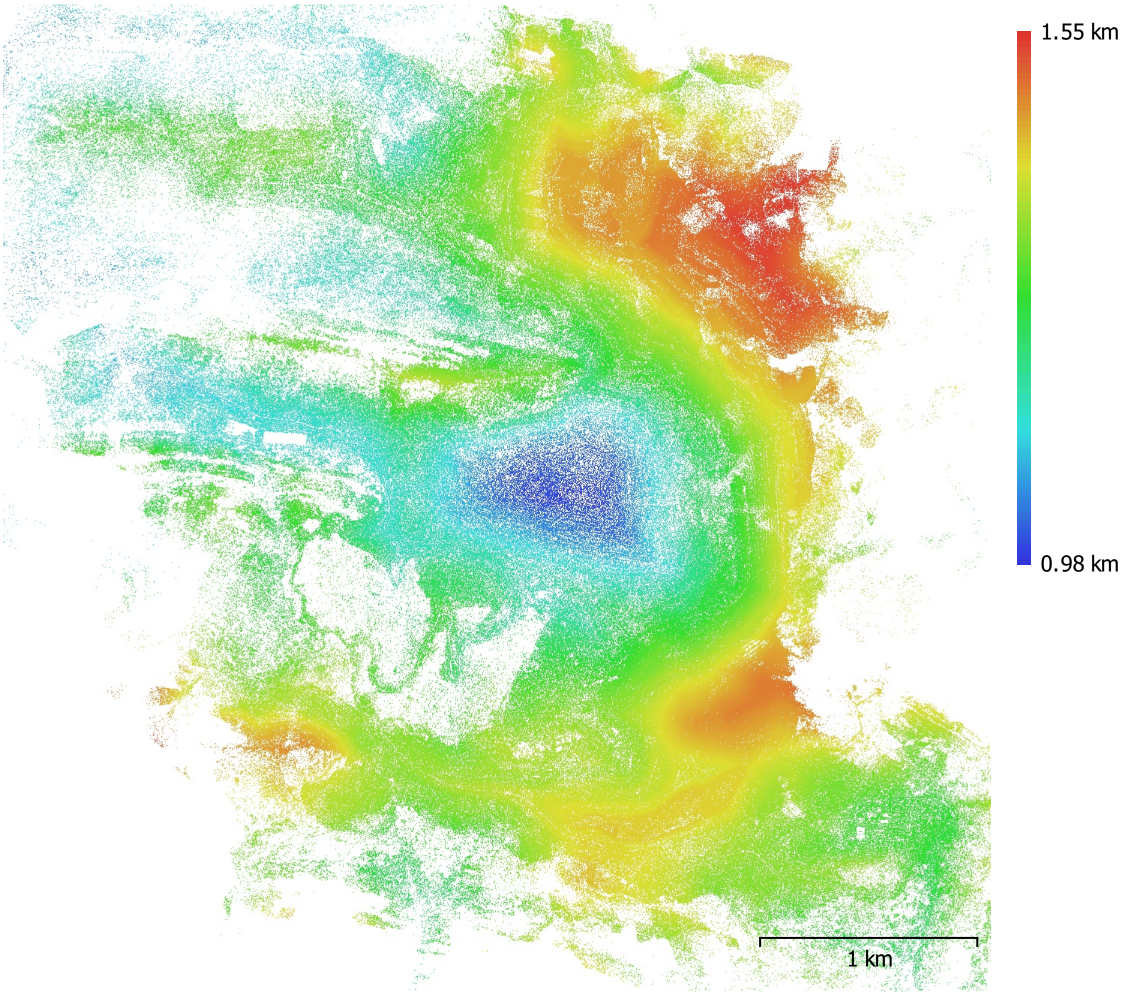

Fig. 9. Reconstructed digital elevation model.

Resolution:	unknown
Point density:	unknown

Processing Parameters

**General**	
Cameras	2595
Aligned cameras	2577
Markers	110
**Shapes**	
Polygon	1
Coordinate system	ETRS89 / UTM zone 30N (EPSG::25830)
Rotation angles	Yaw, Pitch, Roll
**Tie Points**	
Points	1,736,575 of 12,529,745
RMS reprojection error	0.138186 (0.325103 pix)
Max reprojection error	0.299859 (1.58949 pix)
Mean key point size	2.31253 pix
Point colors	3 bands, uint8
Key points	No
Average tie point multiplicity	3.65511
**Alignment parameters**	
Accuracy	High
Generic preselection	Yes
Reference preselection	No
Key point limit	60,000
Key point limit per Mpx	1,000
Tie point limit	0
Exclude stationary tie points	Yes
Guided image matching	No
Adaptive camera model fitting	No
Matching time	4 hours 7 minutes
Matching memory usage	3.73 GB
Alignment time	2 hours 17 minutes
Alignment memory usage	4.82 GB
**Optimization parameters**	
Parameters	f, cx, cy, k1-k3, p1, p2
Adaptive camera model fitting	No
Optimization time	27 seconds
Date created	2023:11:13 15:04:46
Software version	2.0.0.15597
File size	774.75 MB
**System**	
Software name	Agisoft Metashape Professional
Software version	2.0.3 build 16960
OS	Windows 64 bit
RAM	63.90 GB
CPU	Intel(R) Core(TM) i7-7700 CPU @ 3.60GHz
GPU(s)	Quadro M4000
