# Supplementary material for: Automated cleaning of tie point clouds following USGS guidelines in Agisoft Metashape professional (ver. 2.1.0)
Source: MethodsX. 2024 Mar 26;12:102679. doi: 10.1016/j.mex.2024.102679 (PMC10992719; doi:10.1016/j.mex.2024.102679)
Supplement: Supplementary file 3 — The supplementary material includes supplementary text, figures and the processing reports generated by the software. [file mmc3.zip › Lucia_SCC-Optimized_r2.pdf]

Lucia_SCC-Optimized_r2

Automatically cleaned sparse cloud using the SCC script (optimized settings). UAS data provided by Sanz-Ablanedo et al. (2018).

Sanz-Ablanedo, E., Chandler, J. H., Rodríguez-Pérez, J. R., and Ordóñez, C.: Accuracy of Unmanned Aerial Vehicle (UAV) and SfM Photogrammetry Survey as a Function of the Number and Location of Ground Control Points Used, Remote Sensing, 10, 1606, 2018.

28 December 2023

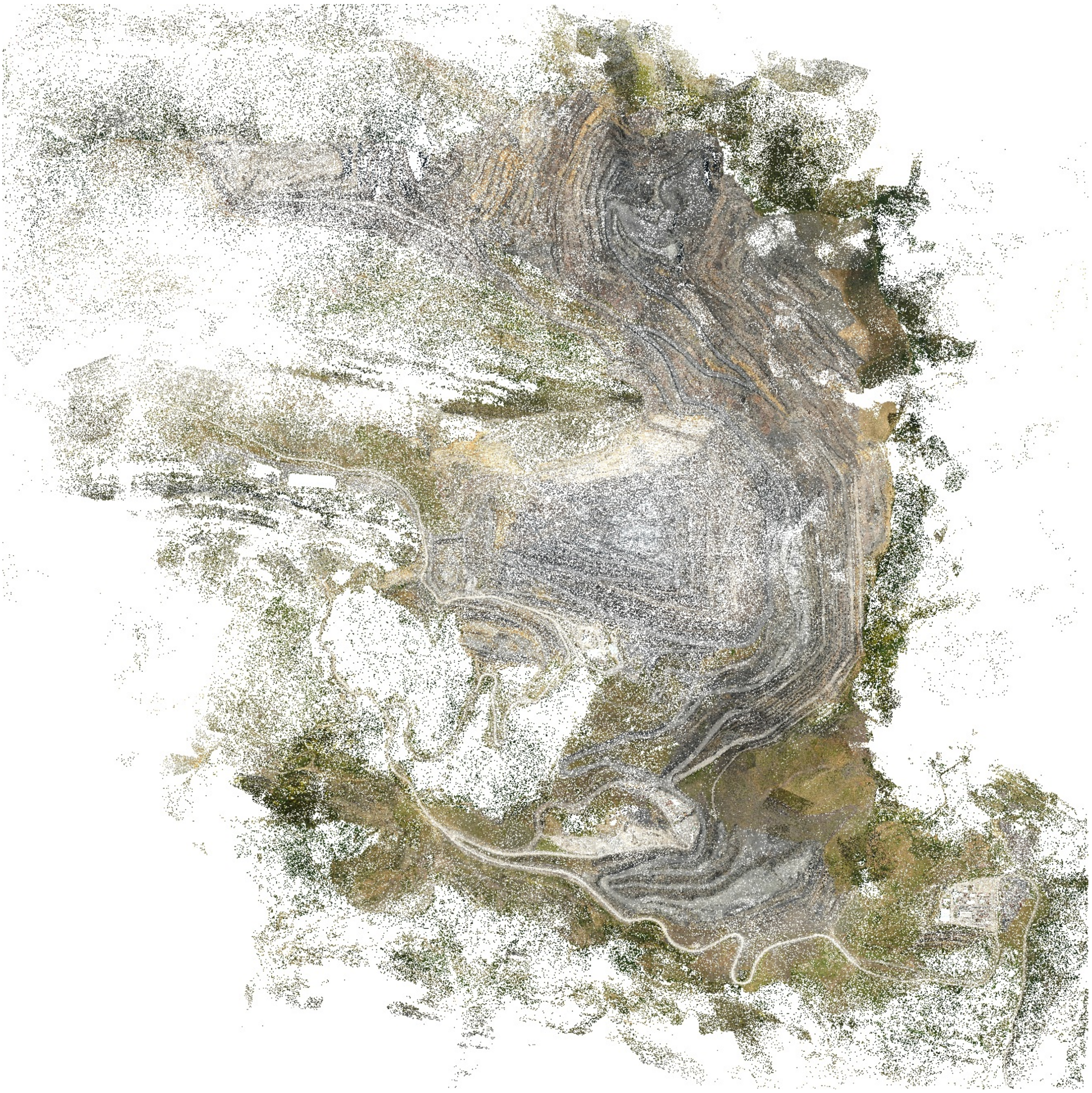

Survey Data

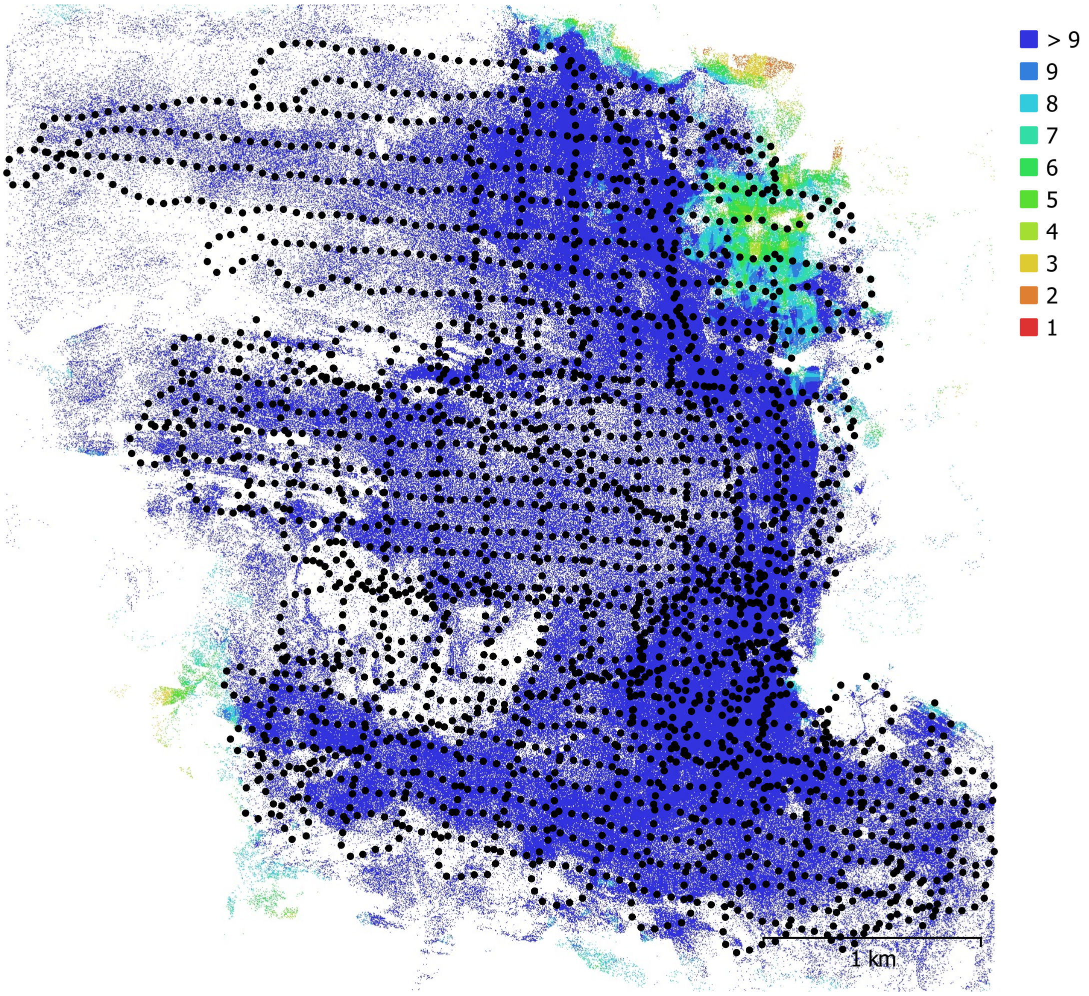

Fig. 1. Camera locations and image overlap.

Number of images:	2,595	Camera stations:	2,577
Flying altitude:	349 m	Tie points:	1,755,989
Ground resolution:	6.2 cm/pix	Projections:	4,150,099
Coverage area:	7.5 km²	Reprojection error:	0.327 pix

Camera Model	Resolution	Focal Length	Pixel Size	Precalibrated
NX500 (20 mm)	6480 x 4320	20 mm	3.7 x 3.7 μm	No
NX500 (20 mm)	6480 x 4320	20 mm	3.7 x 3.7 μm	No
NX500 (20 mm)	6480 x 4320	20 mm	3.7 x 3.7 μm	No
NX500 (20 mm)	6480 x 4320	20 mm	3.7 x 3.7 μm	No
NX500 (20 mm)	6480 x 4320	20 mm	3.7 x 3.7 μm	No

Camera Model	Resolution	Focal Length	Pixel Size	Precalibrated
NX500 (20 mm)	6480 x 4320	20 mm	3.7 x 3.7 μm	No

Table 1. Cameras.

Camera Calibration

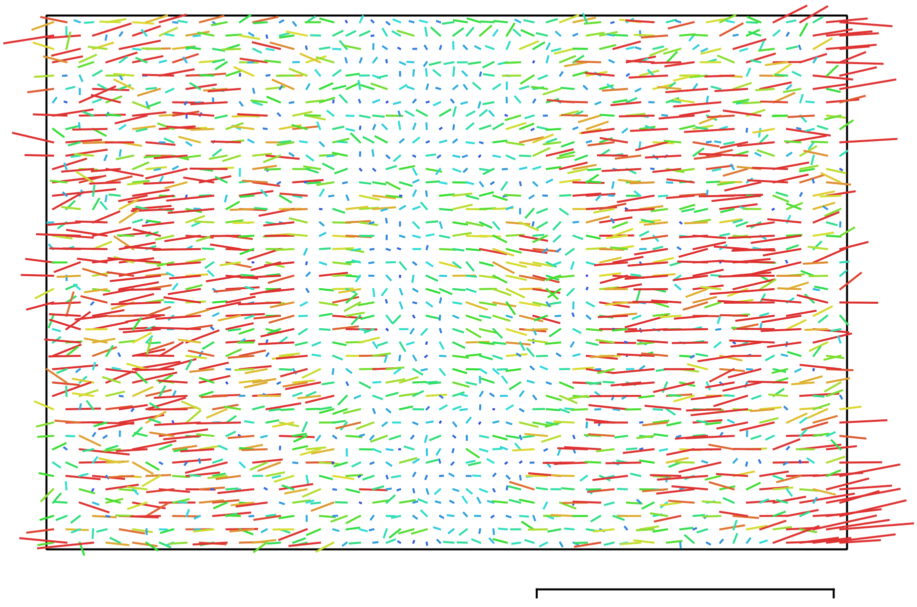

Fig. 2. Image residuals for NX500 (20 mm).

NX500 (20 mm)

200 images

Type	Resolution	Focal Length	Pixel Size
**Frame**	**6480 x 4320**	**20 mm**	**3.7 x 3.7 µm**

	Value	Error	F	Cx	Cy	K1	K2	K3	P1	P2
**F**	**5620.32**	0.051	1.00	0.02	0.01	-0.38	0.33	-0.29	-0.00	0.07
**Cx**	**93.2803**	0.061		1.00	-0.04	0.03	-0.01	0.01	0.82	0.06
**Cy**	**36.9105**	0.07			1.00	-0.00	0.00	-0.00	-0.02	0.78
**K1**	**-0.0120434**	6.4e-05				1.00	-0.96	0.91	0.05	0.00
**K2**	**0.0261633**	0.00032					1.00	-0.98	-0.05	-0.01
**K3**	**-0.022597**	0.00047						1.00	0.05	0.01
**P1**	**0.00274429**	3.6e-06							1.00	0.04
**P2**	**0.000822944**	4.3e-06								1.00

Table 2. Calibration coefficients and correlation matrix.

Camera Calibration

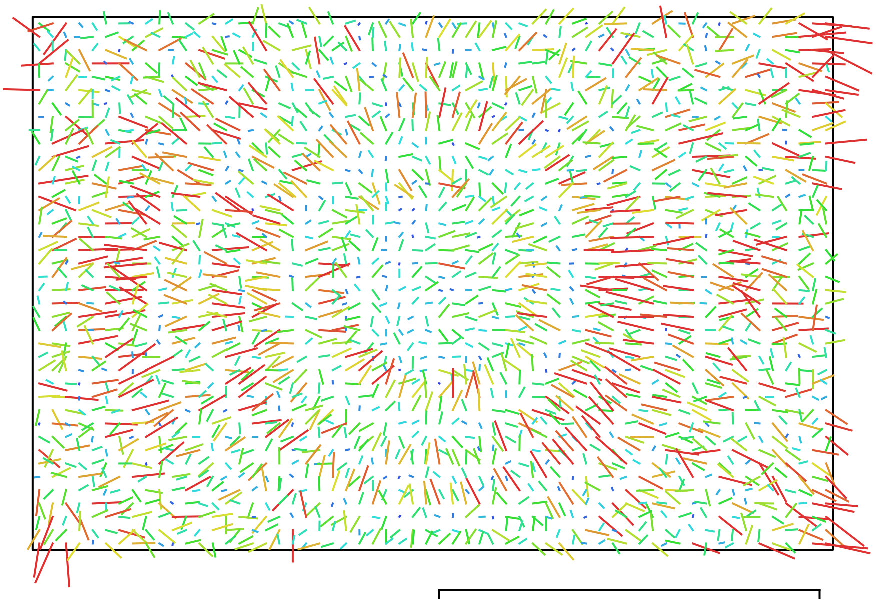

Fig. 3. Image residuals for NX500 (20 mm).

NX500 (20 mm)

462 images

Type	Resolution	Focal Length	Pixel Size
**Frame**	**6480 x 4320**	**20 mm**	**3.7 x 3.7 μm**

	Value	Error	F	Cx	Cy	K1	K2	K3	P1	P2
**F**	**5629.02**	0.042	1.00	-0.16	-0.12	-0.34	0.31	-0.28	-0.04	-0.02
**Cx**	**71.5273**	0.042		1.00	0.06	0.03	-0.03	0.03	0.88	0.02
**Cy**	**44.2309**	0.035			1.00	-0.00	-0.02	0.02	0.06	0.78
**K1**	**-0.0117203**	4.7e-05				1.00	-0.97	0.91	0.03	0.01
**K2**	**0.0267752**	0.00024					1.00	-0.98	-0.04	-0.03
**K3**	**-0.0243889**	0.00035						1.00	0.04	0.03
**P1**	**0.00226701**	2.6e-06							1.00	0.03
**P2**	**0.00118379**	2e-06								1.00

Table 3. Calibration coefficients and correlation matrix.

Camera Calibration

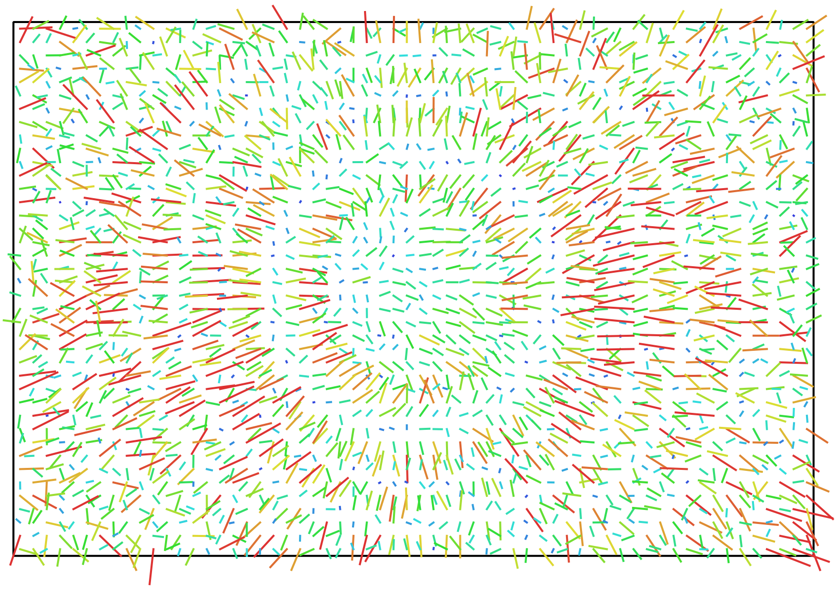

Fig. 4. Image residuals for NX500 (20 mm).

NX500 (20 mm)

530 images

Type	Resolution	Focal Length	Pixel Size
**Frame**	**6480 x 4320**	**20 mm**	**3.7 x 3.7 µm**

	**Value**	**Error**	**F**	**Cx**	**Cy**	**K1**	**K2**	**K3**	**P1**	**P2**
**F**	**5628.61**	0.044	1.00	-0.03	-0.13	-0.26	0.25	-0.22	-0.00	-0.02
**Cx**	**84.1**	0.039		1.00	-0.02	0.01	-0.01	0.01	0.83	0.00
**Cy**	**35.1838**	0.03			1.00	0.01	-0.02	0.01	-0.01	0.68
**K1**	**-0.0120013**	4.1e-05				1.00	-0.96	0.91	0.02	0.01
**K2**	**0.0305168**	0.00021					1.00	-0.98	-0.02	-0.02
**K3**	**-0.0319377**	0.00033						1.00	0.03	0.02
**P1**	**0.0025394**	2.3e-06							1.00	0.02
**P2**	**0.000927813**	1.7e-06								1.00

Table 4. Calibration coefficients and correlation matrix.

Camera Calibration

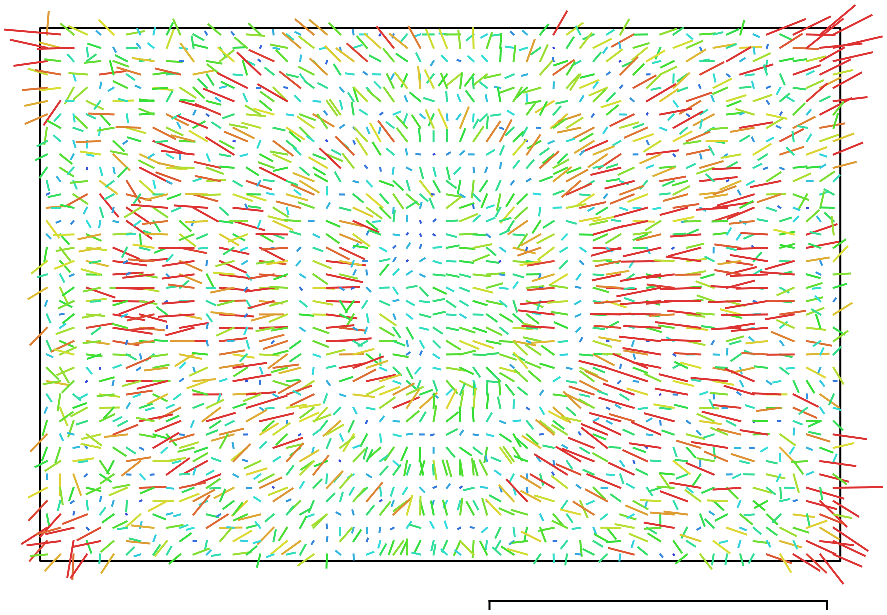

Fig. 5. Image residuals for NX500 (20 mm).

NX500 (20 mm)

513 images

Type	Resolution	Focal Length	Pixel Size
**Frame**	**6480 x 4320**	**20 mm**	**3.7 x 3.7 µm**

	Value	Error	F	Cx	Cy	K1	K2	K3	P1	P2
F	**5624.08**	0.053	1.00	-0.08	-0.08	-0.21	0.20	-0.18	0.00	-0.03
Cx	**84.0031**	0.035		1.00	-0.01	0.01	-0.01	0.02	0.80	-0.01
Cy	**59.997**	0.028			1.00	0.01	-0.02	0.02	-0.02	0.72
K1	**-0.010597**	3.6e-05				1.00	-0.96	0.90	0.03	0.01
K2	**0.0213585**	0.00019					1.00	-0.98	-0.03	-0.01
K3	**-0.0147773**	0.00029						1.00	0.04	0.01
P1	**0.00251084**	2.1e-06							1.00	-0.02
P2	**0.00151258**	1.7e-06								1.00

Table 5. Calibration coefficients and correlation matrix.

Camera Calibration

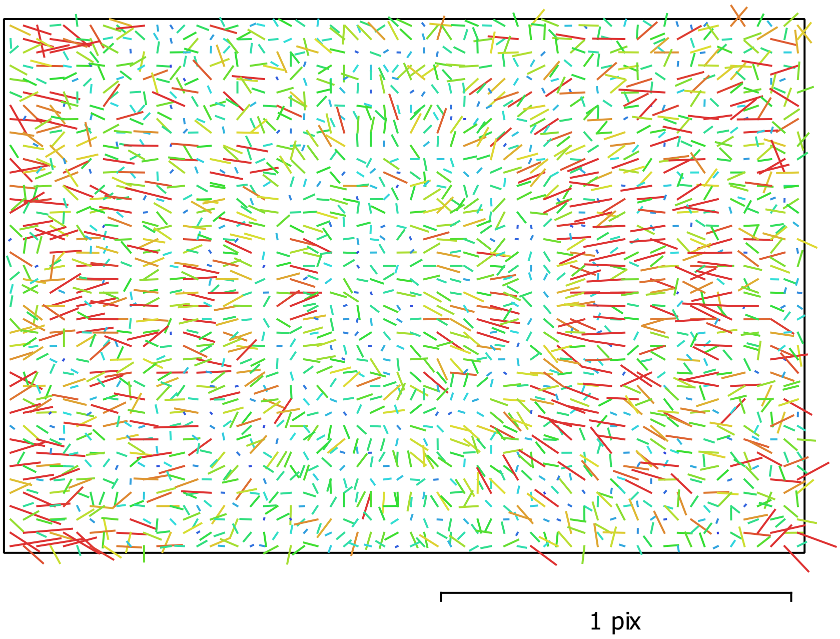

Fig. 6. Image residuals for NX500 (20 mm).

NX500 (20 mm)

412 images

Type	Resolution	Focal Length	Pixel Size
**Frame**	**6480 x 4320**	**20 mm**	**3.7 x 3.7 μm**

	Value	Error	F	Cx	Cy	K1	K2	K3	P1	P2
F	**5626.67**	0.046	1.00	0.04	-0.10	-0.37	0.35	-0.32	0.03	-0.01
Cx	**88.9033**	0.049		1.00	0.05	-0.01	0.01	0.00	0.87	0.04
Cy	**45.4841**	0.039			1.00	-0.02	0.03	-0.04	0.05	0.74
K1	**-0.0126985**	5.4e-05				1.00	-0.97	0.91	0.01	-0.01
K2	**0.0313749**	0.00027					1.00	-0.98	-0.01	0.00
K3	**-0.0331474**	0.00041						1.00	0.02	-0.01
P1	**0.00261709**	3e-06							1.00	0.05
P2	**0.00113324**	2.2e-06								1.00

Table 6. Calibration coefficients and correlation matrix.

Camera Calibration

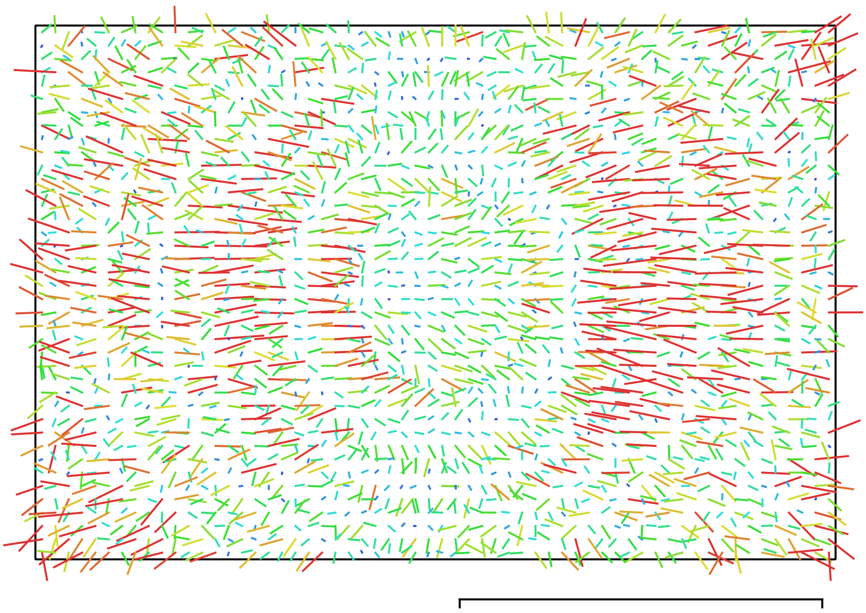

Fig. 7. Image residuals for NX500 (20 mm).

NX500 (20 mm)

478 images

Type	Resolution	Focal Length	Pixel Size
**Frame**	**6480 x 4320**	**20 mm**	**3.7 x 3.7 μm**

	Value	Error	F	Cx	Cy	K1	K2	K3	P1	P2
**F**	**5627.28**	0.032	1.00	-0.00	-0.00	-0.44	0.39	-0.34	-0.01	0.02
**Cx**	**68.9253**	0.041		1.00	-0.01	0.02	-0.01	0.01	0.87	-0.03
**Cy**	**48.1518**	0.036			1.00	0.02	-0.03	0.03	-0.02	0.74
**K1**	**-0.0125418**	4.8e-05				1.00	-0.97	0.91	0.02	0.01
**K2**	**0.0353351**	0.00025					1.00	-0.98	-0.01	-0.01
**K3**	**-0.0392209**	0.00037						1.00	0.01	0.02
**P1**	**0.00201727**	2.6e-06							1.00	-0.03
**P2**	**0.00125103**	2.1e-06								1.00

Table 7. Calibration coefficients and correlation matrix.

Ground Control Points

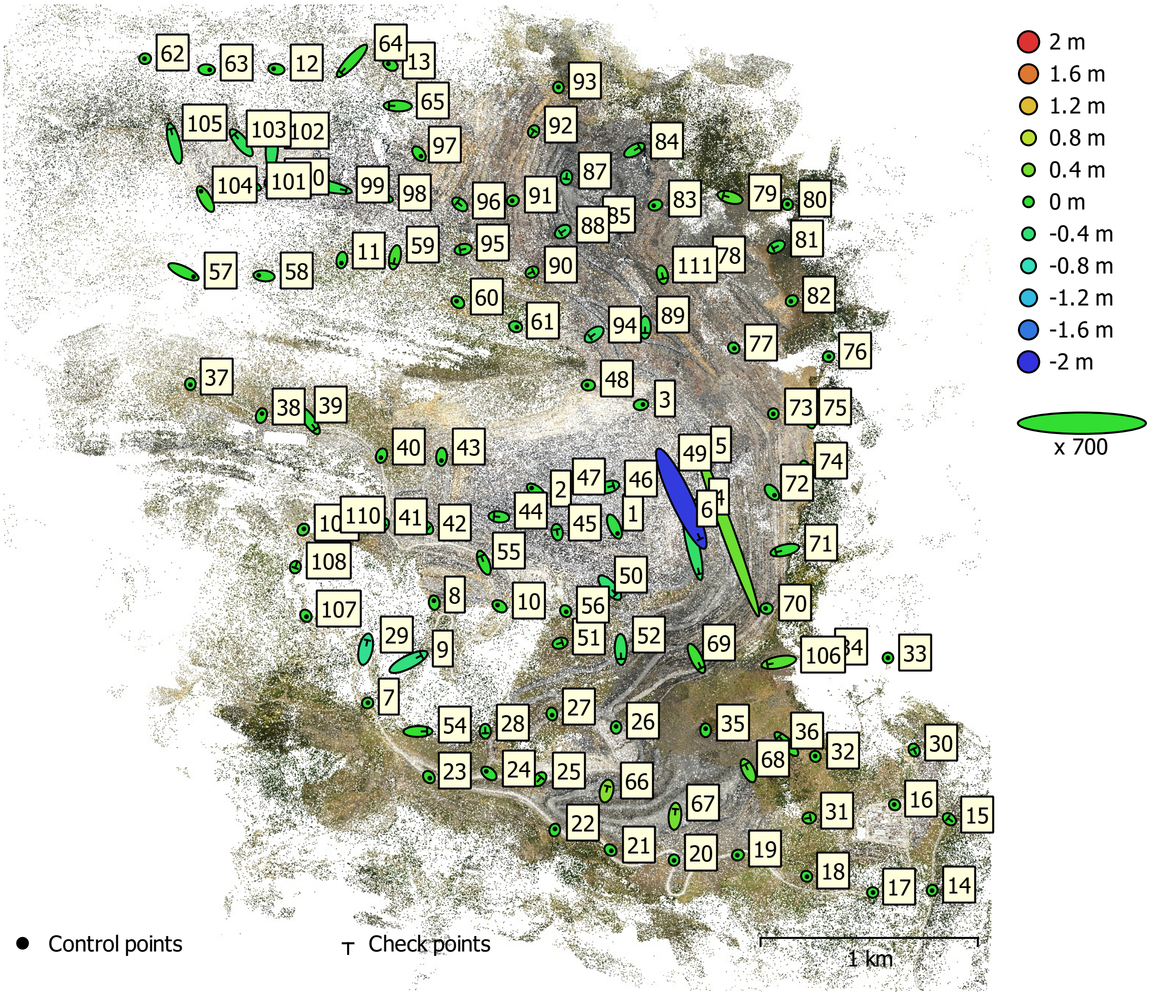

Fig. 8. GCP locations and error estimates.
Z error is represented by ellipse color. X,Y errors are represented by ellipse shape.
Estimated GCP locations are marked with a dot or crossing.

Count	X error (cm)	Y error (cm)	Z error (cm)	XY error (cm)	Total (cm)
55	2.79457	2.87485	2.72269	4.00929	4.84638

Table 8. Control points RMSE.
X - Easting, Y - Northing, Z - Altitude.

Count	X error (cm)	Y error (cm)	Z error (cm)	XY error (cm)	Total (cm)
54	10.1522	18.2795	32.4148	20.9095	38.5736

Table 9. Check points RMSE.
X - Easting, Y - Northing, Z - Altitude.

Label	X error (cm)	Y error (cm)	Z error (cm)	Total (cm)	Image (pix)
1	4.12144	-9.01514	-10.7416	14.6164	0.502 (104)
2	-5.90571	4.56682	-0.0463886	7.46562	0.484 (109)
3	2.62836	0.345249	-0.291764	2.66694	0.180 (51)
4	2.01715	9.22822	14.833	17.5854	0.637 (50)
7	0.496987	0.264356	0.0210259	0.563314	0.084 (24)
8	-0.395573	2.56743	-0.139083	2.60145	0.349 (32)
10	-2.649	1.64301	2.25454	3.84703	0.499 (42)
11	-0.644592	-3.87733	-0.689135	3.9905	0.261 (36)
12	-3.67604	0.570212	0.398498	3.74128	0.407 (26)
13	-2.76297	1.42099	0.460372	3.14088	0.179 (20)
14	-0.183155	-0.371481	0.0489498	0.417061	0.071 (23)
16	-0.549979	0.416809	0.0867944	0.695514	0.088 (34)
17	0.437018	-0.0221596	-0.0614082	0.441868	0.070 (23)
18	0.463298	-0.419505	-0.0574307	0.627637	0.111 (25)
19	-0.879316	-0.248936	-0.0933079	0.918625	0.103 (20)
20	-0.0295037	-0.459851	-0.0207921	0.461265	0.120 (16)
21	1.16063	-0.958763	0.177204	1.51581	0.133 (15)
22	0.349684	1.43434	-0.0382667	1.47684	0.119 (13)
23	1.13821	-1.34515	0.142655	1.76785	0.133 (18)
24	-3.5074	2.67382	-0.515995	4.44043	0.330 (27)
26	0.024583	1.31688	-0.560148	1.43128	0.126 (33)
27	0.361921	-1.4326	0.0760913	1.47957	0.197 (27)
32	-0.228952	0.379151	-0.0128981	0.443104	0.067 (18)
33	0.0111542	0.00102501	-0.00906342	0.0144088	0.001 (3)
34	-0.0147689	0.0663291	-0.0259355	0.0727346	0.014 (4)
35	0.0296018	2.05144	-0.43458	2.09717	0.257 (11)
37	0.0319624	-0.887695	0.289111	0.934136	0.119 (46)
38	0.883092	2.73418	-0.527374	2.92126	0.179 (57)
40	-0.8735	-2.61626	-1.30076	3.04956	0.272 (66)
43	-0.26417	-4.91187	0.301554	4.9282	0.490 (69)
48	-1.81296	0.0481858	-0.545448	1.89385	0.186 (44)

Label	X error (cm)	Y error (cm)	Z error (cm)	Total (cm)	Image (pix)
56	0.718351	-0.961548	-0.0580018	1.20165	0.308 (50)
57	13.3353	-6.36749	-1.55034	14.8586	1.479 (14)
58	-6.95956	0.768291	-1.20443	7.10467	0.872 (30)
60	-1.71125	1.38358	0.130867	2.20449	0.157 (32)
61	1.64414	-0.59891	0.599972	1.84982	0.131 (20)
62	-0.812843	-0.0839013	0.226495	0.84797	0.146 (17)
63	3.94622	-0.221898	-0.275948	3.96208	0.308 (16)
70	-1.20842	0.158116	-0.847712	1.48455	0.101 (30)
72	3.28149	-3.75446	-3.23552	5.94414	0.270 (18)
73	-0.10994	-0.196951	0.200121	0.301537	0.062 (15)
76	0.59666	0.148038	-0.0197594	0.615068	0.092 (9)
77	0.715703	-0.529663	0.0457226	0.891551	0.087 (11)
78	0.39499	-1.50734	-0.221522	1.5739	0.116 (11)
80	0.00619658	0.320701	0.0257798	0.321795	0.088 (7)
82	-1.20233	-0.665849	-0.269834	1.40063	0.203 (6)
83	-2.1417	-0.65286	-0.138849	2.2433	0.199 (13)
85	0.375376	0.0323385	-0.359101	0.520487	0.202 (10)
91	-0.905673	-0.266509	-0.11543	0.951102	0.192 (20)
93	-0.06051	0.602514	-0.0973309	0.613317	0.168 (16)
97	2.60279	-3.15902	0.256064	4.10115	0.362 (40)
100	1.96812	0.406167	-3.35113	3.9075	0.823 (28)
104	-5.92295	10.2672	5.55271	13.0893	1.068 (21)
107	0.814953	-1.19168	-0.454595	1.51358	0.102 (21)
109	0.857503	0.909087	2.18312	2.51551	0.263 (22)
**Total**	**2.79457**	**2.87485**	**2.72269**	**4.84638**	**0.395**

Table 10. Control points.
X - Easting, Y - Northing, Z - Altitude.

Label	X error (cm)	Y error (cm)	Z error (cm)	Total (cm)	Image (pix)
5	-38.2126	105.232	33.139	116.757	0.577 (38)
6	8.73609	-38.4912	-31.5713	50.5434	0.270 (66)
9	17.1379	8.59612	-43.3674	47.4166	0.244 (29)

Label	X error (cm)	Y error (cm)	Z error (cm)	Total (cm)	Image (pix)
15	-1.95269	1.48705	1.47183	2.86192	0.114 (27)
25	2.13303	2.42335	6.893	7.61156	0.101 (22)
28	0.0768812	-2.54644	-7.35507	7.78379	0.162 (29)
29	2.4658	11.8081	-54.446	55.7663	0.028 (18)
30	-0.770997	1.61156	-0.126239	1.79095	0.101 (20)
31	1.59819	0.264875	8.18681	8.34555	0.131 (26)
36	-9.7378	9.8687	3.9725	14.4221	0.133 (14)
39	8.35047	-11.6319	-6.39491	15.682	0.206 (40)
41	1.259	1.92631	-10.1622	10.4195	0.397 (56)
42	2.55978	-2.2712	-11.2146	11.7251	0.372 (48)
44	-6.49557	0.673852	-5.72743	8.6862	0.604 (73)
45	-0.634153	3.60479	-8.90221	9.62527	0.556 (94)
46	6.52554	1.90087	-17.5575	18.8272	0.409 (63)
47	2.84602	5.70533	-15.1492	16.4362	0.478 (107)
49	24.8892	-53.1405	-194.616	203.27	0.772 (59)
50	8.17414	-8.91806	-36.652	38.5968	0.425 (56)
51	2.97652	0.909976	-2.25466	3.84334	0.350 (42)
52	0.338519	-12.5515	-24.6136	27.6312	0.308 (56)
54	11.9837	0.222455	1.23729	12.0495	0.118 (31)
55	-3.72285	8.97964	-1.08278	9.78089	0.343 (51)
59	-1.86615	-8.68057	9.77441	13.2051	0.255 (9)
64	-14.393	-15.5285	4.04338	21.5555	0.289 (13)
65	-11.4816	0.318299	3.00505	11.8726	0.346 (31)
66	1.4699	6.26322	49.6275	50.0428	0.175 (22)
67	0.71389	9.50052	37.2026	38.4032	0.161 (12)
68	-4.00387	8.11084	17.1574	19.3957	0.135 (16)
69	6.2889	-12.6079	6.88251	15.6805	0.213 (28)
71	-11.8229	-2.83514	-4.67867	13.0273	0.226 (24)
74	2.69647	-6.50493	6.41104	9.52295	0.100 (10)
75	-2.01099	6.58545	2.6264	7.36954	0.024 (6)
79	-9.03216	2.5389	17.6295	19.9706	0.139 (8)
81	-4.27636	-2.15926	-10.2526	11.3166	0.233 (6)

Label	X error (cm)	Y error (cm)	Z error (cm)	Total (cm)	Image (pix)
84	6.8567	3.82599	-3.30869	8.52056	0.173 (12)
87	-0.217425	-1.85143	-19.5623	19.6509	0.237 (10)
88	-2.97678	-1.90881	-21.9991	22.2815	0.240 (13)
89	-0.086625	-7.32853	-16.4796	18.0358	0.160 (27)
90	1.66329	0.810565	3.73165	4.16518	0.150 (28)
92	0.67005	1.0896	2.34178	2.66835	0.223 (18)
94	-5.13061	-3.73529	-27.5724	28.2934	0.148 (35)
95	-4.16439	-0.813691	-2.19468	4.77712	0.249 (38)
96	-3.38828	2.70787	-4.8497	6.50635	0.198 (23)
98	16.6848	-3.61213	-1.359	17.1254	0.574 (32)
99	30.3132	-6.76983	-14.169	34.1391	0.575 (30)
101	-17.1256	7.76144	-2.48994	18.9665	0.668 (26)
102	0.85686	17.0746	-26.4136	31.4635	0.365 (21)
103	-9.04993	11.153	-20.6709	25.171	0.478 (20)
105	-4.83549	19.1613	-17.0222	26.0825	0.199 (24)
106	-15.1574	-2.98609	20.2575	25.4761	0.085 (13)
108	-0.399109	-1.07645	4.08345	4.24177	0.178 (19)
110	-2.24166	3.16	-6.90152	7.91465	0.262 (28)
111	1.26685	-5.18609	3.0283	6.13768	0.140 (14)
**Total**	**10.1522**	**18.2795**	**32.4148**	**38.5736**	**0.387**

Table 11. Check points.

X - Easting, Y - Northing, Z - Altitude.

Digital Elevation Model

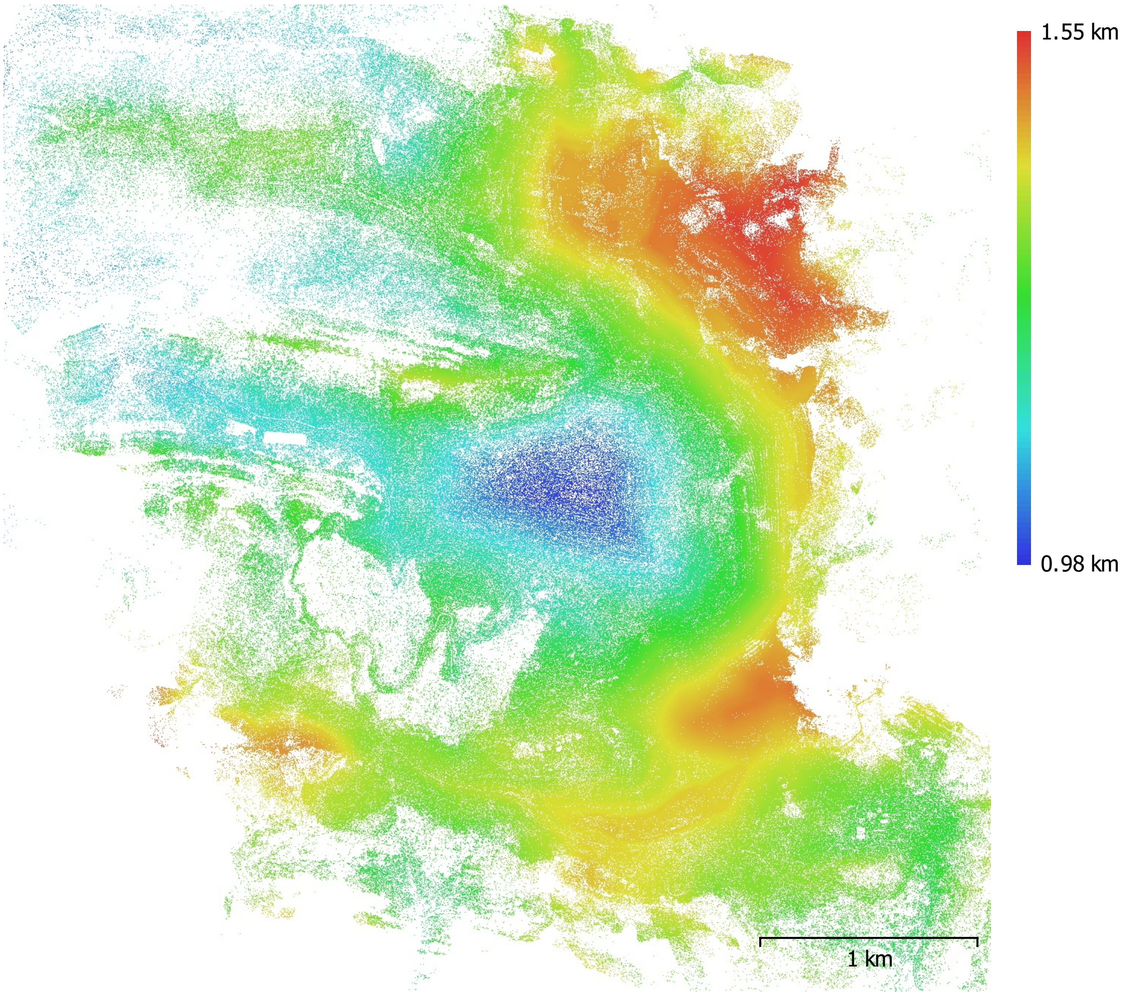

Fig. 9. Reconstructed digital elevation model.

Resolution:	unknown
Point density:	unknown

Processing Parameters

**General**	
Cameras	2595
Aligned cameras	2577
Markers	110
**Shapes**	
Polygon	1
Coordinate system	ETRS89 / UTM zone 30N (EPSG::25830)
Rotation angles	Yaw, Pitch, Roll
**Tie Points**	
Points	1,755,989 of 12,529,745
RMS reprojection error	0.139188 (0.327499 pix)
Max reprojection error	0.299902 (1.64763 pix)
Mean key point size	2.31288 pix
Point colors	3 bands, uint8
Key points	No
Average tie point multiplicity	3.65511
**Alignment parameters**	
Accuracy	High
Generic preselection	Yes
Reference preselection	No
Key point limit	60,000
Key point limit per Mpx	1,000
Tie point limit	0
Exclude stationary tie points	Yes
Guided image matching	No
Adaptive camera model fitting	No
Matching time	4 hours 7 minutes
Matching memory usage	3.73 GB
Alignment time	2 hours 17 minutes
Alignment memory usage	4.82 GB
**Optimization parameters**	
Parameters	f, cx, cy, k1-k3, p1, p2
Adaptive camera model fitting	No
Optimization time	34 seconds
Date created	2023:11:13 15:04:46
Software version	2.0.0.15597
File size	775.20 MB
**System**	
Software name	Agisoft Metashape Professional
Software version	2.0.3 build 16960
OS	Windows 64 bit
RAM	63.90 GB
CPU	Intel(R) Core(TM) i7-7700 CPU @ 3.60GHz
GPU(s)	Quadro M4000
