# Supplementary material for: Automated cleaning of tie point clouds following USGS guidelines in Agisoft Metashape professional (ver. 2.1.0)
Source: MethodsX. 2024 Mar 26;12:102679. doi: 10.1016/j.mex.2024.102679 (PMC10992719; doi:10.1016/j.mex.2024.102679)
Supplement: Supplementary file 3 — The supplementary material includes supplementary text, figures and the processing reports generated by the software. [file mmc3.zip › Lucia_SCC-Optimized_r3.pdf]

Lucia_SCC-Optimized_r3

Automatically cleaned sparse cloud using the SCC script (optimized settings). UAS data provided by Sanz-Ablanedo et al. (2018).

Sanz-Ablanedo, E., Chandler, J. H., Rodríguez-Pérez, J. R., and Ordóñez, C.: Accuracy of Unmanned Aerial Vehicle (UAV) and SfM Photogrammetry Survey as a Function of the Number and Location of Ground Control Points Used, Remote Sensing, 10, 1606, 2018.

28 December 2023

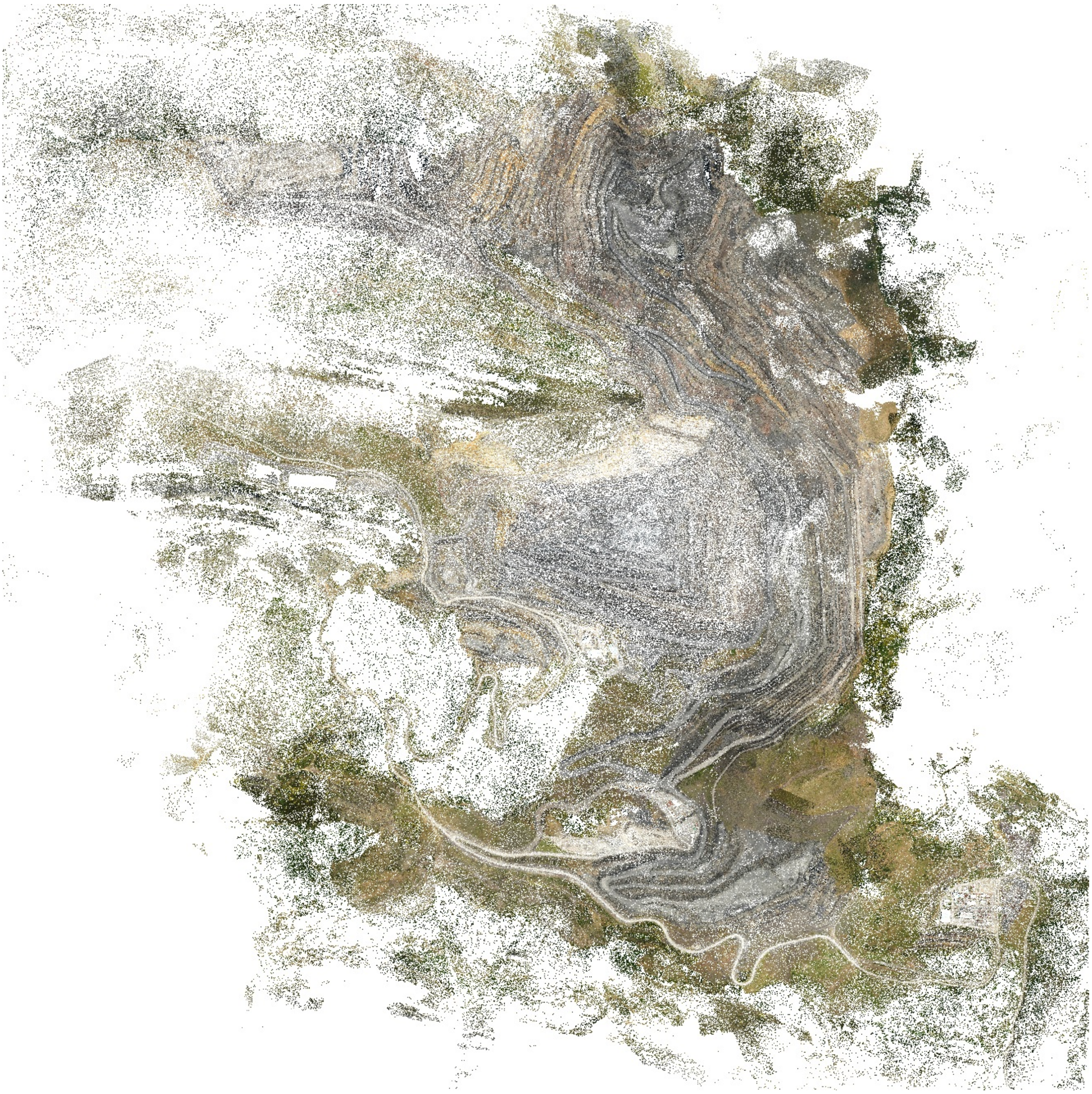

Survey Data

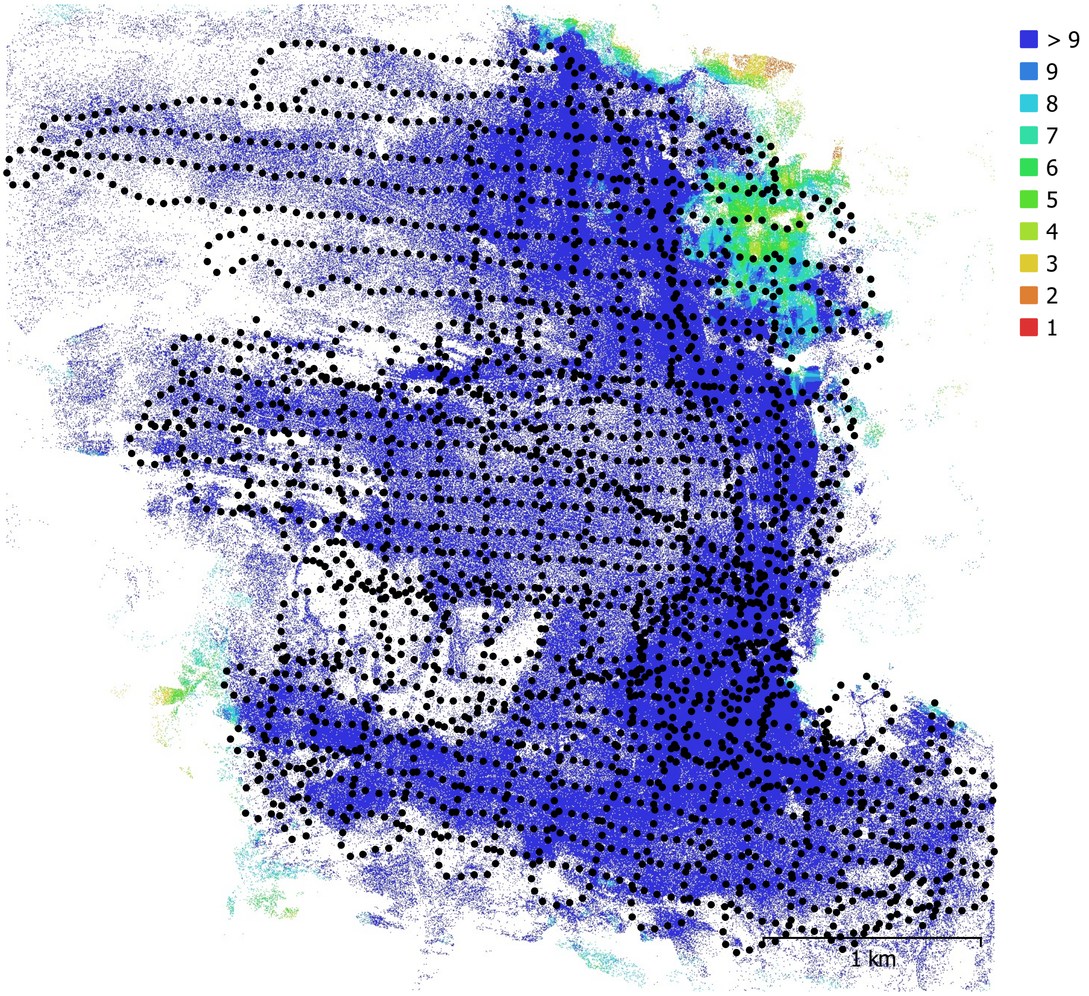

Fig. 1. Camera locations and image overlap.

Number of images:	2,595	Camera stations:	2,577
Flying altitude:	349 m	Tie points:	1,760,863
Ground resolution:	6.2 cm/pix	Projections:	4,164,465
Coverage area:	7.51 km²	Reprojection error:	0.328 pix

Camera Model	Resolution	Focal Length	Pixel Size	Precalibrated
NX500 (20 mm)	6480 x 4320	20 mm	3.7 x 3.7 μm	No
NX500 (20 mm)	6480 x 4320	20 mm	3.7 x 3.7 μm	No
NX500 (20 mm)	6480 x 4320	20 mm	3.7 x 3.7 μm	No
NX500 (20 mm)	6480 x 4320	20 mm	3.7 x 3.7 μm	No
NX500 (20 mm)	6480 x 4320	20 mm	3.7 x 3.7 μm	No

Camera Model	Resolution	Focal Length	Pixel Size	Precalibrated
NX500 (20 mm)	6480 x 4320	20 mm	3.7 x 3.7 µm	No

Table 1. Cameras.

Camera Calibration

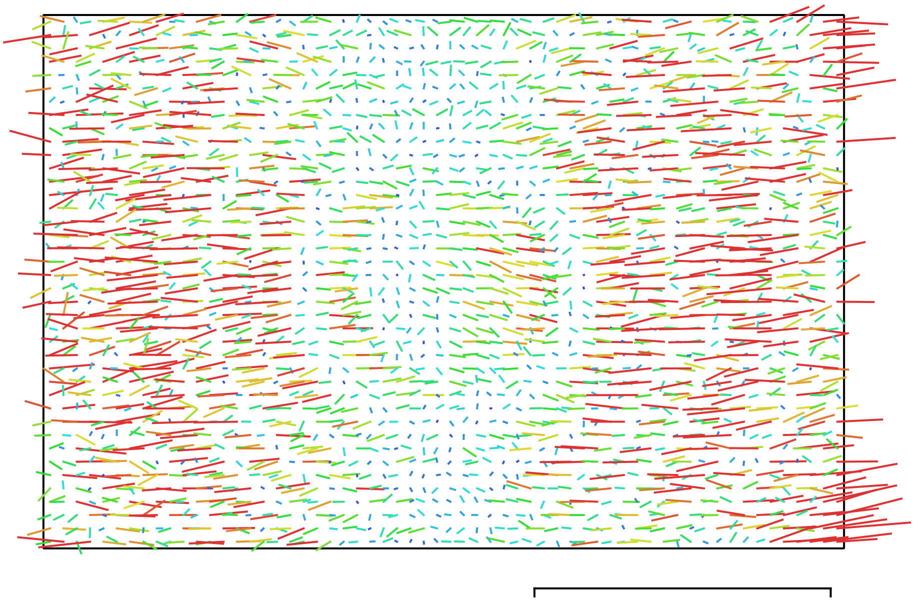

Fig. 2. Image residuals for NX500 (20 mm).

NX500 (20 mm)

200 images

Type	Resolution	Focal Length	Pixel Size
**Frame**	**6480 x 4320**	**20 mm**	**3.7 x 3.7 µm**

	Value	Error	F	Cx	Cy	K1	K2	K3	P1	P2
**F**	**5620.5**	0.051	1.00	0.02	0.01	-0.38	0.33	-0.30	-0.00	0.07
**Cx**	**93.407**	0.061		1.00	-0.04	0.03	-0.02	0.01	0.82	0.06
**Cy**	**36.8086**	0.069			1.00	-0.00	0.00	-0.01	-0.02	0.78
**K1**	**-0.0120445**	6.4e-05				1.00	-0.96	0.91	0.05	0.00
**K2**	**0.026286**	0.00032					1.00	-0.98	-0.05	-0.01
**K3**	**-0.0228416**	0.00047						1.00	0.05	0.01
**P1**	**0.00274933**	3.6e-06							1.00	0.04
**P2**	**0.000819064**	4.3e-06								1.00

Table 2. Calibration coefficients and correlation matrix.

Camera Calibration

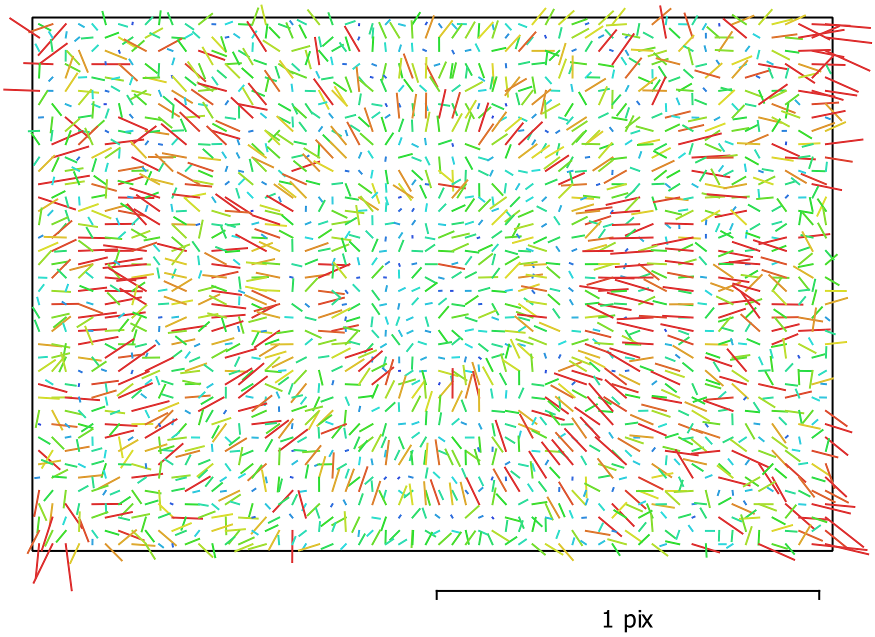

Fig. 3. Image residuals for NX500 (20 mm).

NX500 (20 mm)

462 images

Type	Resolution	Focal Length	Pixel Size
**Frame**	**6480 x 4320**	**20 mm**	**3.7 x 3.7 µm**

	Value	Error	F	Cx	Cy	K1	K2	K3	P1	P2
F	**5629.06**	0.041	1.00	-0.16	-0.12	-0.34	0.31	-0.28	-0.04	-0.02
Cx	**71.6303**	0.041		1.00	0.06	0.03	-0.03	0.03	0.88	0.02
Cy	**44.2981**	0.035			1.00	-0.00	-0.02	0.02	0.05	0.78
K1	**-0.0117469**	4.7e-05				1.00	-0.97	0.91	0.03	0.01
K2	**0.0269842**	0.00024					1.00	-0.98	-0.04	-0.03
K3	**-0.0247542**	0.00035						1.00	0.04	0.03
P1	**0.00227301**	2.6e-06							1.00	0.02
P2	**0.00118757**	2e-06								1.00

Table 3. Calibration coefficients and correlation matrix.

Camera Calibration

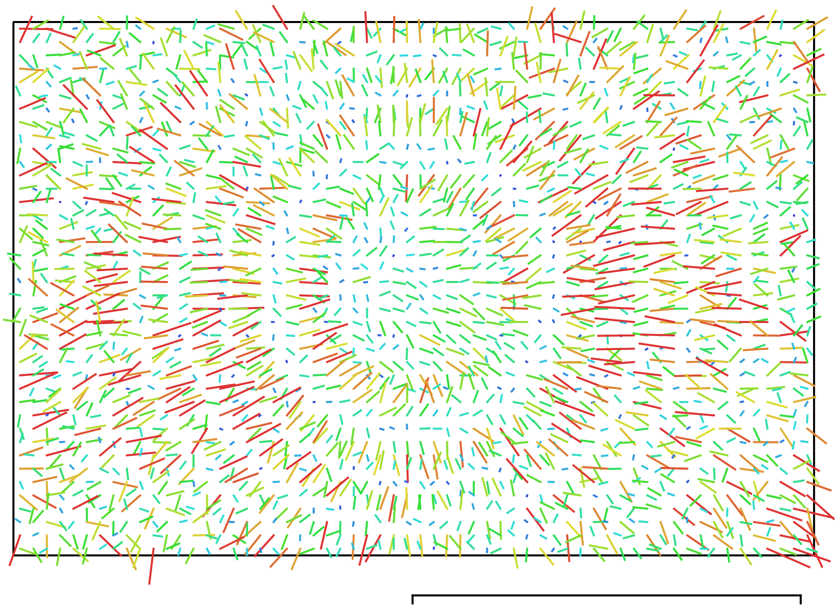

Fig. 4. Image residuals for NX500 (20 mm).

NX500 (20 mm)

530 images

Type	Resolution	Focal Length	Pixel Size
**Frame**	**6480 x 4320**	**20 mm**	**3.7 x 3.7 μm**

	Value	Error	F	Cx	Cy	K1	K2	K3	P1	P2
**F**	**5628.56**	0.044	1.00	-0.03	-0.13	-0.26	0.25	-0.22	-0.00	-0.02
**Cx**	**84.1366**	0.039		1.00	-0.02	0.01	-0.01	0.01	0.83	0.00
**Cy**	**35.1933**	0.03			1.00	0.01	-0.02	0.01	-0.01	0.68
**K1**	**-0.0120058**	4.1e-05				1.00	-0.96	0.91	0.02	0.01
**K2**	**0.0305093**	0.00021					1.00	-0.98	-0.02	-0.02
**K3**	**-0.031891**	0.00033						1.00	0.03	0.02
**P1**	**0.00254015**	2.3e-06							1.00	0.02
**P2**	**0.000928363**	1.7e-06								1.00

Table 4. Calibration coefficients and correlation matrix.

Camera Calibration

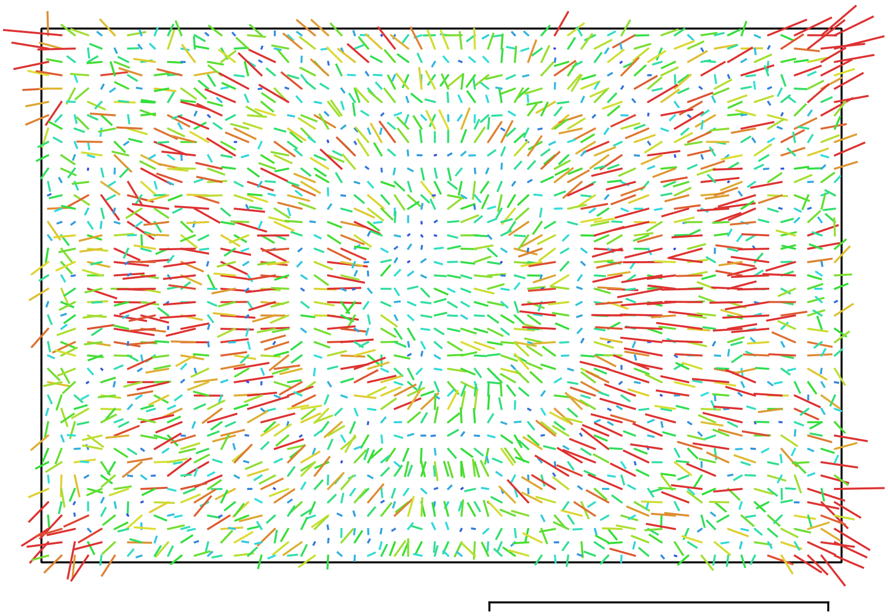

Fig. 5. Image residuals for NX500 (20 mm).

NX500 (20 mm)

513 images

Type	Resolution	Focal Length	Pixel Size
**Frame**	**6480 x 4320**	**20 mm**	**3.7 x 3.7 µm**

	Value	Error	F	Cx	Cy	K1	K2	K3	P1	P2
**F**	**5624.19**	0.053	1.00	-0.08	-0.08	-0.20	0.20	-0.18	0.00	-0.03
**Cx**	**83.9929**	0.035		1.00	-0.01	0.01	-0.01	0.02	0.80	-0.01
**Cy**	**59.9349**	0.028			1.00	0.01	-0.02	0.03	-0.02	0.73
**K1**	**-0.010573**	3.6e-05				1.00	-0.96	0.90	0.03	0.01
**K2**	**0.0212451**	0.00019					1.00	-0.98	-0.03	-0.01
**K3**	**-0.014579**	0.00028						1.00	0.04	0.01
**P1**	**0.00251039**	2.1e-06							1.00	-0.02
**P2**	**0.00151235**	1.7e-06								1.00

Table 5. Calibration coefficients and correlation matrix.

Camera Calibration

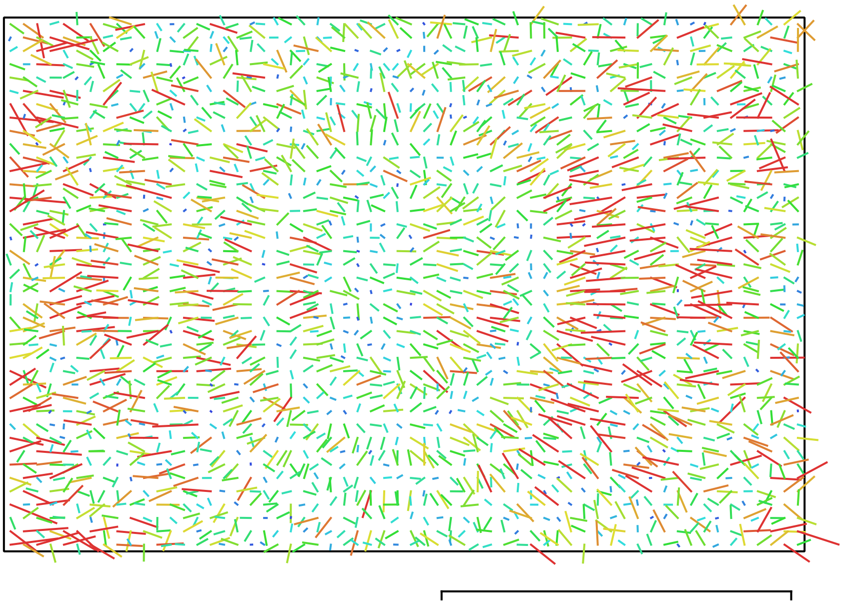

Fig. 6. Image residuals for NX500 (20 mm).

NX500 (20 mm)

412 images

Type	Resolution	Focal Length	Pixel Size
**Frame**	**6480 x 4320**	**20 mm**	**3.7 x 3.7 μm**

	Value	Error	F	Cx	Cy	K1	K2	K3	P1	P2
**F**	**5626.57**	0.046	1.00	0.05	-0.10	-0.37	0.34	-0.32	0.04	-0.01
**Cx**	**89.0181**	0.048		1.00	0.05	-0.01	0.01	0.00	0.87	0.04
**Cy**	**45.3986**	0.039			1.00	-0.02	0.03	-0.04	0.05	0.74
**K1**	**-0.0126415**	5.4e-05				1.00	-0.97	0.91	0.01	-0.01
**K2**	**0.0308217**	0.00027					1.00	-0.98	-0.01	0.00
**K3**	**-0.0320866**	0.00041						1.00	0.02	-0.01
**P1**	**0.00262056**	3e-06							1.00	0.05
**P2**	**0.00112948**	2.2e-06								1.00

Table 6. Calibration coefficients and correlation matrix.

Camera Calibration

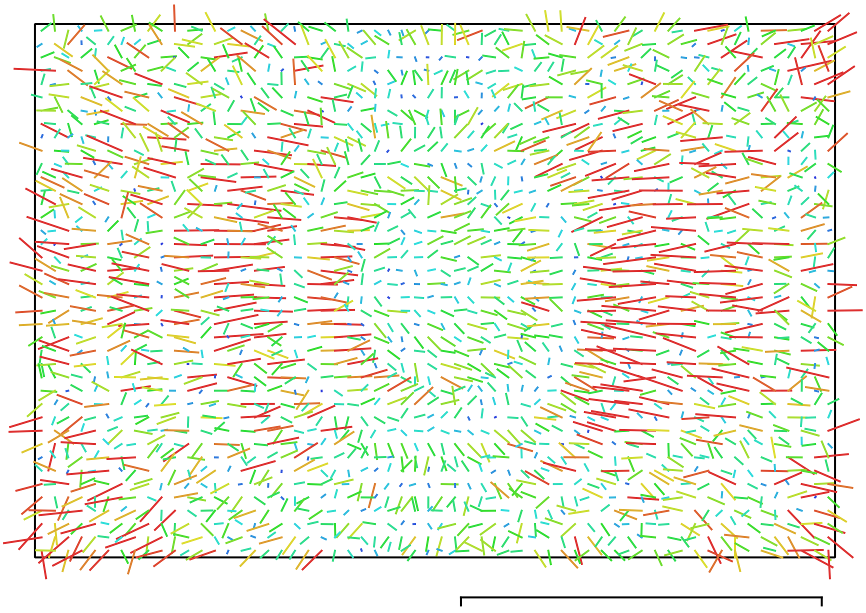

Fig. 7. Image residuals for NX500 (20 mm).

NX500 (20 mm)

478 images

Type	Resolution	Focal Length	Pixel Size
**Frame**	**6480 x 4320**	**20 mm**	**3.7 x 3.7 µm**

	Value	Error	F	Cx	Cy	K1	K2	K3	P1	P2
**F**	**5627.28**	0.032	1.00	-0.00	-0.00	-0.44	0.39	-0.34	-0.01	0.02
**Cx**	**68.8848**	0.041		1.00	-0.01	0.02	-0.01	0.01	0.87	-0.03
**Cy**	**48.2002**	0.036			1.00	0.02	-0.03	0.03	-0.02	0.74
**K1**	**-0.0125618**	4.8e-05				1.00	-0.97	0.91	0.02	0.01
**K2**	**0.0354216**	0.00025					1.00	-0.98	-0.01	-0.01
**K3**	**-0.0393292**	0.00037						1.00	0.01	0.02
**P1**	**0.0020157**	2.6e-06							1.00	-0.03
**P2**	**0.00125553**	2.1e-06								1.00

Table 7. Calibration coefficients and correlation matrix.

Ground Control Points

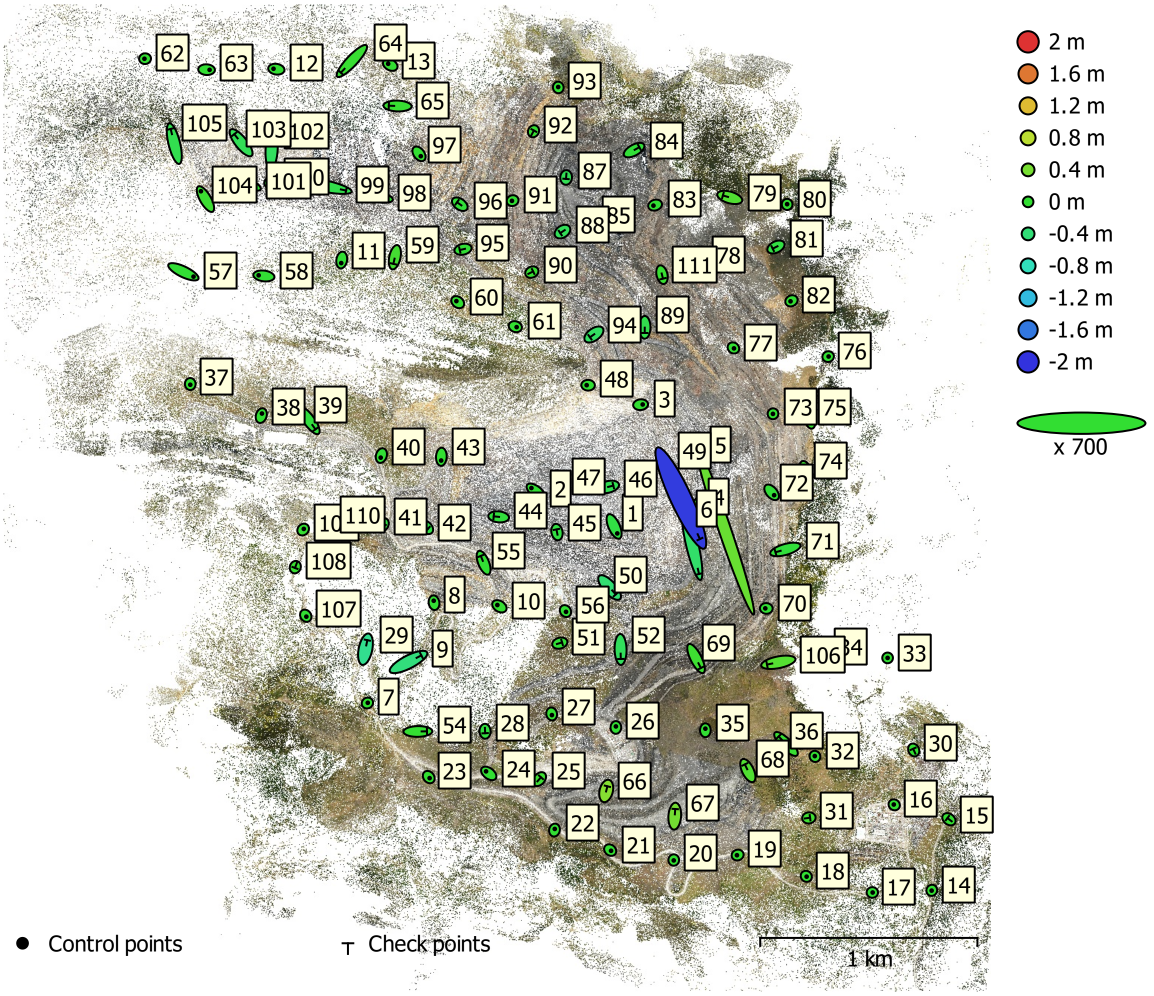

Fig. 8. GCP locations and error estimates.
Z error is represented by ellipse color. X,Y errors are represented by ellipse shape.
Estimated GCP locations are marked with a dot or crossing.

Count	X error (cm)	Y error (cm)	Z error (cm)	XY error (cm)	Total (cm)
55	2.7992	2.91713	2.75658	4.04292	4.89325

Table 8. Control points RMSE.
X - Easting, Y - Northing, Z - Altitude.

Count	X error (cm)	Y error (cm)	Z error (cm)	XY error (cm)	Total (cm)
54	9.94836	18.227	32.2781	20.7652	38.3806

Table 9. Check points RMSE.
X - Easting, Y - Northing, Z - Altitude.

Label	X error (cm)	Y error (cm)	Z error (cm)	Total (cm)	Image (pix)
1	4.18785	-9.18612	-10.9061	14.8615	0.504 (104)
2	-5.9115	4.49607	0.0157771	7.42702	0.486 (109)
3	2.74736	0.279026	-0.402107	2.79061	0.194 (51)
4	1.9146	9.6271	15.0248	17.9469	0.646 (50)
7	0.521209	0.302375	0.0305901	0.603345	0.087 (24)
8	-0.412596	2.61466	-0.146339	2.65106	0.350 (32)
10	-2.67559	1.68427	2.2625	3.88773	0.502 (42)
11	-0.651695	-4.01101	-0.724923	4.12776	0.264 (36)
12	-3.69003	0.567857	0.394994	3.75431	0.410 (26)
13	-2.75041	1.44041	0.456321	3.13811	0.180 (20)
14	-0.174141	-0.373015	0.0507828	0.414782	0.072 (23)
16	-0.556287	0.408509	0.0886008	0.695834	0.088 (34)
17	0.44226	-0.0172872	-0.0586759	0.44647	0.071 (23)
18	0.459421	-0.41119	-0.0548159	0.618991	0.112 (25)
19	-0.890152	-0.268966	-0.0924921	0.934488	0.103 (20)
20	-0.0275503	-0.505927	0.00641879	0.506717	0.121 (16)
21	1.17858	-0.936222	0.154944	1.51313	0.137 (15)
22	0.357915	1.47927	-0.0581963	1.52306	0.120 (13)
23	1.15282	-1.37731	0.143716	1.80184	0.134 (18)
24	-3.54223	2.67863	-0.506256	4.46976	0.332 (27)
26	0.0427496	1.3413	-0.576455	1.46055	0.127 (33)
27	0.331836	-1.49318	0.0768763	1.53154	0.207 (27)
32	-0.225629	0.38176	-0.0082381	0.443528	0.067 (18)
33	0.0108725	0.00172907	-0.00745323	0.0132948	0.001 (3)
34	-0.0133469	0.0678951	-0.0253691	0.0736985	0.014 (4)
35	0.0251453	2.06362	-0.42452	2.10699	0.258 (11)
37	0.0273278	-0.903928	0.286323	0.948585	0.119 (46)
38	0.884766	2.74202	-0.529162	2.92942	0.179 (57)
40	-0.859655	-2.68717	-1.328	3.11825	0.273 (66)
43	-0.251895	-4.90935	0.321515	4.92631	0.491 (69)
48	-1.88468	0.0514557	-0.543351	1.96211	0.187 (44)

Label	X error (cm)	Y error (cm)	Z error (cm)	Total (cm)	Image (pix)
56	0.730192	-1.01296	-0.0582688	1.25007	0.308 (50)
57	13.3529	-6.37856	-1.55311	14.8794	1.481 (14)
58	-6.86657	0.85596	-1.11917	7.00964	0.870 (30)
60	-1.75862	1.38473	0.141916	2.24285	0.160 (32)
61	1.72494	-0.620259	0.606763	1.93088	0.134 (20)
62	-0.811181	-0.0878496	0.22525	0.846446	0.147 (17)
63	3.95806	-0.2224	-0.279814	3.97416	0.311 (16)
70	-1.32206	0.0943972	-0.903098	1.60385	0.104 (30)
72	3.30167	-3.7804	-3.26479	5.9876	0.275 (18)
73	-0.0810609	-0.15384	0.223691	0.283329	0.062 (15)
76	0.594011	0.152093	-0.0232136	0.613612	0.092 (9)
77	0.761506	-0.561522	0.0693749	0.948689	0.086 (11)
78	0.411853	-1.51819	-0.202457	1.58604	0.116 (11)
80	-0.00211759	0.327903	0.0242725	0.328807	0.088 (7)
82	-1.21061	-0.660213	-0.268489	1.40483	0.204 (6)
83	-2.14517	-0.663896	-0.149475	2.25053	0.199 (13)
85	0.384869	0.0272463	-0.348587	0.519981	0.203 (10)
91	-0.82478	-0.316996	-0.0714553	0.886485	0.200 (20)
93	-0.112565	0.580662	-0.106202	0.600931	0.169 (16)
97	2.36619	-3.05097	0.217303	3.86711	0.363 (40)
100	2.07155	0.444469	-3.38672	3.99484	0.826 (28)
104	-5.95754	10.3169	5.58338	13.1569	1.069 (21)
107	0.805786	-1.2004	-0.465636	1.51891	0.102 (21)
109	0.861548	0.896459	2.18658	2.51536	0.264 (22)
**Total**	**2.7992**	**2.91713**	**2.75658**	**4.89325**	**0.397**

Table 10. Control points.

X - Easting, Y - Northing, Z - Altitude.

Label	X error (cm)	Y error (cm)	Z error (cm)	Total (cm)	Image (pix)
5	-35.1895	104.038	31.0184	114.124	0.611 (38)
6	9.10532	-39.3017	-31.7199	51.3195	0.282 (66)
9	16.8824	8.67719	-42.7233	46.7503	0.245 (29)

Label	X error (cm)	Y error (cm)	Z error (cm)	Total (cm)	Image (pix)
15	-1.97361	1.48634	1.76324	3.03536	0.115 (27)
25	2.13337	2.40547	6.19554	6.98013	0.103 (22)
28	0.111765	-2.53814	-7.23882	7.67171	0.163 (29)
29	2.45265	11.876	-54.3146	55.6518	0.029 (18)
30	-0.708737	1.56617	0.0397857	1.71953	0.101 (20)
31	1.55904	0.208538	8.14304	8.29357	0.132 (26)
36	-9.66992	9.8235	4.09028	14.3784	0.134 (14)
39	8.33047	-11.6722	-6.4042	15.7051	0.206 (40)
41	1.25723	1.9141	-10.0929	10.3495	0.399 (56)
42	2.58534	-2.28619	-11.0965	11.6208	0.374 (48)
44	-6.46191	0.657822	-5.73794	8.66677	0.605 (73)
45	-0.696763	3.49091	-8.86962	9.55731	0.557 (94)
46	6.57672	1.68524	-17.6279	18.8901	0.412 (63)
47	2.78921	5.51868	-15.0105	16.2342	0.481 (107)
49	25.09	-53.9604	-194.593	203.488	0.777 (59)
50	8.11947	-9.069	-36.4758	38.4533	0.426 (56)
51	2.98082	0.892189	-2.1417	3.77732	0.351 (42)
52	0.263986	-12.514	-24.2591	27.2979	0.310 (56)
54	11.9401	0.20259	1.19843	12.0018	0.119 (31)
55	-3.69261	8.97649	-1.05049	9.763	0.344 (51)
59	-1.92408	-8.74999	10.1745	13.5568	0.260 (9)
64	-14.448	-15.3442	3.17139	21.3131	0.290 (13)
65	-11.4548	0.374065	2.82361	11.8036	0.345 (31)
66	1.55017	6.14726	47.9535	48.3708	0.172 (22)
67	0.610877	9.41061	37.5074	38.6748	0.164 (12)
68	-3.94448	8.06651	17.4102	19.5893	0.136 (16)
69	6.33425	-12.4686	6.92744	15.607	0.214 (28)
71	-12.9814	-3.67962	-4.81708	14.3269	0.230 (24)
74	2.64532	-6.29509	6.85943	9.67872	0.098 (10)
75	-2.0136	6.7115	2.84125	7.56119	0.024 (6)
79	-9.01165	2.58397	17.7604	20.0828	0.139 (8)
81	-4.26884	-2.11269	-9.96622	11.0459	0.235 (6)

Label	X error (cm)	Y error (cm)	Z error (cm)	Total (cm)	Image (pix)
84	6.88746	3.743	-3.53369	8.5985	0.174 (12)
87	-0.158955	-1.91925	-19.5683	19.6628	0.239 (10)
88	-2.89292	-1.9111	-21.985	22.2567	0.243 (13)
89	-0.0183247	-7.40979	-15.5555	17.2302	0.163 (27)
90	1.69853	0.845343	3.32205	3.82565	0.152 (28)
92	0.512797	0.955561	2.33181	2.57165	0.224 (18)
94	-5.19431	-3.68768	-26.964	27.7062	0.151 (35)
95	-4.2522	-0.966911	-1.67963	4.67304	0.252 (38)
96	-3.61308	2.56577	-3.98802	5.9617	0.206 (23)
98	16.609	-3.18359	-1.63474	16.9902	0.577 (32)
99	29.9756	-6.26078	-14.4062	33.8418	0.580 (30)
101	-17.0117	7.83734	-2.45035	18.8898	0.669 (26)
102	0.964504	17.1722	-26.2702	31.3997	0.366 (21)
103	-8.9535	11.2358	-20.4794	25.0163	0.478 (20)
105	-4.75341	19.3041	-16.6669	25.9428	0.198 (24)
106	-14.9395	-2.9662	20.0616	25.1883	0.086 (13)
108	-0.40615	-1.06954	4.01333	4.17321	0.177 (19)
110	-2.2269	3.08509	-6.7231	7.72508	0.263 (28)
111	1.30067	-5.23746	3.52757	6.44721	0.140 (14)
**Total**	**9.94836**	**18.227**	**32.2781**	**38.3806**	**0.391**

Table 11. Check points.

X - Easting, Y - Northing, Z - Altitude.

Digital Elevation Model

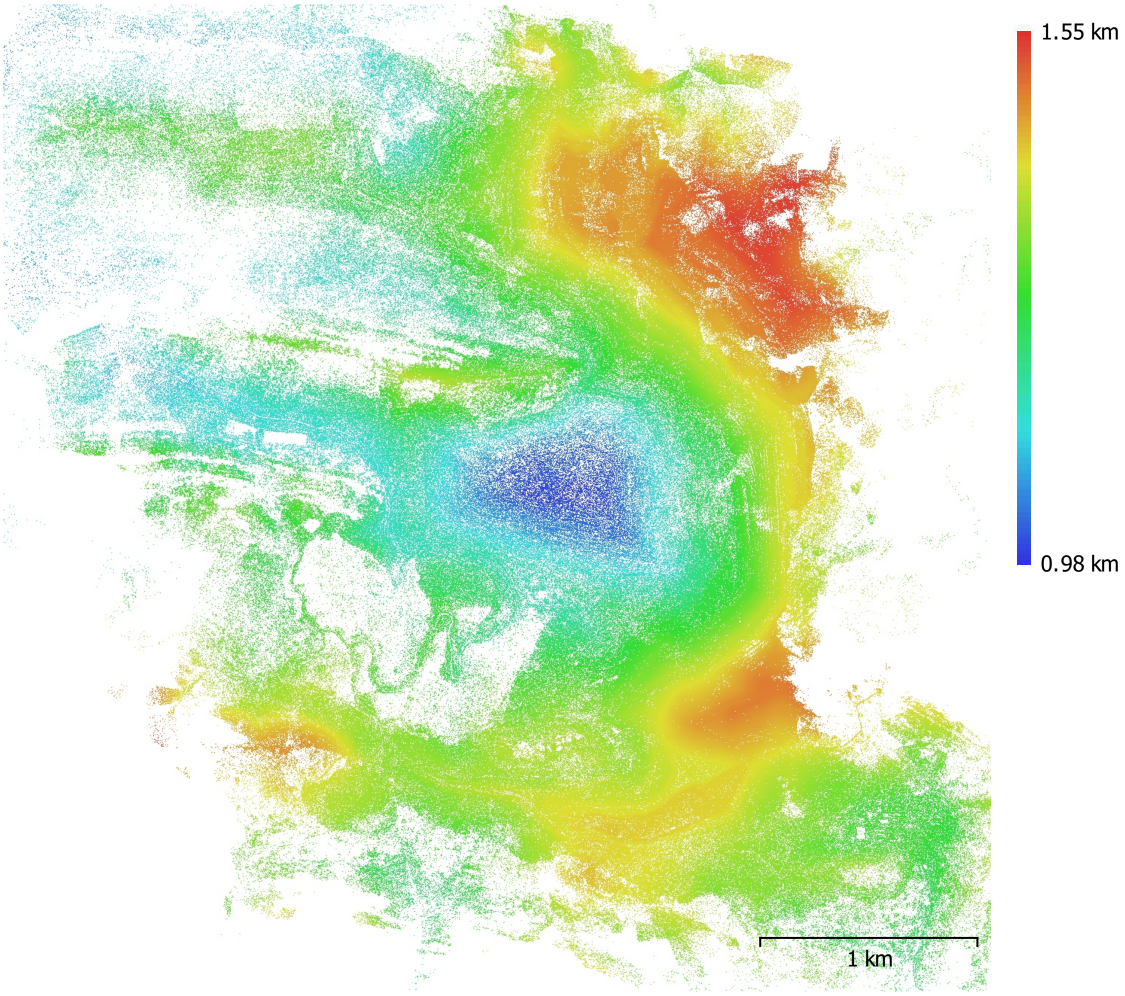

Fig. 9. Reconstructed digital elevation model.

Resolution:	unknown
Point density:	unknown

Processing Parameters

**General**	
Cameras	2595
Aligned cameras	2577
Markers	110
**Shapes**	
Polygon	1
Coordinate system	ETRS89 / UTM zone 30N (EPSG::25830)
Rotation angles	Yaw, Pitch, Roll
**Tie Points**	
Points	1,760,863 of 12,529,745
RMS reprojection error	0.139417 (0.328057 pix)
Max reprojection error	0.299926 (1.62794 pix)
Mean key point size	2.31299 pix
Point colors	3 bands, uint8
Key points	No
Average tie point multiplicity	3.65511
**Alignment parameters**	
Accuracy	High
Generic preselection	Yes
Reference preselection	No
Key point limit	60,000
Key point limit per Mpx	1,000
Tie point limit	0
Exclude stationary tie points	Yes
Guided image matching	No
Adaptive camera model fitting	No
Matching time	4 hours 7 minutes
Matching memory usage	3.73 GB
Alignment time	2 hours 17 minutes
Alignment memory usage	4.82 GB
**Optimization parameters**	
Parameters	f, cx, cy, k1-k3, p1, p2
Adaptive camera model fitting	No
Optimization time	27 seconds
Date created	2023:11:13 15:04:46
Software version	2.0.0.15597
File size	775.31 MB
**System**	
Software name	Agisoft Metashape Professional
Software version	2.0.3 build 16960
OS	Windows 64 bit
RAM	63.90 GB
CPU	Intel(R) Core(TM) i7-7700 CPU @ 3.60GHz
GPU(s)	Quadro M4000
