# Supplementary material for: Automated cleaning of tie point clouds following USGS guidelines in Agisoft Metashape professional (ver. 2.1.0)
Source: MethodsX. 2024 Mar 26;12:102679. doi: 10.1016/j.mex.2024.102679 (PMC10992719; doi:10.1016/j.mex.2024.102679)
Supplement: Supplementary file 3 — The supplementary material includes supplementary text, figures and the processing reports generated by the software. [file mmc3.zip › Lucia_SCC-Optimized_r4.pdf]

Lucia_SCC-Optimized_r4

Automatically cleaned sparse cloud using the SCC script (optimized settings). UAS data provided by Sanz-Ablanedo et al. (2018).

Sanz-Ablanedo, E., Chandler, J. H., Rodríguez-Pérez, J. R., and Ordóñez, C.: Accuracy of Unmanned Aerial Vehicle (UAV) and SfM Photogrammetry Survey as a Function of the Number and Location of Ground Control Points Used, Remote Sensing, 10, 1606, 2018.

28 December 2023

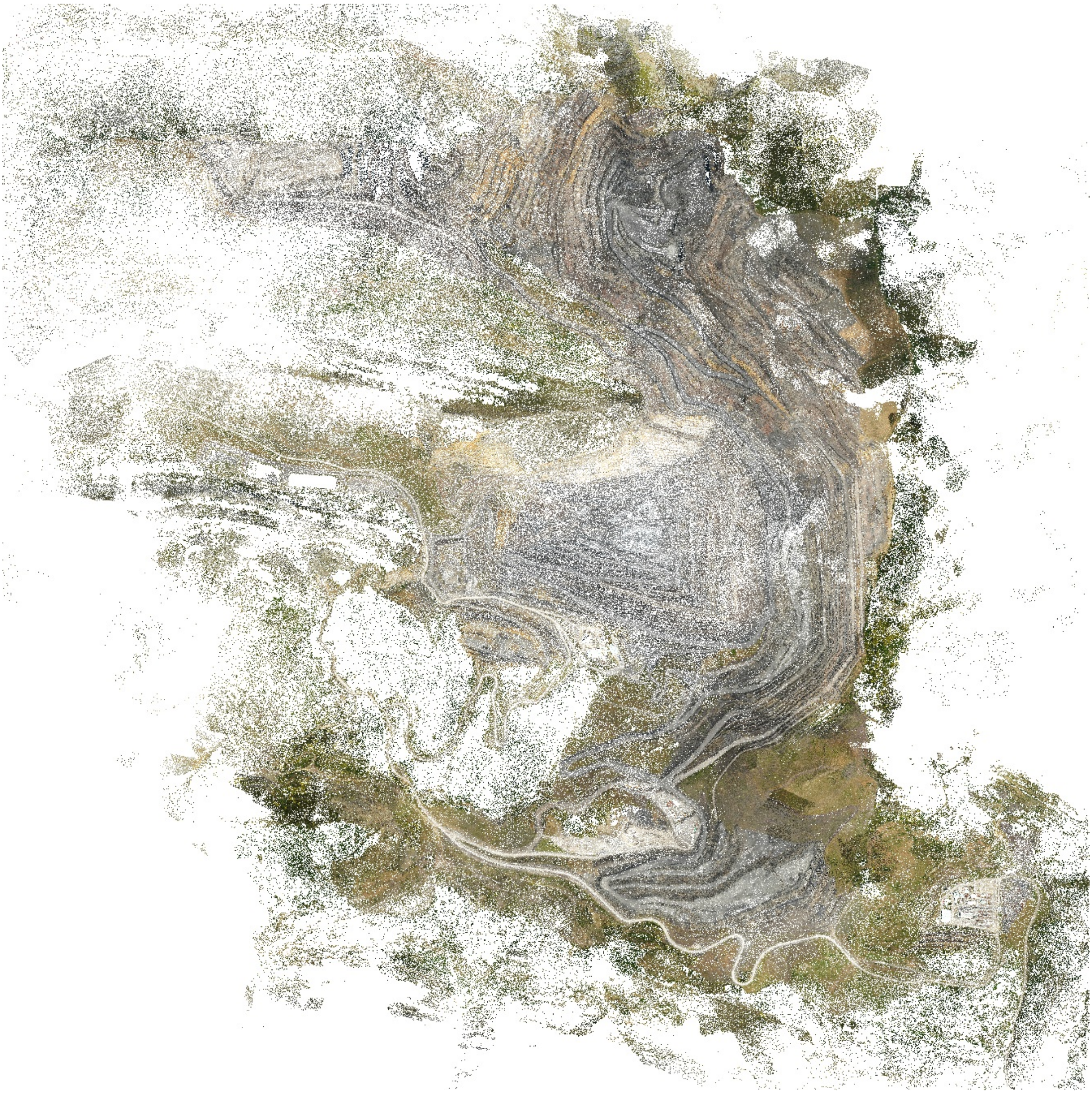

Survey Data

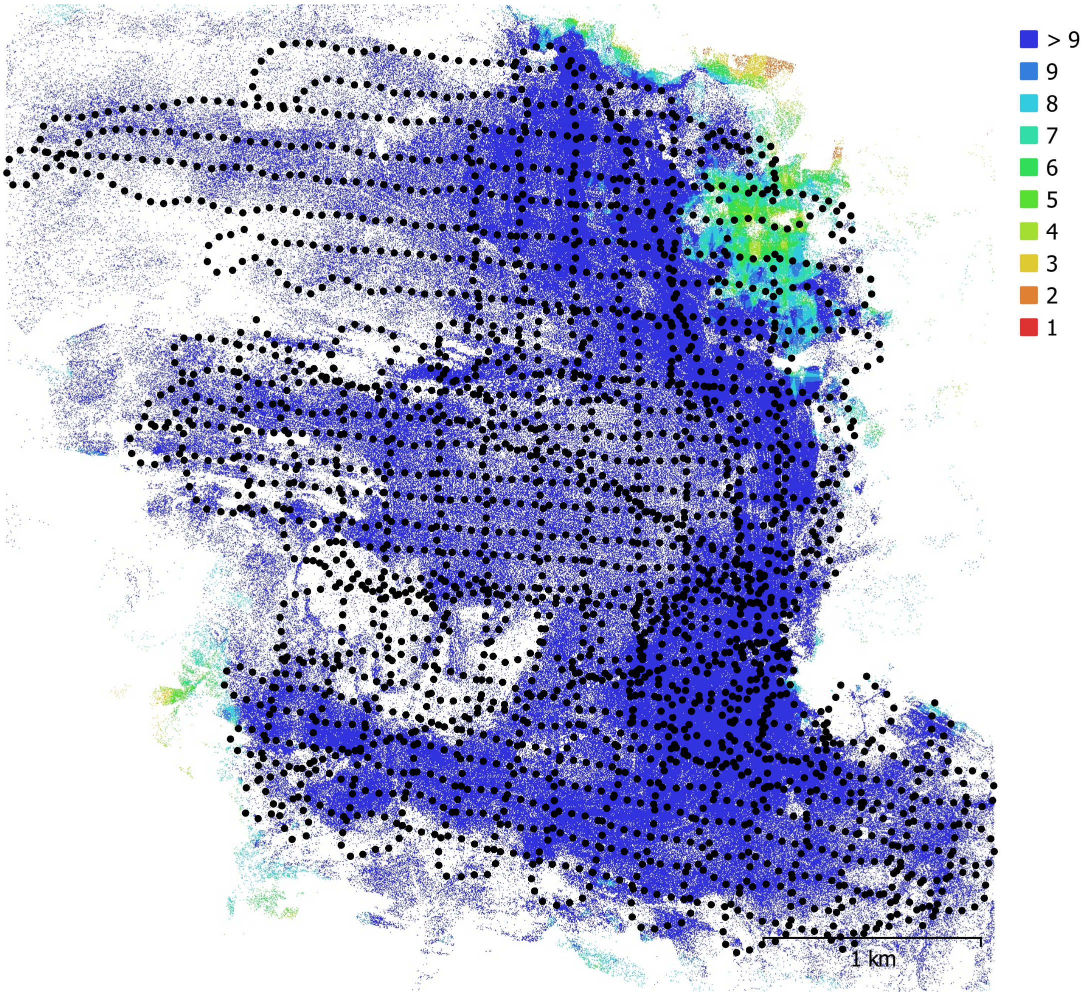

Fig. 1. Camera locations and image overlap.

Number of images:	2,595	Camera stations:	2,577
Flying altitude:	349 m	Tie points:	1,794,285
Ground resolution:	6.2 cm/pix	Projections:	4,265,693
Coverage area:	7.53 km²	Reprojection error:	0.332 pix

**Camera Model**	**Resolution**	**Focal Length**	**Pixel Size**	**Precalibrated**
NX500 (20 mm)	6480 x 4320	20 mm	3.7 x 3.7 μm	No
NX500 (20 mm)	6480 x 4320	20 mm	3.7 x 3.7 μm	No
NX500 (20 mm)	6480 x 4320	20 mm	3.7 x 3.7 μm	No
NX500 (20 mm)	6480 x 4320	20 mm	3.7 x 3.7 μm	No
NX500 (20 mm)	6480 x 4320	20 mm	3.7 x 3.7 μm	No

Camera Model	Resolution	Focal Length	Pixel Size	Precalibrated
NX500 (20 mm)	6480 x 4320	20 mm	3.7 x 3.7 μm	No

Table 1. Cameras.

Camera Calibration

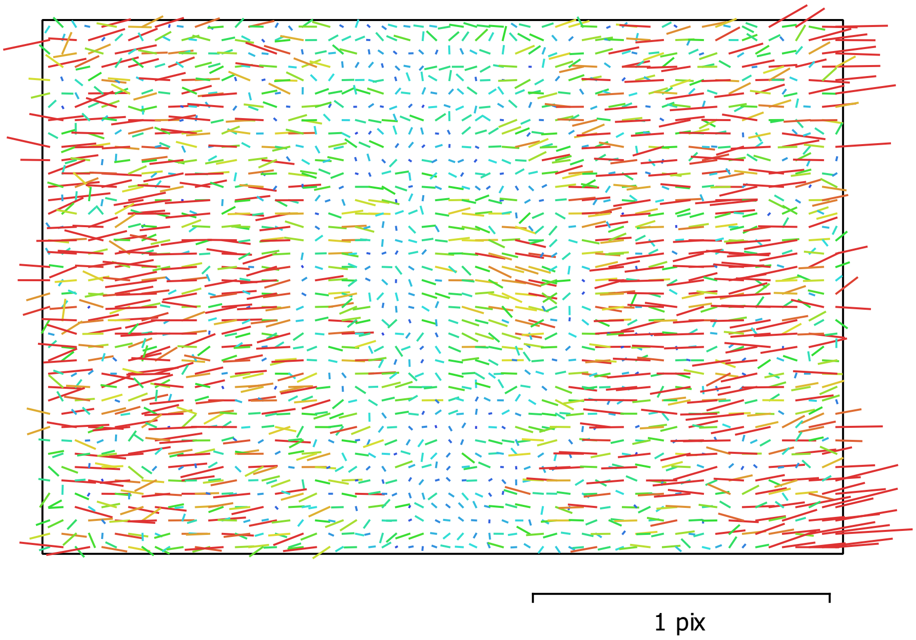

Fig. 2. Image residuals for NX500 (20 mm).

NX500 (20 mm)

200 images

Type	Resolution	Focal Length	Pixel Size
**Frame**	**6480 x 4320**	**20 mm**	**3.7 x 3.7 µm**

	Value	Error	F	Cx	Cy	K1	K2	K3	P1	P2
**F**	**5621.3**	0.05	1.00	0.02	0.01	-0.39	0.33	-0.30	-0.00	0.07
**Cx**	**93.8815**	0.06		1.00	-0.04	0.03	-0.02	0.02	0.83	0.06
**Cy**	**36.2332**	0.068			1.00	-0.01	0.00	-0.01	-0.03	0.78
**K1**	**-0.0121186**	6.4e-05				1.00	-0.96	0.91	0.05	-0.00
**K2**	**0.02712**	0.00032					1.00	-0.98	-0.05	-0.01
**K3**	**-0.0241897**	0.00047						1.00	0.05	0.01
**P1**	**0.00277359**	3.6e-06							1.00	0.04
**P2**	**0.000807946**	4.2e-06								1.00

Table 2. Calibration coefficients and correlation matrix.

Camera Calibration

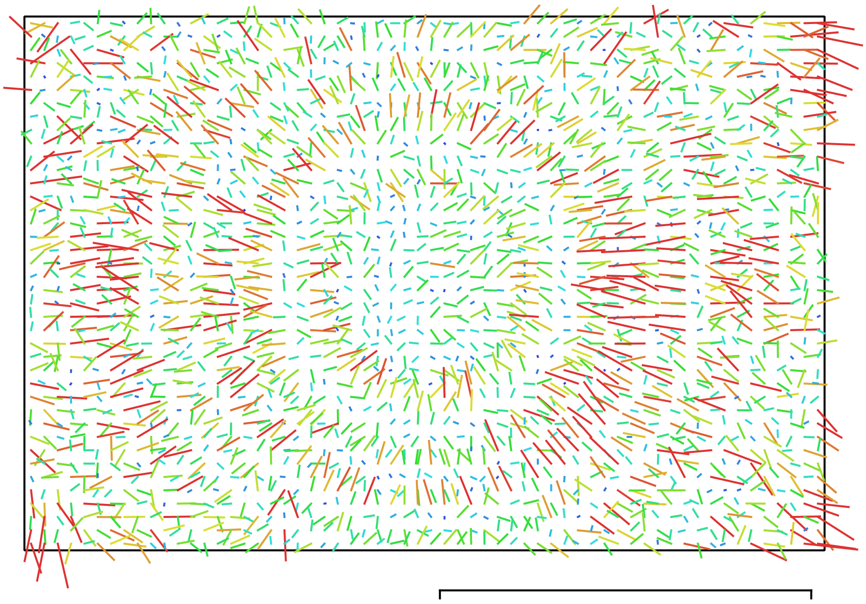

Fig. 3. Image residuals for NX500 (20 mm).

NX500 (20 mm)

462 images

Type	Resolution	Focal Length	Pixel Size
**Frame**	**6480 x 4320**	**20 mm**	**3.7 x 3.7 µm**

	Value	Error	F	Cx	Cy	K1	K2	K3	P1	P2
**F**	**5629.42**	0.041	1.00	-0.16	-0.12	-0.35	0.32	-0.28	-0.04	-0.02
**Cx**	**72.0479**	0.041		1.00	0.06	0.03	-0.03	0.03	0.88	0.01
**Cy**	**44.5558**	0.035			1.00	-0.00	-0.02	0.02	0.05	0.78
**K1**	**-0.0119053**	4.7e-05				1.00	-0.97	0.91	0.03	0.01
**K2**	**0.0280331**	0.00023					1.00	-0.98	-0.03	-0.03
**K3**	**-0.0267018**	0.00035						1.00	0.04	0.03
**P1**	**0.00229501**	2.6e-06							1.00	0.02
**P2**	**0.00119916**	2e-06								1.00

Table 3. Calibration coefficients and correlation matrix.

Camera Calibration

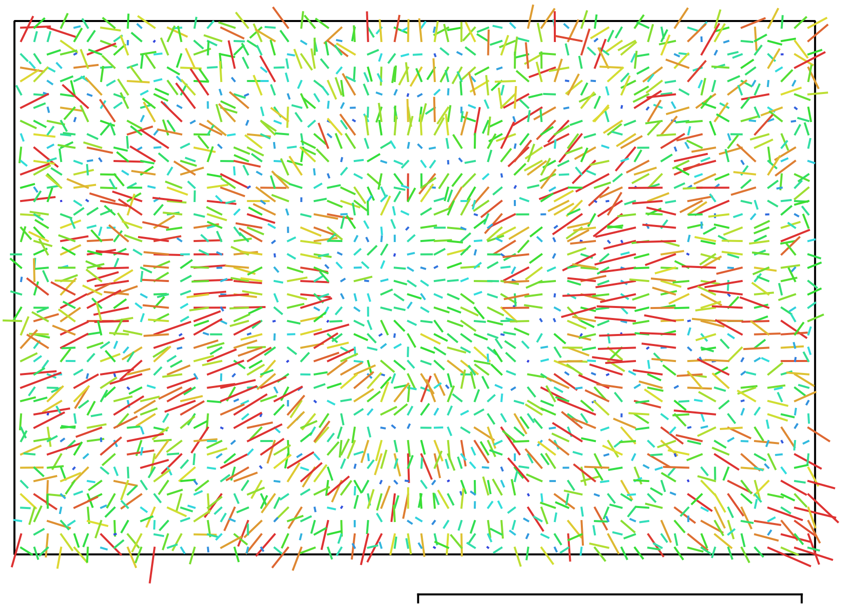

Fig. 4. Image residuals for NX500 (20 mm).

NX500 (20 mm)

530 images

Type	Resolution	Focal Length	Pixel Size
**Frame**	**6480 x 4320**	**20 mm**	**3.7 x 3.7 µm**

	**Value**	**Error**	**F**	**Cx**	**Cy**	**K1**	**K2**	**K3**	**P1**	**P2**
**F**	**5628.27**	0.044	1.00	-0.02	-0.13	-0.26	0.25	-0.23	-0.00	-0.01
**Cx**	**84.269**	0.039		1.00	-0.02	0.01	-0.01	0.01	0.83	-0.00
**Cy**	**35.2359**	0.03			1.00	0.01	-0.02	0.01	-0.01	0.68
**K1**	**-0.0119735**	4.1e-05				1.00	-0.96	0.91	0.02	0.01
**K2**	**0.0303109**	0.00021					1.00	-0.98	-0.02	-0.02
**K3**	**-0.0315451**	0.00033						1.00	0.03	0.02
**P1**	**0.00253953**	2.3e-06							1.00	0.02
**P2**	**0.000930503**	1.7e-06								1.00

Table 4. Calibration coefficients and correlation matrix.

Camera Calibration

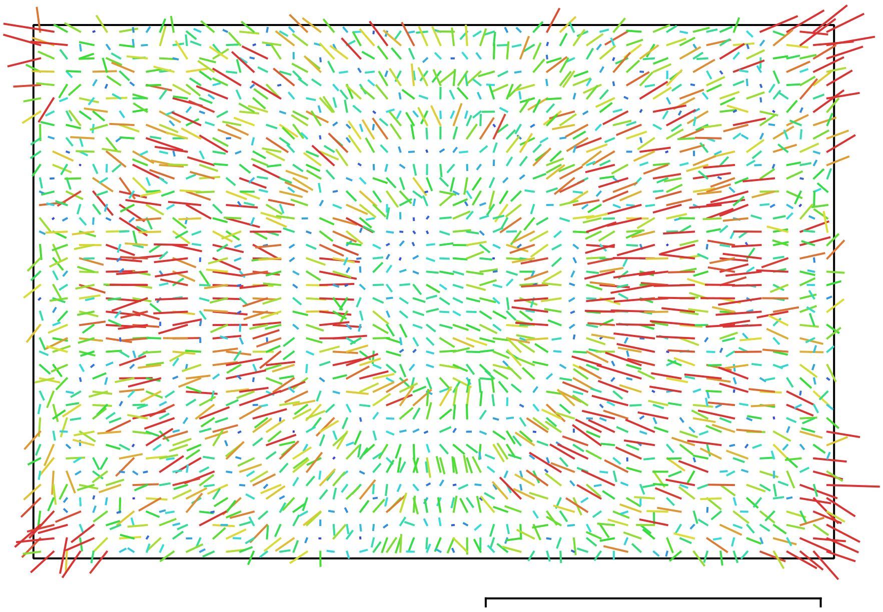

Fig. 5. Image residuals for NX500 (20 mm).

NX500 (20 mm)

513 images

Type	Resolution	Focal Length	Pixel Size
**Frame**	**6480 x 4320**	**20 mm**	**3.7 x 3.7 μm**

	Value	Error	F	Cx	Cy	K1	K2	K3	P1	P2
**F**	**5625.08**	0.052	1.00	-0.08	-0.08	-0.21	0.20	-0.18	0.01	-0.02
**Cx**	**84.0624**	0.035		1.00	-0.01	0.01	-0.01	0.01	0.81	-0.01
**Cy**	**59.4579**	0.028			1.00	0.01	-0.03	0.03	-0.02	0.73
**K1**	**-0.0105505**	3.6e-05				1.00	-0.96	0.90	0.03	0.01
**K2**	**0.0213666**	0.00019					1.00	-0.98	-0.03	-0.01
**K3**	**-0.014972**	0.00028						1.00	0.04	0.01
**P1**	**0.00251443**	2.1e-06							1.00	-0.02
**P2**	**0.00150427**	1.7e-06								1.00

Table 5. Calibration coefficients and correlation matrix.

Camera Calibration

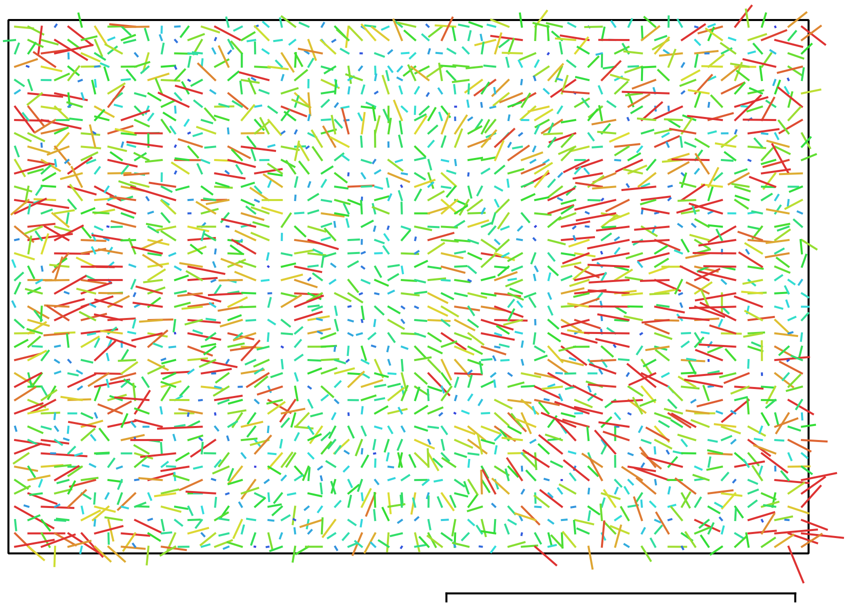

Fig. 6. Image residuals for NX500 (20 mm).

NX500 (20 mm)

412 images

Type	Resolution	Focal Length	Pixel Size
**Frame**	**6480 x 4320**	**20 mm**	**3.7 x 3.7 µm**

	**Value**	**Error**	**F**	**Cx**	**Cy**	**K1**	**K2**	**K3**	**P1**	**P2**
**F**	**5626.22**	0.045	1.00	0.05	-0.10	-0.38	0.35	-0.32	0.04	-0.01
**Cx**	**89.2669**	0.048		1.00	0.06	-0.01	0.01	-0.00	0.88	0.04
**Cy**	**45.2186**	0.038			1.00	-0.02	0.02	-0.03	0.05	0.75
**K1**	**-0.0126588**	5.4e-05				1.00	-0.97	0.91	0.01	-0.01
**K2**	**0.0307323**	0.00027					1.00	-0.98	-0.01	0.00
**K3**	**-0.0314517**	0.0004						1.00	0.02	-0.01
**P1**	**0.00262345**	2.9e-06							1.00	0.05
**P2**	**0.00112999**	2.2e-06								1.00

Table 6. Calibration coefficients and correlation matrix.

Camera Calibration

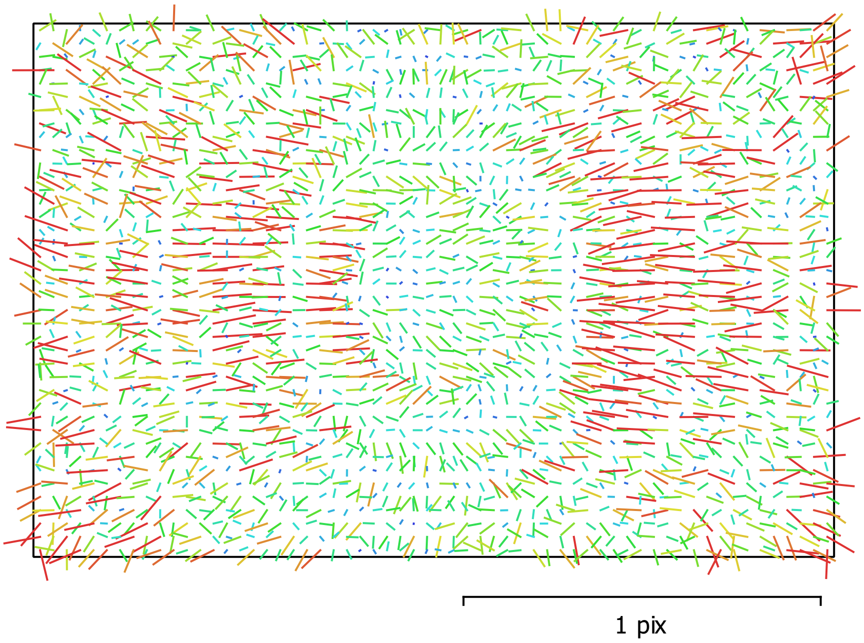

Fig. 7. Image residuals for NX500 (20 mm).

NX500 (20 mm)

478 images

Type	Resolution	Focal Length	Pixel Size
**Frame**	**6480 x 4320**	**20 mm**	**3.7 x 3.7 μm**

	Value	Error	F	Cx	Cy	K1	K2	K3	P1	P2
**F**	**5627.15**	0.032	1.00	-0.01	-0.00	-0.44	0.39	-0.34	-0.01	0.02
**Cx**	**68.6155**	0.041		1.00	-0.01	0.01	-0.01	0.00	0.87	-0.03
**Cy**	**48.4852**	0.036			1.00	0.02	-0.03	0.03	-0.02	0.74
**K1**	**-0.0126167**	4.8e-05				1.00	-0.97	0.91	0.01	0.01
**K2**	**0.0354408**	0.00025					1.00	-0.98	-0.01	-0.02
**K3**	**-0.0393425**	0.00037						1.00	0.01	0.02
**P1**	**0.00201422**	2.6e-06							1.00	-0.02
**P2**	**0.00127158**	2.1e-06								1.00

Table 7. Calibration coefficients and correlation matrix.

Ground Control Points

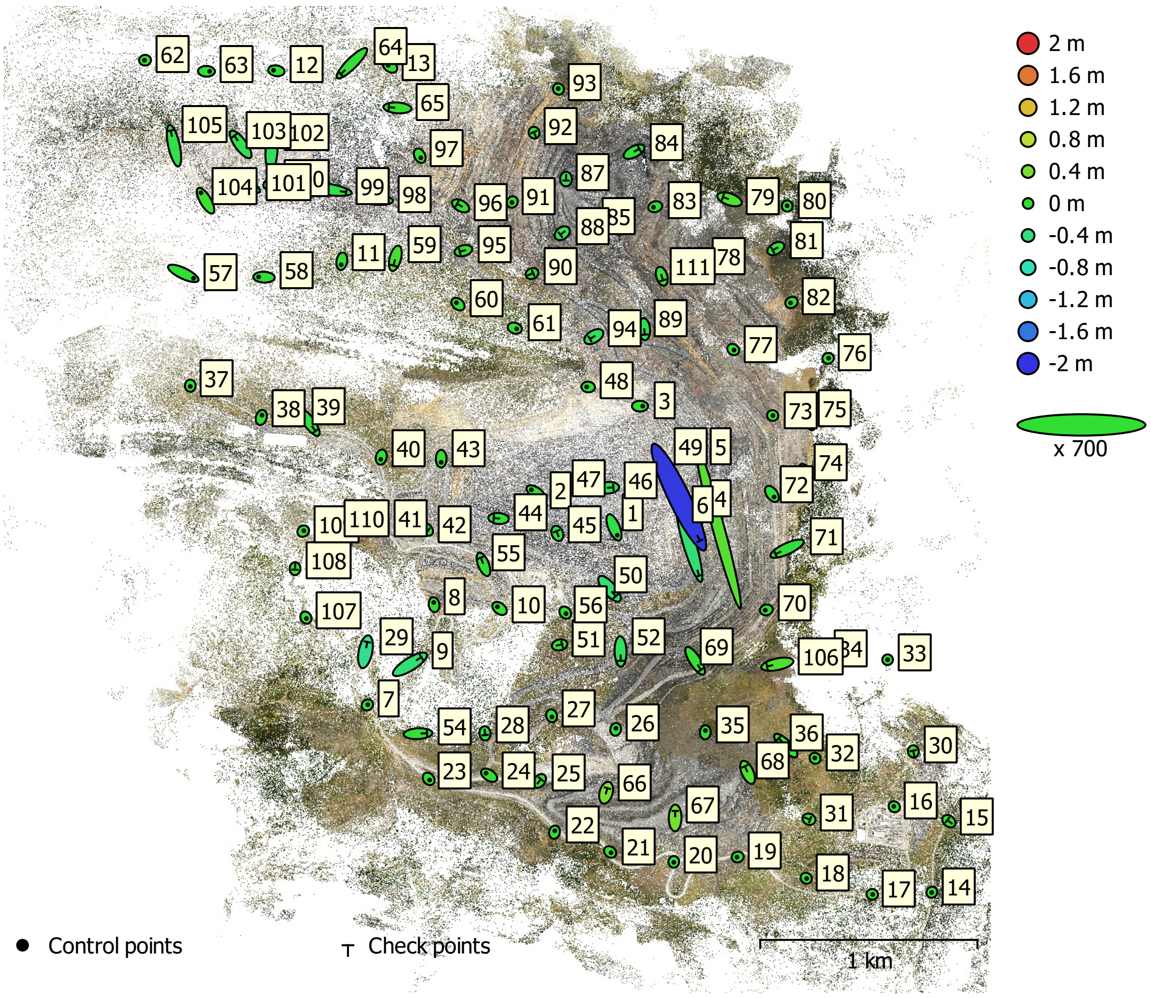

Fig. 8. GCP locations and error estimates.
Z error is represented by ellipse color. X,Y errors are represented by ellipse shape.
Estimated GCP locations are marked with a dot or crossing.

Count	X error (cm)	Y error (cm)	Z error (cm)	XY error (cm)	Total (cm)
55	2.86061	3.15019	3.01301	4.25521	5.21392

Table 8. Control points RMSE.
X - Easting, Y - Northing, Z - Altitude.

Count	X error (cm)	Y error (cm)	Z error (cm)	XY error (cm)	Total (cm)
54	9.46664	18.0144	32.0117	20.3504	37.9327

Table 9. Check points RMSE.
X - Easting, Y - Northing, Z - Altitude.

Label	X error (cm)	Y error (cm)	Z error (cm)	Total (cm)	Image (pix)
1	4.32223	-10.2641	-11.796	16.2228	0.518 (104)
2	-5.97282	4.03167	0.299289	7.21238	0.504 (109)
3	3.54813	-0.0543424	-0.936102	3.66994	0.208 (51)
4	0.207377	11.7952	16.651	20.4065	0.662 (50)
7	0.635527	0.455538	0.110863	0.789747	0.102 (24)
8	-0.430398	2.81146	-0.0368379	2.84446	0.353 (32)
10	-2.87075	2.17944	2.27967	4.26475	0.523 (42)
11	-0.741692	-4.5348	-0.873706	4.67738	0.289 (36)
12	-3.73906	0.621874	0.461119	3.81837	0.422 (26)
13	-2.61457	1.64774	0.506173	3.13165	0.188 (20)
14	-0.268321	-0.410548	0.0489167	0.492888	0.079 (23)
16	-0.59913	0.435425	0.054857	0.742672	0.094 (34)
17	0.53116	-0.109841	-0.100018	0.551543	0.076 (23)
18	0.563097	-0.48754	-0.0928667	0.750598	0.120 (25)
19	-0.989025	-0.215549	-0.109214	1.01812	0.111 (20)
20	-0.0612122	-0.68089	0.0579571	0.686089	0.133 (16)
21	1.25777	-0.978532	0.128116	1.59872	0.158 (15)
22	0.392271	1.74031	-0.102676	1.78692	0.134 (13)
23	1.26754	-1.5028	0.203654	1.9765	0.147 (18)
24	-3.98437	2.59519	-0.497631	4.78099	0.343 (27)
26	0.302789	1.39177	-0.577124	1.5368	0.138 (33)
27	0.436824	-1.49223	0.0428739	1.55545	0.228 (27)
32	-0.34491	0.397847	-0.0155637	0.52677	0.078 (18)
33	0.0129134	-0.00574428	-0.0257231	0.0293501	0.003 (3)
34	-0.000130613	0.0642685	-0.0125559	0.0654836	0.012 (4)
35	0.118188	2.31286	-0.442736	2.35782	0.269 (11)
37	-0.0148751	-0.999826	0.310292	1.04697	0.127 (46)
38	0.891534	2.94153	-0.386429	3.09786	0.180 (57)
40	-0.694687	-2.99604	-1.3445	3.35657	0.279 (66)
43	-0.024833	-4.65105	0.241763	4.6574	0.502 (69)
48	-1.93631	0.154173	-0.475186	1.99972	0.207 (44)

Label	X error (cm)	Y error (cm)	Z error (cm)	Total (cm)	Image (pix)
56	1.04471	-1.3199	-0.256988	1.70282	0.320 (50)
57	13.4864	-6.43752	-1.52146	15.0213	1.497 (14)
58	-7.04138	0.299599	-1.81173	7.27689	0.975 (30)
60	-2.10501	1.80852	0.307279	2.79217	0.193 (32)
61	2.42812	-0.838159	0.660342	2.65223	0.162 (20)
62	-0.799541	-0.086006	0.269026	0.847961	0.152 (17)
63	4.09147	-0.11508	-0.255148	4.10103	0.325 (16)
70	-1.37286	-0.31854	-1.16518	1.82862	0.118 (30)
72	2.87473	-3.96418	-3.60125	6.07847	0.289 (18)
73	0.391347	-0.0923047	0.296146	0.499374	0.066 (15)
76	0.616085	0.163917	-0.0258449	0.638042	0.097 (9)
77	1.04443	-0.694822	0.124716	1.26062	0.091 (11)
78	0.541703	-1.56275	-0.0873959	1.65628	0.120 (11)
80	-0.00387194	0.355536	0.0239258	0.356361	0.092 (7)
82	-1.2674	-0.670934	-0.295801	1.46423	0.213 (6)
83	-2.24676	-0.609627	-0.168853	2.33412	0.205 (13)
85	0.491178	0.148983	-0.297646	0.593334	0.209 (10)
91	-0.436616	-0.619791	0.145462	0.771967	0.277 (20)
93	-0.333453	0.57678	-0.082949	0.671376	0.187 (16)
97	1.39713	-2.72559	0.0937116	3.06424	0.380 (40)
100	2.63841	0.657391	-3.54978	4.4715	0.844 (28)
104	-6.06216	10.3584	5.74669	13.3068	1.080 (21)
107	0.634974	-1.10894	-0.449077	1.35448	0.105 (21)
109	0.788249	0.602175	2.32991	2.53228	0.274 (22)
**Total**	**2.86061**	**3.15019**	**3.01301**	**5.21392**	**0.412**

Table 10. Control points.
X - Easting, Y - Northing, Z - Altitude.

Label	X error (cm)	Y error (cm)	Z error (cm)	Total (cm)	Image (pix)
5	-26.6158	98.8234	22.07	104.697	0.659 (38)
6	12.6153	-42.2304	-36.8773	57.4673	0.320 (66)
9	15.0228	9.34243	-36.4262	40.4949	0.255 (29)

Label	X error (cm)	Y error (cm)	Z error (cm)	Total (cm)	Image (pix)
15	-1.91197	1.54939	2.38593	3.42766	0.122 (27)
25	1.90911	2.33669	4.36237	5.30426	0.114 (22)
28	0.205035	-2.19808	-6.91642	7.2602	0.173 (29)
29	2.51679	12.8082	-53.6927	55.2566	0.031 (18)
30	-0.223043	1.03879	1.46277	1.80791	0.106 (20)
31	1.36505	-0.528189	7.46841	7.61049	0.146 (26)
36	-9.38208	9.49673	4.0769	13.9582	0.139 (14)
39	8.76821	-12.0158	-5.40621	15.8268	0.211 (40)
41	1.16032	2.45458	-9.55886	9.93696	0.420 (56)
42	2.91925	-2.23075	-9.66187	10.3368	0.389 (48)
44	-6.33469	0.485542	-5.28013	8.26098	0.612 (73)
45	-1.14851	2.84889	-9.49149	9.97615	0.571 (94)
46	6.74938	0.491105	-17.6298	18.884	0.425 (63)
47	2.46286	4.46411	-14.689	15.5486	0.499 (107)
49	27.673	-57.4556	-196.386	206.481	0.827 (59)
50	8.15322	-9.74436	-36.025	38.1998	0.440 (56)
51	3.20184	0.787415	-1.26679	3.53221	0.356 (42)
52	0.312068	-12.2719	-23.5194	26.5304	0.314 (56)
54	11.7733	0.647825	2.01112	11.9614	0.130 (31)
55	-3.48647	8.72001	-0.802547	9.4254	0.354 (51)
59	-2.51212	-9.44871	12.3564	15.7566	0.273 (9)
64	-14.4749	-14.4269	-0.640164	20.4467	0.295 (13)
65	-11.3635	0.810476	2.03492	11.5726	0.342 (31)
66	1.72063	5.97586	43.5536	43.9953	0.179 (22)
67	0.0977743	9.23646	38.583	39.6733	0.172 (12)
68	-3.59054	7.92613	18.2708	20.237	0.143 (16)
69	7.60495	-11.9005	4.10676	14.7079	0.225 (28)
71	-15.2642	-7.04276	-5.1196	17.5729	0.243 (24)
74	3.15843	-5.6228	7.36691	9.79096	0.099 (10)
75	-2.20233	6.59753	4.44638	8.25518	0.027 (6)
79	-8.70344	2.98116	18.7277	20.8654	0.142 (8)
81	-4.08189	-2.26805	-7.98874	9.25342	0.251 (6)

Label	X error (cm)	Y error (cm)	Z error (cm)	Total (cm)	Image (pix)
84	6.98512	3.71456	-5.01431	9.3666	0.192 (12)
87	-0.0385927	-1.92825	-19.849	19.9425	0.266 (10)
88	-2.56261	-1.93072	-21.7054	21.9412	0.262 (13)
89	0.556887	-7.09488	-13.7457	15.4788	0.186 (27)
90	1.45378	0.609841	0.169297	1.58558	0.169 (28)
92	-0.319156	0.768487	1.9089	2.08239	0.236 (18)
94	-5.10046	-3.08157	-25.0492	25.7483	0.178 (35)
95	-4.70268	-1.27832	0.0228182	4.87338	0.271 (38)
96	-4.6358	2.53999	-1.21304	5.42343	0.296 (23)
98	15.6154	-1.41763	-1.81446	15.7843	0.599 (32)
99	27.9242	-4.24051	-14.9241	31.9448	0.616 (30)
101	-16.4922	8.15275	-1.84164	18.4892	0.685 (26)
102	1.15862	17.3435	-23.8797	29.5361	0.374 (21)
103	-8.34765	11.4181	-18.7162	23.4596	0.480 (20)
105	-4.29996	19.9162	-14.545	25.034	0.194 (24)
106	-13.6534	-2.85419	17.5028	22.381	0.091 (13)
108	-0.0659024	-1.40926	3.60195	3.86839	0.174 (19)
110	-2.95571	2.3291	-4.70976	6.0285	0.277 (28)
111	1.61972	-5.39544	5.13788	7.62444	0.148 (14)
**Total**	**9.46664**	**18.0144**	**32.0117**	**37.9327**	**0.408**

Table 11. Check points.

X - Easting, Y - Northing, Z - Altitude.

Digital Elevation Model

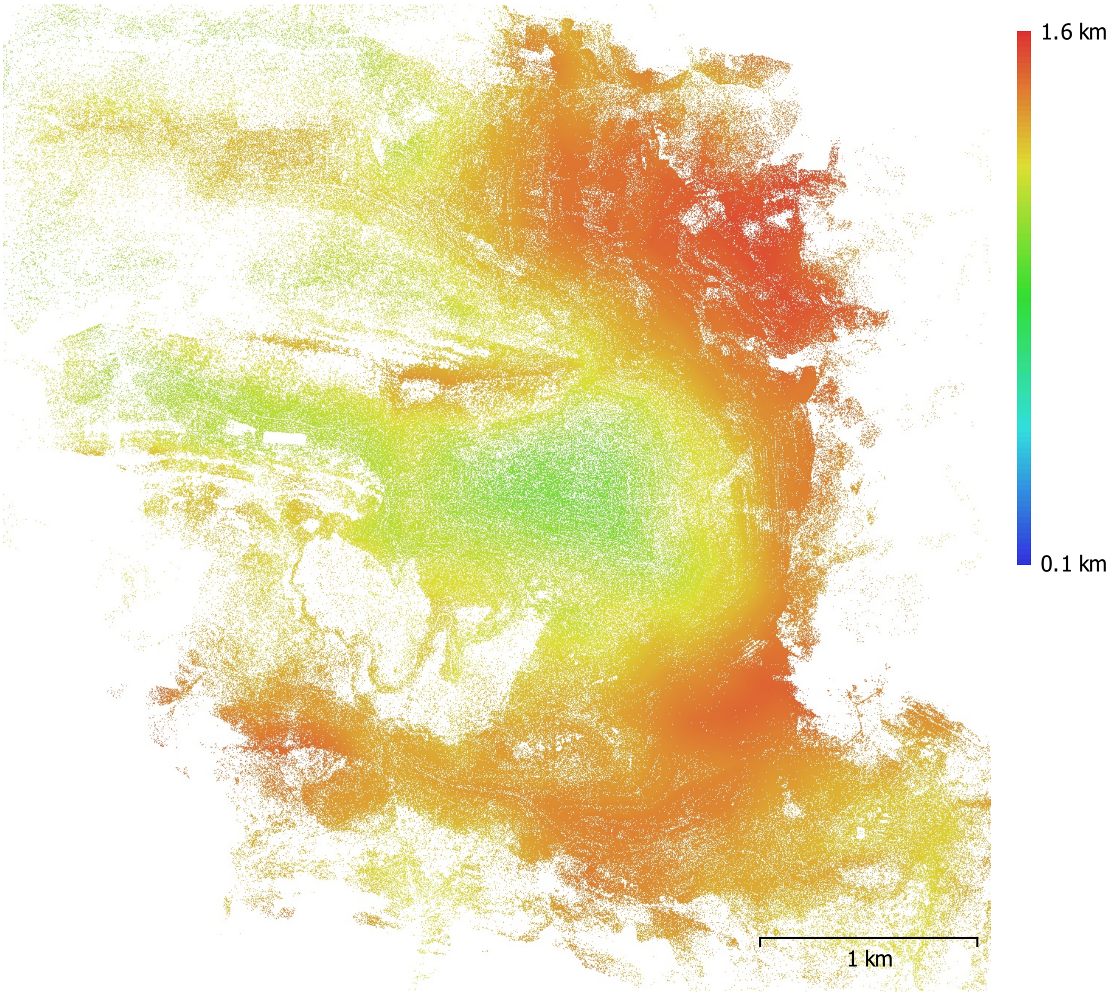

Fig. 9. Reconstructed digital elevation model.

Resolution:	unknown
Point density:	unknown

Processing Parameters

**General**	
Cameras	2595
Aligned cameras	2577
Markers	110
**Shapes**	
Polygon	1
Coordinate system	ETRS89 / UTM zone 30N (EPSG::25830)
Rotation angles	Yaw, Pitch, Roll
**Tie Points**	
Points	1,794,285 of 12,529,745
RMS reprojection error	0.141149 (0.332172 pix)
Max reprojection error	0.299998 (1.66088 pix)
Mean key point size	2.31358 pix
Point colors	3 bands, uint8
Key points	No
Average tie point multiplicity	3.65511
**Alignment parameters**	
Accuracy	High
Generic preselection	Yes
Reference preselection	No
Key point limit	60,000
Key point limit per Mpx	1,000
Tie point limit	0
Exclude stationary tie points	Yes
Guided image matching	No
Adaptive camera model fitting	No
Matching time	4 hours 7 minutes
Matching memory usage	3.73 GB
Alignment time	2 hours 17 minutes
Alignment memory usage	4.82 GB
**Optimization parameters**	
Parameters	f, cx, cy, k1-k3, p1, p2
Adaptive camera model fitting	No
Optimization time	2 minutes 34 seconds
Date created	2023:11:13 15:04:46
Software version	2.0.0.15597
File size	776.07 MB
**System**	
Software name	Agisoft Metashape Professional
Software version	2.0.3 build 16960
OS	Windows 64 bit
RAM	63.90 GB
CPU	Intel(R) Core(TM) i7-7700 CPU @ 3.60GHz
GPU(s)	Quadro M4000
