# Supplementary material for: Automated cleaning of tie point clouds following USGS guidelines in Agisoft Metashape professional (ver. 2.1.0)
Source: MethodsX. 2024 Mar 26;12:102679. doi: 10.1016/j.mex.2024.102679 (PMC10992719; doi:10.1016/j.mex.2024.102679)
Supplement: Supplementary file 3 — The supplementary material includes supplementary text, figures and the processing reports generated by the software. [file mmc3.zip › Lucia_SCC-Optimized_r5.pdf]

Lucia_SCC-Optimized_r5

Automatically cleaned sparse cloud using the SCC script (optimized settings). UAS data provided by Sanz-Ablanedo et al. (2018).

Sanz-Ablanedo, E., Chandler, J. H., Rodríguez-Pérez, J. R., and Ordóñez, C.: Accuracy of Unmanned Aerial Vehicle (UAV) and SfM Photogrammetry Survey as a Function of the Number and Location of Ground Control Points Used, Remote Sensing, 10, 1606, 2018.

28 December 2023

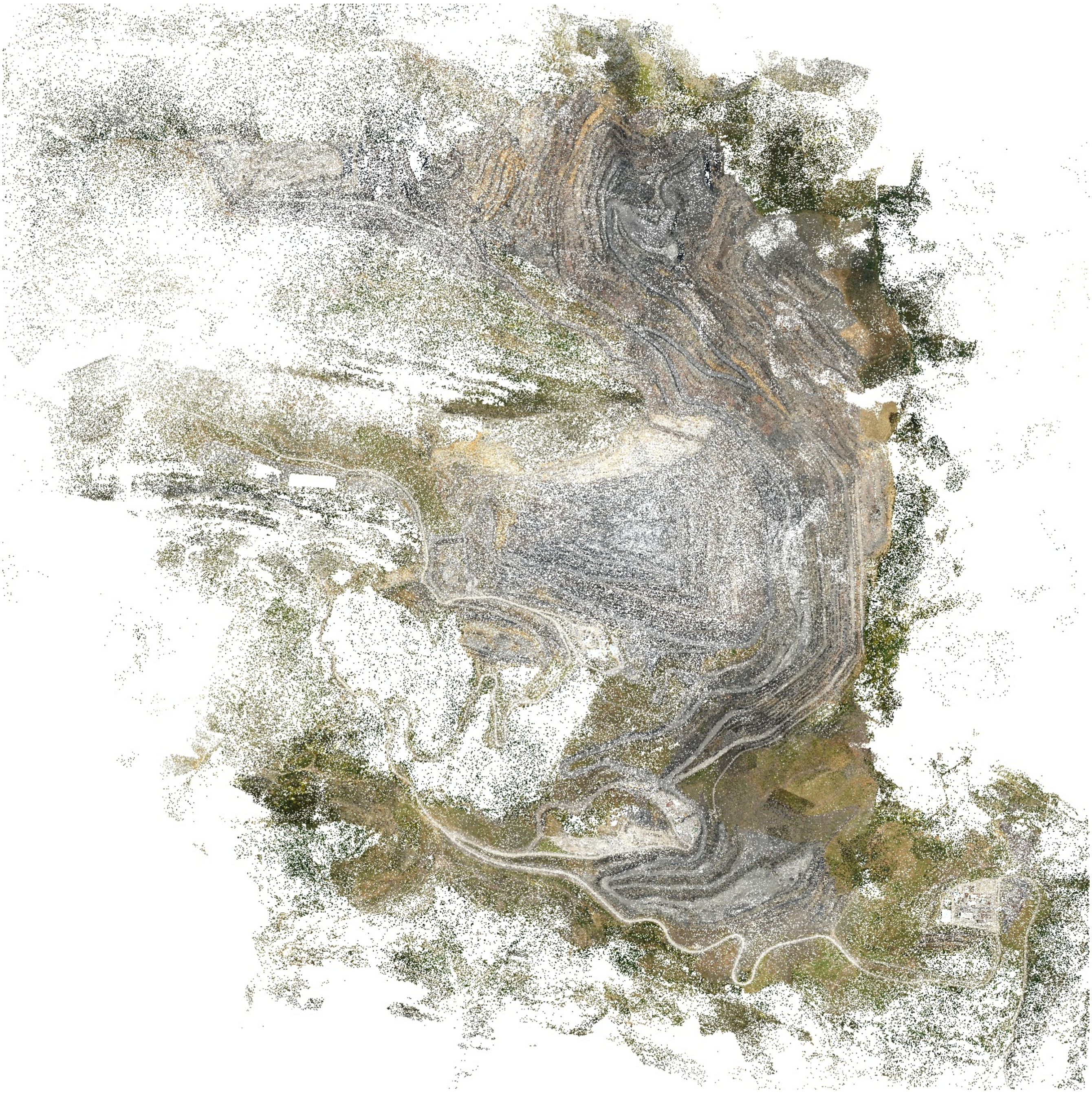

Survey Data

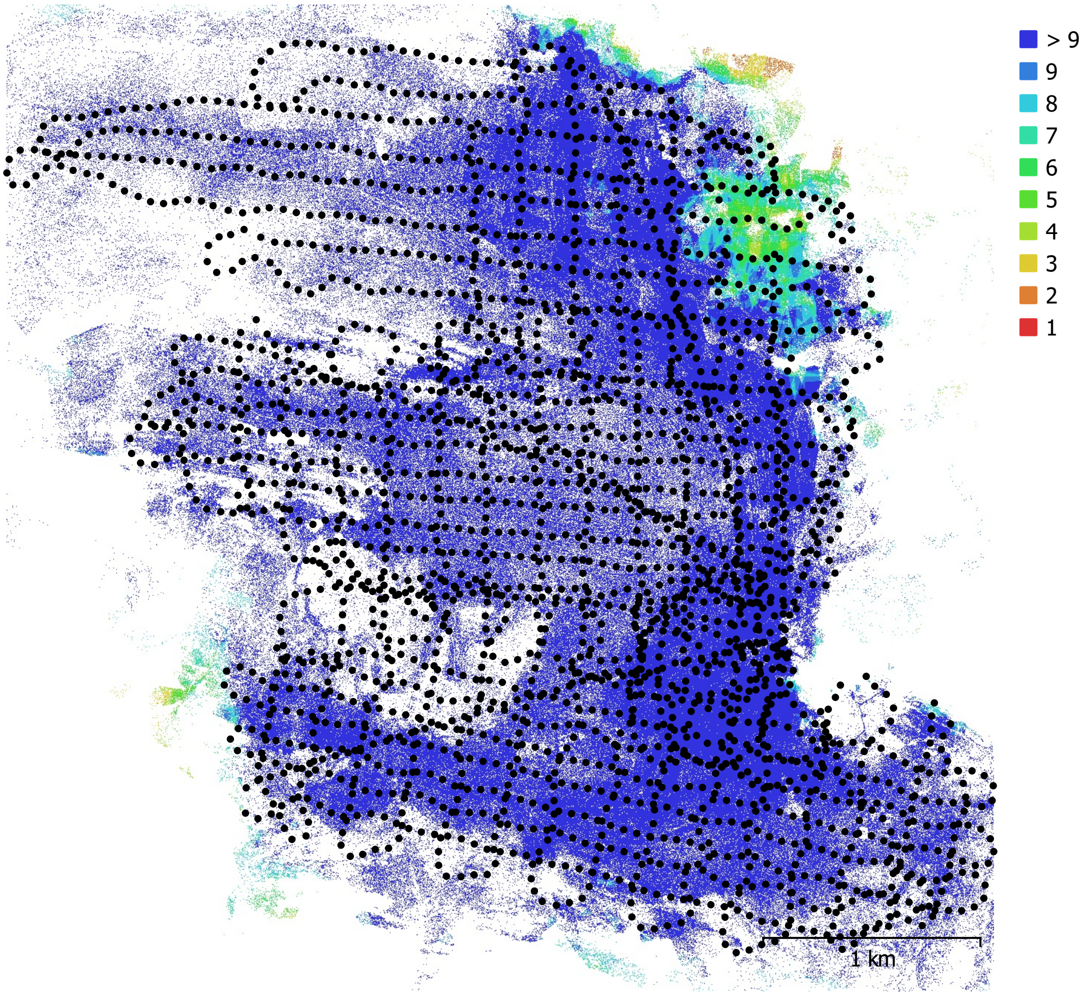

Fig. 1. Camera locations and image overlap.

Number of images:	2,595	Camera stations:	2,577
Flying altitude:	349 m	Tie points:	1,775,103
Ground resolution:	6.2 cm/pix	Projections:	4,207,316
Coverage area:	7.51 km²	Reprojection error:	0.33 pix

Camera Model	Resolution	Focal Length	Pixel Size	Precalibrated
NX500 (20 mm)	6480 x 4320	20 mm	3.7 x 3.7 μm	No
NX500 (20 mm)	6480 x 4320	20 mm	3.7 x 3.7 μm	No
NX500 (20 mm)	6480 x 4320	20 mm	3.7 x 3.7 μm	No
NX500 (20 mm)	6480 x 4320	20 mm	3.7 x 3.7 μm	No
NX500 (20 mm)	6480 x 4320	20 mm	3.7 x 3.7 μm	No

Camera Model	Resolution	Focal Length	Pixel Size	Precalibrated
NX500 (20 mm)	6480 x 4320	20 mm	3.7 x 3.7 μm	No

Table 1. Cameras.

Camera Calibration

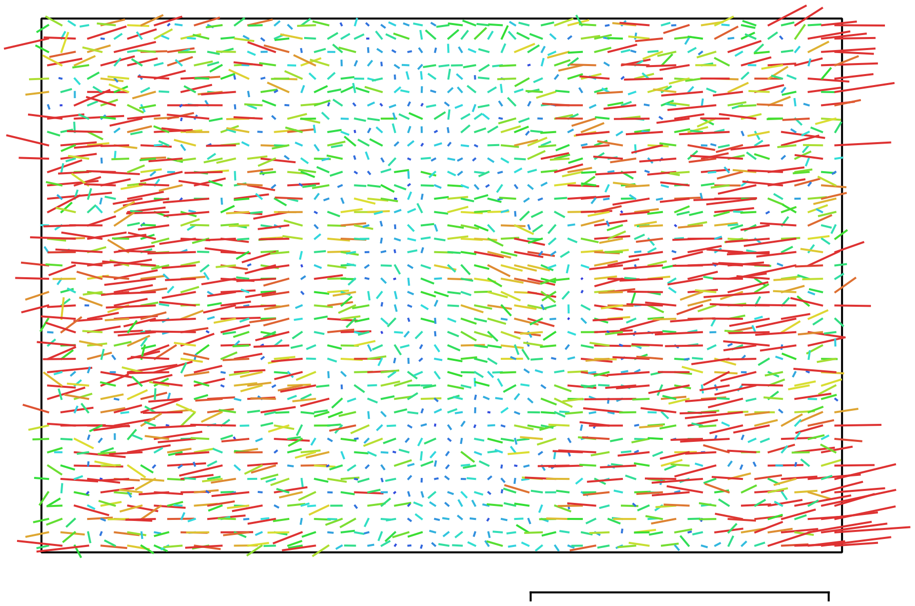

Fig. 2. Image residuals for NX500 (20 mm).

NX500 (20 mm)

200 images

Type	Resolution	Focal Length	Pixel Size
**Frame**	**6480 x 4320**	**20 mm**	**3.7 x 3.7 µm**

	**Value**	**Error**	**F**	**Cx**	**Cy**	**K1**	**K2**	**K3**	**P1**	**P2**
**F**	**5620.91**	0.05	1.00	0.02	0.01	-0.39	0.33	-0.30	-0.00	0.07
**Cx**	**93.6685**	0.06		1.00	-0.04	0.03	-0.02	0.01	0.83	0.06
**Cy**	**36.4713**	0.068			1.00	-0.00	0.00	-0.01	-0.03	0.78
**K1**	**-0.0120867**	6.4e-05				1.00	-0.96	0.91	0.05	0.00
**K2**	**0.0267011**	0.00032					1.00	-0.98	-0.04	-0.01
**K3**	**-0.0235054**	0.00047						1.00	0.05	0.01
**P1**	**0.00276072**	3.6e-06							1.00	0.04
**P2**	**0.000810105**	4.3e-06								1.00

Table 2. Calibration coefficients and correlation matrix.

Camera Calibration

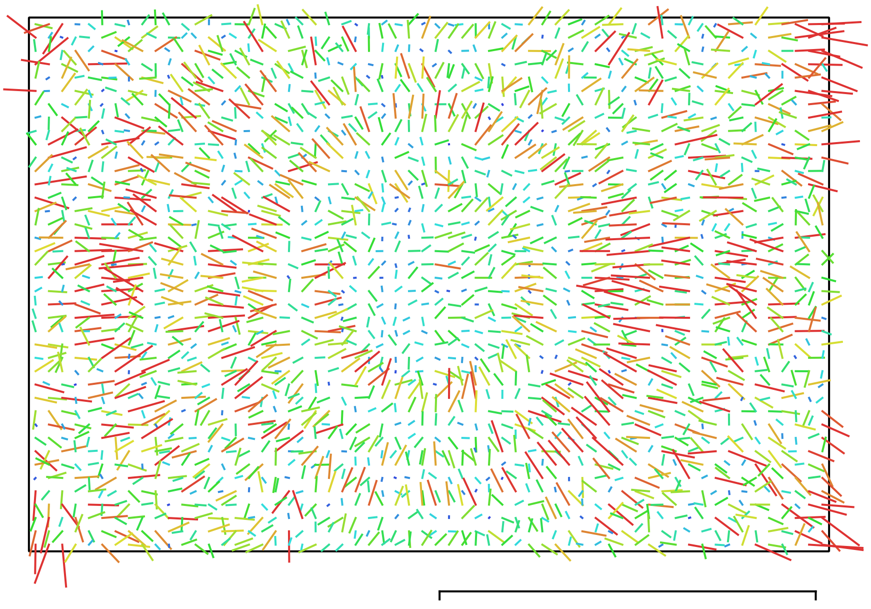

Fig. 3. Image residuals for NX500 (20 mm).

NX500 (20 mm)

462 images

Type	Resolution	Focal Length	Pixel Size
**Frame**	**6480 x 4320**	**20 mm**	**3.7 x 3.7 μm**

	**Value**	**Error**	**F**	**Cx**	**Cy**	**K1**	**K2**	**K3**	**P1**	**P2**
**F**	**5629.21**	0.041	1.00	-0.16	-0.12	-0.35	0.32	-0.28	-0.04	-0.02
**Cx**	**71.7705**	0.041		1.00	0.06	0.03	-0.03	0.03	0.88	0.01
**Cy**	**44.3299**	0.035			1.00	-0.00	-0.02	0.02	0.05	0.78
**K1**	**-0.0117981**	4.7e-05				1.00	-0.97	0.91	0.03	0.01
**K2**	**0.0274197**	0.00024					1.00	-0.98	-0.04	-0.03
**K3**	**-0.0255659**	0.00035						1.00	0.04	0.03
**P1**	**0.00228079**	2.6e-06							1.00	0.02
**P2**	**0.00118879**	2e-06								1.00

Table 3. Calibration coefficients and correlation matrix.

Camera Calibration

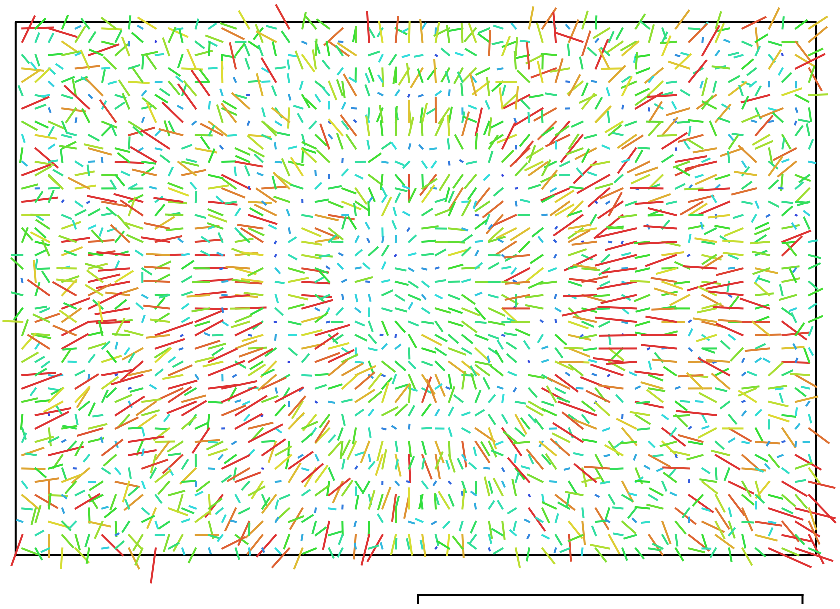

Fig. 4. Image residuals for NX500 (20 mm).

NX500 (20 mm)

530 images

Type	Resolution	Focal Length	Pixel Size
**Frame**	**6480 x 4320**	**20 mm**	**3.7 x 3.7 µm**

	**Value**	**Error**	**F**	**Cx**	**Cy**	**K1**	**K2**	**K3**	**P1**	**P2**
**F**	**5628.43**	0.044	1.00	-0.03	-0.13	-0.26	0.25	-0.22	-0.00	-0.02
**Cx**	**84.2136**	0.039		1.00	-0.02	0.01	-0.01	0.01	0.83	0.00
**Cy**	**35.21**	0.03			1.00	0.01	-0.02	0.01	-0.01	0.68
**K1**	**-0.0119954**	4.1e-05				1.00	-0.96	0.91	0.02	0.01
**K2**	**0.0304358**	0.00021					1.00	-0.98	-0.02	-0.02
**K3**	**-0.0317507**	0.00033						1.00	0.03	0.02
**P1**	**0.00254026**	2.3e-06							1.00	0.02
**P2**	**0.000929241**	1.7e-06								1.00

Table 4. Calibration coefficients and correlation matrix.

Camera Calibration

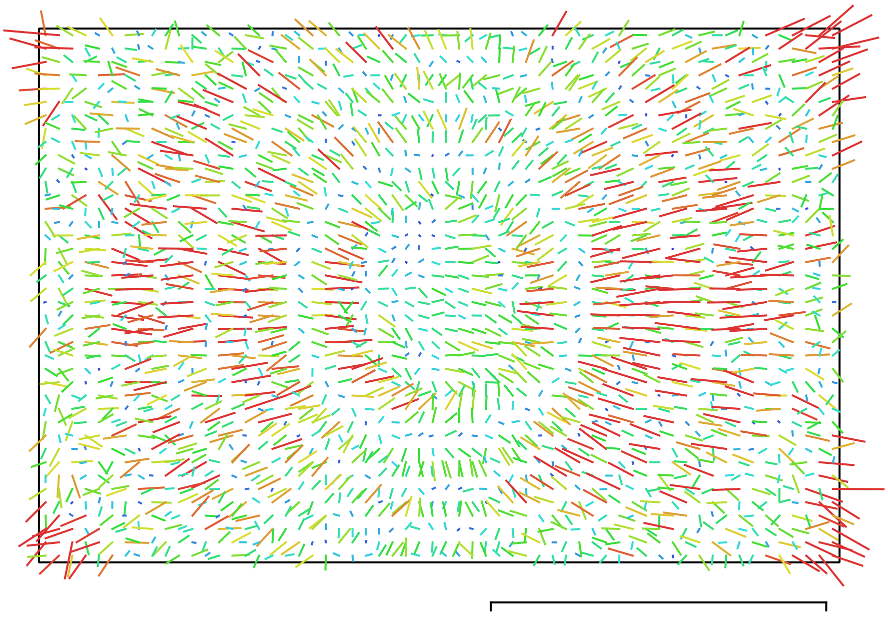

Fig. 5. Image residuals for NX500 (20 mm).

NX500 (20 mm)

513 images

Type	Resolution	Focal Length	Pixel Size
**Frame**	**6480 x 4320**	**20 mm**	**3.7 x 3.7 µm**

	**Value**	**Error**	**F**	**Cx**	**Cy**	**K1**	**K2**	**K3**	**P1**	**P2**
**F**	**5624.63**	0.052	1.00	-0.08	-0.08	-0.20	0.20	-0.18	0.01	-0.02
**Cx**	**83.9971**	0.035		1.00	-0.01	0.01	-0.01	0.02	0.80	-0.01
**Cy**	**59.708**	0.028			1.00	0.01	-0.03	0.03	-0.02	0.73
**K1**	**-0.0105557**	3.6e-05				1.00	-0.96	0.90	0.03	0.01
**K2**	**0.0212411**	0.00019					1.00	-0.98	-0.04	-0.01
**K3**	**-0.0146063**	0.00028						1.00	0.05	0.01
**P1**	**0.00251109**	2.1e-06							1.00	-0.02
**P2**	**0.00150919**	1.7e-06								1.00

Table 5. Calibration coefficients and correlation matrix.

Camera Calibration

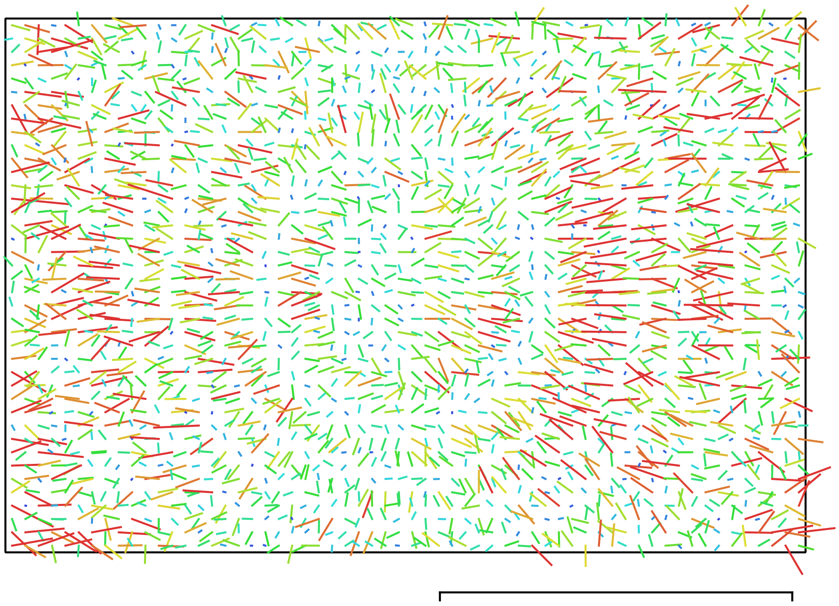

Fig. 6. Image residuals for NX500 (20 mm).

NX500 (20 mm)

412 images

Type	Resolution	Focal Length	Pixel Size
**Frame**	**6480 x 4320**	**20 mm**	**3.7 x 3.7 μm**

	Value	Error	F	Cx	Cy	K1	K2	K3	P1	P2
**F**	**5626.42**	0.046	1.00	0.05	-0.10	-0.37	0.35	-0.32	0.04	-0.01
**Cx**	**89.131**	0.048		1.00	0.05	-0.01	0.01	-0.00	0.88	0.04
**Cy**	**45.2803**	0.038			1.00	-0.02	0.03	-0.04	0.05	0.74
**K1**	**-0.0126337**	5.4e-05				1.00	-0.97	0.91	0.01	-0.01
**K2**	**0.0306162**	0.00027					1.00	-0.98	-0.01	0.00
**K3**	**-0.0314946**	0.0004						1.00	0.02	-0.01
**P1**	**0.00262175**	3e-06							1.00	0.05
**P2**	**0.0011266**	2.2e-06								1.00

Table 6. Calibration coefficients and correlation matrix.

Camera Calibration

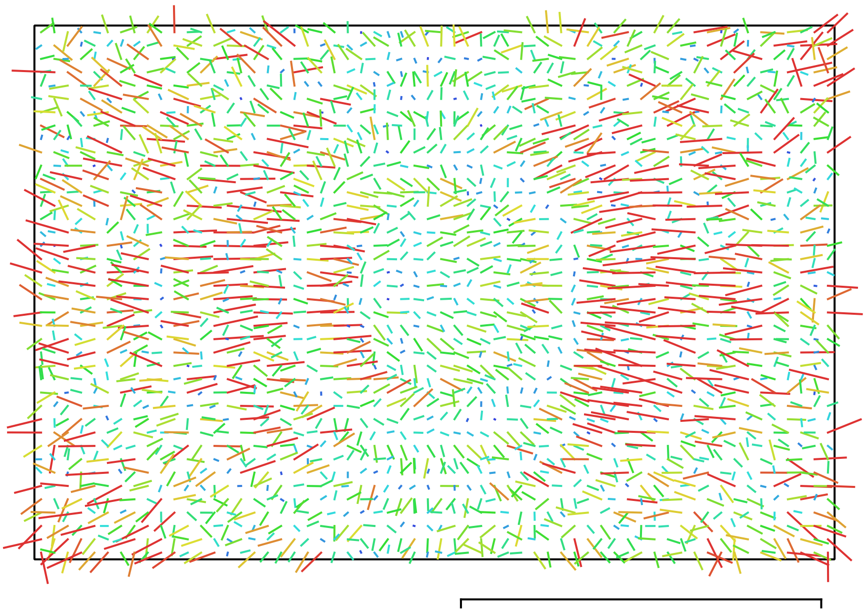

Fig. 7. Image residuals for NX500 (20 mm).

NX500 (20 mm)

478 images

Type	Resolution	Focal Length	Pixel Size
**Frame**	**6480 x 4320**	**20 mm**	**3.7 x 3.7 μm**

	Value	Error	F	Cx	Cy	K1	K2	K3	P1	P2
**F**	**5627.24**	0.032	1.00	-0.01	-0.00	-0.44	0.39	-0.34	-0.01	0.02
**Cx**	**68.7701**	0.041		1.00	-0.01	0.02	-0.01	0.01	0.87	-0.03
**Cy**	**48.3268**	0.036			1.00	0.02	-0.03	0.03	-0.02	0.74
**K1**	**-0.012592**	4.8e-05				1.00	-0.97	0.91	0.01	0.01
**K2**	**0.0354664**	0.00025					1.00	-0.98	-0.01	-0.02
**K3**	**-0.0393648**	0.00037						1.00	0.01	0.02
**P1**	**0.00201353**	2.6e-06							1.00	-0.03
**P2**	**0.00126344**	2.1e-06								1.00

Table 7. Calibration coefficients and correlation matrix.

Ground Control Points

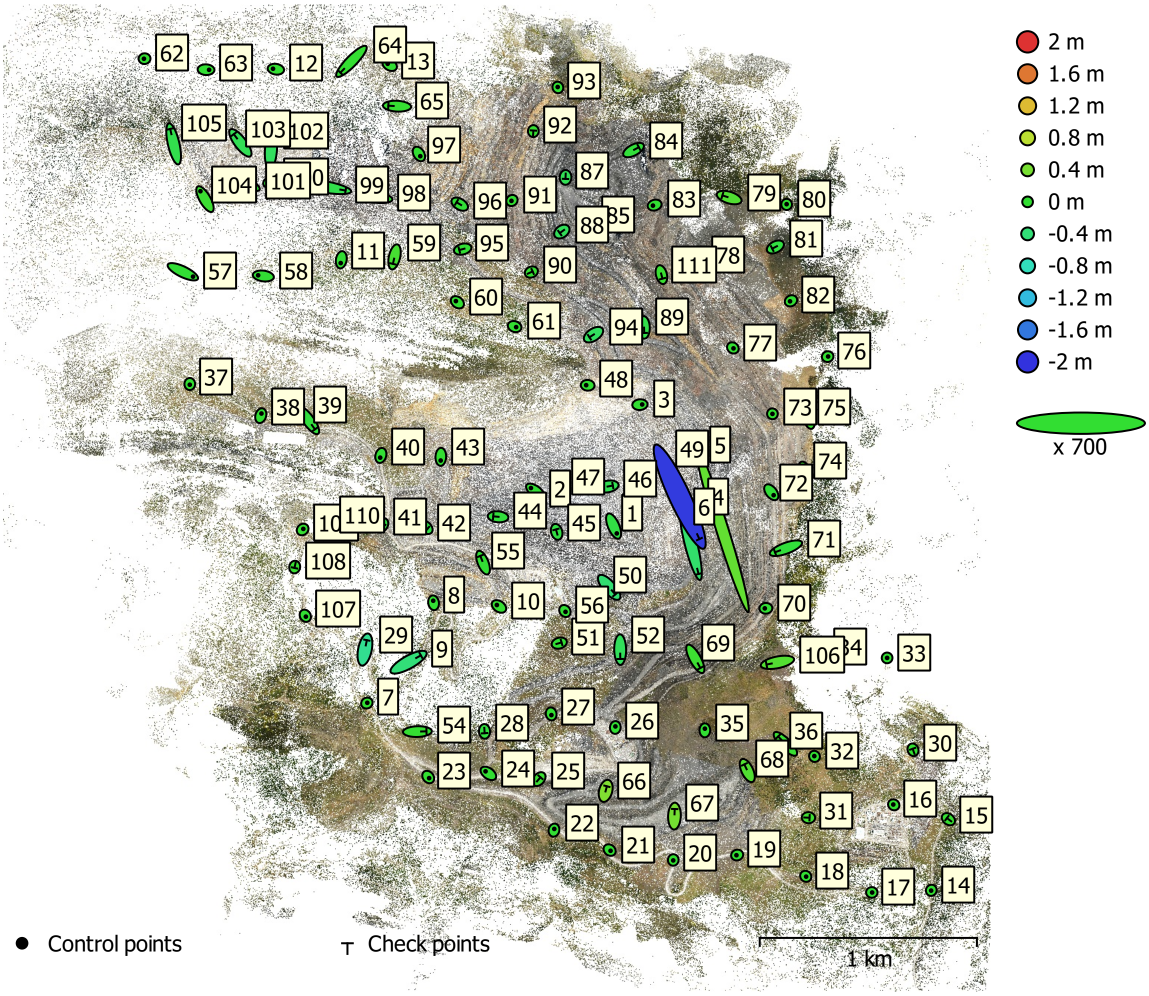

Fig. 8. GCP locations and error estimates.
Z error is represented by ellipse color. X,Y errors are represented by ellipse shape.
Estimated GCP locations are marked with a dot or crossing.

Count	X error (cm)	Y error (cm)	Z error (cm)	XY error (cm)	Total (cm)
55	2.81887	3.00811	2.82591	4.12247	4.99805

Table 8. Control points RMSE.
X - Easting, Y - Northing, Z - Altitude.

Count	X error (cm)	Y error (cm)	Z error (cm)	XY error (cm)	Total (cm)
54	9.7397	18.2196	32.094	20.6595	38.1685

Table 9. Check points RMSE.
X - Easting, Y - Northing, Z - Altitude.

Label	X error (cm)	Y error (cm)	Z error (cm)	Total (cm)	Image (pix)
1	4.21434	-9.63017	-11.1451	15.3204	0.508 (104)
2	-5.90916	4.32589	0.118409	7.32432	0.493 (109)
3	2.9857	0.244819	-0.535165	3.04315	0.204 (51)
4	1.86491	10.3563	15.4641	18.7048	0.643 (50)
7	0.578416	0.365261	0.0485798	0.685814	0.093 (24)
8	-0.442429	2.69818	-0.132356	2.73741	0.351 (32)
10	-2.75649	1.87031	2.26078	4.02584	0.510 (42)
11	-0.736145	-4.33894	-0.84998	4.48228	0.275 (36)
12	-3.74084	0.585224	0.395826	3.80697	0.415 (26)
13	-2.69329	1.53088	0.464151	3.13255	0.184 (20)
14	-0.195615	-0.383024	0.0601985	0.434277	0.074 (23)
16	-0.567448	0.405901	0.084221	0.702742	0.090 (34)
17	0.475172	-0.0390309	-0.0684393	0.48166	0.073 (23)
18	0.498954	-0.42535	-0.0584473	0.65825	0.114 (25)
19	-0.915752	-0.255364	-0.0916655	0.955099	0.105 (20)
20	-0.0439789	-0.576176	0.0378668	0.579092	0.125 (16)
21	1.21098	-0.94685	0.133218	1.54296	0.146 (15)
22	0.377167	1.59524	-0.083025	1.64133	0.126 (13)
23	1.21715	-1.44816	0.163038	1.89874	0.139 (18)
24	-3.72706	2.66367	-0.50805	4.60915	0.337 (27)
26	0.119191	1.38206	-0.59441	1.50917	0.132 (33)
27	0.321002	-1.53237	0.0571022	1.56667	0.219 (27)
32	-0.260551	0.383017	-0.00123408	0.463239	0.069 (18)
33	0.0105862	0.00161381	-0.00745488	0.0130479	0.001 (3)
34	-0.00969125	0.0703381	-0.018235	0.0733068	0.014 (4)
35	0.0381852	2.17321	-0.419221	2.21361	0.263 (11)
37	-0.00430901	-0.933823	0.27288	0.972886	0.122 (46)
38	0.887938	2.80165	-0.48667	2.97901	0.180 (57)
40	-0.813841	-2.87543	-1.34201	3.27589	0.275 (66)
43	-0.189694	-4.82941	0.295561	4.84216	0.496 (69)
48	-1.97863	0.136106	-0.546393	2.05719	0.195 (44)

Label	X error (cm)	Y error (cm)	Z error (cm)	Total (cm)	Image (pix)
56	0.794701	-1.0943	-0.100379	1.35614	0.311 (50)
57	13.4178	-6.41228	-1.55474	14.9523	1.489 (14)
58	-6.82634	0.899695	-1.07891	6.96939	0.875 (30)
60	-1.96548	1.53846	0.191525	2.50333	0.173 (32)
61	1.99499	-0.721353	0.629751	2.2129	0.144 (20)
62	-0.798913	-0.0982448	0.226448	0.836177	0.148 (17)
63	4.01345	-0.188779	-0.284272	4.02793	0.317 (16)
70	-1.41787	-0.0717954	-0.996332	1.73442	0.110 (30)
72	3.09613	-3.88608	-3.38867	6.01421	0.279 (18)
73	0.0520962	-0.108451	0.252352	0.279566	0.064 (15)
76	0.594709	0.157634	-0.0258868	0.61579	0.094 (9)
77	0.813594	-0.62935	0.0915841	1.03267	0.088 (11)
78	0.432588	-1.55256	-0.164945	1.62012	0.117 (11)
80	-0.0098439	0.337529	0.0228569	0.338445	0.089 (7)
82	-1.23234	-0.657487	-0.275791	1.42373	0.208 (6)
83	-2.18996	-0.655329	-0.167282	2.29203	0.201 (13)
85	0.43752	0.046588	-0.342466	0.557563	0.206 (10)
91	-0.606268	-0.498208	0.0428734	0.785882	0.235 (20)
93	-0.233398	0.564162	-0.111303	0.620597	0.176 (16)
97	1.8152	-2.87609	0.14569	3.40412	0.370 (40)
100	2.38683	0.539542	-3.46026	4.2381	0.834 (28)
104	-5.99292	10.3613	5.65254	13.2372	1.074 (21)
107	0.735911	-1.17603	-0.472721	1.46564	0.103 (21)
109	0.872869	0.804372	2.19866	2.4986	0.267 (22)
**Total**	**2.81887**	**3.00811**	**2.82591**	**4.99805**	**0.401**

Table 10. Control points.
X - Easting, Y - Northing, Z - Altitude.

Label	X error (cm)	Y error (cm)	Z error (cm)	Total (cm)	Image (pix)
5	-32.0651	102.424	28.4548	111.034	0.629 (38)
6	10.2175	-40.7541	-32.9359	53.386	0.302 (66)
9	16.1327	8.96322	-40.4194	44.4334	0.248 (29)

Label	X error (cm)	Y error (cm)	Z error (cm)	Total (cm)	Image (pix)
15	-1.91994	1.44622	2.20762	3.26363	0.118 (27)
25	2.06437	2.39488	5.24132	6.12115	0.108 (22)
28	0.165806	-2.38745	-7.0626	7.45706	0.166 (29)
29	2.50121	12.3258	-54.5871	56.0173	0.030 (18)
30	-0.47682	1.32776	0.987085	1.72181	0.103 (20)
31	1.48501	-0.0696428	7.92048	8.05879	0.135 (26)
36	-9.54215	9.71286	4.1071	14.2218	0.136 (14)
39	8.48405	-11.8665	-6.02754	15.7837	0.208 (40)
41	1.19675	2.04212	-9.89851	10.1776	0.407 (56)
42	2.69743	-2.29859	-10.6073	11.1837	0.380 (48)
44	-6.39616	0.572545	-5.60409	8.52317	0.608 (73)
45	-0.918547	3.20896	-8.85545	9.46362	0.562 (94)
46	6.64442	1.19419	-17.6189	18.868	0.416 (63)
47	2.64782	5.08078	-14.8033	15.8734	0.488 (107)
49	25.9561	-55.7598	-194.889	204.364	0.796 (59)
50	7.98054	-9.29372	-35.9611	37.9903	0.431 (56)
51	2.99902	0.879633	-1.72993	3.57219	0.353 (42)
52	0.153609	-12.3044	-23.4555	26.4873	0.313 (56)
54	11.9256	0.37023	1.42387	12.016	0.124 (31)
55	-3.6103	8.85272	-0.960543	9.60872	0.348 (51)
59	-2.13374	-9.22342	11.3263	14.7617	0.267 (9)
64	-14.5528	-14.8838	1.1049	20.8454	0.293 (13)
65	-11.4751	0.612214	2.30942	11.7212	0.343 (31)
66	1.60445	6.03337	45.6191	46.0443	0.173 (22)
67	0.345418	9.33354	38.043	39.1727	0.168 (12)
68	-3.83345	8.03	17.7897	19.8909	0.138 (16)
69	6.66691	-12.0637	6.23642	15.1286	0.219 (28)
71	-14.4017	-5.2921	-4.8276	16.0848	0.237 (24)
74	2.90879	-5.91773	7.04032	9.64607	0.097 (10)
75	-2.00008	6.83988	2.93473	7.70694	0.026 (6)
79	-8.89478	2.74062	18.1093	20.3611	0.140 (8)
81	-4.20007	-2.16091	-9.02294	10.1845	0.240 (6)

Label	X error (cm)	Y error (cm)	Z error (cm)	Total (cm)	Image (pix)
84	6.93494	3.6714	-4.19188	8.89632	0.181 (12)
87	-0.0758457	-1.99718	-19.7386	19.8395	0.249 (10)
88	-2.69771	-1.95374	-21.9528	22.2041	0.252 (13)
89	0.228206	-7.29144	-14.6185	16.3376	0.172 (27)
90	1.63903	0.750977	1.69945	2.4776	0.158 (28)
92	0.05119	0.798282	2.12827	2.27363	0.228 (18)
94	-5.33067	-3.43457	-26.0235	26.7849	0.162 (35)
95	-4.50186	-1.17453	-0.677996	4.70169	0.259 (38)
96	-4.16624	2.51111	-2.40092	5.42472	0.248 (23)
98	16.2485	-2.1902	-1.77193	16.491	0.586 (32)
99	29.0144	-5.11341	-14.6323	32.895	0.598 (30)
101	-16.6736	8.00711	-2.14901	18.621	0.672 (26)
102	1.18812	17.3793	-25.3288	30.7409	0.368 (21)
103	-8.65173	11.4115	-19.6421	24.3082	0.479 (20)
105	-4.55611	19.6624	-15.6304	25.528	0.196 (24)
106	-14.3876	-2.89417	19.1607	24.1353	0.087 (13)
108	-0.255292	-1.16494	3.7531	3.93802	0.175 (19)
110	-2.473	2.82563	-6.0791	7.1453	0.266 (28)
111	1.41834	-5.3292	4.29179	6.98796	0.143 (14)
**Total**	**9.7397**	**18.2196**	**32.094**	**38.1685**	**0.397**

Table 11. Check points.

X - Easting, Y - Northing, Z - Altitude.

Digital Elevation Model

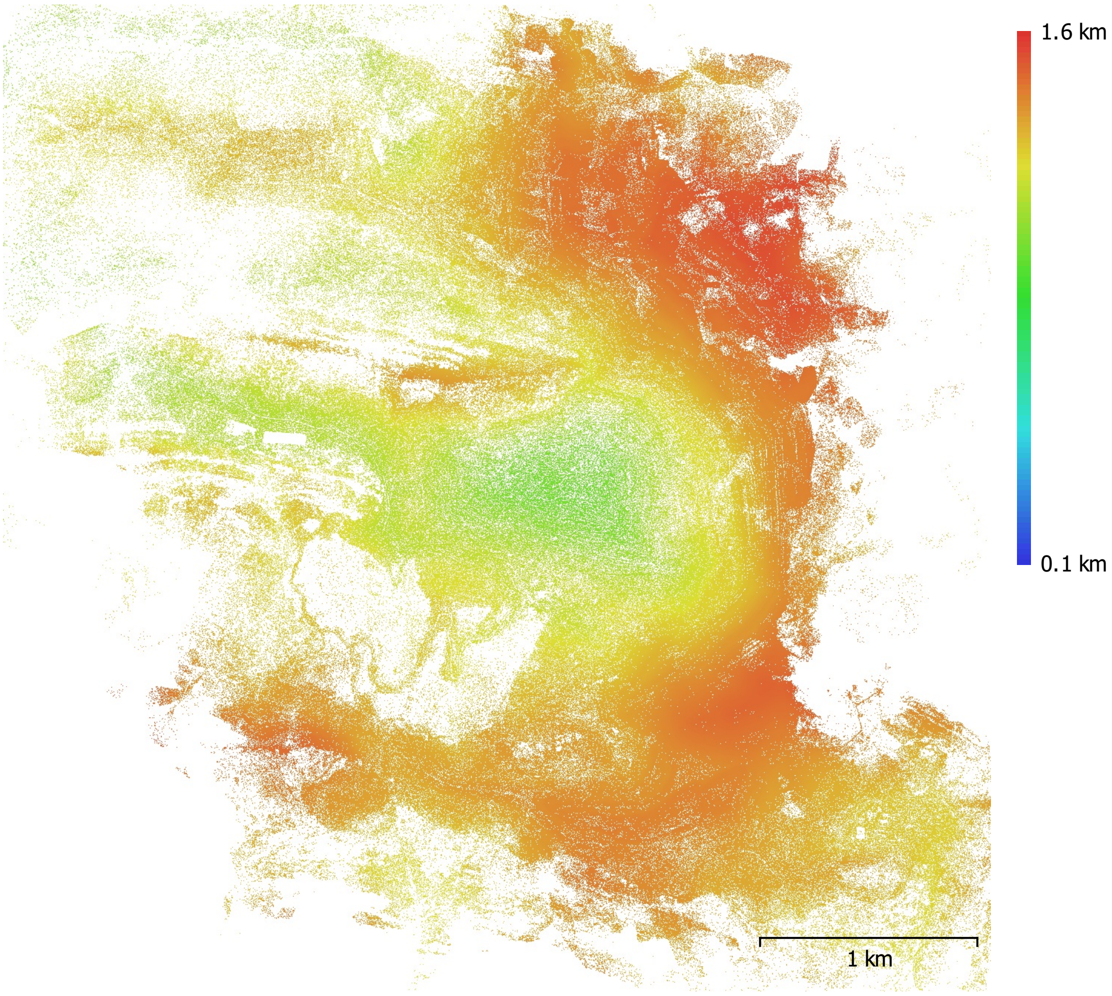

Fig. 9. Reconstructed digital elevation model.

Resolution:	unknown
Point density:	unknown

Processing Parameters

**General**	
Cameras	2595
Aligned cameras	2577
Markers	110
**Shapes**	
Polygon	1
Coordinate system	ETRS89 / UTM zone 30N (EPSG::25830)
Rotation angles	Yaw, Pitch, Roll
**Tie Points**	
Points	1,775,103 of 12,529,745
RMS reprojection error	0.140134 (0.329735 pix)
Max reprojection error	0.300298 (1.66876 pix)
Mean key point size	2.31318 pix
Point colors	3 bands, uint8
Key points	No
Average tie point multiplicity	3.65511
**Alignment parameters**	
Accuracy	High
Generic preselection	Yes
Reference preselection	No
Key point limit	60,000
Key point limit per Mpx	1,000
Tie point limit	0
Exclude stationary tie points	Yes
Guided image matching	No
Adaptive camera model fitting	No
Matching time	4 hours 7 minutes
Matching memory usage	3.73 GB
Alignment time	2 hours 17 minutes
Alignment memory usage	4.82 GB
**Optimization parameters**	
Parameters	f, cx, cy, k1-k3, p1, p2
Adaptive camera model fitting	No
Optimization time	4 minutes 46 seconds
Date created	2023:11:13 15:04:46
Software version	2.0.0.15597
File size	775.63 MB
**System**	
Software name	Agisoft Metashape Professional
Software version	2.0.3 build 16960
OS	Windows 64 bit
RAM	63.90 GB
CPU	Intel(R) Core(TM) i7-7700 CPU @ 3.60GHz
GPU(s)	Quadro M4000
