# Supplementary material for: Automated cleaning of tie point clouds following USGS guidelines in Agisoft Metashape professional (ver. 2.1.0)
Source: MethodsX. 2024 Mar 26;12:102679. doi: 10.1016/j.mex.2024.102679 (PMC10992719; doi:10.1016/j.mex.2024.102679)
Supplement: Supplementary file 3 — The supplementary material includes supplementary text, figures and the processing reports generated by the software. [file mmc3.zip › Lucia_SCC-RMSEm_r1.pdf]

Lucia_SCC-RMSEm_r1

Automatically cleaned sparse cloud using the SCC script (aiming for minimizing the unweighted RMS reprojection error). UAS data provided by Sanz-Ablanedo et al. (2018).

Sanz-Ablanedo, E., Chandler, J. H., Rodríguez-Pérez, J. R., and Ordóñez, C.: Accuracy of Unmanned Aerial Vehicle (UAV) and SfM Photogrammetry Survey as a Function of the Number and Location of Ground Control Points Used, Remote Sensing, 10, 1606, 2018.

28 December 2023

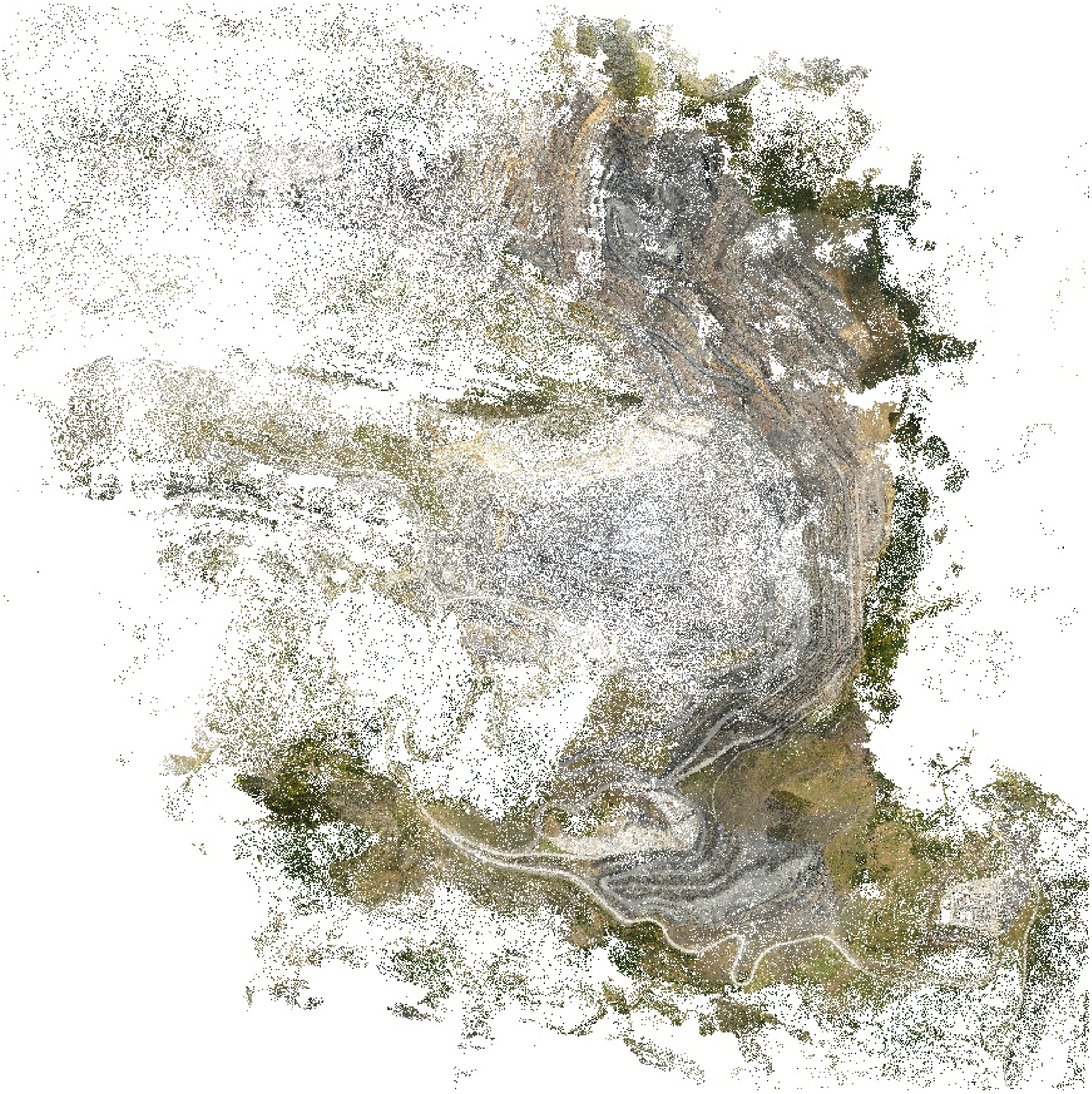

Survey Data

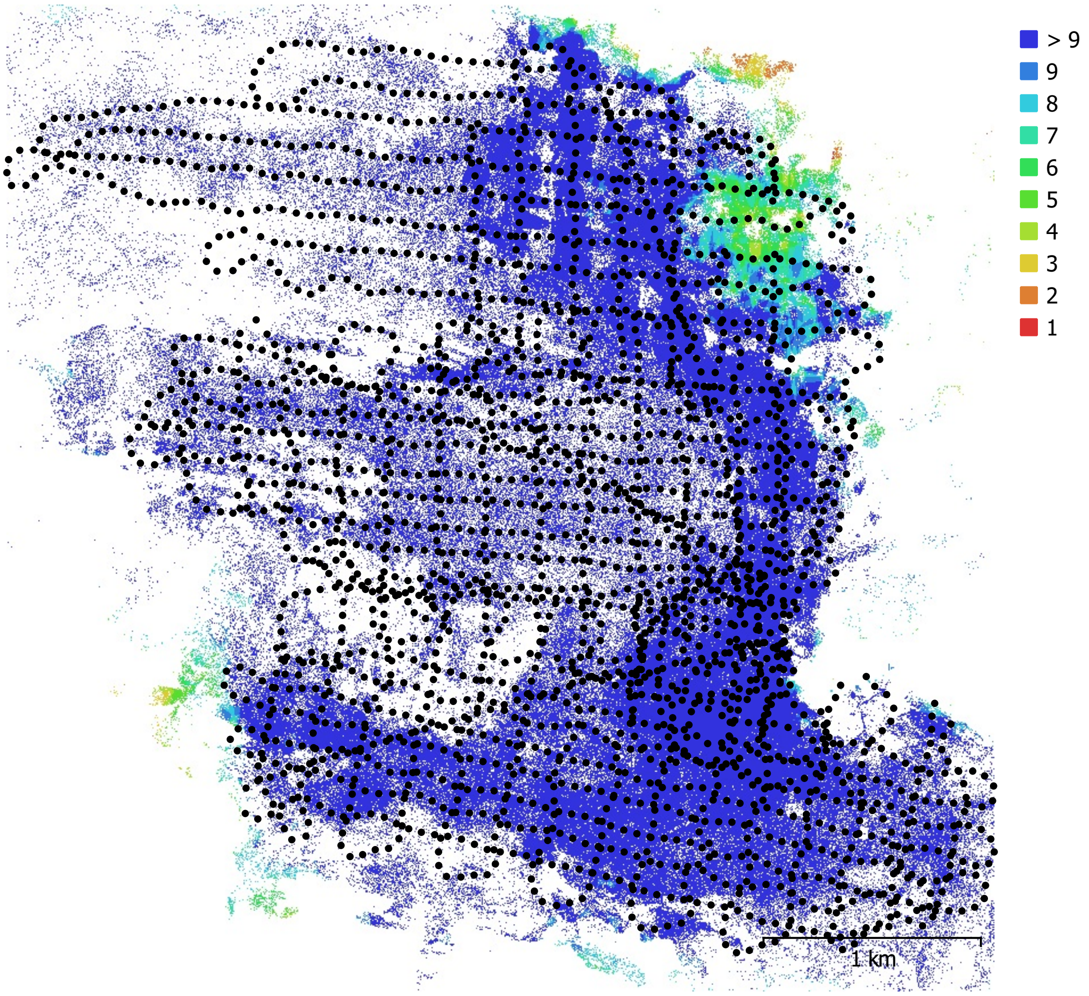

Fig. 1. Camera locations and image overlap.

Number of images:	2,595	Camera stations:	2,575
Flying altitude:	350 m	Tie points:	753,153
Ground resolution:	6.23 cm/pix	Projections:	1,547,655
Coverage area:	6.87 km²	Reprojection error:	0.18 pix

Camera Model	Resolution	Focal Length	Pixel Size	Precalibrated
NX500 (20 mm)	6480 x 4320	20 mm	3.7 x 3.7 μm	No
NX500 (20 mm)	6480 x 4320	20 mm	3.7 x 3.7 μm	No
NX500 (20 mm)	6480 x 4320	20 mm	3.7 x 3.7 μm	No
NX500 (20 mm)	6480 x 4320	20 mm	3.7 x 3.7 μm	No
NX500 (20 mm)	6480 x 4320	20 mm	3.7 x 3.7 μm	No

Camera Model	Resolution	Focal Length	Pixel Size	Precalibrated
NX500 (20 mm)	6480 x 4320	20 mm	3.7 x 3.7 μm	No

Table 1. Cameras.

Camera Calibration

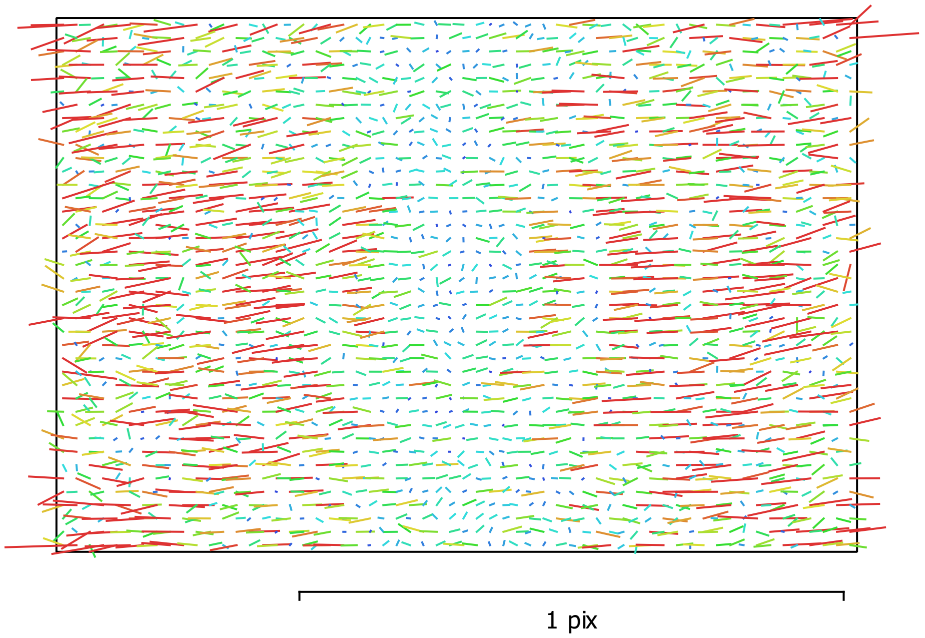

Fig. 2. Image residuals for NX500 (20 mm).

NX500 (20 mm)

200 images, additional corrections

Type	Resolution	Focal Length	Pixel Size
**Frame**	**6480 x 4320**	**20 mm**	**3.7 x 3.7 μm**

F:	5619.42		
Cx:	87.1105	B1:	3.31845
Cy:	20.6795	B2:	0.881204
K1:	-0.0129719	P1:	0.00222237
K2:	0.0298972	P2:	-0.000638762
K3:	-0.0171629	P3:	0
K4:	-0.019295	P4:	0

Camera Calibration

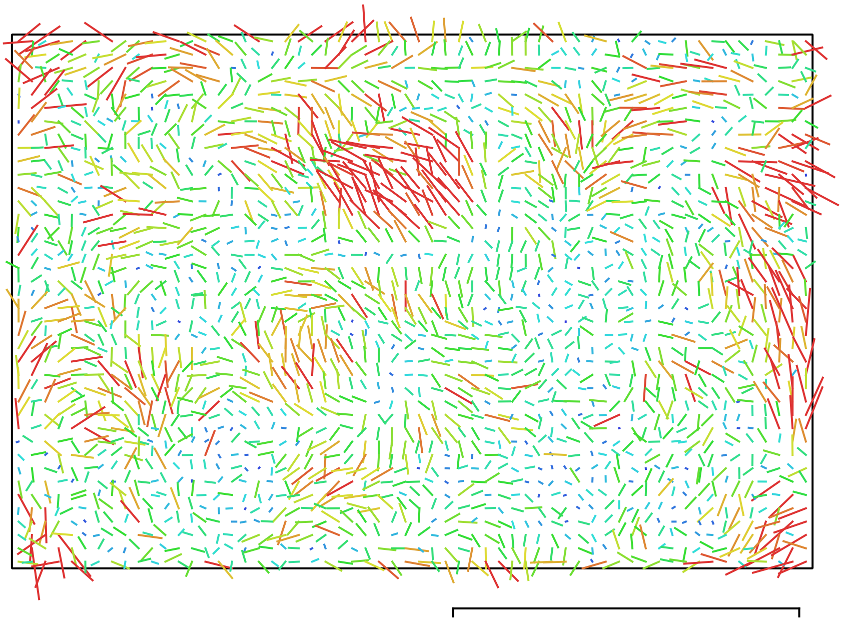

Fig. 3. Image residuals for NX500 (20 mm).

NX500 (20 mm)

462 images, additional corrections

Type	Resolution	Focal Length	Pixel Size
**Frame**	**6480 x 4320**	**20 mm**	**3.7 x 3.7 µm**

F:	5616.22		
Cx:	80.785	B1:	-1.18806
Cy:	28.8498	B2:	-0.655876
K1:	0.043621	P1:	0.00278557
K2:	-0.36658	P2:	-0.000677628
K3:	0.9929	P3:	0
K4:	-0.890621	P4:	0

Camera Calibration

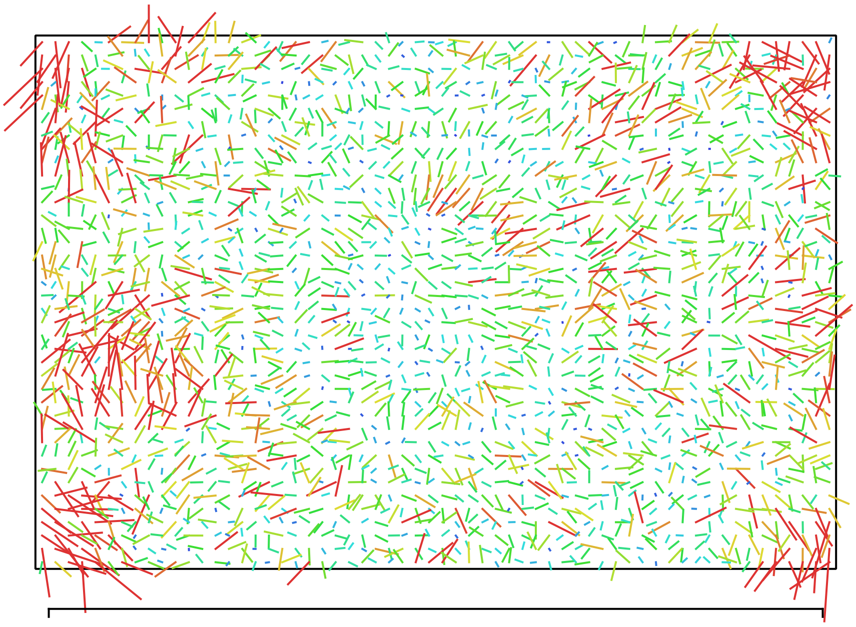

Fig. 4. Image residuals for NX500 (20 mm).

NX500 (20 mm)

530 images, additional corrections

Type	Resolution	Focal Length	Pixel Size
**Frame**	**6480 x 4320**	**20 mm**	**3.7 x 3.7 µm**

F:	5631.95		
Cx:	78.2043	B1:	0.419574
Cy:	35.7424	B2:	0.193962
K1:	-0.0237645	P1:	0.00204745
K2:	0.11398	P2:	0.000970156
K3:	-0.257806	P3:	0
K4:	0.204582	P4:	0

Camera Calibration

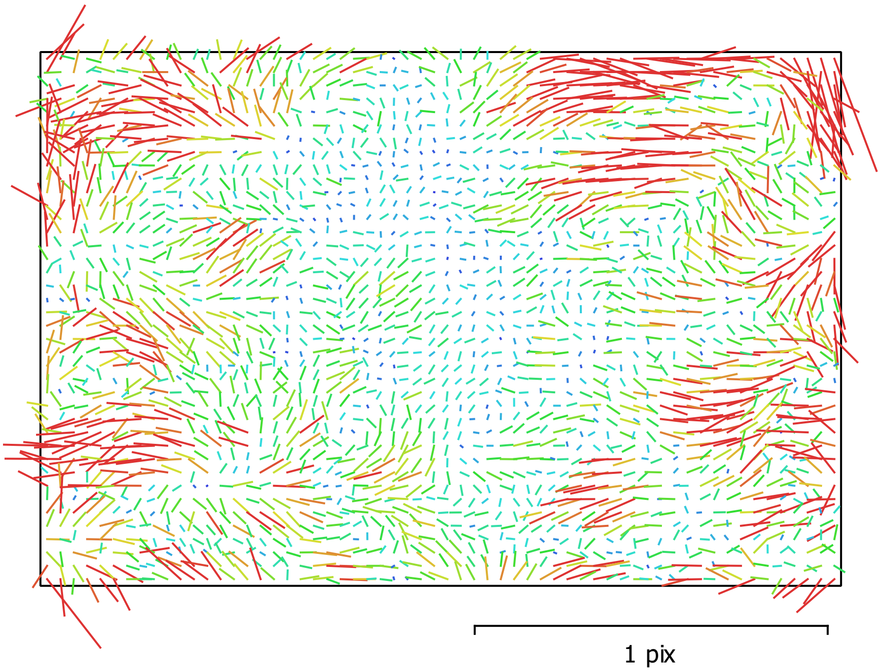

Fig. 5. Image residuals for NX500 (20 mm).

NX500 (20 mm)

513 images, additional corrections

Type	Resolution	Focal Length	Pixel Size
**Frame**	**6480 x 4320**	**20 mm**	**3.7 x 3.7 µm**

F:	5622.39		
Cx:	87.9376	B1:	-1.71079
Cy:	80.5573	B2:	-0.317096
K1:	-0.0308976	P1:	0.00227123
K2:	0.130815	P2:	0.00257426
K3:	-0.305124	P3:	0
K4:	0.300818	P4:	0

Camera Calibration

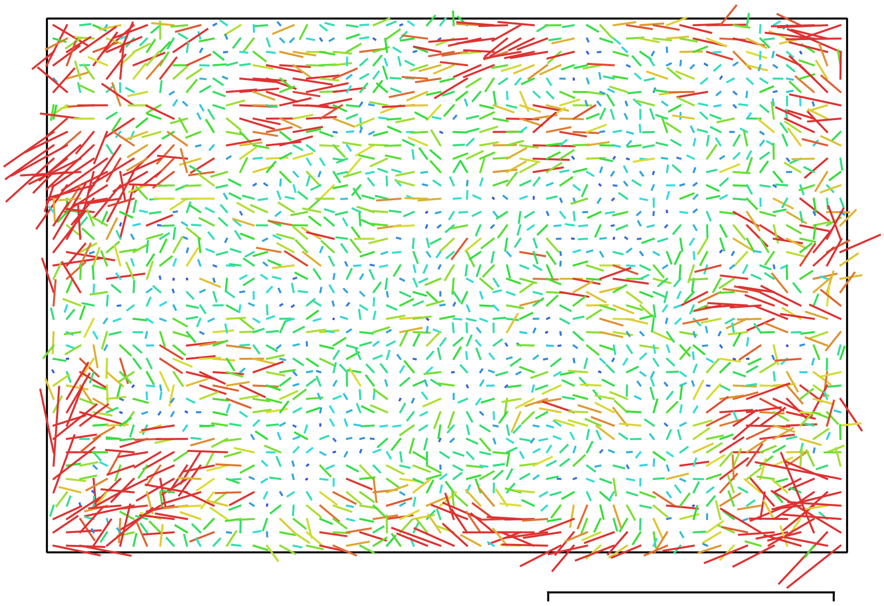

Fig. 6. Image residuals for NX500 (20 mm).

NX500 (20 mm)

412 images, additional corrections

Type	Resolution	Focal Length	Pixel Size
**Frame**	**6480 x 4320**	**20 mm**	**3.7 x 3.7 µm**

F:	5620.58		
Cx:	102.818	B1:	5.22234
Cy:	65.9774	B2:	-0.467427
K1:	-0.0189718	P1:	0.00380592
K2:	0.145484	P2:	0.00221089
K3:	-0.466417	P3:	0
K4:	0.462225	P4:	0

Camera Calibration

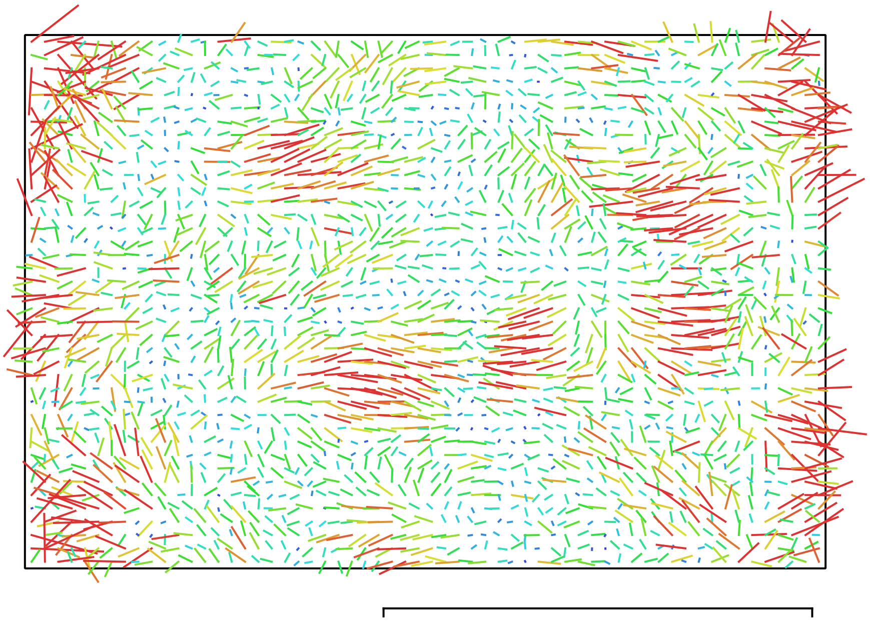

Fig. 7. Image residuals for NX500 (20 mm).

NX500 (20 mm)

478 images, additional corrections

Type	Resolution	Focal Length	Pixel Size
**Frame**	**6480 x 4320**	**20 mm**	**3.7 x 3.7 μm**

F:	5625.55		
Cx:	83.7065	B1:	4.54009
Cy:	28.6251	B2:	-0.847706
K1:	0.0143161	P1:	0.00338475
K2:	-0.195583	P2:	-0.000470534
K3:	0.712489	P3:	0
K4:	-0.826594	P4:	0

Ground Control Points

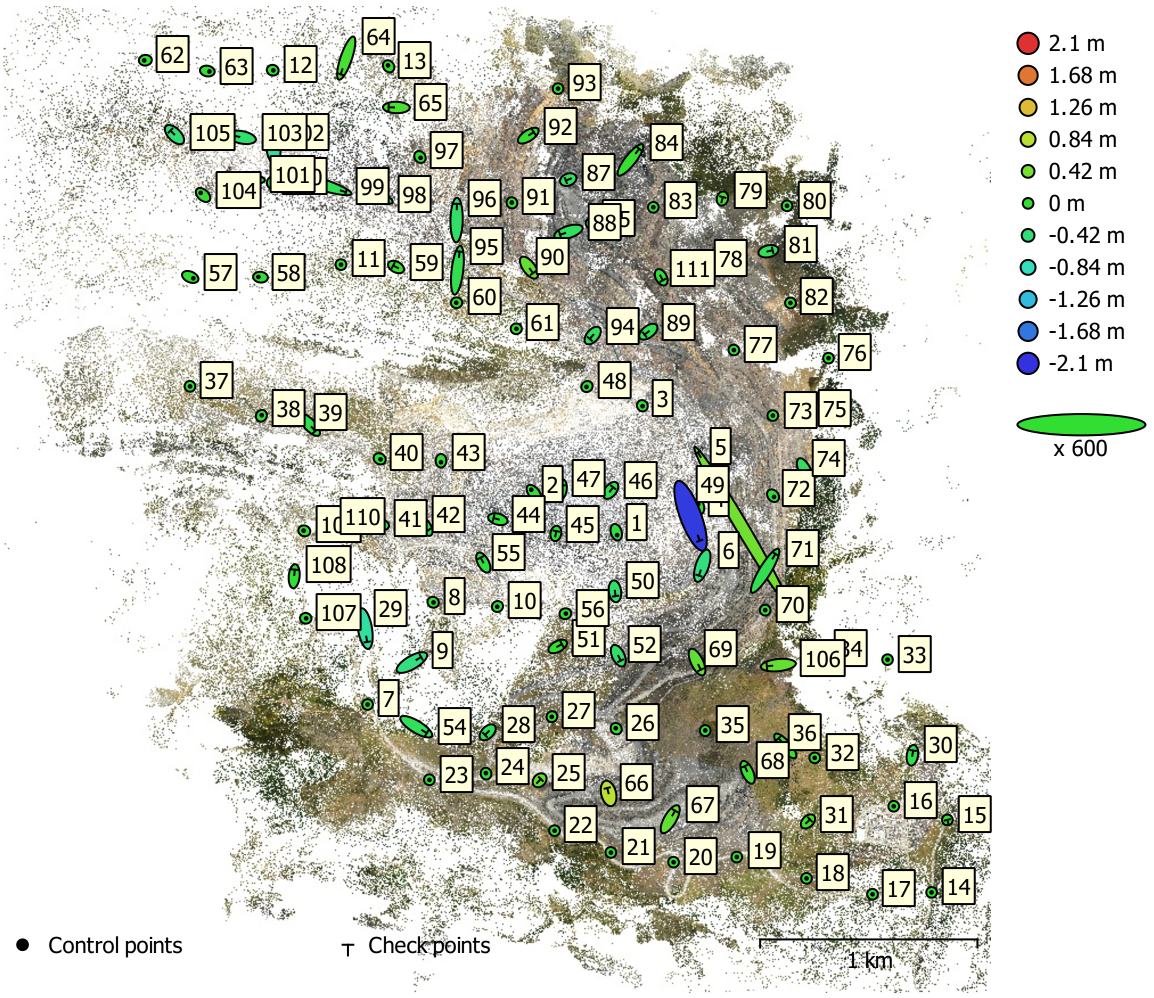

Fig. 8. GCP locations and error estimates.
Z error is represented by ellipse color. X,Y errors are represented by ellipse shape.
Estimated GCP locations are marked with a dot or crossing.

Count	X error (cm)	Y error (cm)	Z error (cm)	XY error (cm)	Total (cm)
55	1.25	1.26457	0.997333	1.7781	2.0387

Table 2. Control points RMSE.
X - Easting, Y - Northing, Z - Altitude.

Count	X error (cm)	Y error (cm)	Z error (cm)	XY error (cm)	Total (cm)
54	13.8196	19.2585	38.9392	23.7039	45.5865

Table 3. Check points RMSE.
X - Easting, Y - Northing, Z - Altitude.

Label	X error (cm)	Y error (cm)	Z error (cm)	Total (cm)	Image (pix)
1	1.33844	-4.44656	-4.13938	6.22075	0.286 (104)
2	-3.84597	4.57147	0.0757888	5.97458	0.291 (109)
3	0.33335	-0.607458	0.206472	0.72302	0.037 (51)
4	-0.789267	3.89194	4.80878	6.23655	0.325 (50)
7	0.0365442	0.000774457	-0.0177817	0.0406481	0.006 (24)
8	0.610726	-0.332384	-0.417902	0.811238	0.180 (32)
10	-0.370143	-0.303211	1.59431	1.66456	0.222 (42)
11	0.13821	-0.124546	0.394059	0.435771	0.119 (36)
12	0.879176	-0.239245	-0.218591	0.937001	0.233 (26)
13	-0.945157	1.33113	0.299994	1.65988	0.138 (20)
14	-0.043765	-0.0362094	0.00482228	0.0570066	0.006 (23)
16	-0.0543333	-0.0054639	-0.00580713	0.0549152	0.012 (34)
17	0.0776563	0.000729009	-0.00243681	0.077698	0.006 (23)
18	0.0444048	-0.107805	-0.00109105	0.116597	0.012 (25)
19	0.103513	0.0912043	-0.0264198	0.140468	0.015 (20)
20	0.0600035	-0.0457405	-0.0520199	0.0916443	0.017 (16)
21	0.151876	-0.171731	0.077031	0.241851	0.022 (15)
22	0.10082	-0.052684	0.0540155	0.125928	0.018 (13)
23	0.103445	-0.174154	-0.00914325	0.202766	0.014 (18)
24	-0.179249	0.820018	-0.108092	0.846312	0.080 (27)
26	-0.597887	0.756418	-0.217147	0.988327	0.034 (33)
27	-0.249214	-0.253458	0.014599	0.355755	0.056 (27)
32	-0.0669258	0.0181588	-0.00166104	0.0693654	0.012 (18)
33	0.00112753	-0.000615163	-0.000357624	0.00133329	0.000 (3)
34	-0.00139132	0.0020331	0.00450574	0.00513527	0.001 (4)
35	0.0269635	0.236589	0.0152732	0.23861	0.032 (11)
37	0.0898981	-0.11496	0.47088	0.492976	0.059 (46)
38	0.516728	0.905305	-0.70937	1.26087	0.104 (57)
40	1.29409	-0.796586	-0.461953	1.58828	0.151 (66)
43	-0.0483185	-2.21219	-0.555624	2.28141	0.251 (69)
48	-0.664066	-0.498508	-0.17676	0.848963	0.024 (44)

Label	X error (cm)	Y error (cm)	Z error (cm)	Total (cm)	Image (pix)
56	0.825931	0.228187	0.287405	0.903788	0.214 (50)
57	4.67166	-2.03366	-0.613447	5.1319	0.488 (14)
58	-3.13951	0.346794	-0.371335	3.18036	0.260 (30)
60	-0.513409	-0.0522627	-0.17815	0.545947	0.038 (32)
61	0.391229	0.0278439	0.00927181	0.392328	0.030 (20)
62	-1.75	-0.172795	0.254769	1.77687	0.202 (17)
63	3.16613	-0.591008	0.0400305	3.22106	0.373 (16)
70	-0.0450032	0.123	-0.211454	0.248731	0.018 (30)
72	1.48126	-1.83052	-1.15621	2.62331	0.119 (18)
73	0.0300747	0.00993933	0.0289668	0.0429227	0.019 (15)
76	0.0235558	-0.0246799	-0.00165858	0.0341573	0.010 (9)
77	0.0889055	-0.0437585	0.0223349	0.101577	0.017 (11)
78	-0.104694	0.0095128	0.0214049	0.107282	0.054 (11)
80	0.00423124	0.0571645	-0.00970825	0.0581372	0.015 (7)
82	-0.130003	-0.0208826	-0.0184904	0.132961	0.032 (6)
83	0.0253952	0.0779519	-0.00440148	0.0821023	0.039 (13)
85	-0.0937018	0.0571737	0.0310651	0.114078	0.029 (10)
91	-0.242689	0.464324	0.126351	0.538943	0.038 (20)
93	0.0253726	0.0235778	-0.0463468	0.0578594	0.020 (16)
97	0.960251	-1.17649	-0.209785	1.53304	0.159 (40)
100	0.308877	-1.13561	-1.62078	2.00299	0.343 (28)
104	-3.57879	3.24287	2.16592	5.29294	0.574 (21)
107	0.606277	-0.123954	-0.0821274	0.624244	0.040 (21)
109	-1.06796	0.40508	0.756964	1.37026	0.109 (22)
**Total**	**1.25**	**1.26457**	**0.997333**	**2.0387**	**0.193**

Table 4. Control points.
X - Easting, Y - Northing, Z - Altitude.

Label	X error (cm)	Y error (cm)	Z error (cm)	Total (cm)	Image (pix)
5	-66.2609	112.073	39.8621	136.162	0.183 (38)
6	-5.17794	-15.1798	-45.7631	48.4923	0.084 (66)
9	14.293	8.32313	-46.8962	49.7274	0.090 (29)

Label	X error (cm)	Y error (cm)	Z error (cm)	Total (cm)	Image (pix)
15	-0.0270728	0.110045	0.0707825	0.133615	0.015 (27)
25	1.85739	1.88199	29.7366	29.8539	0.026 (22)
28	-4.14925	-4.06244	-22.4961	23.2334	0.034 (29)
29	3.38496	-20.4863	-67.3415	70.47	0.005 (18)
30	1.12465	7.33525	-11.5763	13.7507	0.007 (20)
31	3.10787	2.98653	8.68457	9.69535	0.014 (26)
36	-10.3651	11.8814	2.83571	16.0202	0.025 (14)
39	8.62325	-9.46263	-13.833	18.8482	0.113 (40)
41	5.21926	-1.18143	-12.3594	13.4682	0.176 (56)
42	4.90092	-7.17577	-15.4646	17.7388	0.176 (48)
44	-6.5708	1.79755	-6.68415	9.54382	0.318 (73)
45	0.692989	3.56144	-1.3987	3.8885	0.289 (94)
46	3.74608	4.80929	-18.7103	19.6783	0.217 (63)
47	1.47658	7.78987	-9.79754	12.6037	0.328 (107)
49	14.1658	-38.2405	-204.519	208.545	0.427 (59)
50	1.02172	-7.35451	-32.7539	33.585	0.198 (56)
51	6.52889	3.56253	-1.11945	7.52138	0.170 (42)
52	3.61997	-7.6305	-31.1579	32.2823	0.140 (56)
54	16.2001	-9.76633	-24.5699	31.0082	0.017 (31)
55	-4.08073	7.59128	-4.26165	9.61465	0.223 (51)
59	-4.33717	2.27898	1.96536	5.27896	0.107 (9)
64	-8.48037	-24.8123	13.5878	29.5329	0.167 (13)
65	-11.8545	0.242762	14.1739	18.4794	0.182 (31)
66	-2.07812	8.29388	82.9299	83.3695	0.032 (22)
67	7.49104	12.9475	29.6408	33.2013	0.026 (12)
68	-4.36464	9.43379	7.55356	12.8492	0.028 (16)
69	5.67178	-11.7734	28.147	31.0328	0.025 (28)
71	15.8343	25.904	-18.2865	35.442	0.084 (24)
74	5.01361	-10.5531	-25.2364	27.8097	0.018 (10)
75	5.03464	2.55522	-16.0617	17.0251	0.007 (6)
79	0.59061	2.26127	11.9892	12.2149	0.021 (7)
81	6.44702	1.50284	-22.3121	23.2734	0.058 (6)

Label	X error (cm)	Y error (cm)	Z error (cm)	Total (cm)	Image (pix)
84	13.4628	17.0438	3.85362	22.0588	0.020 (12)
87	-3.66361	-1.46777	-24.5493	24.8645	0.019 (10)
88	-12.8679	-4.16541	-26.2763	29.553	0.024 (13)
89	-5.74114	-4.45101	-20.8415	22.0713	0.021 (27)
90	6.14771	-8.43246	29.78	31.5555	0.023 (28)
92	8.3767	5.42301	2.57414	10.3055	0.020 (18)
94	-4.45662	-5.22419	-24.3897	25.3379	0.034 (35)
95	3.38283	28.6726	-17.5313	33.7773	0.062 (38)
96	0.467147	24.8146	-31.3306	39.9698	0.029 (23)
98	18.1369	-5.87286	-3.74396	19.4282	0.220 (32)
99	46.789	-14.8215	-20.142	53.0527	0.263 (30)
101	-22.6897	2.26074	-11.0397	25.334	0.437 (26)
102	-1.77271	14.4836	-64.7112	66.3359	0.346 (21)
103	-12.0351	2.33835	-35.2147	37.2879	0.448 (20)
105	-6.21693	6.18676	-38.0775	39.0746	0.305 (24)
106	-17.576	-1.78736	29.8488	34.6852	0.005 (13)
108	0.918587	10.0705	-4.33695	11.0031	0.070 (19)
110	-8.03821	11.2588	-23.1646	26.981	0.083 (28)
111	2.49131	-4.56742	-5.63658	7.67066	0.024 (14)
**Total**	**13.8196**	**19.2585**	**38.9392**	**45.5865**	**0.208**

Table 5. Check points.

X - Easting, Y - Northing, Z - Altitude.

Digital Elevation Model

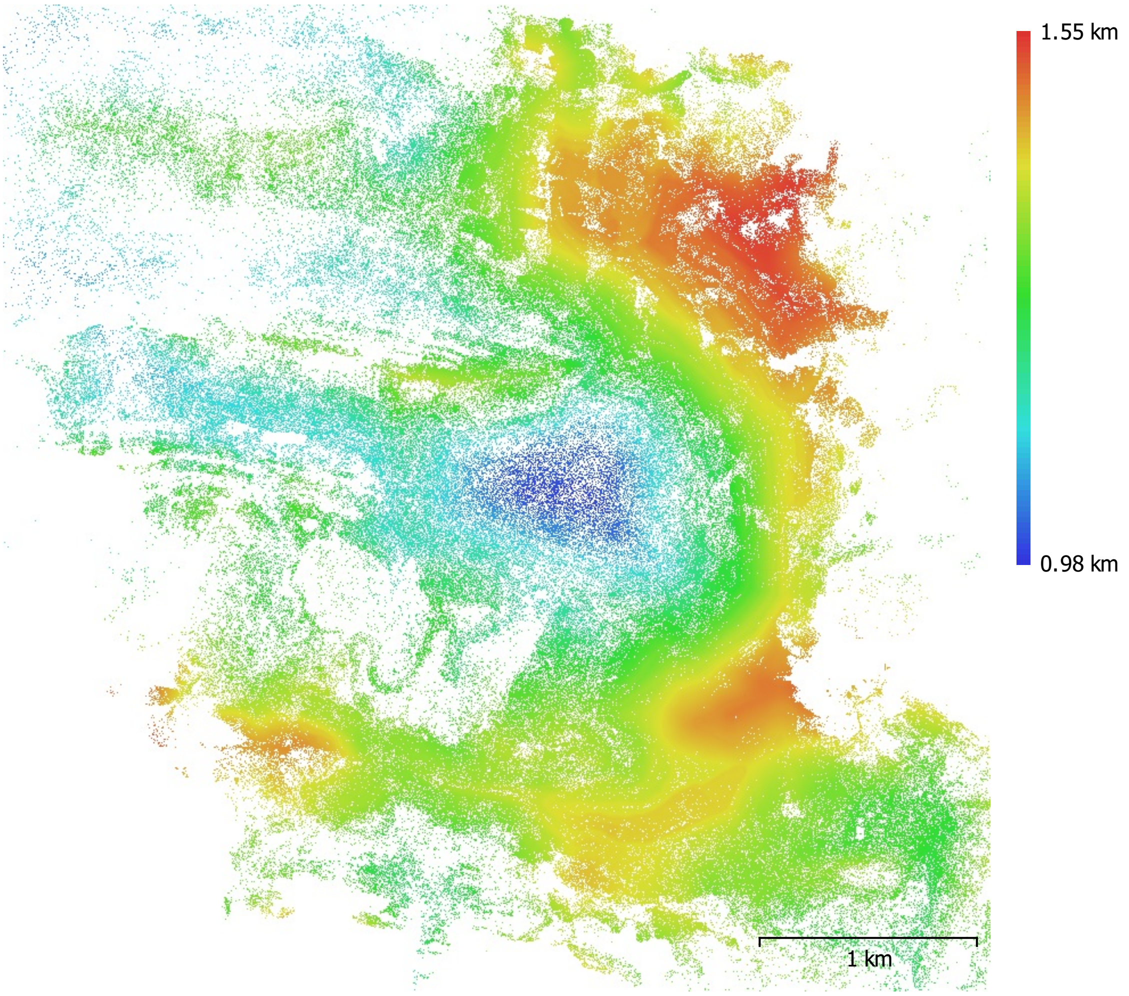

Fig. 9. Reconstructed digital elevation model.

Resolution:	unknown
Point density:	unknown

Processing Parameters

**General**	
Cameras	2595
Aligned cameras	2575
Markers	110
**Shapes**	
Polygon	1
Coordinate system	ETRS89 / UTM zone 30N (EPSG::25830)
Rotation angles	Yaw, Pitch, Roll
**Tie Points**	
Points	753,153 of 12,529,745
RMS reprojection error	0.077046 (0.179607 pix)
Max reprojection error	1.0602 (2.28338 pix)
Mean key point size	2.29929 pix
Point colors	3 bands, uint8
Key points	No
Average tie point multiplicity	3.65511
**Alignment parameters**	
Accuracy	High
Generic preselection	Yes
Reference preselection	No
Key point limit	60,000
Key point limit per Mpx	1,000
Tie point limit	0
Exclude stationary tie points	Yes
Guided image matching	No
Adaptive camera model fitting	No
Matching time	4 hours 7 minutes
Matching memory usage	3.73 GB
Alignment time	2 hours 17 minutes
Alignment memory usage	4.82 GB
**Optimization parameters**	
Parameters	f, b1, b2, cx, cy, k1-k4, p1, p2
Fit additional corrections	Yes
Adaptive camera model fitting	No
Optimization time	8 minutes 18 seconds
Date created	2023:11:13 15:04:46
Software version	2.0.0.15597
File size	752.24 MB
**System**	
Software name	Agisoft Metashape Professional
Software version	2.0.3 build 16960
OS	Windows 64 bit
RAM	63.90 GB
CPU	Intel(R) Core(TM) i7-7700 CPU @ 3.60GHz
GPU(s)	Quadro M4000
