# Supplementary material for: Automated cleaning of tie point clouds following USGS guidelines in Agisoft Metashape professional (ver. 2.1.0)
Source: MethodsX. 2024 Mar 26;12:102679. doi: 10.1016/j.mex.2024.102679 (PMC10992719; doi:10.1016/j.mex.2024.102679)
Supplement: Supplementary file 3 — The supplementary material includes supplementary text, figures and the processing reports generated by the software. [file mmc3.zip › Lucia_SCC-RMSEm_r2.pdf]

Lucia_SCC-RMSEm_r2

Automatically cleaned sparse cloud using the SCC script (aiming for minimizing the unweighted RMS reprojection error). UAS data provided by Sanz-Ablanedo et al. (2018).

Sanz-Ablanedo, E., Chandler, J. H., Rodríguez-Pérez, J. R., and Ordóñez, C.: Accuracy of Unmanned Aerial Vehicle (UAV) and SfM Photogrammetry Survey as a Function of the Number and Location of Ground Control Points Used, Remote Sensing, 10, 1606, 2018.

28 December 2023

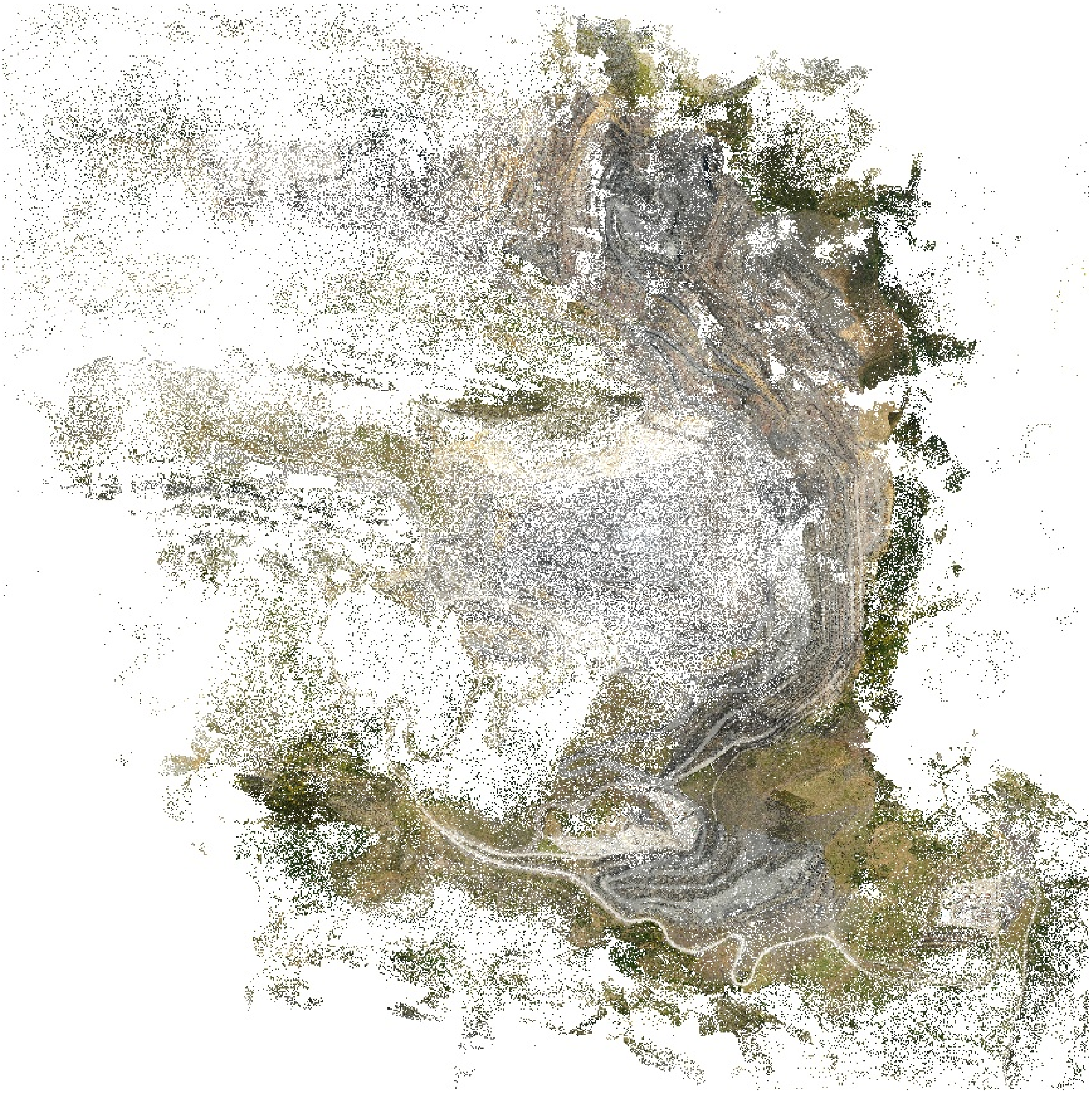

Survey Data

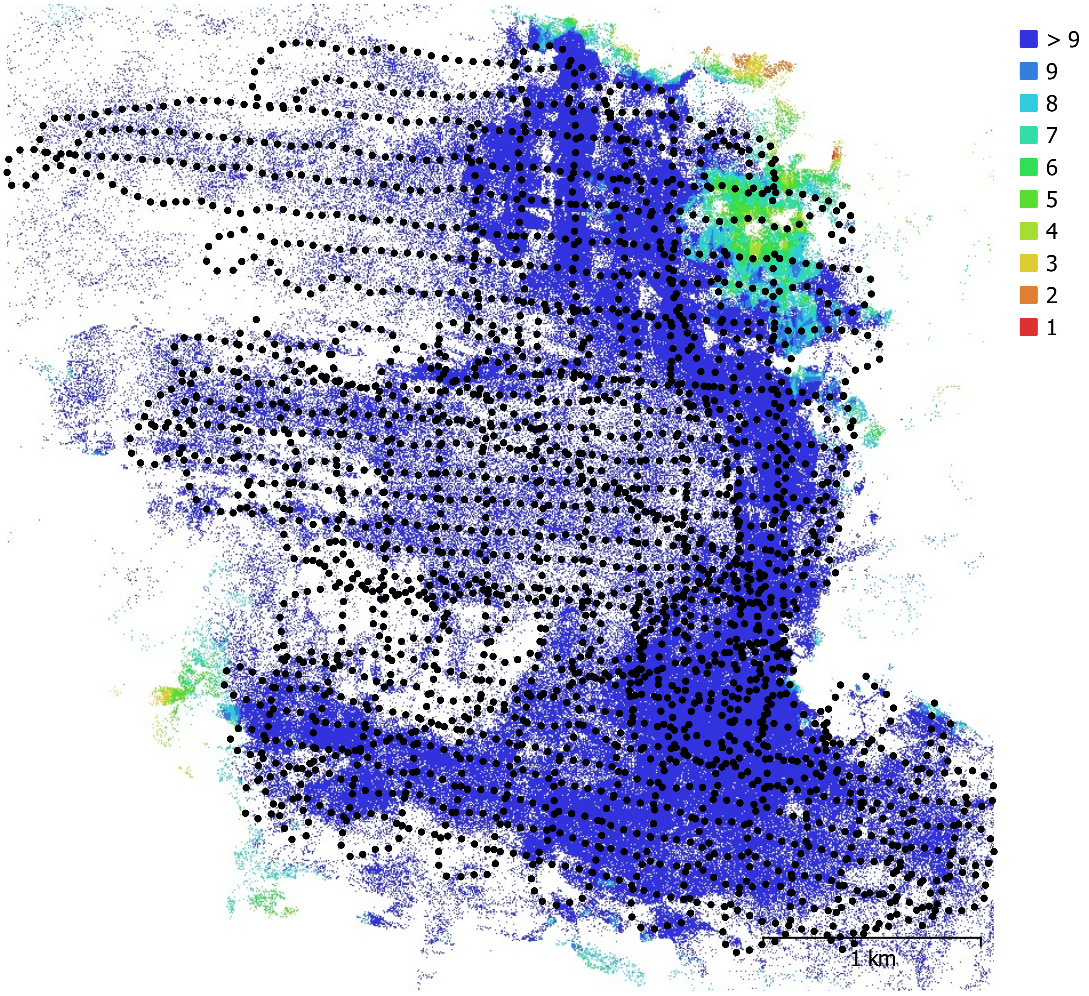

Fig. 1. Camera locations and image overlap.

Number of images:	2,595	Camera stations:	2,575
Flying altitude:	350 m	Tie points:	747,676
Ground resolution:	6.22 cm/pix	Projections:	1,535,681
Coverage area:	6.86 km²	Reprojection error:	0.179 pix

**Camera Model**	**Resolution**	**Focal Length**	**Pixel Size**	**Precalibrated**
NX500 (20 mm)	6480 x 4320	20 mm	3.7 x 3.7 μm	No
NX500 (20 mm)	6480 x 4320	20 mm	3.7 x 3.7 μm	No
NX500 (20 mm)	6480 x 4320	20 mm	3.7 x 3.7 μm	No
NX500 (20 mm)	6480 x 4320	20 mm	3.7 x 3.7 μm	No
NX500 (20 mm)	6480 x 4320	20 mm	3.7 x 3.7 μm	No

Camera Model	Resolution	Focal Length	Pixel Size	Precalibrated
NX500 (20 mm)	6480 x 4320	20 mm	3.7 x 3.7 μm	No

Table 1. Cameras.

Camera Calibration

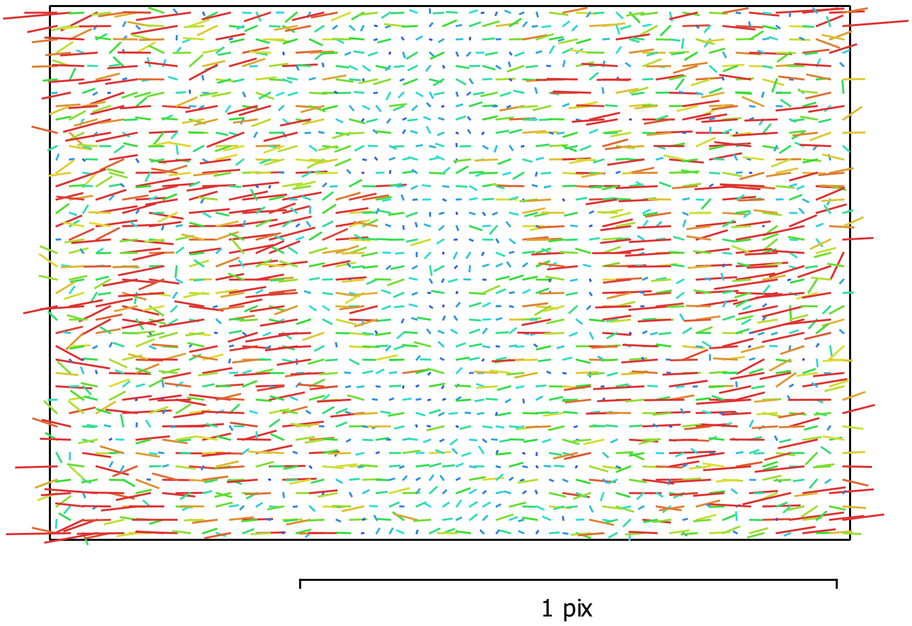

Fig. 2. Image residuals for NX500 (20 mm).

NX500 (20 mm)

200 images, additional corrections

Type	Resolution	Focal Length	Pixel Size
**Frame**	**6480 x 4320**	**20 mm**	**3.7 x 3.7 μm**

F:	5620.05		
Cx:	87.4689	B1:	3.2441
Cy:	21.4761	B2:	0.890924
K1:	-0.0148429	P1:	0.00224409
K2:	0.0386423	P2:	-0.000571485
K3:	-0.0350027	P3:	0
K4:	-0.00564641	P4:	0

Camera Calibration

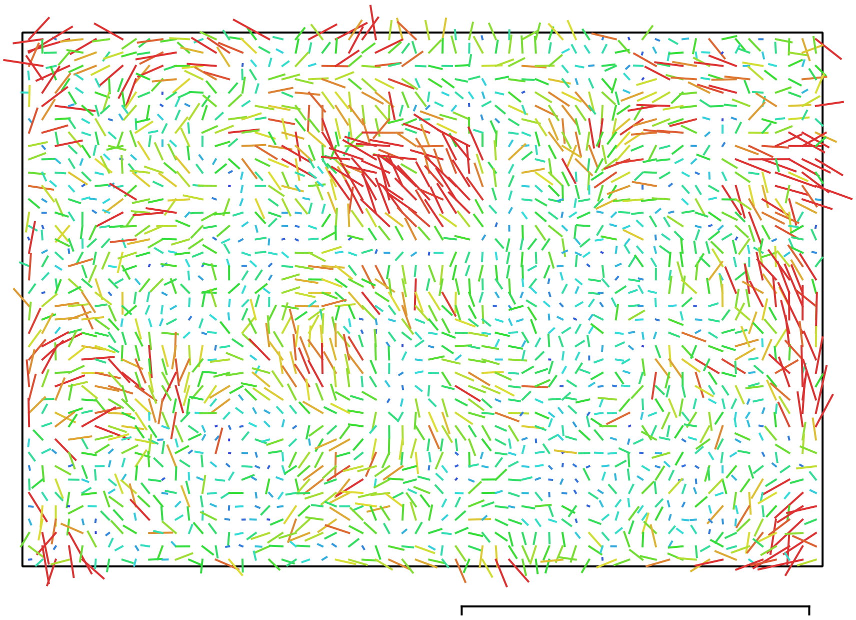

Fig. 3. Image residuals for NX500 (20 mm).

NX500 (20 mm)

462 images, additional corrections

Type	Resolution	Focal Length	Pixel Size
**Frame**	**6480 x 4320**	**20 mm**	**3.7 x 3.7 µm**

F:	5618.52		
Cx:	81.0126	B1:	-1.21253
Cy:	28.5887	B2:	-0.644216
K1:	0.0390299	P1:	0.00281217
K2:	-0.346164	P2:	-0.000705567
K3:	0.952402	P3:	0
K4:	-0.860613	P4:	0

Camera Calibration

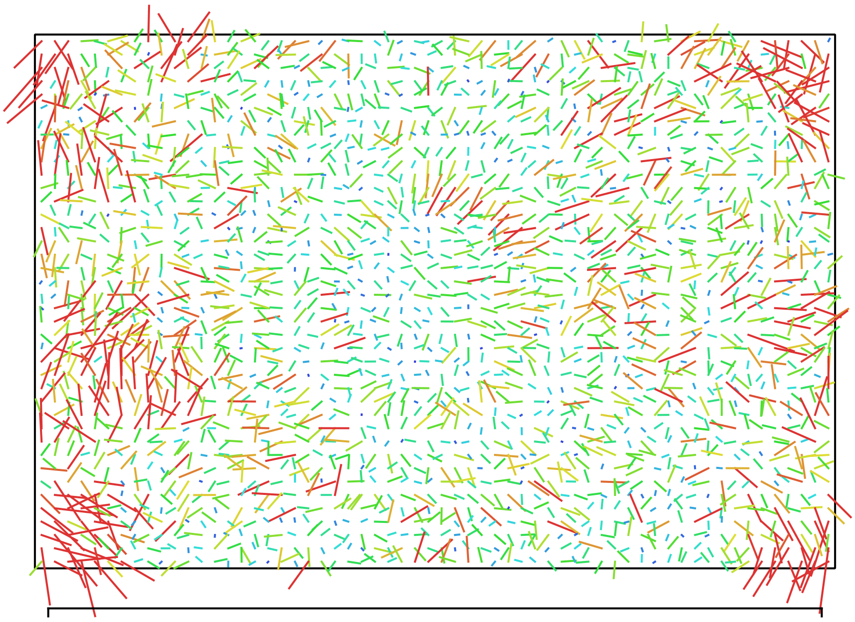

Fig. 4. Image residuals for NX500 (20 mm).

NX500 (20 mm)

530 images, additional corrections

Type	Resolution	Focal Length	Pixel Size
**Frame**	**6480 x 4320**	**20 mm**	**3.7 x 3.7 µm**

F:	5630.67		
Cx:	78.5467	B1:	0.388787
Cy:	35.4919	B2:	0.197826
K1:	-0.020993	P1:	0.00208064
K2:	0.101885	P2:	0.000933032
K3:	-0.235343	P3:	0
K4:	0.189756	P4:	0

Camera Calibration

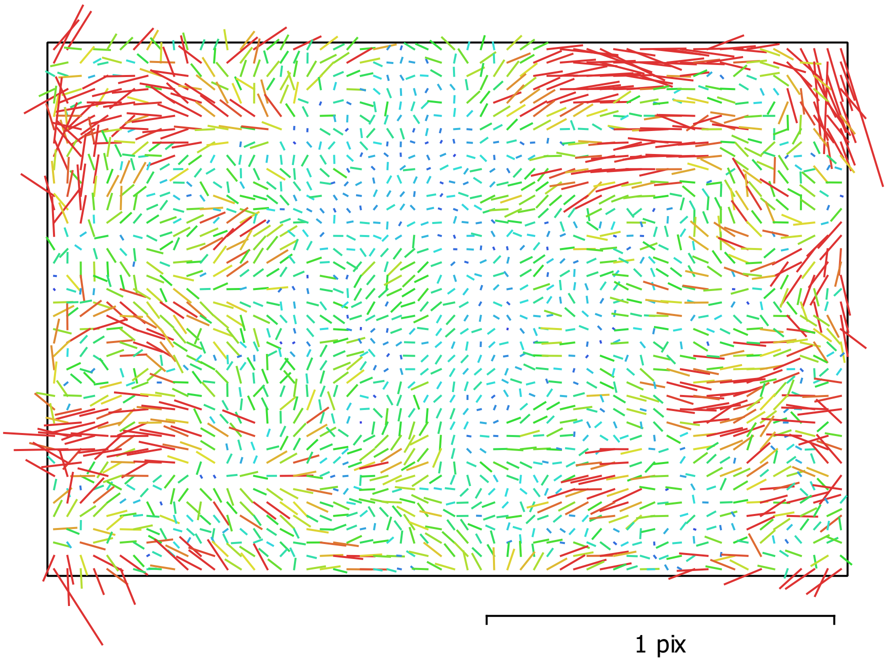

Fig. 5. Image residuals for NX500 (20 mm).

NX500 (20 mm)

513 images, additional corrections

Type	Resolution	Focal Length	Pixel Size
**Frame**	**6480 x 4320**	**20 mm**	**3.7 x 3.7 μm**

F:	5622.62		
Cx:	88.0042	B1:	-1.73328
Cy:	80.693	B2:	-0.295781
K1:	-0.0315993	P1:	0.00226744
K2:	0.133095	P2:	0.00257811
K3:	-0.307425	P3:	0
K4:	0.300568	P4:	0

Camera Calibration

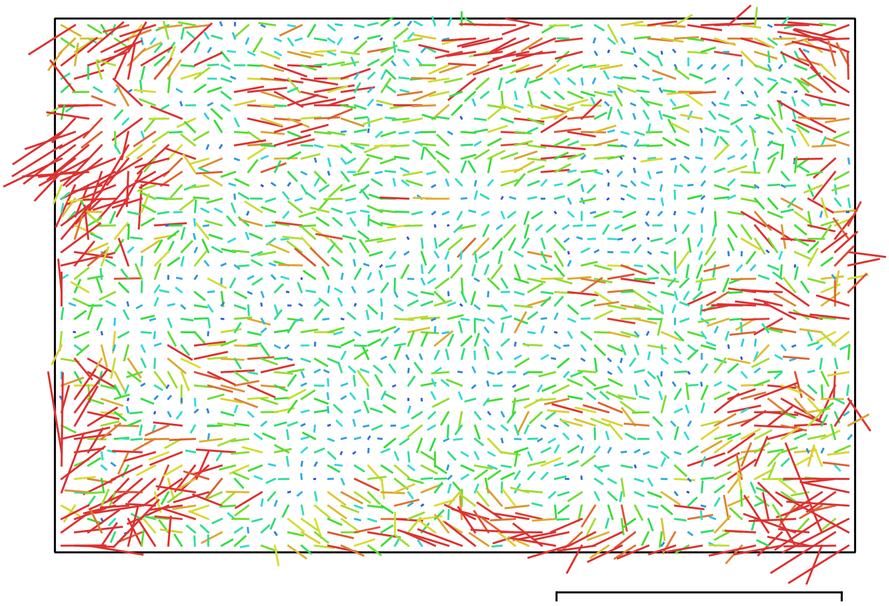

Fig. 6. Image residuals for NX500 (20 mm).

NX500 (20 mm)

412 images, additional corrections

Type	Resolution	Focal Length	Pixel Size
**Frame**	**6480 x 4320**	**20 mm**	**3.7 x 3.7 μm**

F:	5620.03		
Cx:	100.55	B1:	5.16971
Cy:	64.9712	B2:	-0.530711
K1:	-0.0185306	P1:	0.00361211
K2:	0.1461	P2:	0.00208608
K3:	-0.473198	P3:	0
K4:	0.469885	P4:	0

Camera Calibration

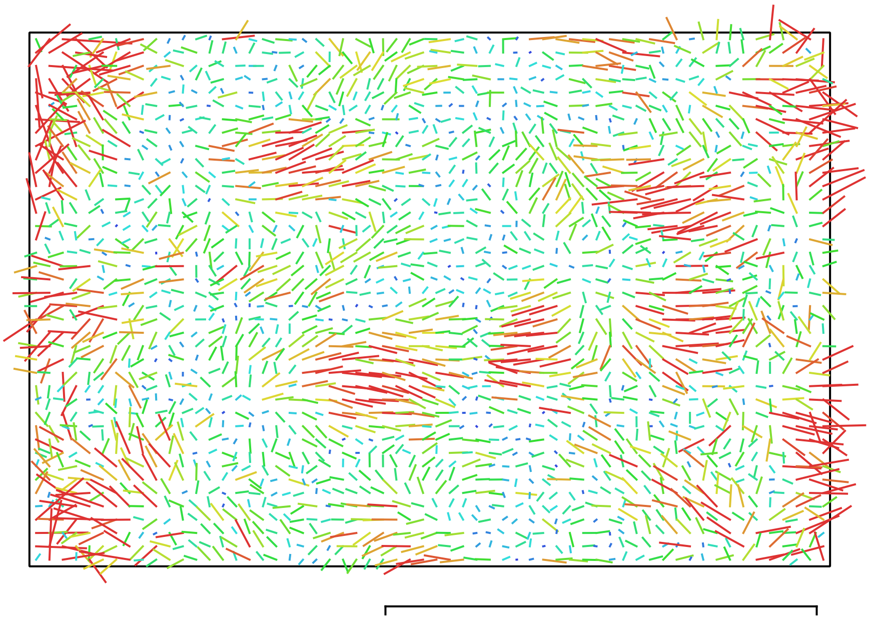

Fig. 7. Image residuals for NX500 (20 mm).

NX500 (20 mm)

478 images, additional corrections

Type	Resolution	Focal Length	Pixel Size
**Frame**	**6480 x 4320**	**20 mm**	**3.7 x 3.7 μm**

F:	5625.18		
Cx:	84.0673	B1:	4.50349
Cy:	28.5202	B2:	-0.865028
K1:	0.01532	P1:	0.00343183
K2:	-0.202905	P2:	-0.000459059
K3:	0.736521	P3:	0
K4:	-0.854775	P4:	0

Ground Control Points

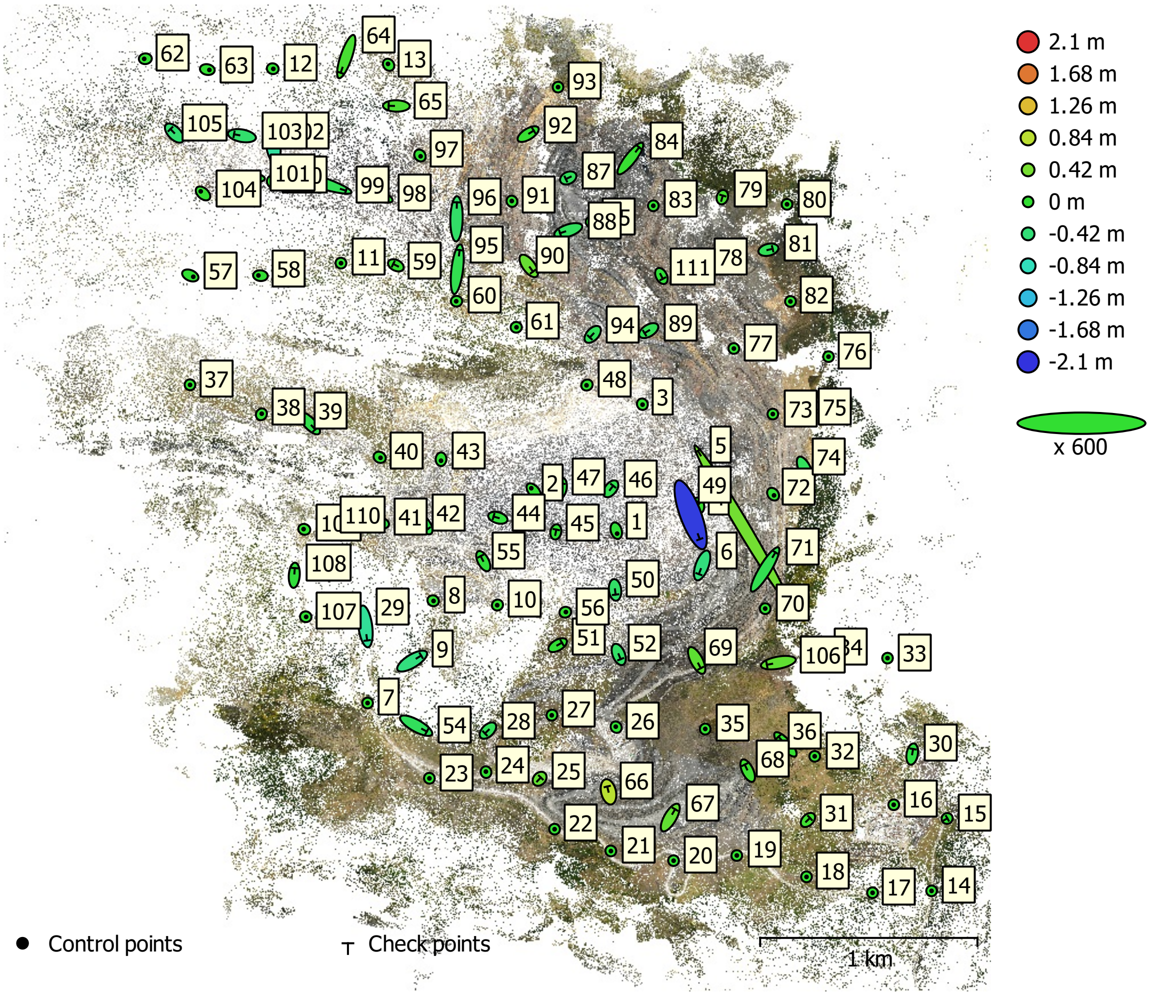

Fig. 8. GCP locations and error estimates.
Z error is represented by ellipse color. X,Y errors are represented by ellipse shape.
Estimated GCP locations are marked with a dot or crossing.

Count	X error (cm)	Y error (cm)	Z error (cm)	XY error (cm)	Total (cm)
55	1.23594	1.25816	0.987998	1.76367	2.02155

Table 2. Control points RMSE.
X - Easting, Y - Northing, Z - Altitude.

Count	X error (cm)	Y error (cm)	Z error (cm)	XY error (cm)	Total (cm)
54	13.8742	19.2805	38.6316	23.7535	45.3501

Table 3. Check points RMSE.
X - Easting, Y - Northing, Z - Altitude.

Label	X error (cm)	Y error (cm)	Z error (cm)	Total (cm)	Image (pix)
1	1.35399	-4.46887	-4.10738	6.21889	0.286 (104)
2	-3.82388	4.55858	0.105795	5.95096	0.291 (109)
3	0.31164	-0.637754	0.178692	0.731971	0.036 (51)
4	-0.796697	3.86478	4.76737	6.18862	0.323 (50)
7	0.0301806	-0.00147246	-0.0169986	0.0346697	0.006 (24)
8	0.635413	-0.325397	-0.421604	0.829086	0.179 (32)
10	-0.38064	-0.257257	1.57832	1.64383	0.219 (42)
11	0.137578	-0.119979	0.388479	0.42923	0.117 (36)
12	0.861371	-0.236377	-0.226753	0.921548	0.231 (26)
13	-0.93711	1.3307	0.298567	1.65472	0.137 (20)
14	-0.0455665	-0.0350278	0.00825939	0.0580644	0.006 (23)
16	-0.0501424	0.00523662	-0.00669117	0.0508572	0.012 (34)
17	0.0737039	-0.00456184	-0.00701505	0.0741774	0.006 (23)
18	0.0374796	-0.102635	0.00169689	0.109277	0.012 (25)
19	0.105675	0.0703964	-0.026613	0.129735	0.015 (20)
20	0.0634233	-0.0486471	-0.0540609	0.0964969	0.017 (16)
21	0.157497	-0.164375	0.0764281	0.240137	0.021 (15)
22	0.0916838	-0.0587196	0.0562256	0.122537	0.018 (13)
23	0.109992	-0.163925	-0.00848086	0.19759	0.014 (18)
24	-0.168966	0.800488	-0.108154	0.825244	0.079 (27)
26	-0.593179	0.758455	-0.213183	0.986186	0.035 (33)
27	-0.265211	-0.252491	0.0176893	0.366608	0.056 (27)
32	-0.0756691	0.0135901	-0.00113081	0.0768881	0.012 (18)
33	0.00114395	-0.000777308	-0.000420656	0.00144561	0.000 (3)
34	-0.00128067	-0.000374863	0.00691042	0.00703808	0.001 (4)
35	0.0394443	0.246115	0.014086	0.249653	0.032 (11)
37	0.0892221	-0.133994	0.465453	0.492505	0.060 (46)
38	0.516558	0.926867	-0.699232	1.27076	0.104 (57)
40	1.29902	-0.773626	-0.4592	1.58013	0.150 (66)
43	-0.0930032	-2.2381	-0.563475	2.30981	0.250 (69)
48	-0.652077	-0.460469	-0.154257	0.813038	0.022 (44)

Label	X error (cm)	Y error (cm)	Z error (cm)	Total (cm)	Image (pix)
56	0.84172	0.250919	0.285059	0.923424	0.212 (50)
57	4.5803	-1.98828	-0.602252	5.02943	0.478 (14)
58	-3.0674	0.31923	-0.383751	3.10775	0.254 (30)
60	-0.51254	-0.048871	-0.175757	0.544037	0.038 (32)
61	0.400626	0.0377847	0.01698	0.402762	0.030 (20)
62	-1.73421	-0.15939	0.25725	1.76042	0.201 (17)
63	3.16666	-0.587504	0.0390209	3.22094	0.374 (16)
70	-0.0173934	0.100993	-0.214907	0.23809	0.018 (30)
72	1.44956	-1.8238	-1.13538	2.59163	0.117 (18)
73	0.023374	0.0210952	0.0256444	0.0406077	0.018 (15)
76	0.0213224	-0.0250301	-0.000746112	0.0328893	0.010 (9)
77	0.0931622	-0.0454364	0.0267953	0.107059	0.017 (11)
78	-0.107307	0.000366689	0.0224732	0.109635	0.053 (11)
80	0.00484885	0.0525883	-0.00944299	0.0536489	0.014 (7)
82	-0.126214	-0.0135828	-0.0195075	0.128433	0.031 (6)
83	0.022757	0.0795084	-0.00676041	0.082977	0.038 (13)
85	-0.0841546	0.0538641	0.0354398	0.106016	0.029 (10)
91	-0.240531	0.457128	0.119195	0.530121	0.036 (20)
93	0.0145105	0.0211552	-0.0457535	0.0524545	0.020 (16)
97	0.938325	-1.16771	-0.210537	1.51272	0.156 (40)
100	0.280608	-1.1168	-1.59383	1.96628	0.338 (28)
104	-3.53551	3.16381	2.13117	5.2011	0.569 (21)
107	0.601469	-0.120227	-0.0842038	0.61912	0.040 (21)
109	-1.06477	0.390828	0.760475	1.36558	0.111 (22)
**Total**	**1.23594**	**1.25816**	**0.987998**	**2.02155**	**0.191**

Table 4. Control points.

X - Easting, Y - Northing, Z - Altitude.

Label	X error (cm)	Y error (cm)	Z error (cm)	Total (cm)	Image (pix)
5	-66.7507	112.151	39.4037	136.331	0.180 (38)
6	-4.98669	-13.3385	-45.603	47.7746	0.083 (66)
9	13.8703	8.42516	-46.2241	48.9901	0.090 (29)

Label	X error (cm)	Y error (cm)	Z error (cm)	Total (cm)	Image (pix)
15	0.330698	0.186277	-1.4712	1.51937	0.015 (27)
25	1.85719	1.91714	29.1632	29.2851	0.026 (22)
28	-4.52704	-4.3781	-23.0536	23.8984	0.033 (29)
29	2.81641	-21.5201	-61.8143	65.5138	0.006 (18)
30	1.37319	7.77622	-11.5765	14.0132	0.007 (20)
31	3.16947	3.02955	8.71689	9.75745	0.013 (26)
36	-10.5006	11.7328	2.86149	16.0034	0.025 (14)
39	8.58329	-9.35406	-14.3021	19.1238	0.111 (40)
41	5.28457	-1.16389	-12.3376	13.4721	0.175 (56)
42	4.92581	-7.09523	-15.6406	17.8672	0.176 (48)
44	-6.51555	1.89444	-6.74235	9.5656	0.315 (73)
45	0.706827	3.54688	-1.40485	3.87989	0.288 (94)
46	3.63559	4.66456	-18.8119	19.7196	0.217 (63)
47	1.46147	7.68192	-9.78456	12.5254	0.328 (107)
49	13.9911	-37.53	-203.815	207.713	0.425 (59)
50	0.836968	-7.17882	-32.4893	33.2835	0.196 (56)
51	6.54154	3.79497	-1.12931	7.64649	0.169 (42)
52	2.95853	-7.27609	-31.105	32.0813	0.140 (56)
54	16.346	-9.05157	-25.0538	31.2541	0.016 (31)
55	-4.04987	7.65052	-4.42941	9.72376	0.223 (51)
59	-4.06011	2.00891	2.38632	5.12003	0.106 (9)
64	-8.52579	-24.8812	13.2084	29.4317	0.165 (13)
65	-11.8344	0.223209	14.5193	18.7326	0.181 (31)
66	-1.9549	8.1774	81.871	82.3016	0.031 (22)
67	7.40632	12.7251	28.8497	32.3896	0.026 (12)
68	-4.49511	9.21076	7.5159	12.7096	0.028 (16)
69	6.29871	-11.9651	26.1775	29.4636	0.025 (28)
71	15.932	26.1499	-18.1793	35.6109	0.084 (24)
74	5.09521	-10.612	-25.1888	27.8039	0.018 (10)
75	6.35709	2.80877	-16.4711	17.8774	0.006 (6)
79	0.715176	2.4443	11.7292	12.0025	0.021 (7)
81	6.45819	1.56007	-21.5487	22.5497	0.057 (6)

Label	X error (cm)	Y error (cm)	Z error (cm)	Total (cm)	Image (pix)
84	13.5417	17.2122	3.536	22.1843	0.019 (12)
87	-3.65877	-1.73559	-24.6974	25.0271	0.020 (10)
88	-12.5822	-4.22231	-25.857	29.0642	0.024 (13)
89	-6.32041	-3.86981	-21.0118	22.2805	0.020 (27)
90	6.43931	-8.75611	32.3554	34.1322	0.023 (28)
92	8.58878	5.35085	2.84438	10.5114	0.019 (18)
94	-4.44807	-4.72249	-23.9568	24.8197	0.033 (35)
95	3.62279	29.3064	-17.9243	34.5437	0.061 (38)
96	0.558113	25.2937	-31.9723	40.7715	0.028 (23)
98	18.0074	-6.01311	-3.62367	19.3276	0.217 (32)
99	46.8692	-15.0313	-20.486	53.3136	0.261 (30)
101	-22.667	2.2499	-11.1567	25.3639	0.434 (26)
102	-1.90796	14.3845	-65.1168	66.7139	0.345 (21)
103	-12.0156	2.14642	-35.435	37.4783	0.446 (20)
105	-5.99697	6.15548	-37.9893	38.9492	0.305 (24)
106	-17.6225	-3.25775	28.0399	33.2776	0.005 (13)
108	0.787041	10.973	-5.29911	12.2109	0.072 (19)
110	-7.943	11.5253	-23.2813	27.1651	0.083 (28)
111	2.24111	-4.28438	-4.82508	6.8308	0.023 (14)
**Total**	**13.8742**	**19.2805**	**38.6316**	**45.3501**	**0.207**

Table 5. Check points.

X - Easting, Y - Northing, Z - Altitude.

Digital Elevation Model

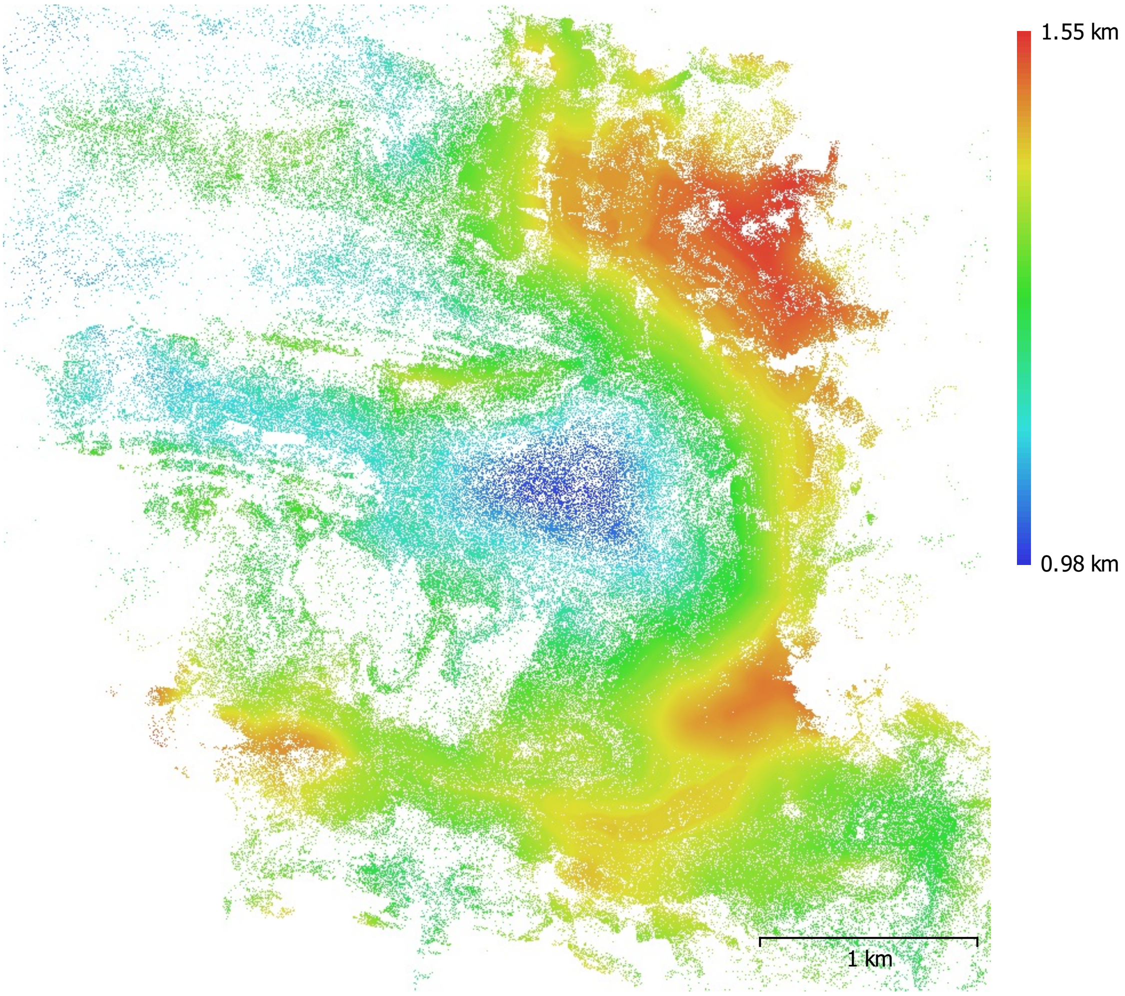

Fig. 9. Reconstructed digital elevation model.

Resolution:	unknown
Point density:	unknown

Processing Parameters

**General**	
Cameras	2595
Aligned cameras	2575
Markers	110
**Shapes**	
Polygon	1
Coordinate system	ETRS89 / UTM zone 30N (EPSG::25830)
Rotation angles	Yaw, Pitch, Roll
**Tie Points**	
Points	747,676 of 12,529,745
RMS reprojection error	0.0767386 (0.178878 pix)
Max reprojection error	1.29335 (2.78552 pix)
Mean key point size	2.29923 pix
Point colors	3 bands, uint8
Key points	No
Average tie point multiplicity	3.65511
**Alignment parameters**	
Accuracy	High
Generic preselection	Yes
Reference preselection	No
Key point limit	60,000
Key point limit per Mpx	1,000
Tie point limit	0
Exclude stationary tie points	Yes
Guided image matching	No
Adaptive camera model fitting	No
Matching time	4 hours 7 minutes
Matching memory usage	3.73 GB
Alignment time	2 hours 17 minutes
Alignment memory usage	4.82 GB
**Optimization parameters**	
Parameters	f, b1, b2, cx, cy, k1-k4, p1, p2
Fit additional corrections	Yes
Adaptive camera model fitting	No
Optimization time	14 minutes 6 seconds
Date created	2023:11:13 15:04:46
Software version	2.0.0.15597
File size	752.12 MB
**System**	
Software name	Agisoft Metashape Professional
Software version	2.0.3 build 16960
OS	Windows 64 bit
RAM	63.90 GB
CPU	Intel(R) Core(TM) i7-7700 CPU @ 3.60GHz
GPU(s)	Quadro M4000
