# Supplementary material for: Automated cleaning of tie point clouds following USGS guidelines in Agisoft Metashape professional (ver. 2.1.0)
Source: MethodsX. 2024 Mar 26;12:102679. doi: 10.1016/j.mex.2024.102679 (PMC10992719; doi:10.1016/j.mex.2024.102679)
Supplement: Supplementary file 3 — The supplementary material includes supplementary text, figures and the processing reports generated by the software. [file mmc3.zip › Lucia_SCC-RMSEm_r3.pdf]

Lucia_SCC-RMSEm_r3

Automatically cleaned sparse cloud using the SCC script (aiming for minimizing the unweighted RMS reprojection error). UAS data provided by Sanz-Ablanedo et al. (2018).

Sanz-Ablanedo, E., Chandler, J. H., Rodríguez-Pérez, J. R., and Ordóñez, C.: Accuracy of Unmanned Aerial Vehicle (UAV) and SfM Photogrammetry Survey as a Function of the Number and Location of Ground Control Points Used, Remote Sensing, 10, 1606, 2018.

28 December 2023

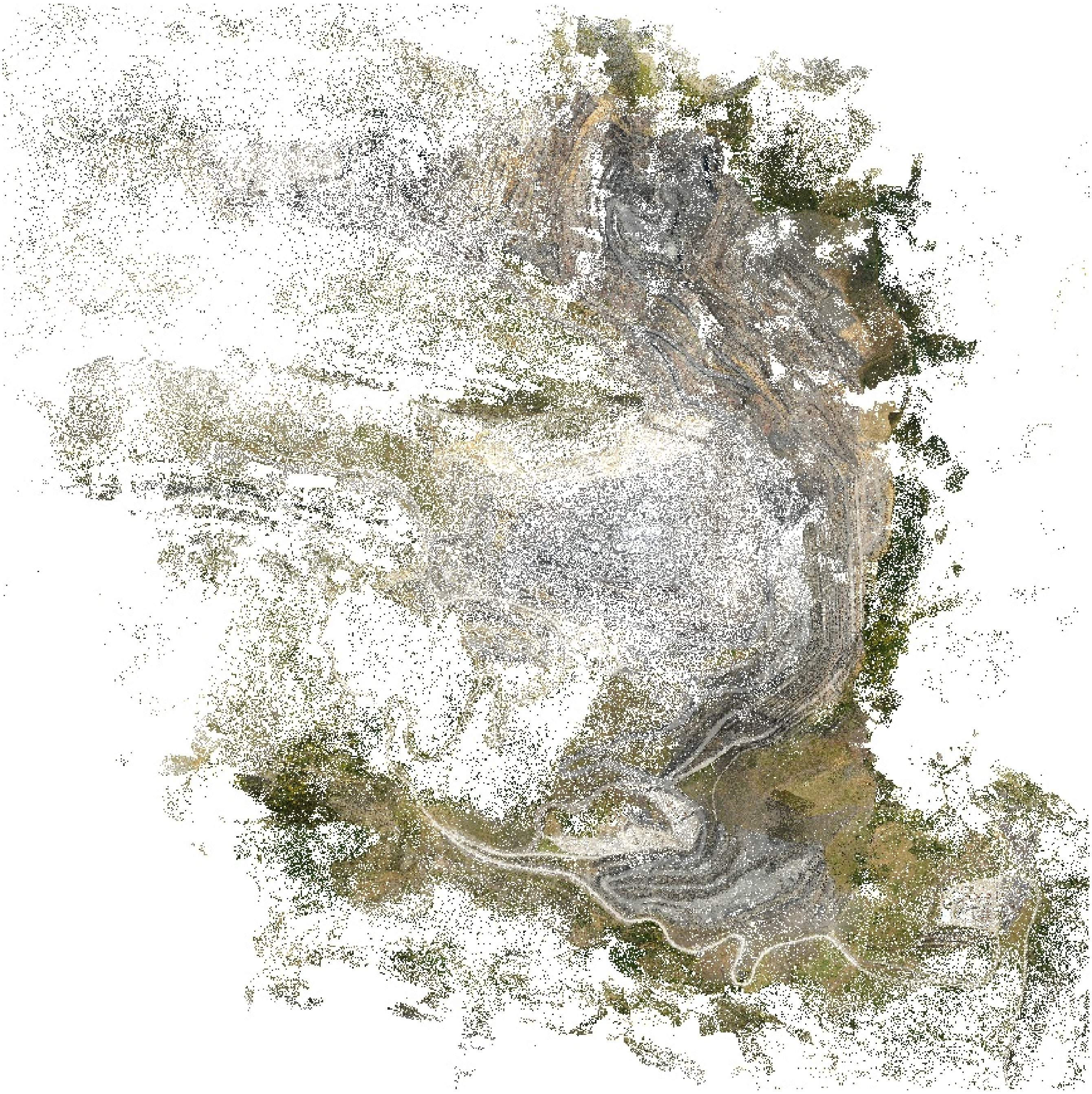

Survey Data

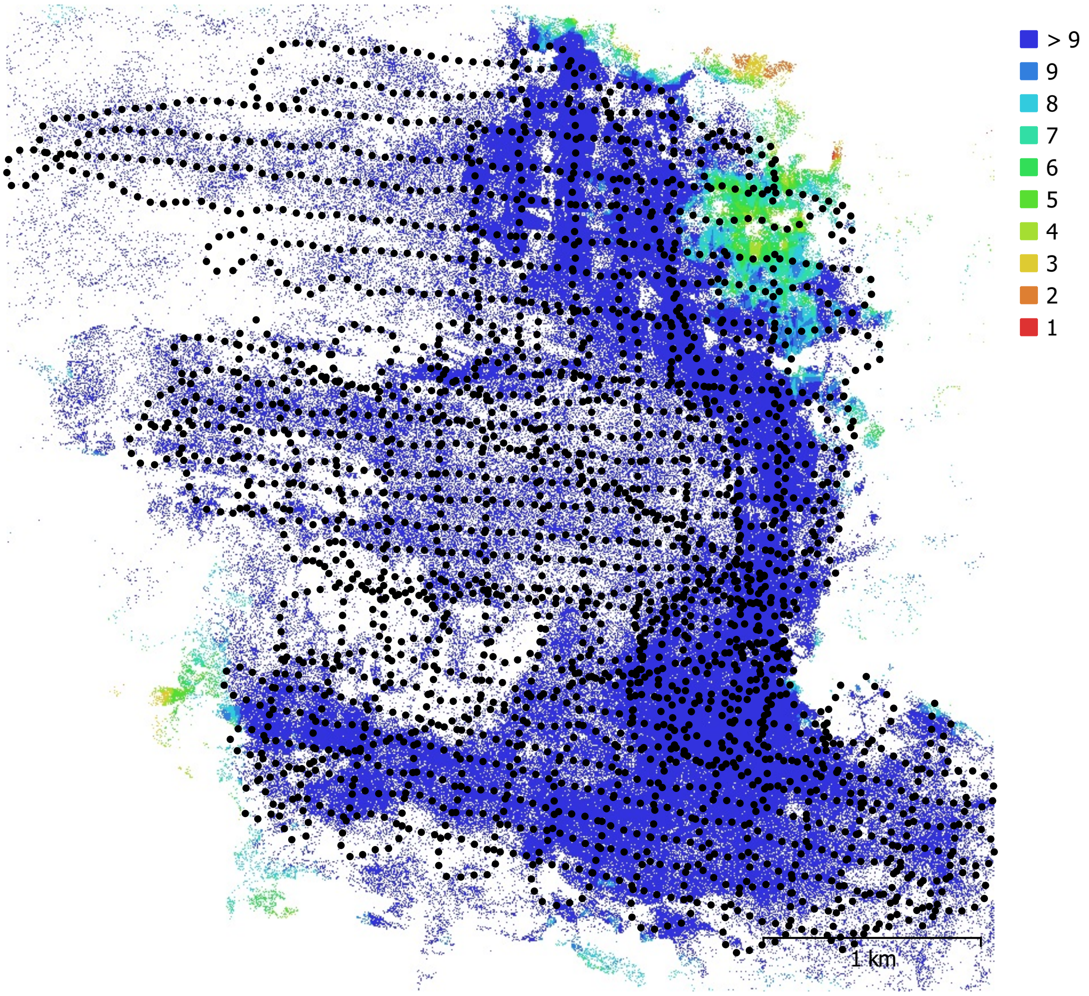

Fig. 1. Camera locations and image overlap.

Number of images:	2,595	Camera stations:	2,575
Flying altitude:	350 m	Tie points:	747,298
Ground resolution:	6.22 cm/pix	Projections:	1,534,884
Coverage area:	6.86 km²	Reprojection error:	0.179 pix

Camera Model	Resolution	Focal Length	Pixel Size	Precalibrated
NX500 (20 mm)	6480 x 4320	20 mm	3.7 x 3.7 μm	No
NX500 (20 mm)	6480 x 4320	20 mm	3.7 x 3.7 μm	No
NX500 (20 mm)	6480 x 4320	20 mm	3.7 x 3.7 μm	No
NX500 (20 mm)	6480 x 4320	20 mm	3.7 x 3.7 μm	No
NX500 (20 mm)	6480 x 4320	20 mm	3.7 x 3.7 μm	No

Camera Model	Resolution	Focal Length	Pixel Size	Precalibrated
NX500 (20 mm)	6480 x 4320	20 mm	3.7 x 3.7 μm	No

Table 1. Cameras.

Camera Calibration

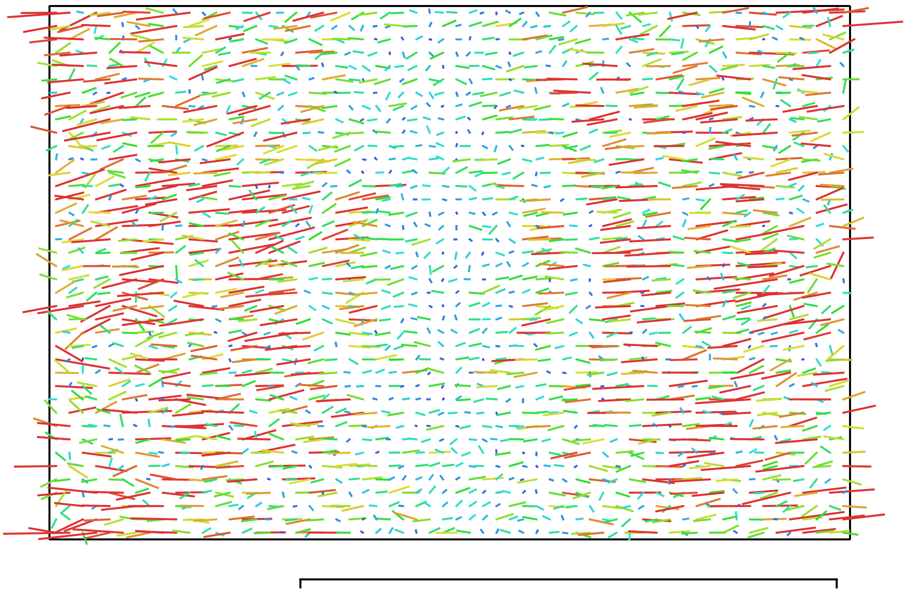

Fig. 2. Image residuals for NX500 (20 mm).

NX500 (20 mm)

200 images, additional corrections

Type	Resolution	Focal Length	Pixel Size
**Frame**	**6480 x 4320**	**20 mm**	**3.7 x 3.7 µm**

F:	5619.96		
Cx:	87.497	B1:	3.22158
Cy:	21.4493	B2:	0.919995
K1:	-0.0147414	P1:	0.00224486
K2:	0.0388659	P2:	-0.000572988
K3:	-0.0368494	P3:	0
K4:	-0.00316268	P4:	0

Camera Calibration

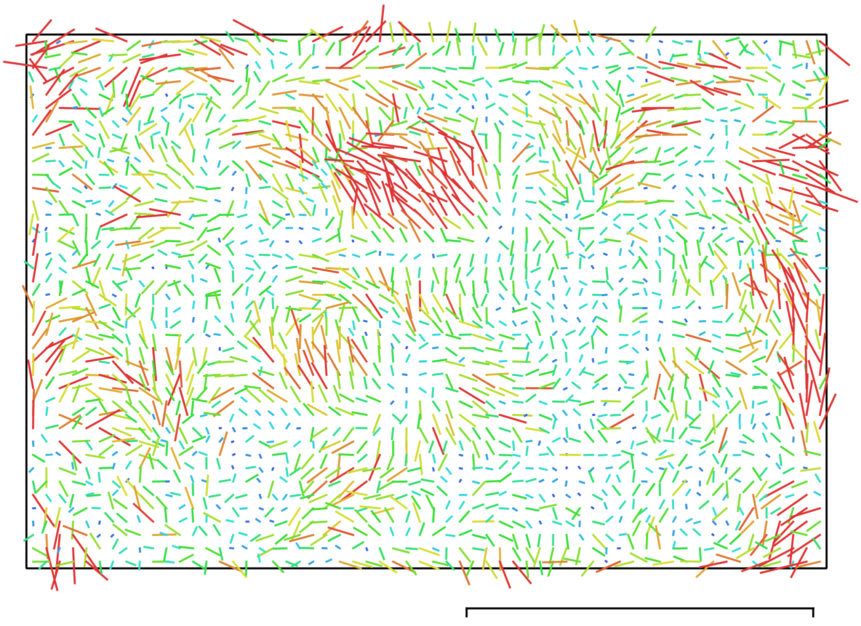

Fig. 3. Image residuals for NX500 (20 mm).

NX500 (20 mm)

462 images, additional corrections

Type	Resolution	Focal Length	Pixel Size
**Frame**	**6480 x 4320**	**20 mm**	**3.7 x 3.7 μm**

F:	5618.21		
Cx:	81.3107	B1:	-1.17448
Cy:	28.4638	B2:	-0.633184
K1:	0.0399982	P1:	0.0028439
K2:	-0.351014	P2:	-0.00071363
K3:	0.961816	P3:	0
K4:	-0.866624	P4:	0

Camera Calibration

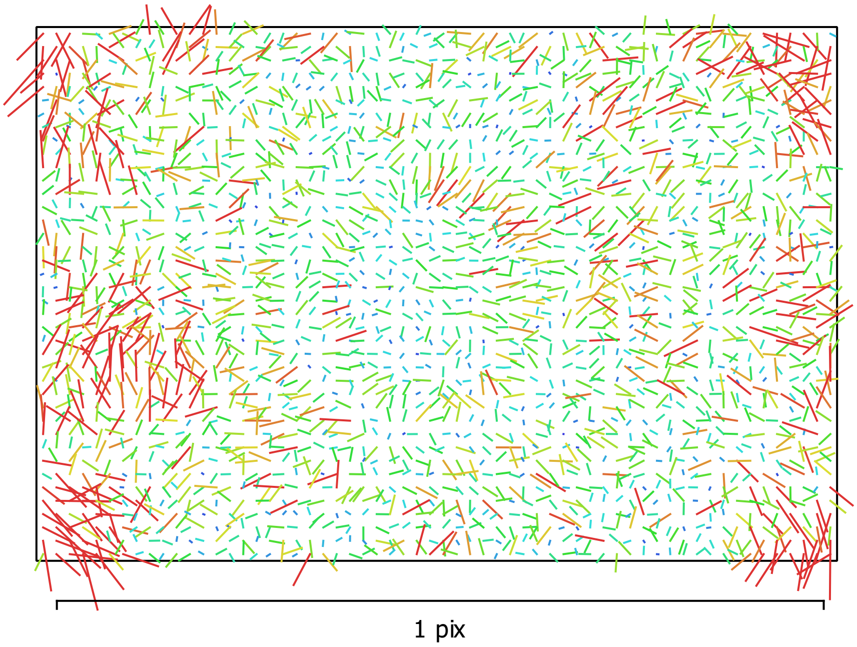

Fig. 4. Image residuals for NX500 (20 mm).

NX500 (20 mm)

530 images, additional corrections

Type	Resolution	Focal Length	Pixel Size
**Frame**	**6480 x 4320**	**20 mm**	**3.7 x 3.7 µm**

F:	5630.69		
Cx:	78.6344	B1:	0.394431
Cy:	35.5277	B2:	0.205339
K1:	-0.0210487	P1:	0.0020878
K2:	0.101978	P2:	0.000932061
K3:	-0.235171	P3:	0
K4:	0.189362	P4:	0

Camera Calibration

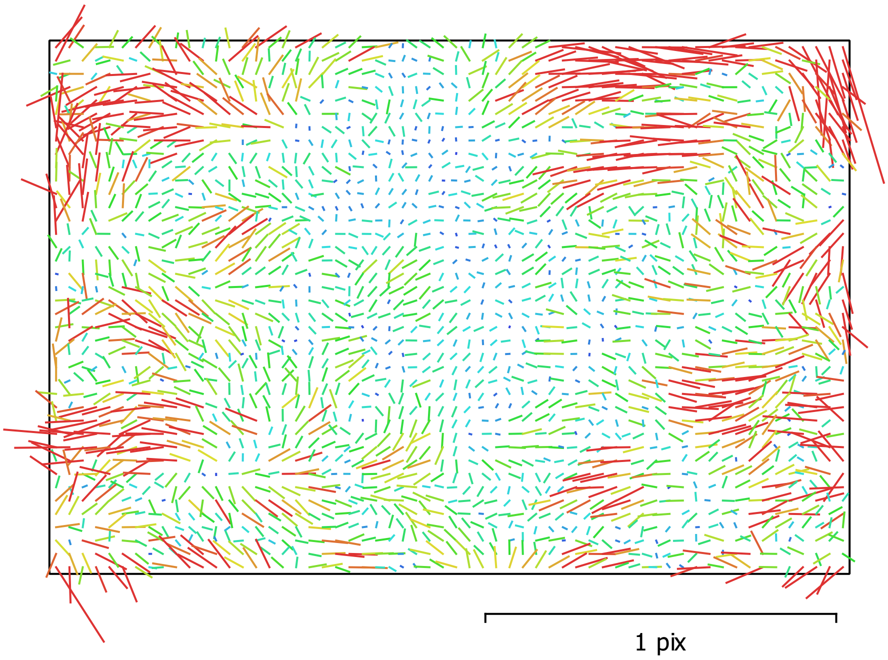

Fig. 5. Image residuals for NX500 (20 mm).

NX500 (20 mm)

513 images, additional corrections

Type	Resolution	Focal Length	Pixel Size
**Frame**	**6480 x 4320**	**20 mm**	**3.7 x 3.7 µm**

F:	5622.84		
Cx:	87.8748	B1:	-1.73804
Cy:	80.6638	B2:	-0.30181
K1:	-0.0320268	P1:	0.00225763
K2:	0.134808	P2:	0.00257519
K3:	-0.310106	P3:	0
K4:	0.301788	P4:	0

Camera Calibration

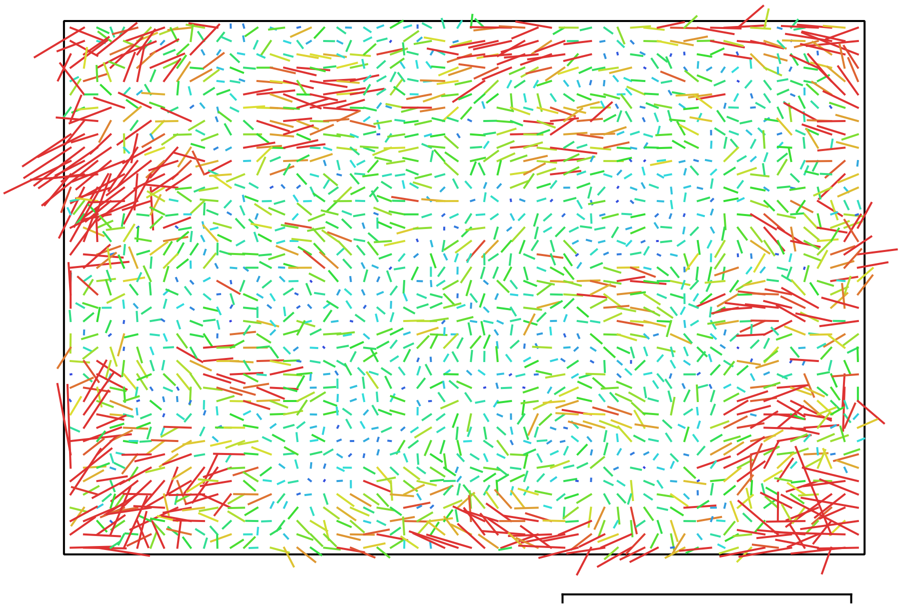

Fig. 6. Image residuals for NX500 (20 mm).

NX500 (20 mm)

412 images, additional corrections

Type	Resolution	Focal Length	Pixel Size
**Frame**	**6480 x 4320**	**20 mm**	**3.7 x 3.7 µm**

F:	5621.02		
Cx:	100.267	B1:	5.05311
Cy:	65.6414	B2:	-0.483861
K1:	-0.020814	P1:	0.00358835
K2:	0.156853	P2:	0.00215268
K3:	-0.49427	P3:	0
K4:	0.484889	P4:	0

Camera Calibration

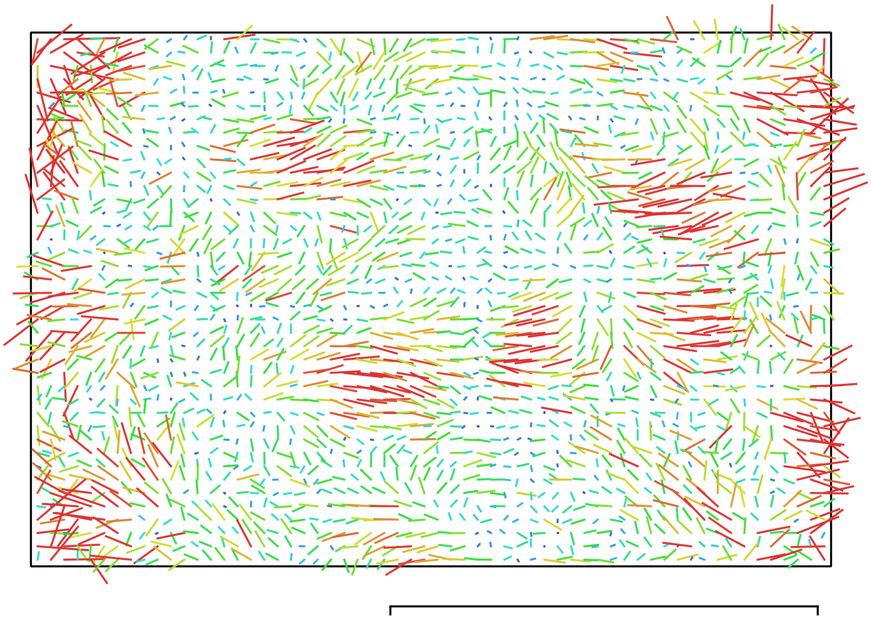

Fig. 7. Image residuals for NX500 (20 mm).

NX500 (20 mm)

478 images, additional corrections

Type	Resolution	Focal Length	Pixel Size
**Frame**	**6480 x 4320**	**20 mm**	**3.7 x 3.7 μm**

F:	5624.87		
Cx:	84.1995	B1:	4.54298
Cy:	28.5038	B2:	-0.857469
K1:	0.0161121	P1:	0.00343937
K2:	-0.207119	P2:	-0.000453632
K3:	0.746004	P3:	0
K4:	-0.862914	P4:	0

Ground Control Points

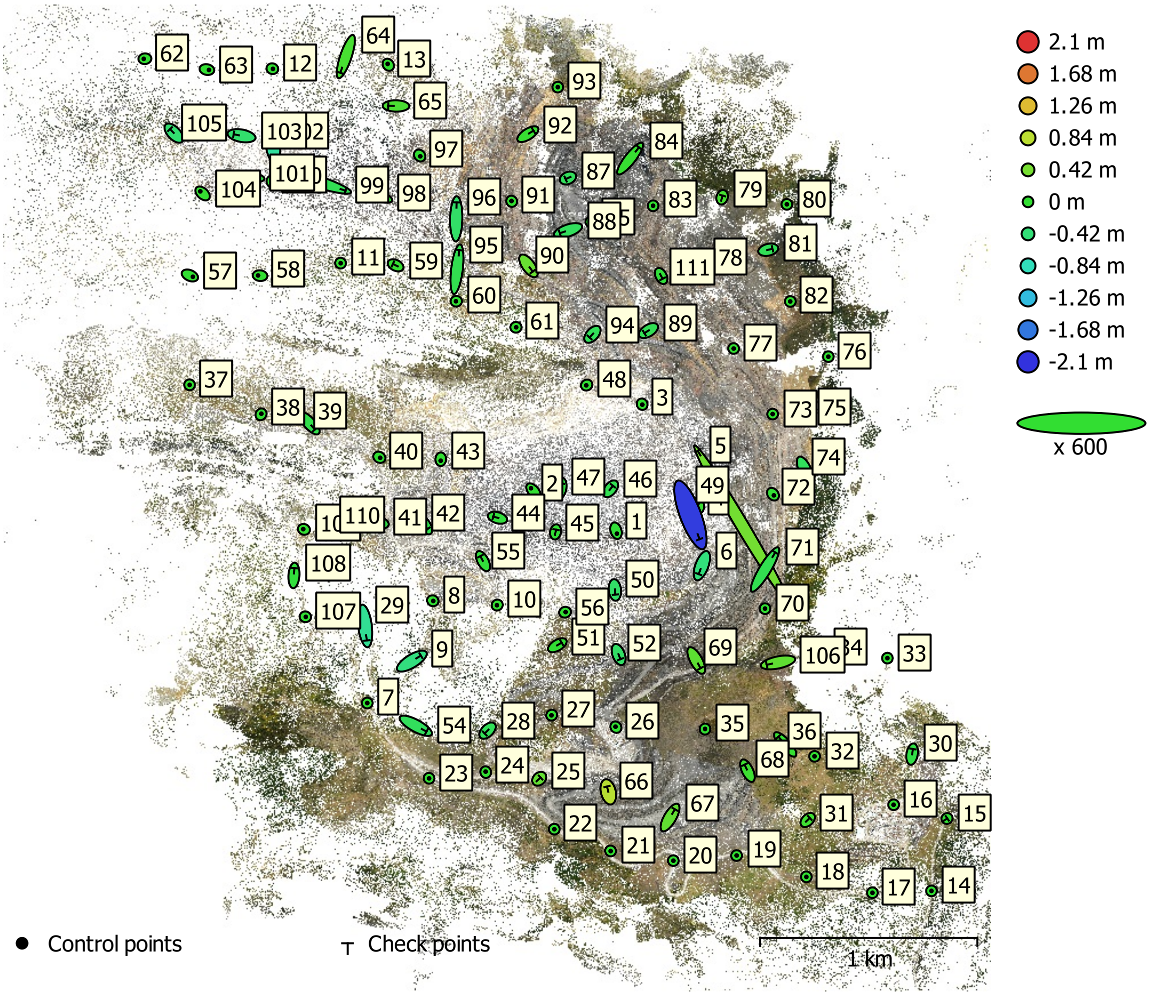

Fig. 8. GCP locations and error estimates.
Z error is represented by ellipse color. X,Y errors are represented by ellipse shape.
Estimated GCP locations are marked with a dot or crossing.

Count	X error (cm)	Y error (cm)	Z error (cm)	XY error (cm)	Total (cm)
55	1.23372	1.25818	0.986464	1.76212	2.01945

Table 2. Control points RMSE.
X - Easting, Y - Northing, Z - Altitude.

Count	X error (cm)	Y error (cm)	Z error (cm)	XY error (cm)	Total (cm)
54	13.8721	19.3014	38.5148	23.7693	45.2589

Table 3. Check points RMSE.
X - Easting, Y - Northing, Z - Altitude.

Label	X error (cm)	Y error (cm)	Z error (cm)	Total (cm)	Image (pix)
1	1.37008	-4.47506	-4.10195	6.22328	0.286 (104)
2	-3.81715	4.56112	0.0985372	5.94846	0.290 (109)
3	0.300139	-0.639865	0.175034	0.728112	0.036 (51)
4	-0.795809	3.86288	4.7606	6.18211	0.323 (50)
7	0.0313696	0.00221449	-0.0172764	0.0358807	0.006 (24)
8	0.636551	-0.329367	-0.426572	0.834052	0.179 (32)
10	-0.379557	-0.25908	1.57653	1.64215	0.219 (42)
11	0.14239	-0.119332	0.386736	0.429045	0.118 (36)
12	0.859655	-0.240704	-0.22476	0.920577	0.230 (26)
13	-0.929838	1.32772	0.298445	1.64818	0.136 (20)
14	-0.0479679	-0.0341792	0.00813006	0.0594579	0.006 (23)
16	-0.0516171	0.00407591	-0.00690114	0.0522357	0.012 (34)
17	0.0747342	-0.00867536	-0.00655745	0.0755213	0.006 (23)
18	0.0416183	-0.105058	0.00111515	0.113007	0.012 (25)
19	0.104615	0.0772197	-0.0265039	0.132702	0.015 (20)
20	0.0614455	-0.0472843	-0.0527543	0.0937783	0.017 (16)
21	0.155613	-0.167652	0.076291	0.241128	0.022 (15)
22	0.0927903	-0.0554938	0.0549516	0.121282	0.018 (13)
23	0.104107	-0.162826	-0.00811239	0.193433	0.014 (18)
24	-0.162696	0.793626	-0.108993	0.81743	0.079 (27)
26	-0.593706	0.76321	-0.218201	0.991256	0.035 (33)
27	-0.272304	-0.263431	0.0227399	0.379556	0.055 (27)
32	-0.0750702	0.0159422	-0.000609517	0.0767467	0.012 (18)
33	0.00114546	-0.000779327	-0.000466864	0.00146198	0.000 (3)
34	-0.000464277	-0.000150358	0.00588958	0.00590977	0.001 (4)
35	0.0476351	0.256625	0.013469	0.261356	0.033 (11)
37	0.086843	-0.130813	0.470793	0.496287	0.060 (46)
38	0.512599	0.924291	-0.701075	1.2683	0.104 (57)
40	1.3035	-0.78962	-0.463811	1.59303	0.150 (66)
43	-0.10113	-2.24118	-0.557559	2.3117	0.250 (69)
48	-0.651373	-0.450176	-0.148647	0.805631	0.021 (44)

Label	X error (cm)	Y error (cm)	Z error (cm)	Total (cm)	Image (pix)
56	0.83581	0.265538	0.292332	0.924417	0.212 (50)
57	4.57192	-1.98519	-0.600703	5.02039	0.477 (14)
58	-3.06826	0.310735	-0.387979	3.10826	0.254 (30)
60	-0.506892	-0.0450223	-0.17504	0.53815	0.037 (32)
61	0.395815	0.0328207	0.0140553	0.397422	0.030 (20)
62	-1.73159	-0.16134	0.256672	1.75793	0.201 (17)
63	3.1567	-0.585641	0.038349	3.21079	0.373 (16)
70	-0.0155936	0.084868	-0.216414	0.232983	0.018 (30)
72	1.44036	-1.81324	-1.13108	2.57717	0.116 (18)
73	0.025984	0.0198883	0.0256396	0.0415705	0.018 (15)
76	0.0210774	-0.0258059	-0.000546738	0.0333242	0.010 (9)
77	0.0899021	-0.0430712	0.0273012	0.103358	0.017 (11)
78	-0.103067	0.0032722	0.0211868	0.105273	0.052 (11)
80	0.00385729	0.055347	-0.00927547	0.0562512	0.014 (7)
82	-0.12195	-0.0171457	-0.0195605	0.124693	0.031 (6)
83	0.0242341	0.0756562	-0.00641651	0.0797015	0.039 (13)
85	-0.0962563	0.0558946	0.0342594	0.116461	0.029 (10)
91	-0.23297	0.459105	0.120786	0.528811	0.037 (20)
93	0.0196436	0.0240626	-0.0449538	0.0546418	0.020 (16)
97	0.931954	-1.17287	-0.209399	1.51262	0.157 (40)
100	0.28263	-1.10449	-1.58698	1.95404	0.337 (28)
104	-3.52605	3.15937	2.12408	5.18906	0.568 (21)
107	0.598507	-0.113545	-0.0832833	0.614849	0.039 (21)
109	-1.06177	0.39397	0.760482	1.36415	0.112 (22)
**Total**	**1.23372**	**1.25818**	**0.986464**	**2.01945**	**0.191**

Table 4. Control points.

X - Easting, Y - Northing, Z - Altitude.

Label	X error (cm)	Y error (cm)	Z error (cm)	Total (cm)	Image (pix)
5	-66.8316	112.179	39.3004	136.364	0.179 (38)
6	-4.8897	-12.9593	-44.9028	46.9906	0.084 (66)
9	13.8043	8.44391	-46.1102	48.8672	0.091 (29)

Label	X error (cm)	Y error (cm)	Z error (cm)	Total (cm)	Image (pix)
15	0.363551	0.247109	-1.39096	1.45877	0.015 (27)
25	1.95956	1.80962	29.1809	29.3025	0.026 (22)
28	-4.41302	-4.34273	-23.163	23.9762	0.033 (29)
29	2.81898	-22.2284	-59.4347	63.5179	0.005 (18)
30	1.31052	7.84814	-11.7135	14.1604	0.007 (20)
31	3.23067	3.03932	8.42374	9.52019	0.013 (26)
36	-10.4886	11.7698	2.59612	15.9775	0.025 (14)
39	8.45919	-9.60975	-14.548	19.3791	0.110 (40)
41	5.3201	-1.19805	-12.3726	13.5211	0.175 (56)
42	4.97105	-7.13431	-15.5549	17.8203	0.177 (48)
44	-6.5229	1.881	-6.74631	9.57074	0.315 (73)
45	0.711068	3.51884	-1.41529	3.85887	0.288 (94)
46	3.66709	4.62415	-18.8297	19.7329	0.217 (63)
47	1.50288	7.68771	-9.83185	12.5708	0.327 (107)
49	14.0248	-37.3743	-203.624	207.5	0.424 (59)
50	0.751418	-7.17291	-32.3814	33.1749	0.196 (56)
51	6.60387	3.91452	-1.09788	7.75499	0.169 (42)
52	2.74389	-7.13796	-31.0761	32.0032	0.140 (56)
54	16.4223	-8.92481	-25.6431	31.7319	0.016 (31)
55	-4.04918	7.61609	-4.4245	9.69417	0.223 (51)
59	-4.13014	1.97173	2.34932	5.14442	0.105 (9)
64	-8.41792	-24.9332	13.4229	29.5415	0.164 (13)
65	-11.7894	0.187998	14.6307	18.7904	0.181 (31)
66	-2.02183	8.1653	81.5959	82.0283	0.031 (22)
67	7.41922	12.6983	28.7647	32.3063	0.026 (12)
68	-4.48918	9.24852	7.29711	12.607	0.027 (16)
69	6.56116	-11.9222	25.7341	29.1107	0.025 (28)
71	15.8001	26.226	-18.1985	35.6178	0.084 (24)
74	4.97476	-10.6031	-25.5117	28.0717	0.018 (10)
75	6.02477	2.93233	-16.9041	18.1837	0.006 (6)
79	0.740999	2.56424	11.1019	11.4183	0.020 (7)
81	6.41714	1.53685	-21.5711	22.5578	0.057 (6)

Label	X error (cm)	Y error (cm)	Z error (cm)	Total (cm)	Image (pix)
84	13.5818	17.3094	2.95636	22.1995	0.019 (12)
87	-3.63407	-1.58962	-25.049	25.3611	0.020 (10)
88	-12.6956	-4.22587	-25.5567	28.8476	0.024 (13)
89	-6.2943	-3.89557	-20.7739	22.0533	0.020 (27)
90	6.44813	-8.85928	32.9683	34.7415	0.022 (28)
92	8.59707	5.42204	2.88259	10.5649	0.019 (18)
94	-4.43168	-4.78166	-23.6059	24.4896	0.033 (35)
95	3.60485	29.3457	-17.9377	34.5822	0.061 (38)
96	0.545499	25.2794	-31.9796	40.7682	0.028 (23)
98	17.9729	-6.01731	-3.56677	19.2861	0.218 (32)
99	46.9113	-15.0525	-20.3391	53.3004	0.261 (30)
101	-22.665	2.28024	-11.1401	25.3575	0.433 (26)
102	-1.94572	14.4404	-65.1717	66.7807	0.344 (21)
103	-12.0284	2.17827	-35.4136	37.464	0.445 (20)
105	-6.00861	6.20617	-37.9782	38.9482	0.305 (24)
106	-17.3417	-3.60651	27.5462	32.7496	0.005 (13)
108	0.736395	11.2246	-5.58169	12.5575	0.072 (19)
110	-7.87636	11.5153	-23.1919	27.0648	0.084 (28)
111	2.3062	-4.32361	-4.59398	6.71691	0.023 (14)
**Total**	**13.8721**	**19.3014**	**38.5148**	**45.2589**	**0.207**

Table 5. Check points.

X - Easting, Y - Northing, Z - Altitude.

Digital Elevation Model

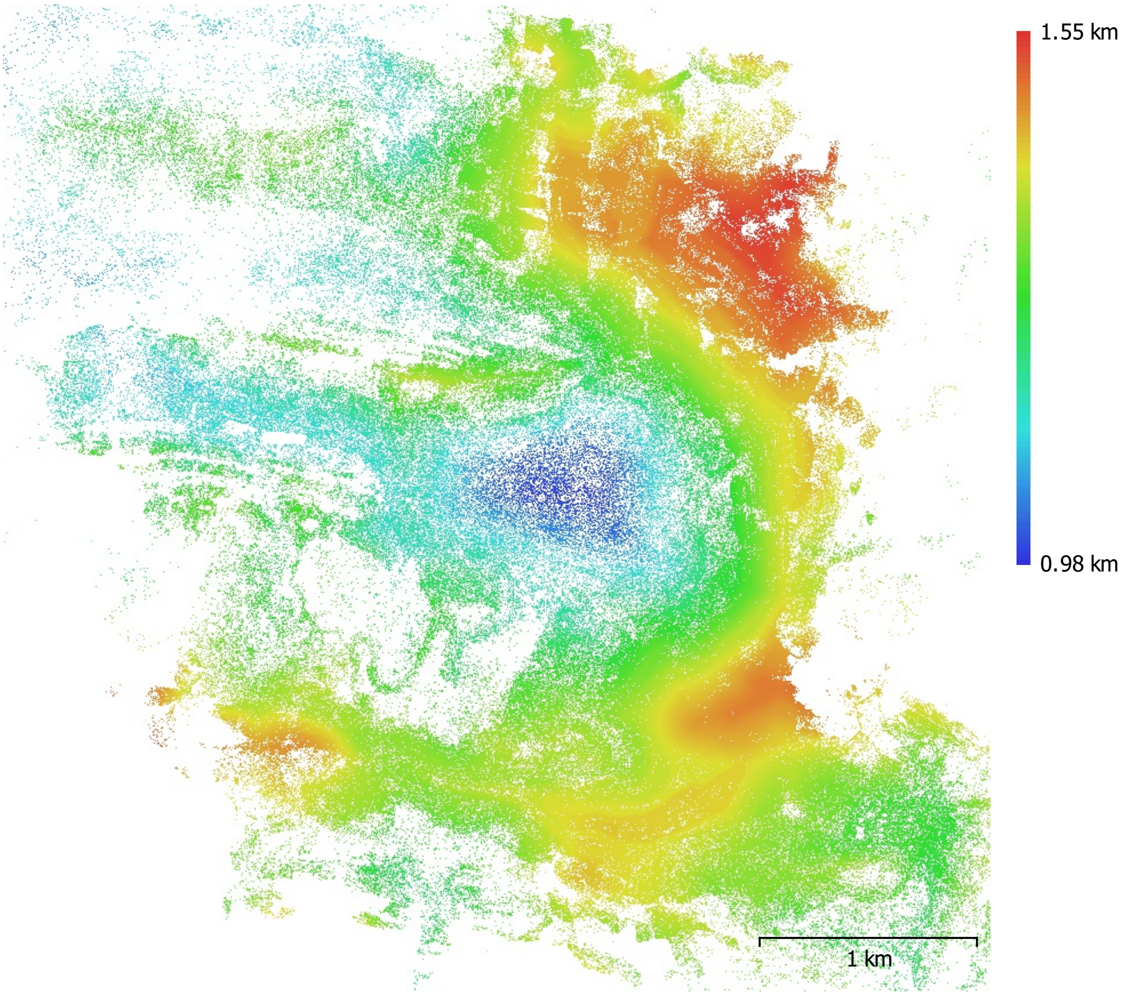

Fig. 9. Reconstructed digital elevation model.

Resolution:	unknown
Point density:	unknown

Processing Parameters

**General**	
Cameras	2595
Aligned cameras	2575
Markers	110
**Shapes**	
Polygon	1
Coordinate system	ETRS89 / UTM zone 30N (EPSG::25830)
Rotation angles	Yaw, Pitch, Roll
**Tie Points**	
Points	747,298 of 12,529,745
RMS reprojection error	0.0767121 (0.178859 pix)
Max reprojection error	0.93961 (1.83016 pix)
Mean key point size	2.29931 pix
Point colors	3 bands, uint8
Key points	No
Average tie point multiplicity	3.65511
**Alignment parameters**	
Accuracy	High
Generic preselection	Yes
Reference preselection	No
Key point limit	60,000
Key point limit per Mpx	1,000
Tie point limit	0
Exclude stationary tie points	Yes
Guided image matching	No
Adaptive camera model fitting	No
Matching time	4 hours 7 minutes
Matching memory usage	3.73 GB
Alignment time	2 hours 17 minutes
Alignment memory usage	4.82 GB
**Optimization parameters**	
Parameters	f, b1, b2, cx, cy, k1-k4, p1, p2
Fit additional corrections	Yes
Adaptive camera model fitting	No
Optimization time	14 minutes 9 seconds
Date created	2023:11:13 15:04:46
Software version	2.0.0.15597
File size	752.11 MB
**System**	
Software name	Agisoft Metashape Professional
Software version	2.0.3 build 16960
OS	Windows 64 bit
RAM	63.90 GB
CPU	Intel(R) Core(TM) i7-7700 CPU @ 3.60GHz
GPU(s)	Quadro M4000
