# Supplementary material for: Automated cleaning of tie point clouds following USGS guidelines in Agisoft Metashape professional (ver. 2.1.0)
Source: MethodsX. 2024 Mar 26;12:102679. doi: 10.1016/j.mex.2024.102679 (PMC10992719; doi:10.1016/j.mex.2024.102679)
Supplement: Supplementary file 3 — The supplementary material includes supplementary text, figures and the processing reports generated by the software. [file mmc3.zip › Lucia_SCC-RMSEm_r4.pdf]

Lucia_SCC-RMSEm_r4

Automatically cleaned sparse cloud using the SCC script (aiming for minimizing the unweighted RMS reprojection error). UAS data provided by Sanz-Ablanedo et al. (2018).

Sanz-Ablanedo, E., Chandler, J. H., Rodríguez-Pérez, J. R., and Ordóñez, C.: Accuracy of Unmanned Aerial Vehicle (UAV) and SfM Photogrammetry Survey as a Function of the Number and Location of Ground Control Points Used, Remote Sensing, 10, 1606, 2018.

28 December 2023

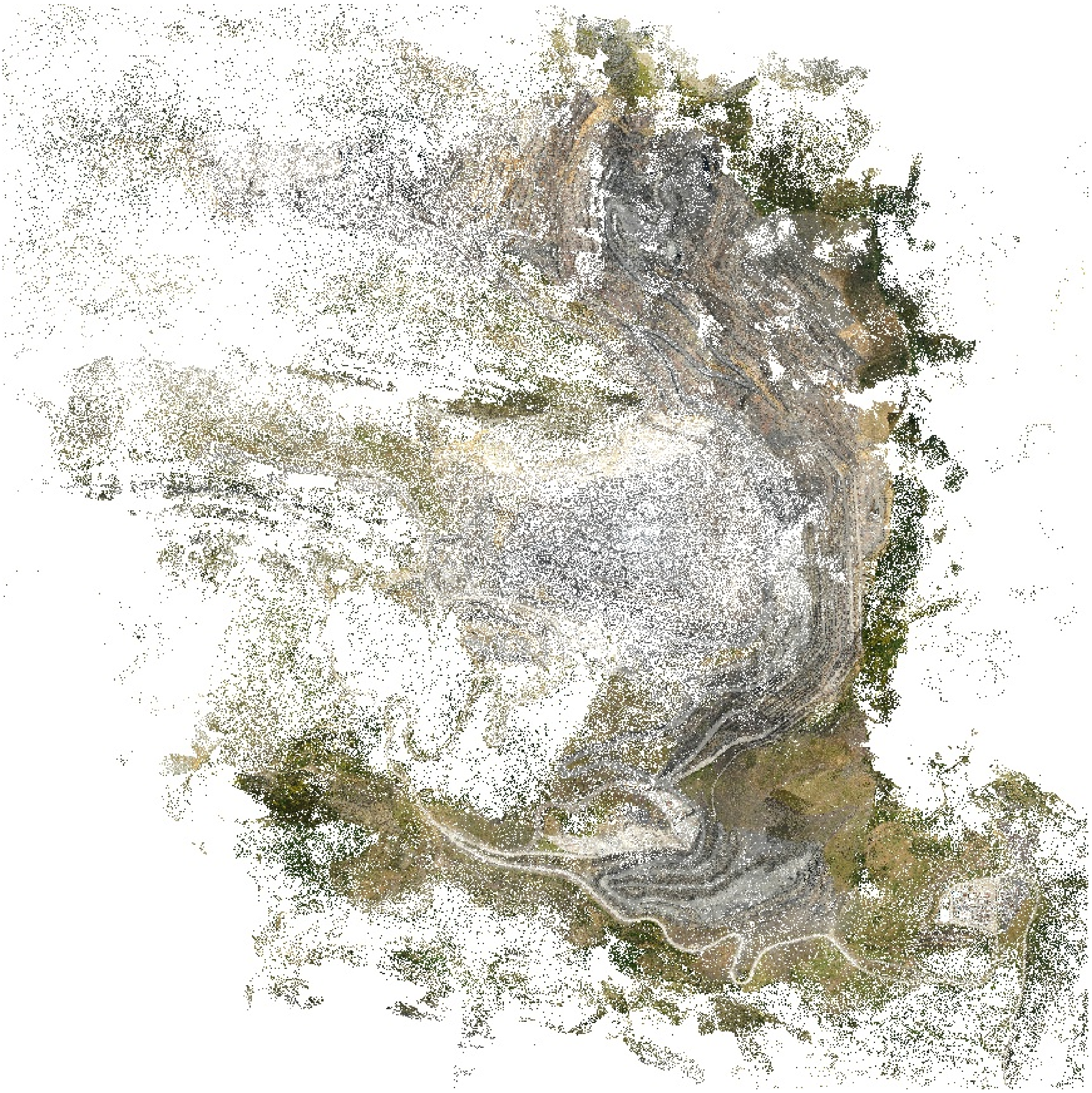

Survey Data

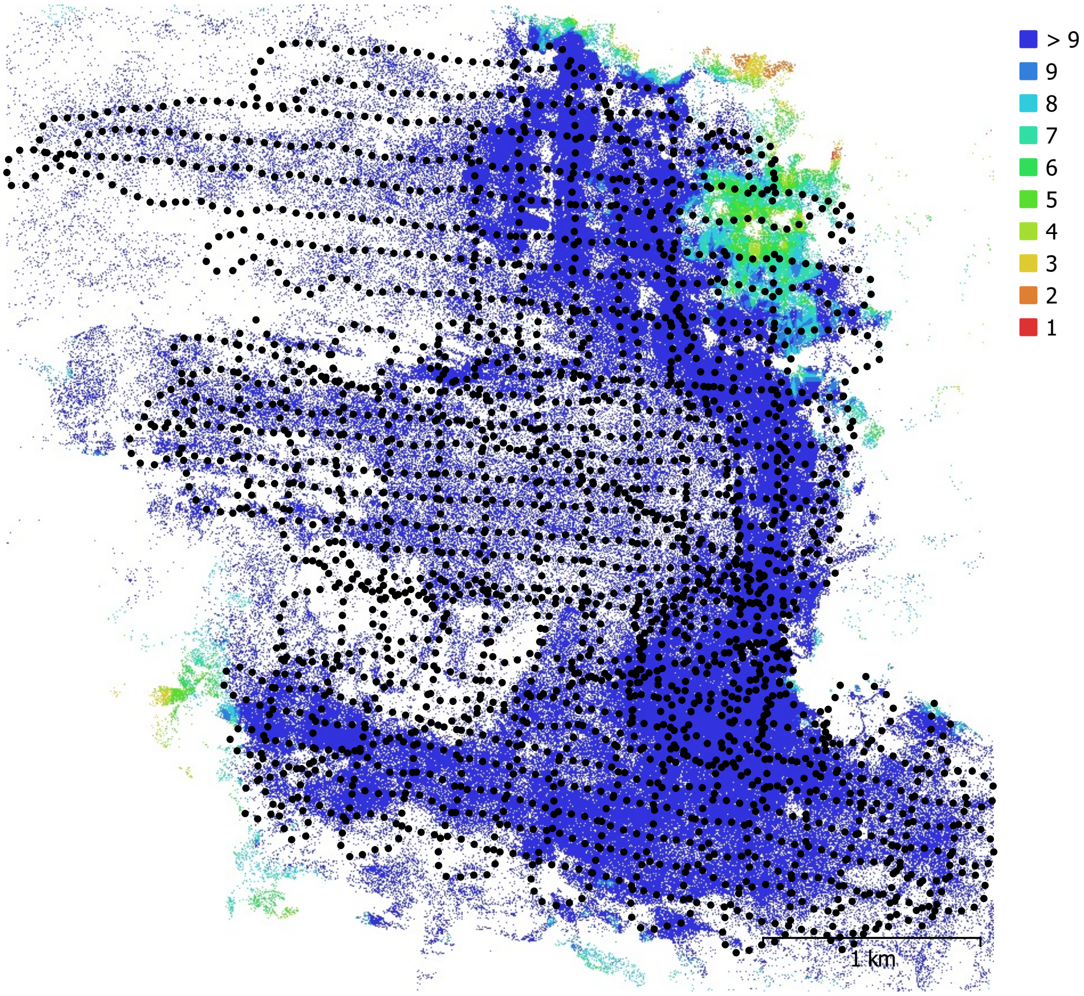

Fig. 1. Camera locations and image overlap.

Number of images:	2,595	Camera stations:	2,575
Flying altitude:	350 m	Tie points:	751,119
Ground resolution:	6.22 cm/pix	Projections:	1,543,197
Coverage area:	6.86 km²	Reprojection error:	0.179 pix

Camera Model	Resolution	Focal Length	Pixel Size	Precalibrated
NX500 (20 mm)	6480 x 4320	20 mm	3.7 x 3.7 μm	No
NX500 (20 mm)	6480 x 4320	20 mm	3.7 x 3.7 μm	No
NX500 (20 mm)	6480 x 4320	20 mm	3.7 x 3.7 μm	No
NX500 (20 mm)	6480 x 4320	20 mm	3.7 x 3.7 μm	No
NX500 (20 mm)	6480 x 4320	20 mm	3.7 x 3.7 μm	No

Camera Model	Resolution	Focal Length	Pixel Size	Precalibrated
NX500 (20 mm)	6480 x 4320	20 mm	3.7 x 3.7 µm	No

Table 1. Cameras.

Camera Calibration

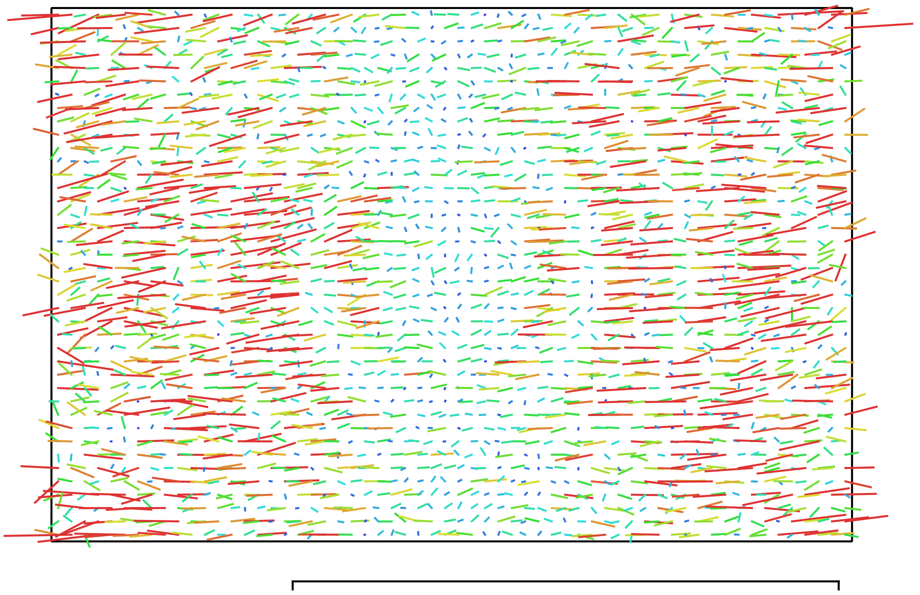

Fig. 2. Image residuals for NX500 (20 mm).

NX500 (20 mm)

200 images, additional corrections

Type	Resolution	Focal Length	Pixel Size
**Frame**	**6480 x 4320**	**20 mm**	**3.7 x 3.7 µm**

F:	5619.62		
Cx:	87.3067	B1:	3.23847
Cy:	21.2259	B2:	0.904228
K1:	-0.0137189	P1:	0.00223462
K2:	0.0340832	P2:	-0.000589679
K3:	-0.0268233	P3:	0
K4:	-0.0112277	P4:	0

Camera Calibration

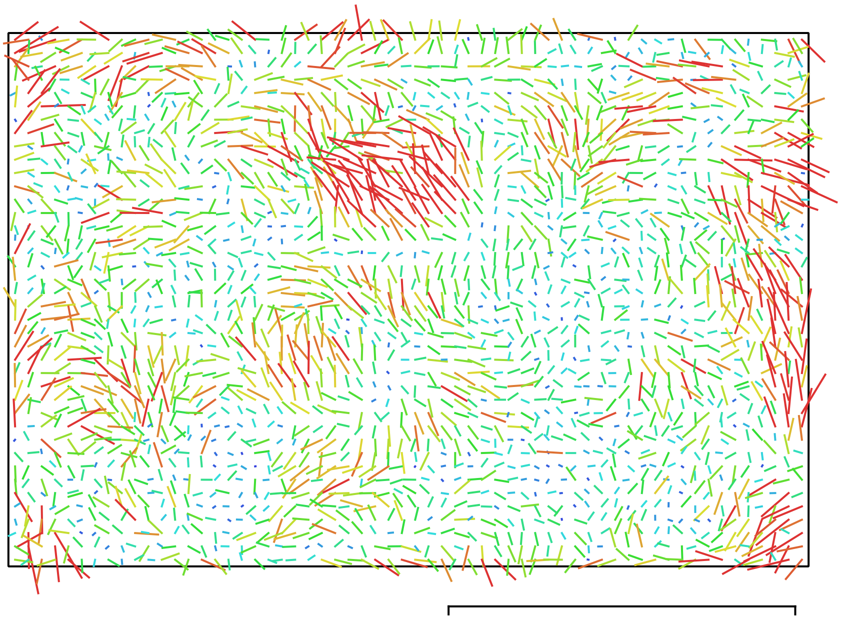

Fig. 3. Image residuals for NX500 (20 mm).

NX500 (20 mm)

462 images, additional corrections

Type	Resolution	Focal Length	Pixel Size
**Frame**	**6480 x 4320**	**20 mm**	**3.7 x 3.7 µm**

F:	5616.7		
Cx:	80.972	B1:	-1.17177
Cy:	28.8002	B2:	-0.654597
K1:	0.0428727	P1:	0.00280749
K2:	-0.36384	P2:	-0.000682328
K3:	0.98805	P3:	0
K4:	-0.88704	P4:	0

Camera Calibration

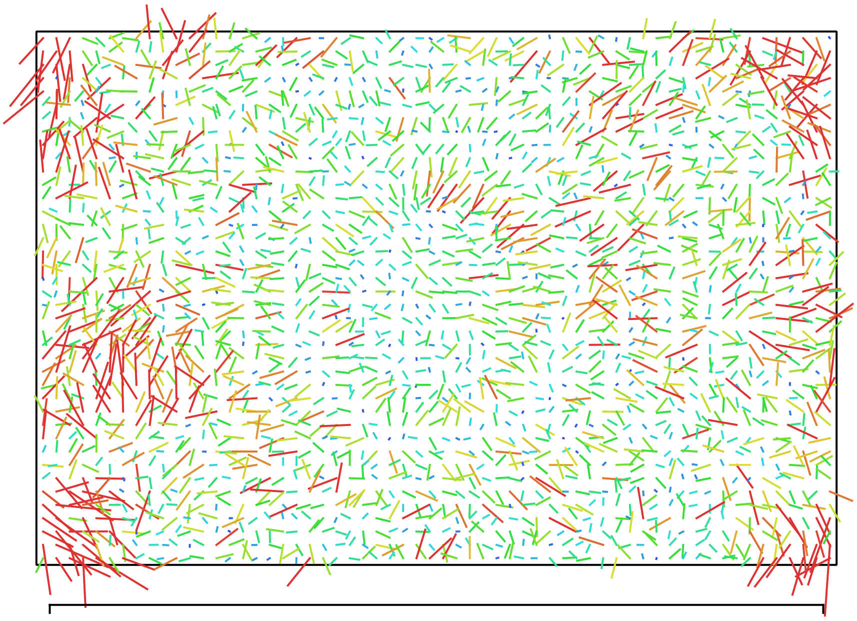

Fig. 4. Image residuals for NX500 (20 mm).

NX500 (20 mm)

530 images, additional corrections

Type	Resolution	Focal Length	Pixel Size
**Frame**	**6480 x 4320**	**20 mm**	**3.7 x 3.7 µm**

F:	5631.61		
Cx:	78.2383	B1:	0.404615
Cy:	35.5391	B2:	0.18709
K1:	-0.0231471	P1:	0.00205077
K2:	0.111533	P2:	0.000949767
K3:	-0.253775	P3:	0
K4:	0.202327	P4:	0

Camera Calibration

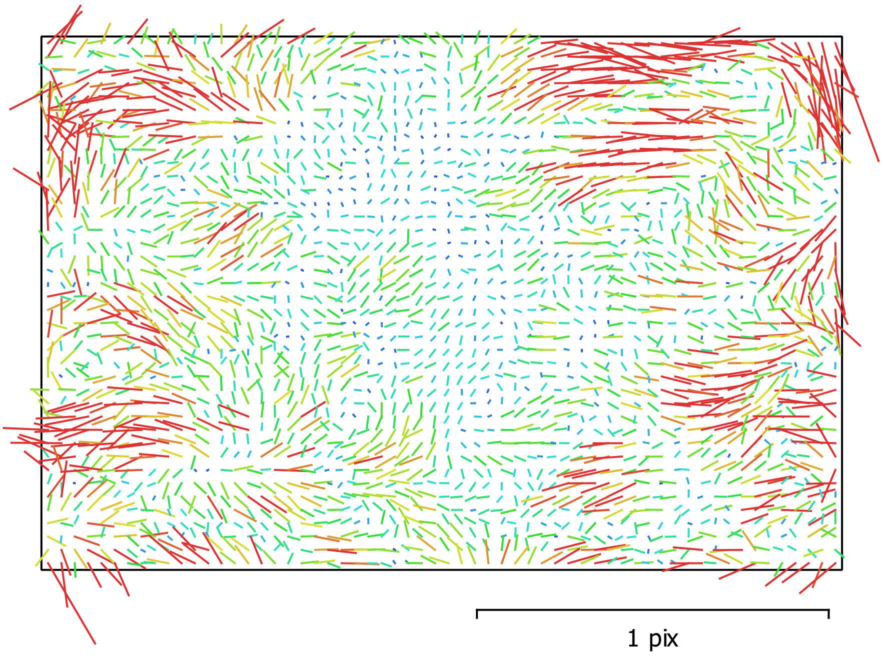

Fig. 5. Image residuals for NX500 (20 mm).

NX500 (20 mm)

513 images, additional corrections

Type	Resolution	Focal Length	Pixel Size
**Frame**	**6480 x 4320**	**20 mm**	**3.7 x 3.7 µm**

F:	5622.49		
Cx:	88.0139	B1:	-1.67963
Cy:	80.8048	B2:	-0.305668
K1:	-0.0310974	P1:	0.00227239
K2:	0.131002	P2:	0.0025944
K3:	-0.304504	P3:	0
K4:	0.299719	P4:	0

Camera Calibration

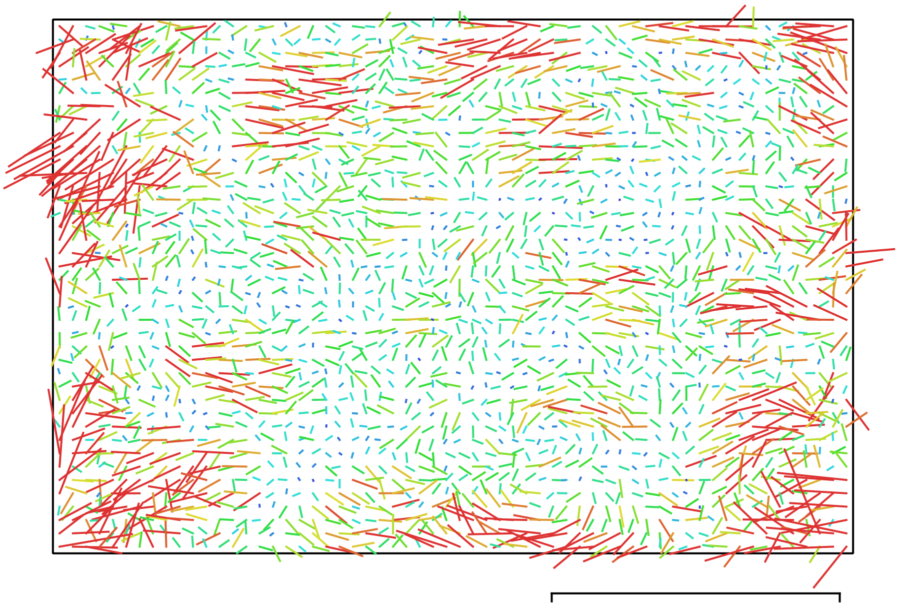

Fig. 6. Image residuals for NX500 (20 mm).

NX500 (20 mm)

412 images, additional corrections

Type	Resolution	Focal Length	Pixel Size
**Frame**	**6480 x 4320**	**20 mm**	**3.7 x 3.7 µm**

F:	5622.41		
Cx:	102.133	B1:	5.165
Cy:	65.8539	B2:	-0.464453
K1:	-0.0238265	P1:	0.00374368
K2:	0.170581	P2:	0.0021911
K3:	-0.521899	P3:	0
K4:	0.506178	P4:	0

Camera Calibration

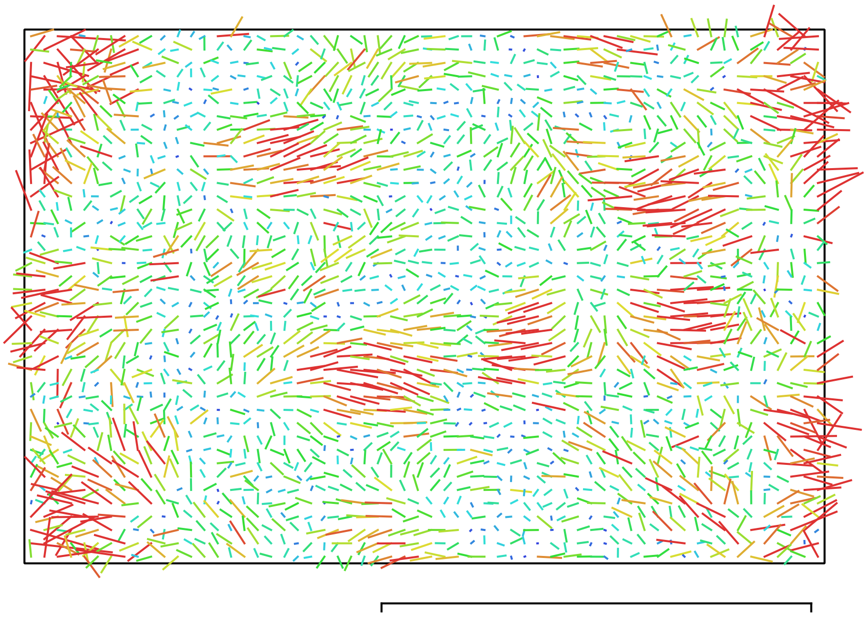

Fig. 7. Image residuals for NX500 (20 mm).

NX500 (20 mm)

478 images, additional corrections

Type	Resolution	Focal Length	Pixel Size
**Frame**	**6480 x 4320**	**20 mm**	**3.7 x 3.7 µm**

F:	5625.41		
Cx:	83.6241	B1:	4.52974
Cy:	28.4023	B2:	-0.849379
K1:	0.0146595	P1:	0.00338099
K2:	-0.197793	P2:	-0.000485925
K3:	0.718812	P3:	0
K4:	-0.833168	P4:	0

Ground Control Points

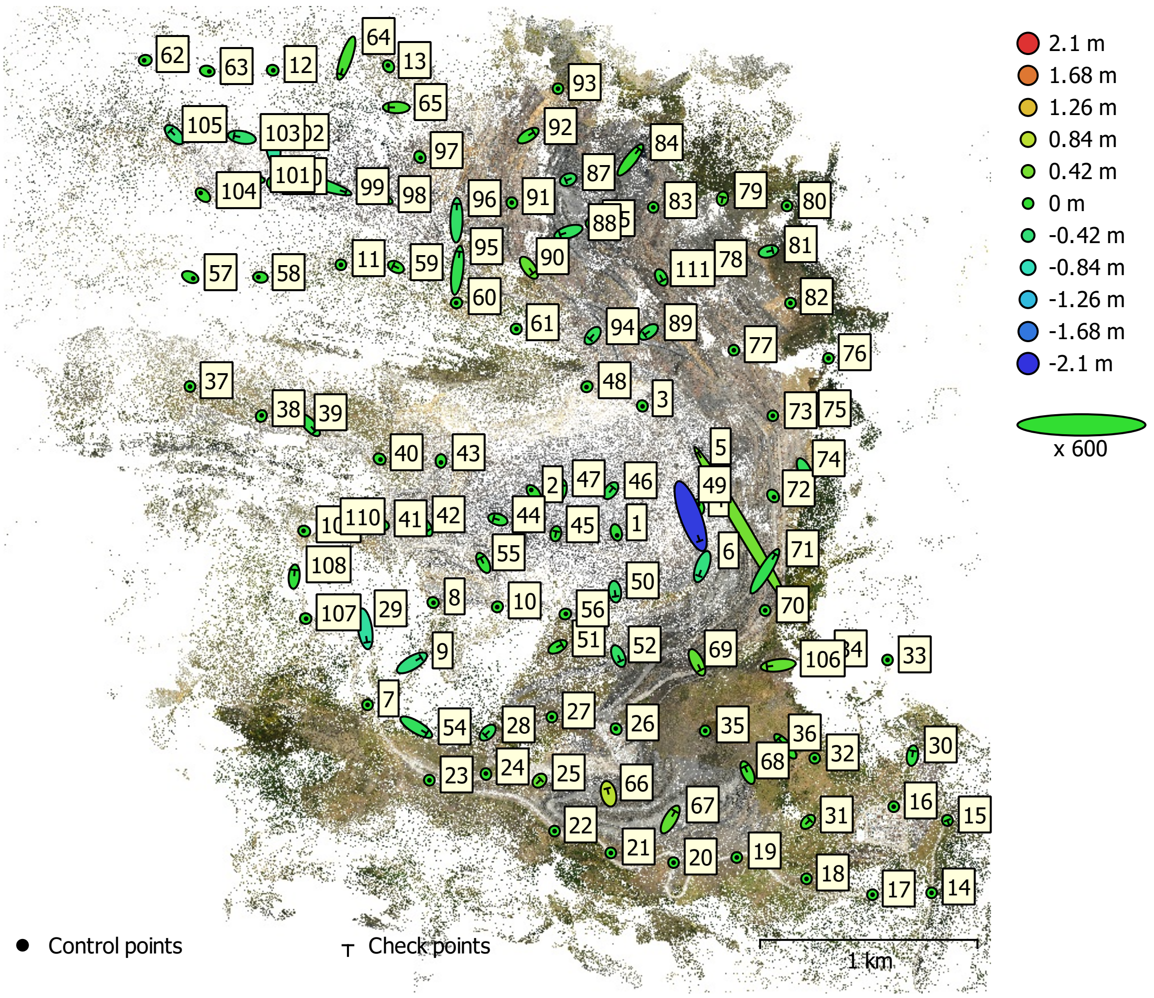

Fig. 8. GCP locations and error estimates.
Z error is represented by ellipse color. X,Y errors are represented by ellipse shape.
Estimated GCP locations are marked with a dot or crossing.

Count	X error (cm)	Y error (cm)	Z error (cm)	XY error (cm)	Total (cm)
55	1.24407	1.26141	0.992775	1.77169	2.03088

Table 2. Control points RMSE.
X - Easting, Y - Northing, Z - Altitude.

Count	X error (cm)	Y error (cm)	Z error (cm)	XY error (cm)	Total (cm)
54	13.8409	19.2693	38.8778	23.725	45.5451

Table 3. Check points RMSE.
X - Easting, Y - Northing, Z - Altitude.

Label	X error (cm)	Y error (cm)	Z error (cm)	Total (cm)	Image (pix)
1	1.33773	-4.45545	-4.11758	6.21249	0.286 (104)
2	-3.83203	4.57024	0.0632621	5.96453	0.291 (109)
3	0.314839	-0.625245	0.192312	0.725975	0.037 (51)
4	-0.790999	3.87558	4.78659	6.20945	0.325 (50)
7	0.0285027	0.00260531	-0.0171077	0.0333447	0.006 (24)
8	0.618545	-0.322577	-0.420092	0.814328	0.180 (32)
10	-0.392517	-0.293286	1.59972	1.67307	0.221 (42)
11	0.143681	-0.121726	0.395134	0.437712	0.119 (36)
12	0.874881	-0.240799	-0.219203	0.933515	0.232 (26)
13	-0.942176	1.3224	0.29799	1.65083	0.137 (20)
14	-0.0463499	-0.0326781	0.00632084	0.0570625	0.006 (23)
16	-0.049164	-0.00154828	-0.00741879	0.0497447	0.011 (34)
17	0.0748446	-0.00512534	-0.00379522	0.0751158	0.006 (23)
18	0.0375114	-0.101404	0.00115128	0.108126	0.012 (25)
19	0.107417	0.0778334	-0.0268357	0.135339	0.015 (20)
20	0.0619718	-0.0524259	-0.0512944	0.0960213	0.017 (16)
21	0.155736	-0.16297	0.0752098	0.237633	0.021 (15)
22	0.0964916	-0.056239	0.0552022	0.124582	0.018 (13)
23	0.113545	-0.169143	-0.00991237	0.203961	0.014 (18)
24	-0.17345	0.812609	-0.106437	0.837704	0.080 (27)
26	-0.601656	0.761645	-0.217315	0.994645	0.034 (33)
27	-0.253654	-0.259215	0.0142407	0.362954	0.056 (27)
32	-0.0673762	0.0203206	-0.000993686	0.0703809	0.012 (18)
33	0.00107491	-0.000532788	-0.000363832	0.00125366	0.000 (3)
34	-0.00173308	0.00219287	0.00372253	0.00465505	0.000 (4)
35	0.0349942	0.240127	0.012945	0.243008	0.033 (11)
37	0.0868429	-0.116707	0.471403	0.493338	0.059 (46)
38	0.518188	0.903075	-0.707319	1.25872	0.103 (57)
40	1.28256	-0.787229	-0.459259	1.5734	0.151 (66)
43	-0.0533883	-2.22263	-0.557005	2.29199	0.251 (69)
48	-0.659605	-0.485014	-0.16776	0.83574	0.023 (44)

Label	X error (cm)	Y error (cm)	Z error (cm)	Total (cm)	Image (pix)
56	0.846201	0.254101	0.278889	0.9265	0.213 (50)
57	4.63506	-2.01575	-0.608867	5.09095	0.484 (14)
58	-3.11523	0.337988	-0.375726	3.15595	0.258 (30)
60	-0.510923	-0.0610091	-0.180429	0.54527	0.038 (32)
61	0.396329	0.0316593	0.0152465	0.397884	0.030 (20)
62	-1.745	-0.168759	0.255312	1.77163	0.201 (17)
63	3.16643	-0.588911	0.0421597	3.221	0.374 (16)
70	-0.0263034	0.109354	-0.207726	0.236221	0.017 (30)
72	1.4616	-1.8324	-1.14328	2.60788	0.118 (18)
73	0.0304337	0.0132747	0.0278664	0.043347	0.018 (15)
76	0.0235154	-0.024593	-0.00155877	0.034062	0.010 (9)
77	0.0924526	-0.0420806	0.0240821	0.104394	0.018 (11)
78	-0.107349	0.0117526	0.0214462	0.1101	0.054 (11)
80	0.00721578	0.0534903	-0.00982379	0.0548615	0.015 (7)
82	-0.128672	-0.0176307	-0.0172369	0.131013	0.032 (6)
83	0.0263864	0.0721428	-0.00360064	0.0769012	0.038 (13)
85	-0.0919159	0.0627438	0.0342508	0.116441	0.028 (10)
91	-0.240357	0.467771	0.122457	0.539979	0.037 (20)
93	0.020001	0.0203984	-0.0459535	0.0541097	0.020 (16)
97	0.954462	-1.16817	-0.208775	1.52289	0.158 (40)
100	0.293394	-1.115	-1.616	1.98513	0.341 (28)
104	-3.56208	3.20519	2.15286	5.25323	0.571 (21)
107	0.605745	-0.121991	-0.083236	0.623488	0.040 (21)
109	-1.06366	0.403563	0.756544	1.36624	0.110 (22)
**Total**	**1.24407**	**1.26141**	**0.992775**	**2.03088**	**0.192**

Table 4. Control points.

X - Easting, Y - Northing, Z - Altitude.

Label	X error (cm)	Y error (cm)	Z error (cm)	Total (cm)	Image (pix)
5	-66.521	112.248	39.6324	136.365	0.183 (38)
6	-5.26186	-14.2348	-47.2468	49.6244	0.084 (66)
9	14.0324	8.37376	-46.3812	49.1756	0.090 (29)

Label	X error (cm)	Y error (cm)	Z error (cm)	Total (cm)	Image (pix)
15	-0.0878383	0.219819	-0.121762	0.266199	0.015 (27)
25	1.9012	1.84872	29.6412	29.7596	0.026 (22)
28	-4.17924	-4.09621	-22.5648	23.3112	0.034 (29)
29	3.26388	-20.6206	-66.6896	69.8811	0.005 (18)
30	1.06788	7.18277	-11.3077	13.4386	0.007 (20)
31	3.12257	2.98913	8.8272	9.82877	0.014 (26)
36	-10.3848	11.8576	2.83365	16.0148	0.025 (14)
39	8.59496	-9.51977	-13.9917	18.9807	0.112 (40)
41	5.2455	-1.1935	-12.3652	13.4847	0.175 (56)
42	4.91864	-7.11218	-15.6017	17.8379	0.176 (48)
44	-6.57373	1.83896	-6.72109	9.5796	0.317 (73)
45	0.697066	3.53987	-1.3904	3.8665	0.289 (94)
46	3.71444	4.77332	-18.7251	19.6776	0.217 (63)
47	1.48909	7.77064	-9.77922	12.5791	0.328 (107)
49	13.9644	-38.123	-204.196	208.193	0.426 (59)
50	0.991496	-7.26254	-32.7494	33.5597	0.197 (56)
51	6.544	3.62359	-1.13002	7.56514	0.170 (42)
52	3.43468	-7.60438	-31.1917	32.2885	0.141 (56)
54	16.1235	-9.65878	-24.815	31.1295	0.016 (31)
55	-4.0919	7.59527	-4.31127	9.64463	0.223 (51)
59	-4.244	2.05121	2.12121	5.169	0.106 (9)
64	-8.52601	-24.8341	13.2739	29.4215	0.166 (13)
65	-11.8639	0.179785	14.337	18.6101	0.181 (31)
66	-2.03298	8.25445	82.5449	82.9815	0.032 (22)
67	7.47731	12.7827	29.1229	32.6718	0.026 (12)
68	-4.33846	9.34231	7.71583	12.8699	0.028 (16)
69	5.76818	-11.7507	27.651	30.593	0.025 (28)
71	16.1694	26.2208	-18.0718	35.7151	0.083 (24)
74	5.17075	-10.7033	-25.0716	27.7468	0.018 (10)
75	5.2685	2.70936	-16.2586	17.3043	0.007 (6)
79	0.465364	2.18825	12.0956	12.3007	0.021 (7)
81	6.29428	1.61213	-21.8642	22.8092	0.058 (6)

Label	X error (cm)	Y error (cm)	Z error (cm)	Total (cm)	Image (pix)
84	13.4924	17.0054	3.55555	21.9971	0.020 (12)
87	-3.70069	-1.52488	-24.3177	24.6449	0.020 (10)
88	-12.8225	-4.16832	-26.2531	29.513	0.024 (13)
89	-6.12379	-4.30869	-20.9752	22.2716	0.021 (27)
90	6.20817	-8.51492	30.2869	32.0678	0.023 (28)
92	8.5648	5.31016	2.77062	10.4513	0.020 (18)
94	-4.43227	-5.19494	-24.2218	25.166	0.034 (35)
95	3.3754	28.6419	-17.4872	33.7277	0.062 (38)
96	0.547925	24.9278	-31.3271	40.0386	0.029 (23)
98	18.0387	-5.94204	-3.64463	19.3387	0.220 (32)
99	46.8056	-14.914	-20.3687	53.1797	0.262 (30)
101	-22.6568	2.24845	-11.1267	25.3415	0.436 (26)
102	-1.85431	14.3888	-64.867	66.4696	0.346 (21)
103	-12.0191	2.20399	-35.3012	37.3562	0.446 (20)
105	-6.13618	6.16988	-38.0014	38.9849	0.305 (24)
106	-17.5073	-2.04365	29.4441	34.3167	0.005 (13)
108	0.898148	10.2668	-4.50281	11.2468	0.071 (19)
110	-7.982	11.2858	-23.1642	26.9753	0.083 (28)
111	2.38736	-4.53243	-5.69617	7.66086	0.023 (14)
**Total**	**13.8409**	**19.2693**	**38.8778**	**45.5451**	**0.208**

Table 5. Check points.

X - Easting, Y - Northing, Z - Altitude.

Digital Elevation Model

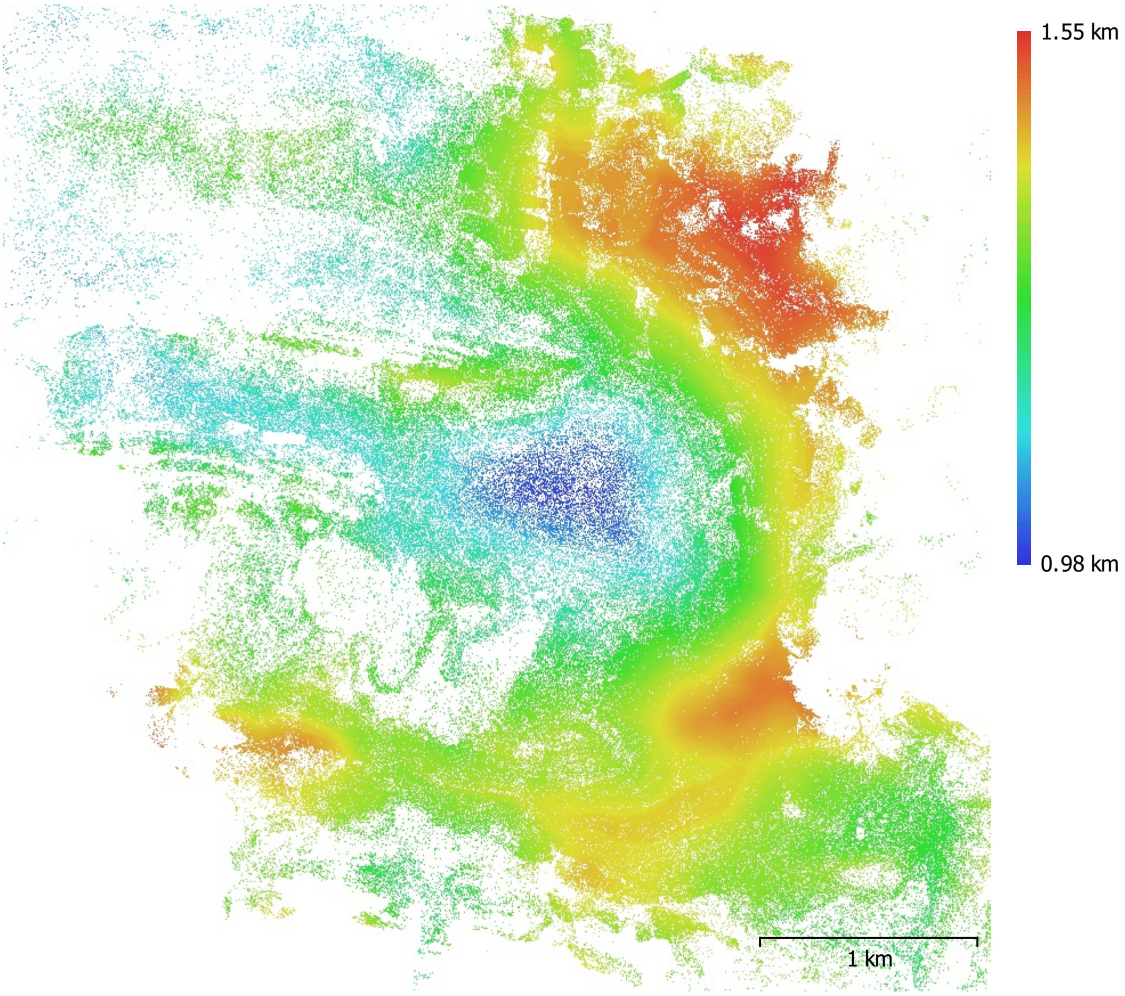

Fig. 9. Reconstructed digital elevation model.

Resolution:	unknown
Point density:	unknown

Processing Parameters

**General**	
Cameras	2595
Aligned cameras	2575
Markers	110
**Shapes**	
Polygon	1
Coordinate system	ETRS89 / UTM zone 30N (EPSG::25830)
Rotation angles	Yaw, Pitch, Roll
**Tie Points**	
Points	751,119 of 12,529,745
RMS reprojection error	0.0769328 (0.179328 pix)
Max reprojection error	1.24116 (2.67311 pix)
Mean key point size	2.29923 pix
Point colors	3 bands, uint8
Key points	No
Average tie point multiplicity	3.65511
**Alignment parameters**	
Accuracy	High
Generic preselection	Yes
Reference preselection	No
Key point limit	60,000
Key point limit per Mpx	1,000
Tie point limit	0
Exclude stationary tie points	Yes
Guided image matching	No
Adaptive camera model fitting	No
Matching time	4 hours 7 minutes
Matching memory usage	3.73 GB
Alignment time	2 hours 17 minutes
Alignment memory usage	4.82 GB
**Optimization parameters**	
Parameters	f, b1, b2, cx, cy, k1-k4, p1, p2
Fit additional corrections	Yes
Adaptive camera model fitting	No
Optimization time	6 minutes 24 seconds
Date created	2023:11:13 15:04:46
Software version	2.0.0.15597
File size	752.20 MB
**System**	
Software name	Agisoft Metashape Professional
Software version	2.0.3 build 16960
OS	Windows 64 bit
RAM	63.90 GB
CPU	Intel(R) Core(TM) i7-7700 CPU @ 3.60GHz
GPU(s)	Quadro M4000
