# Supplementary material for: Automated cleaning of tie point clouds following USGS guidelines in Agisoft Metashape professional (ver. 2.1.0)
Source: MethodsX. 2024 Mar 26;12:102679. doi: 10.1016/j.mex.2024.102679 (PMC10992719; doi:10.1016/j.mex.2024.102679)
Supplement: Supplementary file 3 — The supplementary material includes supplementary text, figures and the processing reports generated by the software. [file mmc3.zip › Lucia_SCC-RMSEm_r5.pdf]

Lucia_SCC-RMSEm_r5

Automatically cleaned sparse cloud using the SCC script (aiming for minimizing the unweighted RMS reprojection error). UAS data provided by Sanz-Ablanedo et al. (2018).

Sanz-Ablanedo, E., Chandler, J. H., Rodríguez-Pérez, J. R., and Ordóñez, C.: Accuracy of Unmanned Aerial Vehicle (UAV) and SfM Photogrammetry Survey as a Function of the Number and Location of Ground Control Points Used, Remote Sensing, 10, 1606, 2018.

28 December 2023

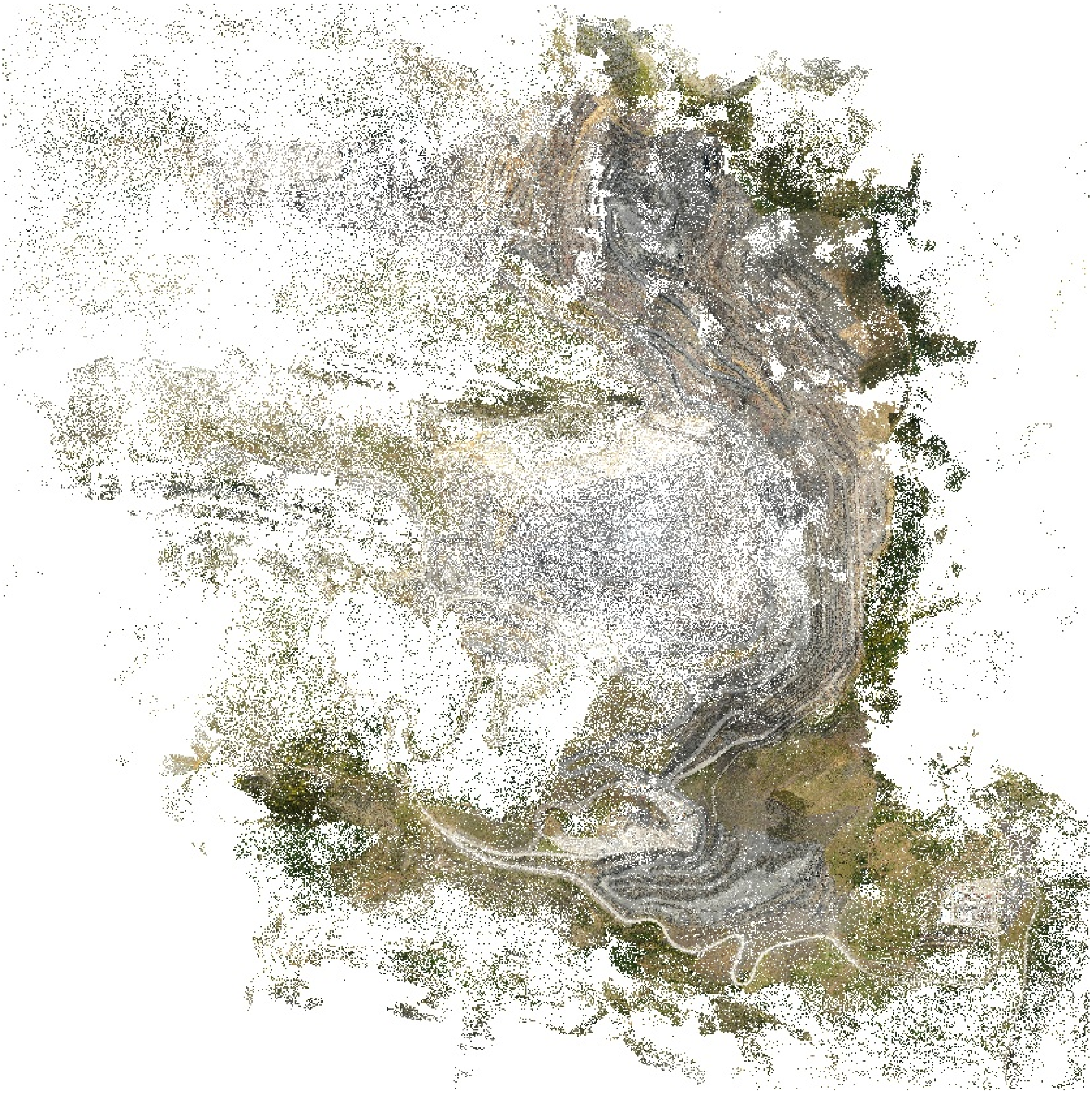

Survey Data

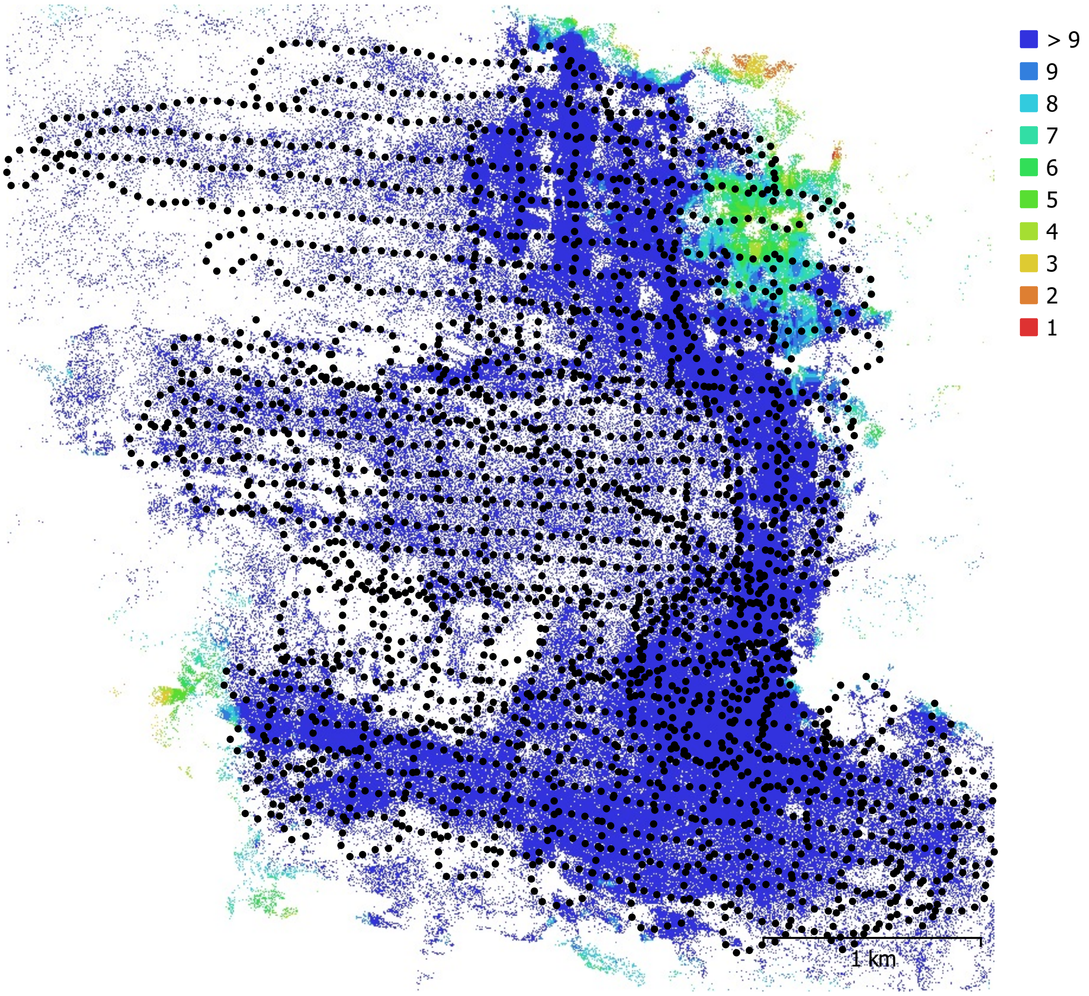

Fig. 1. Camera locations and image overlap.

Number of images:	2,595	Camera stations:	2,575
Flying altitude:	350 m	Tie points:	695,557
Ground resolution:	6.23 cm/pix	Projections:	1,423,470
Coverage area:	6.77 km²	Reprojection error:	0.174 pix

**Camera Model**	**Resolution**	**Focal Length**	**Pixel Size**	**Precalibrated**
NX500 (20 mm)	6480 x 4320	20 mm	3.7 x 3.7 μm	No
NX500 (20 mm)	6480 x 4320	20 mm	3.7 x 3.7 μm	No
NX500 (20 mm)	6480 x 4320	20 mm	3.7 x 3.7 μm	No
NX500 (20 mm)	6480 x 4320	20 mm	3.7 x 3.7 μm	No
NX500 (20 mm)	6480 x 4320	20 mm	3.7 x 3.7 μm	No

Camera Model	Resolution	Focal Length	Pixel Size	Precalibrated
NX500 (20 mm)	6480 x 4320	20 mm	3.7 x 3.7 μm	No

Table 1. Cameras.

Camera Calibration

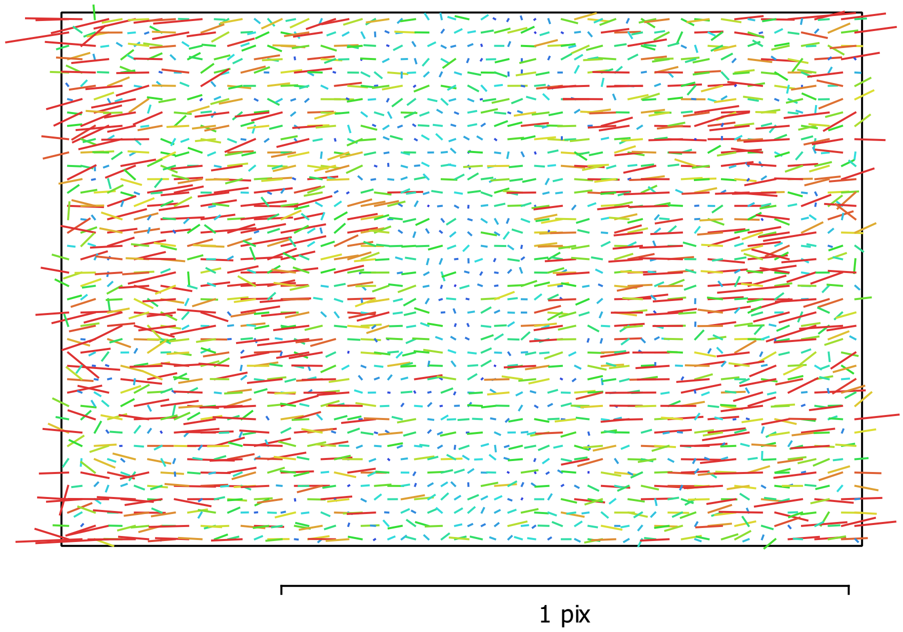

Fig. 2. Image residuals for NX500 (20 mm).

NX500 (20 mm)

200 images, additional corrections

Type	Resolution	Focal Length	Pixel Size
**Frame**	**6480 x 4320**	**20 mm**	**3.7 x 3.7 μm**

F:	5619.66		
Cx:	86.131	B1:	4.10482
Cy:	11.4315	B2:	1.10117
K1:	-0.0120668	P1:	0.00210335
K2:	0.0240065	P2:	-0.00149497
K3:	-0.00416849	P3:	0
K4:	-0.0285848	P4:	0

Camera Calibration

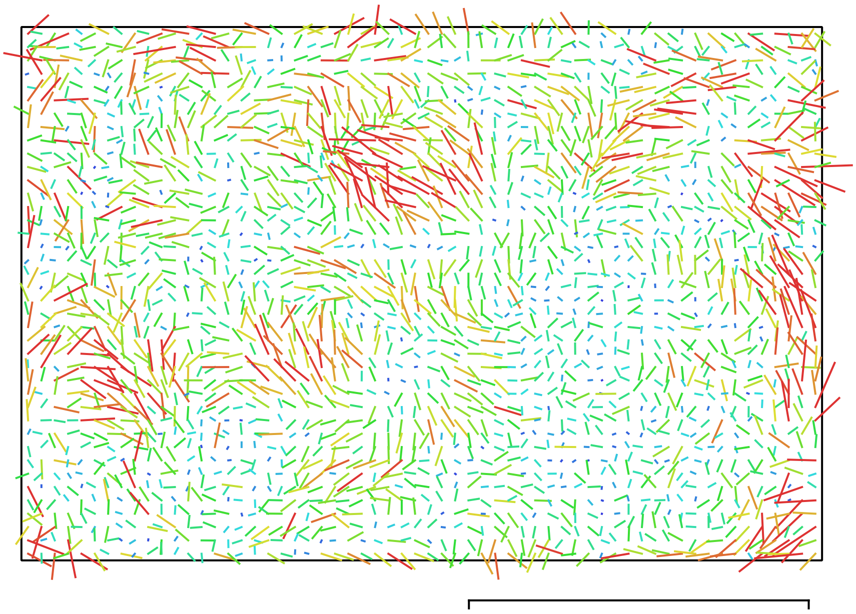

Fig. 3. Image residuals for NX500 (20 mm).

NX500 (20 mm)

462 images, additional corrections

Type	Resolution	Focal Length	Pixel Size
**Frame**	**6480 x 4320**	**20 mm**	**3.7 x 3.7 μm**

F:	5609.23		
Cx:	79.1935	B1:	-1.65068
Cy:	24.0398	B2:	-1.19284
K1:	0.0652604	P1:	0.00254286
K2:	-0.496071	P2:	-0.00126297
K3:	1.30992	P3:	0
K4:	-1.16548	P4:	0

Camera Calibration

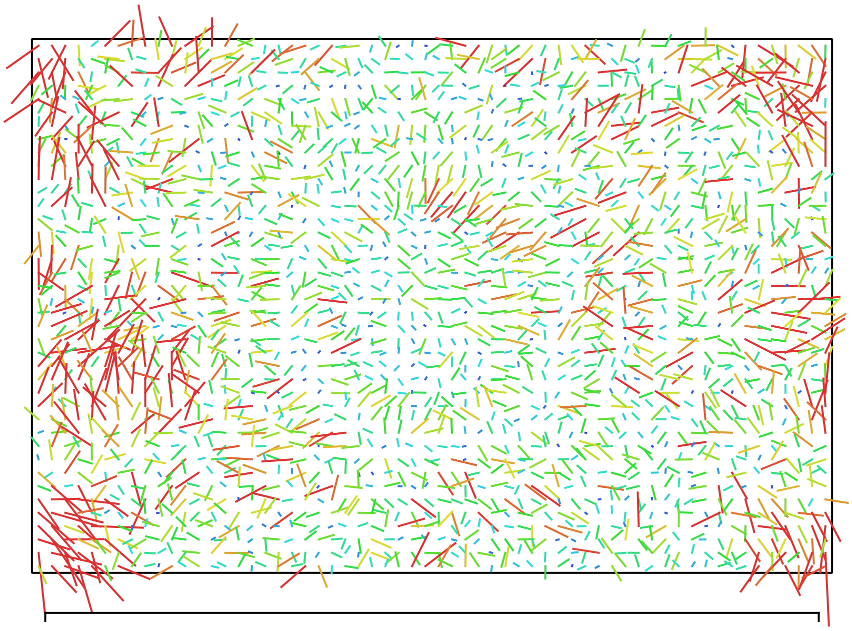

Fig. 4. Image residuals for NX500 (20 mm).

NX500 (20 mm)

530 images, additional corrections

Type	Resolution	Focal Length	Pixel Size
**Frame**	**6480 x 4320**	**20 mm**	**3.7 x 3.7 µm**

F:	5632.32		
Cx:	78.3883	B1:	0.43823
Cy:	35.6528	B2:	0.221069
K1:	-0.0252445	P1:	0.00204111
K2:	0.122176	P2:	0.0009678
K3:	-0.276335	P3:	0
K4:	0.219447	P4:	0

Camera Calibration

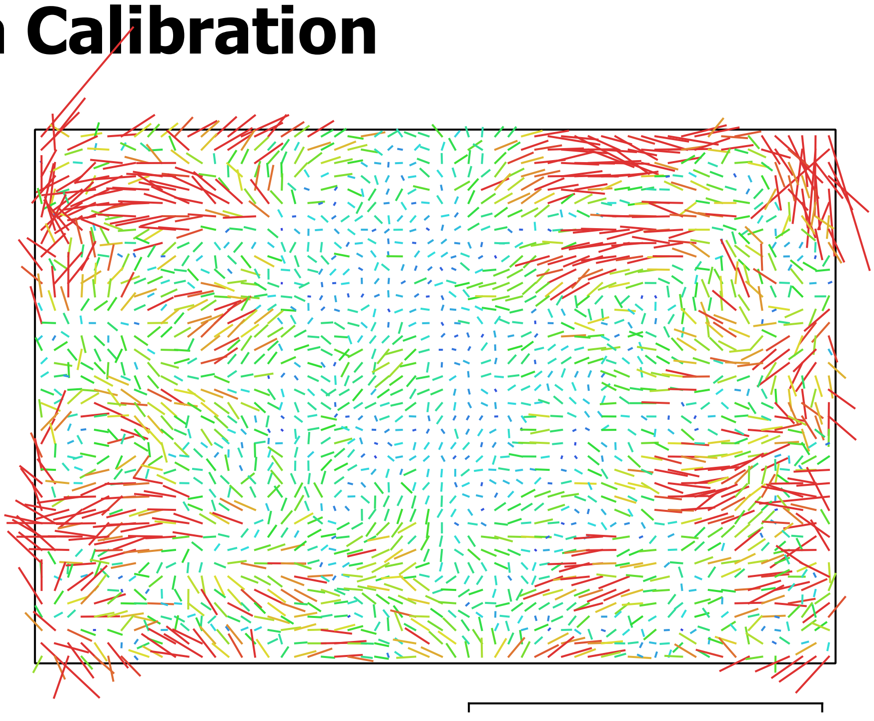

Fig. 5. Image residuals for NX500 (20 mm).

NX500 (20 mm)

513 images, additional corrections

Type	Resolution	Focal Length	Pixel Size
**Frame**	**6480 x 4320**	**20 mm**	**3.7 x 3.7 µm**

F:	5630.85		
Cx:	92.1764	B1:	-1.72615
Cy:	77.7022	B2:	-0.128631
K1:	-0.057147	P1:	0.00254854
K2:	0.293143	P2:	0.00228051
K3:	-0.742001	P3:	0
K4:	0.730184	P4:	0

Camera Calibration

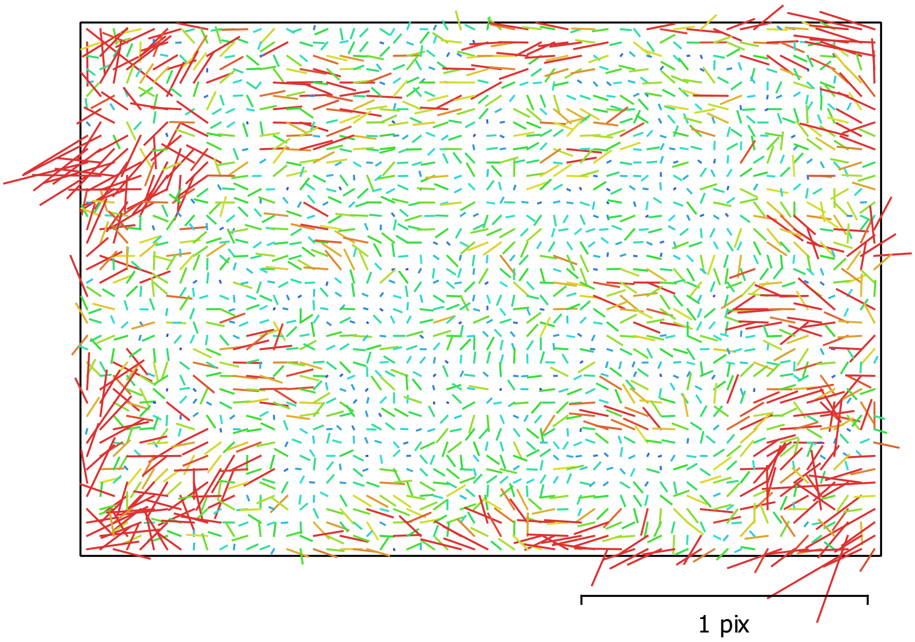

Fig. 6. Image residuals for NX500 (20 mm).

NX500 (20 mm)

412 images, additional corrections

Type	Resolution	Focal Length	Pixel Size
**Frame**	**6480 x 4320**	**20 mm**	**3.7 x 3.7 µm**

F:	5616.38		
Cx:	101.498	B1:	5.35161
Cy:	67.5566	B2:	-0.857203
K1:	-0.0120239	P1:	0.00371294
K2:	0.129195	P2:	0.00221453
K3:	-0.468739	P3:	0
K4:	0.486733	P4:	0

Camera Calibration

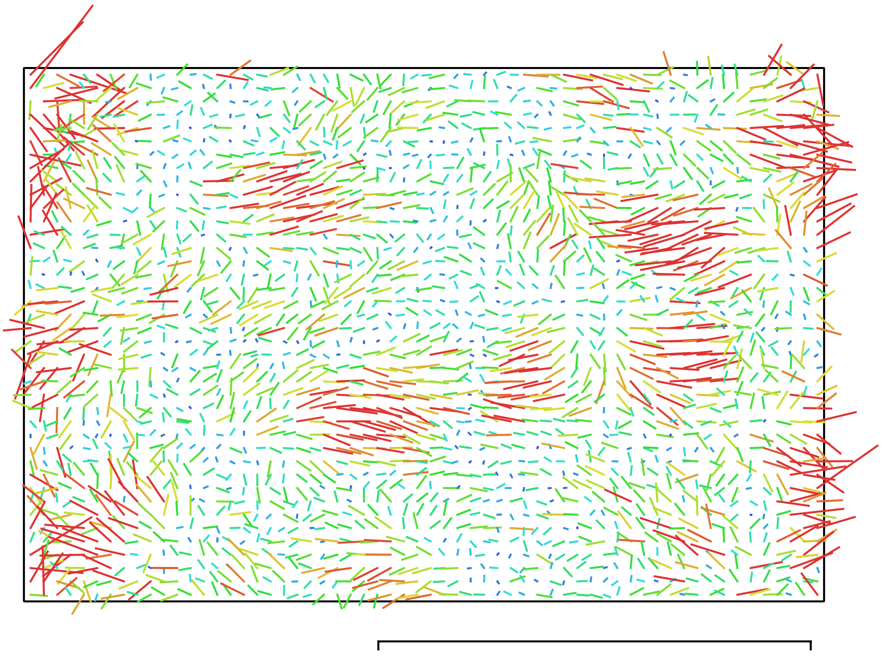

Fig. 7. Image residuals for NX500 (20 mm).

NX500 (20 mm)

478 images, additional corrections

Type	Resolution	Focal Length	Pixel Size
**Frame**	**6480 x 4320**	**20 mm**	**3.7 x 3.7 µm**

F:	5624.46		
Cx:	85.6809	B1:	4.82418
Cy:	27.6852	B2:	-1.03047
K1:	0.0194152	P1:	0.00357091
K2:	-0.233743	P2:	-0.00054549
K3:	0.831008	P3:	0
K4:	-0.954537	P4:	0

Ground Control Points

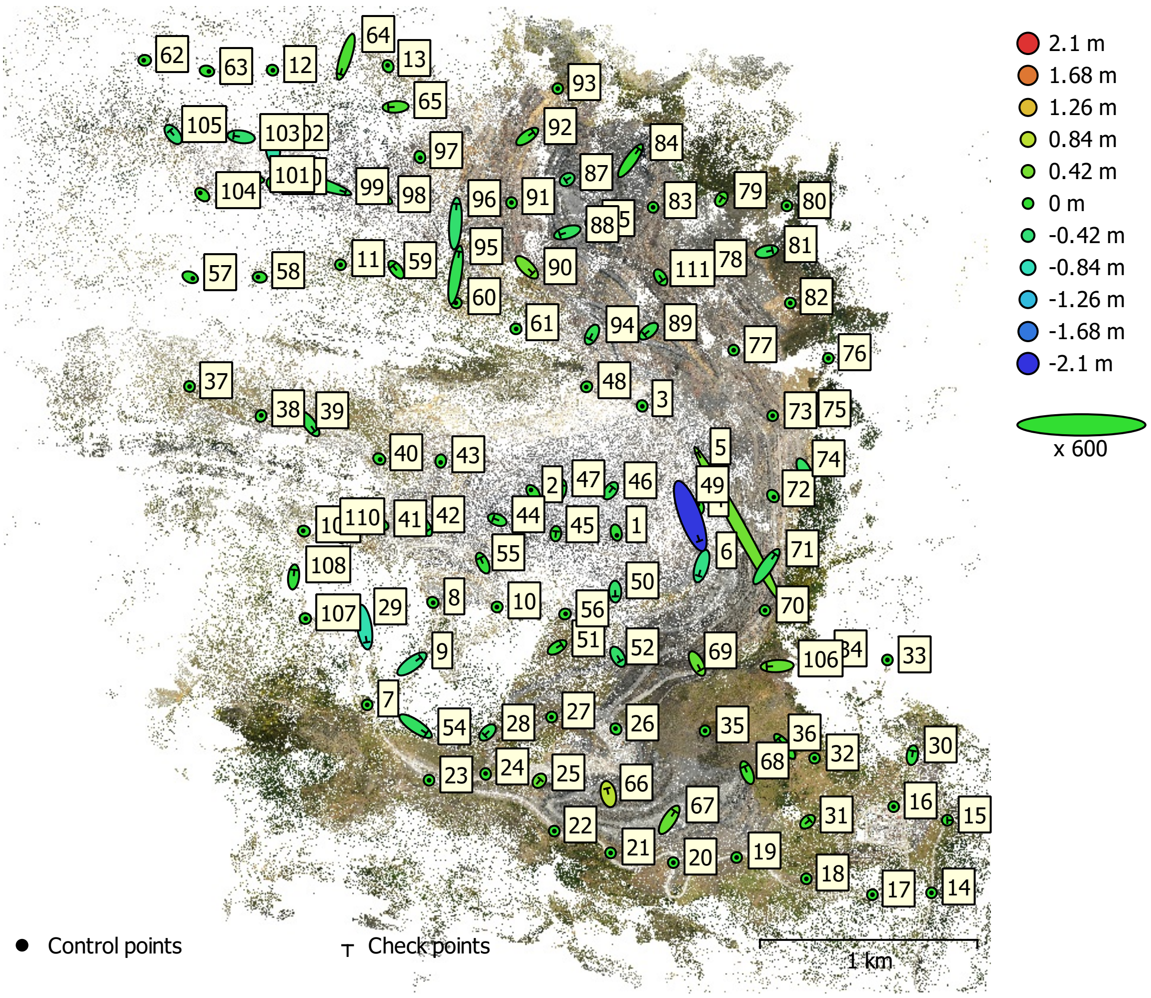

Fig. 8. GCP locations and error estimates.
Z error is represented by ellipse color. X,Y errors are represented by ellipse shape.
Estimated GCP locations are marked with a dot or crossing.

Count	X error (cm)	Y error (cm)	Z error (cm)	XY error (cm)	Total (cm)
55	1.09839	1.15689	0.890165	1.59526	1.82681

Table 2. Control points RMSE.
X - Easting, Y - Northing, Z - Altitude.

Count	X error (cm)	Y error (cm)	Z error (cm)	XY error (cm)	Total (cm)
54	13.3372	19.4819	40.1507	23.6098	46.578

Table 3. Check points RMSE.
X - Easting, Y - Northing, Z - Altitude.

Label	X error (cm)	Y error (cm)	Z error (cm)	Total (cm)	Image (pix)
1	1.12055	-4.25924	-3.81166	5.82456	0.274 (104)
2	-3.34763	4.20997	0.144141	5.38063	0.279 (109)
3	0.198781	-0.592823	0.212006	0.660227	0.033 (51)
4	-0.725903	3.54438	4.224	5.56163	0.304 (50)
7	0.018883	0.00909578	-0.0153265	0.0259654	0.006 (24)
8	0.591791	-0.474556	-0.435425	0.874651	0.169 (32)
10	-0.306538	-0.0523145	1.5464	1.57736	0.213 (42)
11	0.207032	-0.0515672	0.366601	0.424167	0.110 (36)
12	0.592935	-0.261773	-0.287734	0.709145	0.205 (26)
13	-0.666961	1.34137	0.297558	1.5273	0.131 (20)
14	-0.0363784	-0.0382488	0.00560429	0.0530827	0.006 (23)
16	-0.0454516	-0.0035682	-0.00240979	0.045655	0.010 (34)
17	0.0663384	0.0155479	-0.00809156	0.0686148	0.006 (23)
18	0.0431654	-0.100589	0.00379237	0.109525	0.011 (25)
19	0.102793	0.0832674	-0.0240712	0.134459	0.014 (20)
20	0.0801188	-0.0222452	-0.0525343	0.0983551	0.015 (16)
21	0.130953	-0.171176	0.078742	0.229456	0.021 (15)
22	0.107414	-0.0479019	0.0507112	0.128078	0.016 (13)
23	0.0941474	-0.144689	-0.00440643	0.172679	0.013 (18)
24	-0.130435	0.760817	-0.109289	0.779616	0.073 (27)
26	-0.626096	0.708369	-0.214372	0.969401	0.032 (33)
27	-0.29256	-0.234397	0.0142156	0.375148	0.053 (27)
32	-0.0479279	-0.0228218	-0.0112621	0.0542656	0.012 (18)
33	0.00202403	-0.000139751	-0.000643206	0.00212837	0.000 (3)
34	-0.00560113	0.00234804	0.00614042	0.0086366	0.001 (4)
35	0.0274259	0.227975	0.0159165	0.23017	0.026 (11)
37	0.0702089	-0.128359	0.471972	0.494129	0.058 (46)
38	0.489668	0.85972	-0.672735	1.19644	0.101 (57)
40	1.31328	-0.804538	-0.450969	1.60479	0.143 (66)
43	-0.186628	-2.02845	-0.48859	2.09479	0.236 (69)
48	-0.54982	-0.307426	-0.106904	0.638938	0.020 (44)

Label	X error (cm)	Y error (cm)	Z error (cm)	Total (cm)	Image (pix)
56	0.843037	0.254145	0.235824	0.911545	0.204 (50)
57	3.96	-1.70567	-0.5149	4.34235	0.411 (14)
58	-2.65496	0.263451	-0.337782	2.68929	0.218 (30)
60	-0.405649	0.0167803	-0.120259	0.423432	0.030 (32)
61	0.399933	-0.039135	0.00662422	0.401898	0.027 (20)
62	-1.57272	-0.0180231	0.268738	1.59562	0.199 (17)
63	3.0359	-0.59946	0.0331354	3.09469	0.355 (16)
70	-0.0352585	0.0892369	-0.135148	0.165745	0.011 (30)
72	1.3602	-1.63792	-0.995018	2.35011	0.108 (18)
73	0.034758	0.00112276	0.0243541	0.0424559	0.015 (15)
76	0.0141781	-0.0189294	0.000400652	0.0236538	0.009 (9)
77	0.0810275	-0.0353294	0.0200527	0.0906407	0.014 (11)
78	-0.065733	-0.0181655	0.0278131	0.0736504	0.047 (11)
80	0.00950199	0.041549	-0.00770619	0.0433127	0.012 (7)
82	-0.124668	-0.0202582	-0.0158973	0.1273	0.027 (6)
83	0.0139306	0.0876283	-0.00637565	0.0889575	0.036 (13)
85	-0.0966036	0.0363673	0.0280159	0.106957	0.023 (10)
91	-0.163241	0.422053	0.116624	0.467309	0.029 (20)
93	0.00652622	0.0163981	-0.0374774	0.0414252	0.017 (16)
97	0.494265	-1.14821	-0.233127	1.27162	0.132 (40)
100	0.254771	-1.00665	-1.29597	1.66066	0.307 (28)
104	-3.1319	2.66476	1.81056	4.49309	0.522 (21)
107	0.599367	-0.0926378	-0.0538031	0.608865	0.040 (21)
109	-1.15138	0.365884	0.703332	1.39794	0.103 (22)
**Total**	**1.09839**	**1.15689**	**0.890165**	**1.82681**	**0.179**

Table 4. Control points.

X - Easting, Y - Northing, Z - Altitude.

Label	X error (cm)	Y error (cm)	Z error (cm)	Total (cm)	Image (pix)
5	-60.9633	109.955	37.7439	131.268	0.162 (38)
6	-4.47519	-15.0551	-51.1742	53.5302	0.070 (66)
9	13.6348	9.9612	-45.1784	48.2309	0.083 (29)

Label	X error (cm)	Y error (cm)	Z error (cm)	Total (cm)	Image (pix)
15	-0.233368	-0.00361737	0.453119	0.509697	0.013 (27)
25	1.73145	2.00841	30.4733	30.5884	0.023 (22)
28	-4.46056	-4.33082	-23.8096	24.6079	0.028 (29)
29	3.69631	-23.2215	-76.3502	79.889	0.004 (18)
30	1.03551	6.57383	-10.6515	12.5595	0.006 (20)
31	3.43779	2.86221	9.0576	10.102	0.012 (26)
36	-9.67118	11.9434	2.96358	15.6512	0.023 (14)
39	8.16964	-11.1284	-15.3408	20.6379	0.103 (40)
41	6.40958	-2.65256	-11.2896	13.2505	0.169 (56)
42	5.62282	-8.01767	-15.3974	18.2477	0.167 (48)
44	-6.08762	2.31694	-7.19027	9.70192	0.297 (73)
45	0.335101	3.54457	-0.968613	3.68978	0.277 (94)
46	3.74016	4.99999	-19.9915	20.944	0.207 (63)
47	1.7122	7.7431	-8.98486	11.9839	0.318 (107)
49	14.2874	-38.3462	-207.18	211.183	0.388 (59)
50	0.227084	-7.3158	-32.6982	33.5074	0.188 (56)
51	6.89792	4.38637	-1.95624	8.40526	0.161 (42)
52	3.86612	-6.57662	-33.0563	33.9252	0.134 (56)
54	16.7141	-11.2147	-27.5955	34.1561	0.014 (31)
55	-3.81435	7.98143	-4.32632	9.84731	0.215 (51)
59	-5.06994	6.28565	-0.318689	8.08178	0.095 (9)
64	-8.37655	-26.6992	15.8007	32.1353	0.153 (13)
65	-11.2093	-0.631184	16.5208	19.9746	0.167 (31)
66	-2.0777	8.71328	82.8308	83.3137	0.029 (22)
67	8.68688	13.2956	31.2985	35.0974	0.024 (12)
68	-3.92332	9.42132	7.88341	12.8958	0.025 (16)
69	5.36486	-9.96033	30.7531	32.768	0.020 (28)
71	13.5155	19.0326	-26.1948	35.0867	0.065 (24)
74	5.21299	-10.5147	-29.096	31.3737	0.013 (10)
75	3.03678	2.84484	-5.75276	7.09996	0.005 (6)
79	1.92276	3.0052	10.1917	10.7981	0.017 (7)
81	8.67545	1.99084	-22.0542	23.7827	0.048 (6)

Label	X error (cm)	Y error (cm)	Z error (cm)	Total (cm)	Image (pix)
84	13.0606	18.1699	1.70817	22.442	0.017 (12)
87	-2.50603	-1.57856	-26.8989	27.0615	0.017 (10)
88	-11.4922	-3.61913	-25.2091	27.9404	0.021 (13)
89	-6.53198	-5.38582	-14.9209	17.1554	0.018 (27)
90	9.13967	-9.02728	38.235	40.3353	0.018 (28)
92	9.52868	6.8132	2.31786	11.941	0.018 (18)
94	-3.63717	-6.86682	-19.1164	20.6354	0.030 (35)
95	5.60316	35.7585	-19.9787	41.3427	0.048 (38)
96	1.76849	30.0702	-36.5536	47.3657	0.030 (23)
98	16.8238	-6.51564	-2.76922	18.2527	0.201 (32)
99	47.7467	-15.8741	-21.4209	54.6862	0.245 (30)
101	-22.4895	2.28088	-11.0772	25.1731	0.409 (26)
102	-1.60039	14.4089	-70.4566	71.9327	0.337 (21)
103	-11.5264	1.55477	-36.1344	37.9601	0.427 (20)
105	-4.8934	5.93459	-37.2447	38.0307	0.309 (24)
106	-16.0395	-0.794378	29.6965	33.7606	0.004 (13)
108	1.15345	10.8287	-4.9683	11.9698	0.071 (19)
110	-8.50964	12.0575	-24.4257	28.5379	0.082 (28)
111	3.12773	-4.59134	-2.58815	6.12875	0.019 (14)
**Total**	**13.3372**	**19.4819**	**40.1507**	**46.578**	**0.197**

Table 5. Check points.

X - Easting, Y - Northing, Z - Altitude.

Digital Elevation Model

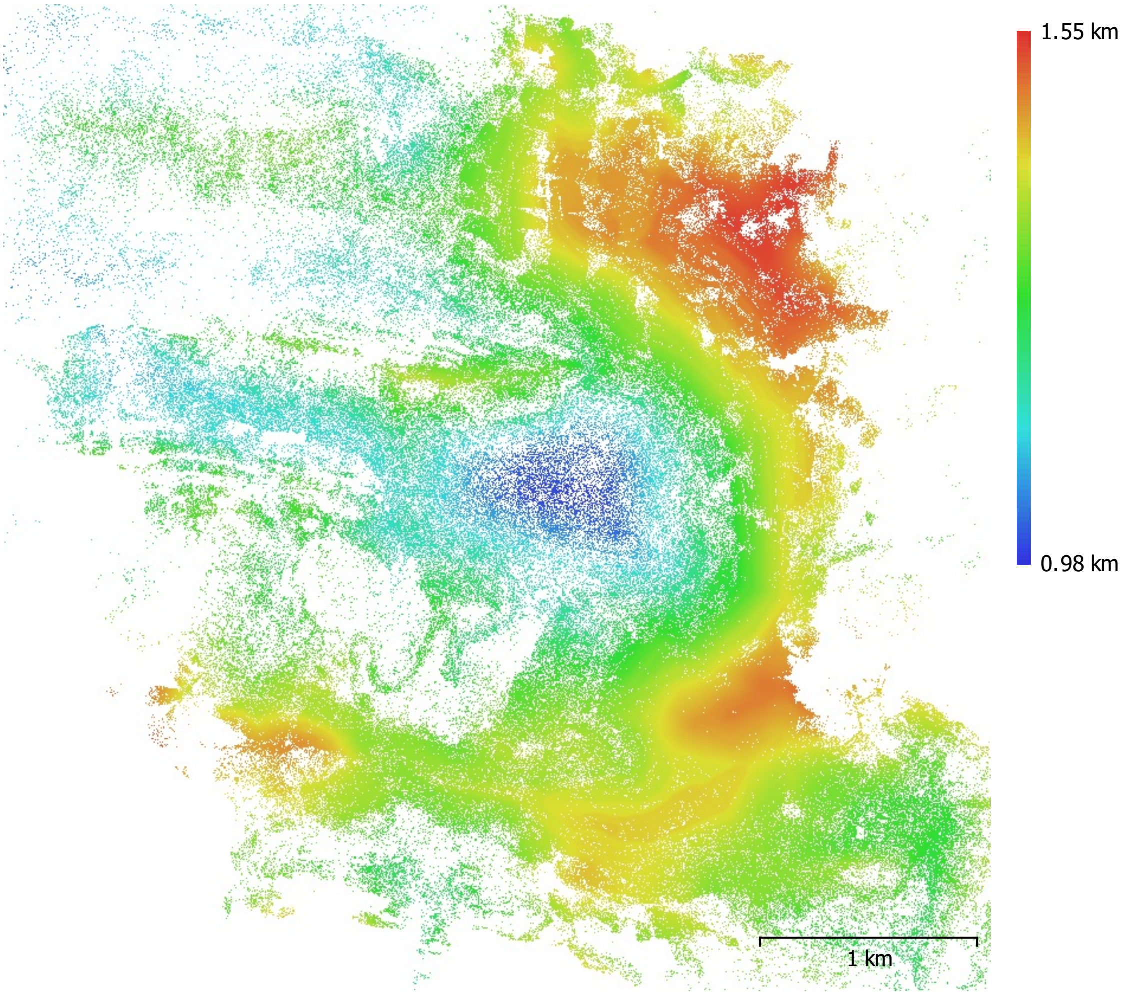

Fig. 9. Reconstructed digital elevation model.

Resolution:	unknown
Point density:	unknown

Processing Parameters

**General**	
Cameras	2595
Aligned cameras	2575
Markers	110
**Shapes**	
Polygon	1
Coordinate system	ETRS89 / UTM zone 30N (EPSG::25830)
Rotation angles	Yaw, Pitch, Roll
**Tie Points**	
Points	695,557 of 12,529,745
RMS reprojection error	0.0745317 (0.173758 pix)
Max reprojection error	1.18003 (2.44598 pix)
Mean key point size	2.29942 pix
Point colors	3 bands, uint8
Key points	No
Average tie point multiplicity	3.65511
**Alignment parameters**	
Accuracy	High
Generic preselection	Yes
Reference preselection	No
Key point limit	60,000
Key point limit per Mpx	1,000
Tie point limit	0
Exclude stationary tie points	Yes
Guided image matching	No
Adaptive camera model fitting	No
Matching time	4 hours 7 minutes
Matching memory usage	3.73 GB
Alignment time	2 hours 17 minutes
Alignment memory usage	4.82 GB
**Optimization parameters**	
Parameters	f, b1, b2, cx, cy, k1-k4, p1, p2
Fit additional corrections	Yes
Adaptive camera model fitting	No
Optimization time	13 minutes 23 seconds
Date created	2023:11:13 15:04:46
Software version	2.0.0.15597
File size	750.92 MB
**System**	
Software name	Agisoft Metashape Professional
Software version	2.0.3 build 16960
OS	Windows 64 bit
RAM	63.90 GB
CPU	Intel(R) Core(TM) i7-7700 CPU @ 3.60GHz
GPU(s)	Quadro M4000
