# Supplementary material for: Automated cleaning of tie point clouds following USGS guidelines in Agisoft Metashape professional (ver. 2.1.0)
Source: MethodsX. 2024 Mar 26;12:102679. doi: 10.1016/j.mex.2024.102679 (PMC10992719; doi:10.1016/j.mex.2024.102679)
Supplement: Supplementary file 3 — The supplementary material includes supplementary text, figures and the processing reports generated by the software. [file mmc3.zip › Urft_SCC-Optimized_r3.pdf]

Urft_SCC-Optimized_r3

Automatically cleaned sparse cloud using the SCC script (optimized settings). UAS data provided by Stauch et al. (2023).

Stauch, G., Dörwald, L., Esch, A., and Walk, J.: 115 years of sediment deposition in a reservoir in Central Europe: Topographic change detection, Earth Surface Processes and Landforms, doi: 10.1002/esp.5722, 2023.

29 December 2023

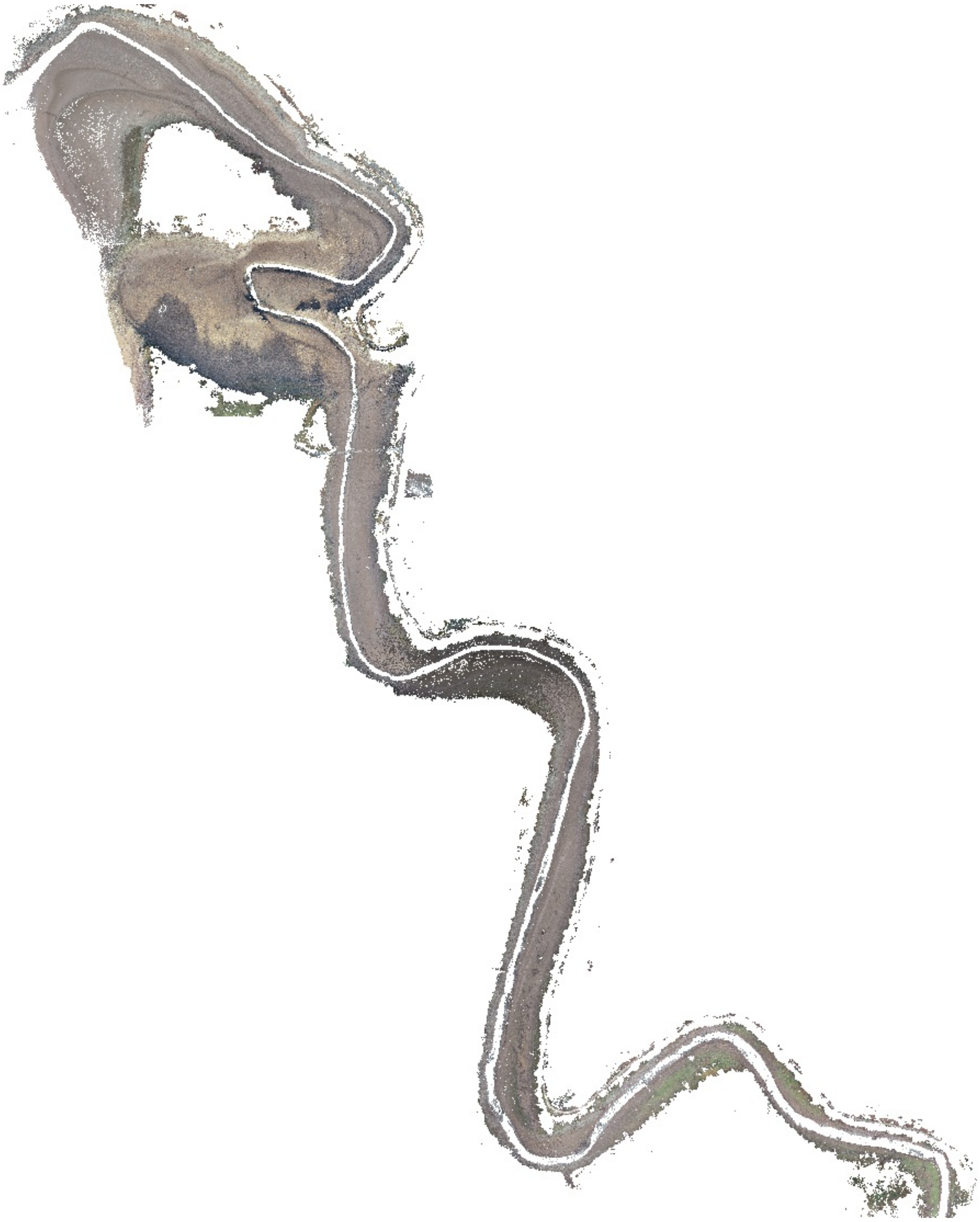

Survey Data

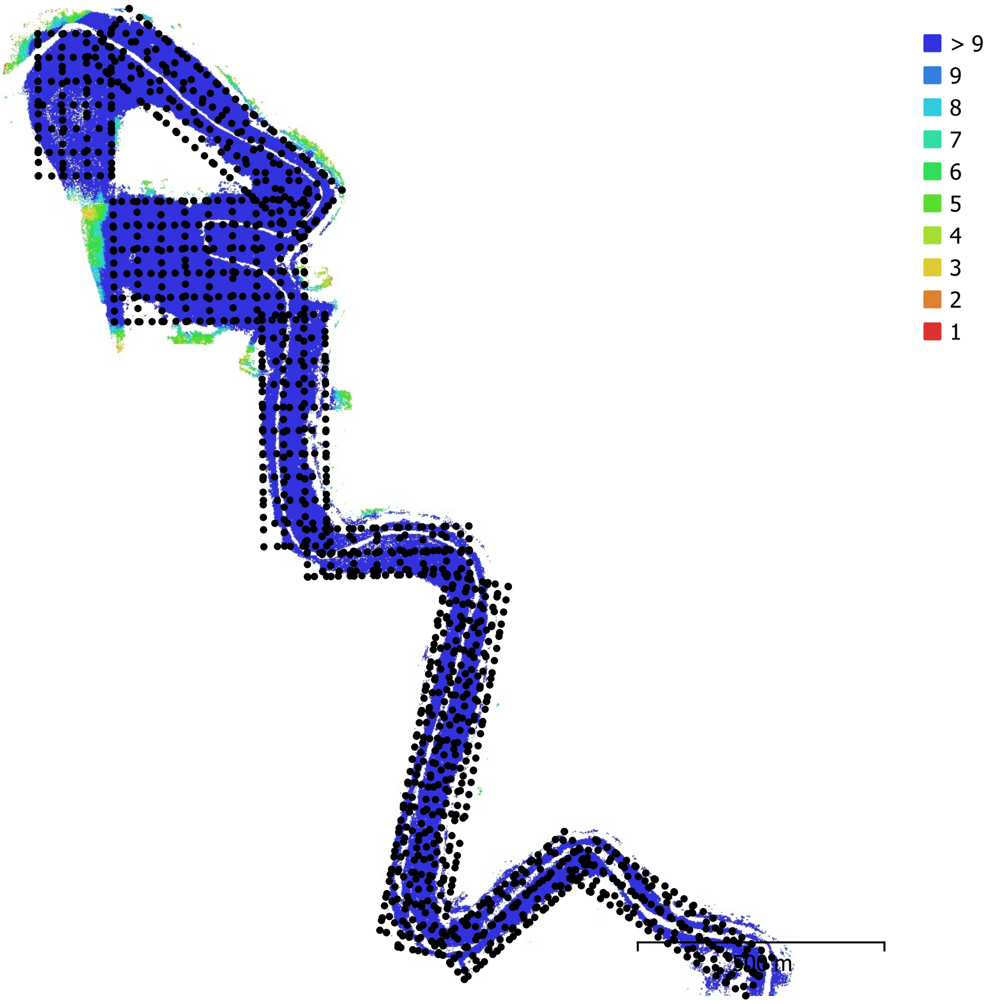

Fig. 1. Camera locations and image overlap.

Number of images:	1,527	Camera stations:	1,498
Flying altitude:	90 m	Tie points:	1,226,122
Ground resolution:	2.46 cm/pix	Projections:	3,165,136
Coverage area:	0.415 km²	Reprojection error:	0.28 pix

Camera Model	Resolution	Focal Length	Pixel Size	Precalibrated
FC6310S (8.8mm)	5472 x 3648	8.8 mm	2.41 x 2.41 µm	No

Table 1. Cameras.

Camera Calibration

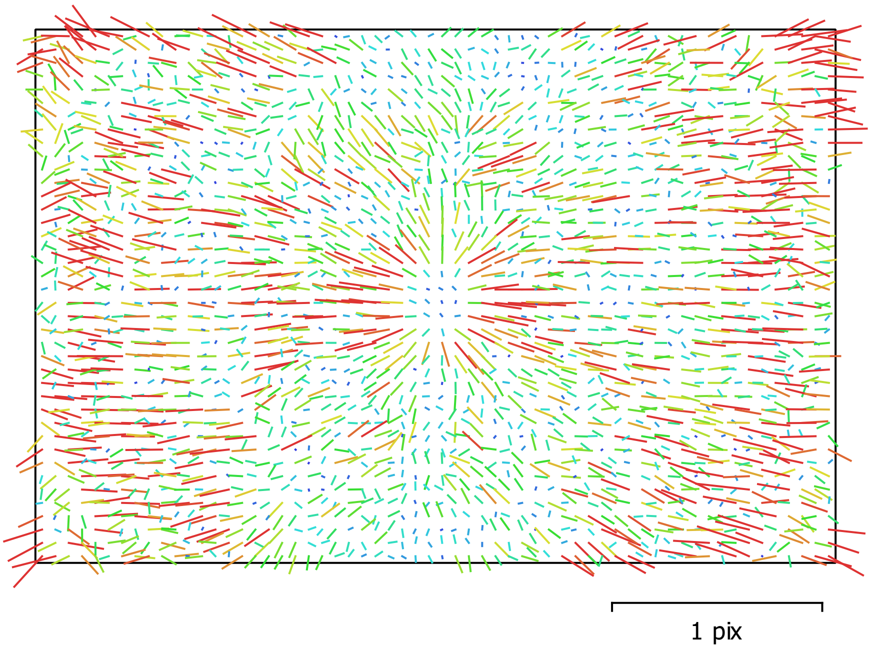

Fig. 2. Image residuals for FC6310S (8.8mm).

FC6310S (8.8mm)

1527 images

Type	Resolution	Focal Length	Pixel Size
**Frame**	**5472 x 3648**	**8.8 mm**	**2.41 x 2.41 µm**

F:	3655.85		
Cx:	0.389949	B1:	0
Cy:	36.9572	B2:	0
K1:	0.0014484	P1:	0.00016332
K2:	-0.0149768	P2:	0.00214924
K3:	0.0146666	P3:	0
K4:	0	P4:	0

Ground Control Points

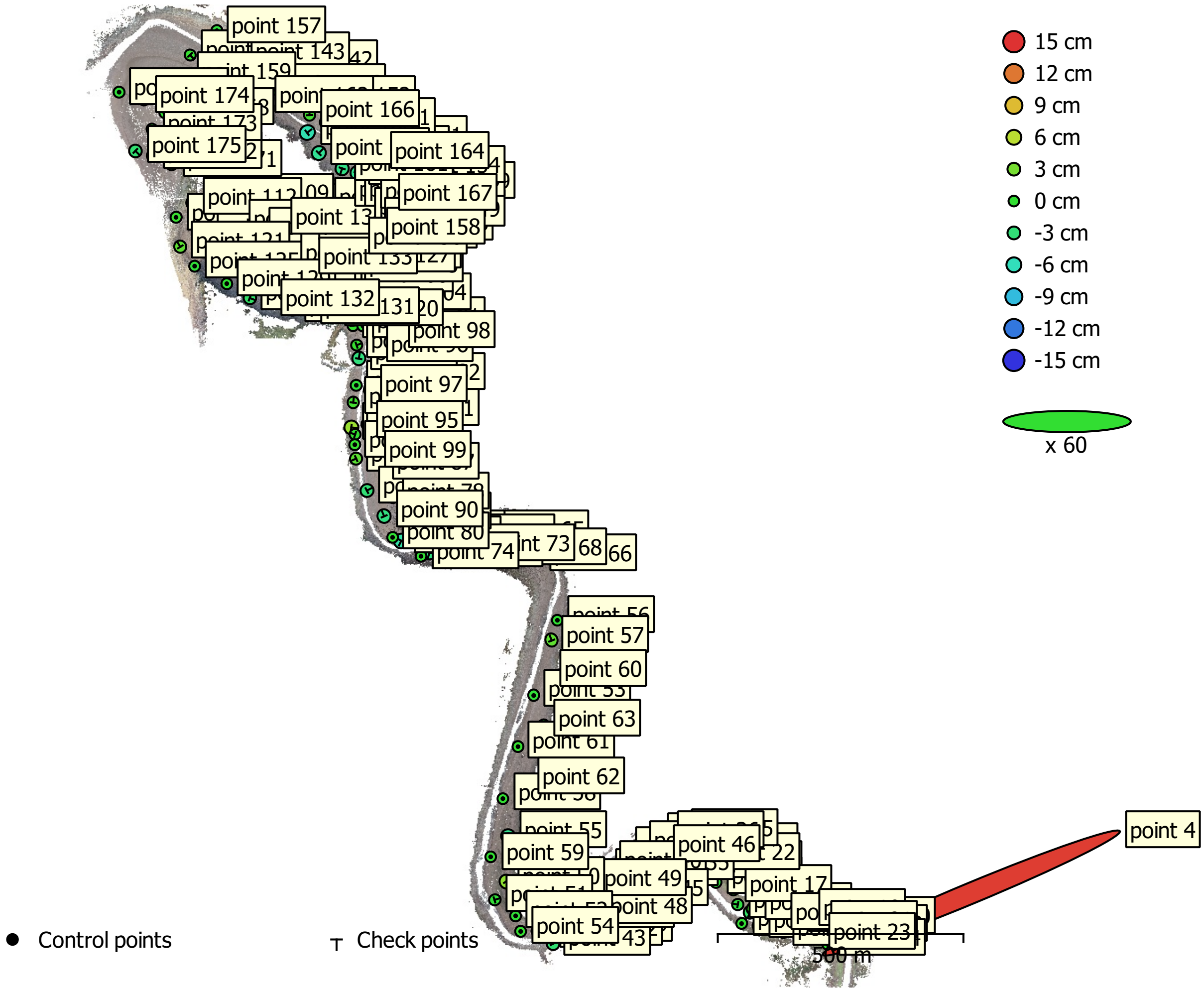

Fig. 3. GCP locations and error estimates.
Z error is represented by ellipse color. X,Y errors are represented by ellipse shape.
Estimated GCP locations are marked with a dot or crossing.

Count	X error (m)	Y error (m)	Z error (m)	XY error (m)	Total (m)
85	0.00658491	0.00790091	0.00490396	0.0102852	0.0113945

Table 2. Control points RMSE.
X - Longitude, Y - Latitude, Z - Altitude.

Count	X error (m)	Y error (m)	Z error (m)	XY error (m)	Total (m)
85	1.02182	0.425251	0.031451	1.10678	1.10722

Table 3. Check points RMSE.
X - Longitude, Y - Latitude, Z - Altitude.

Label	X error (m)	Y error (m)	Z error (m)	Total (m)	Image (pix)
point 1	-0.00596992	-0.0156384	-0.00367289	0.0171374	0.365 (24)
point 5	-0.00890268	-0.0115867	-0.000209377	0.0146135	0.318 (31)
point 8	0.000376371	0.0030698	4.79046e-05	0.00309316	0.304 (24)
point 12	-0.00603613	0.00498238	0.00297433	0.00837291	0.320 (26)
point 13	-0.00525474	0.0143806	-0.00763895	0.0171105	0.425 (26)
point 14	-0.00730783	-0.0148867	0.000198004	0.0165848	0.462 (26)
point 16	0.00576027	0.00742736	0.00706464	0.0117582	0.352 (27)
point 17	0.00500542	0.00934919	0.00467324	0.0115888	0.314 (26)
point 18	0.008671	-0.0131472	-0.0135643	0.0207852	0.454 (25)
point 19	0.00786357	0.0125826	0.00801007	0.0168617	0.367 (19)
point 20	0.00750487	0.00411354	0.0031512	0.00912	0.297 (26)
point 22	0.00267002	0.00889647	-0.00341617	0.00989679	0.291 (27)
point 23	-0.00279651	-0.00407848	0.00187238	0.00528775	0.278 (27)
point 26	0.0033248	-0.00171054	-0.00497601	0.00622422	0.298 (30)
point 27	-0.00462582	0.0132742	0.00564041	0.0151465	0.428 (32)
point 29	-0.00309491	0.000401673	0.000959541	0.00326504	0.313 (27)
point 30	-0.00242238	0.00658518	0.00408002	0.00811659	0.340 (27)
point 31	-0.0126489	0.00150419	0.00693951	0.0145056	0.319 (26)
point 35	-0.00109481	-0.0100583	0.00412474	0.0109262	0.318 (25)
point 38	-0.010032	-0.00851547	-0.00858349	0.0157108	0.357 (26)
point 39	0.00521779	-0.0183961	0.00171463	0.0191985	0.339 (26)
point 40	-0.00129148	-0.00149836	-0.000741443	0.00211252	0.259 (33)
point 41	0.00557132	-0.00133244	-0.00500748	0.00760854	0.330 (26)
point 44	-0.000134351	0.00902458	-0.00426949	0.00998447	0.345 (25)
point 45	-0.000693028	0.0130265	0.00120596	0.0131005	0.299 (26)
point 49	0.015637	-0.00300956	-0.000145621	0.0159247	0.291 (30)
point 52	0.00183542	0.00353442	-0.00254755	0.00472767	0.288 (28)
point 53	0.00170424	-0.0211621	-0.00268834	0.0214001	0.394 (25)
point 54	0.00288334	-0.00774188	0.0018546	0.00846699	0.249 (20)
point 56	0.00243086	-0.00222174	-0.000747291	0.00337693	0.254 (28)
point 58	0.000909554	0.00452864	-2.04842e-06	0.00461907	0.210 (22)

Label	X error (m)	Y error (m)	Z error (m)	Total (m)	Image (pix)
point 59	0.000145735	-0.00208166	0.000121007	0.00209026	0.195 (25)
point 60	-0.00728379	0.0145938	0.00239139	0.0164849	0.358 (33)
point 61	-0.00109354	-0.00284594	0.00227535	0.00380426	0.277 (27)
point 62	-0.00495217	-0.00224184	-0.000859563	0.00550351	0.227 (27)
point 63	0.00693549	0.0104121	-0.000504008	0.0125207	0.305 (25)
point 65	-0.00382953	-0.00382593	-0.00121202	0.00554726	0.258 (27)
point 66	0.00278334	0.00243566	0.000405012	0.00372068	0.225 (25)
point 69	0.00840641	0.0101518	0.00221152	0.0133648	0.264 (27)
point 73	0.00104106	0.0028832	0.0013145	0.00333535	0.250 (22)
point 74	-0.00303412	-0.00664465	-0.000667502	0.00733504	0.231 (29)
point 80	-0.00414033	-0.00413355	-0.000715779	0.00589415	0.372 (13)
point 84	0.00289927	0.00215545	0.00396842	0.00536657	0.266 (18)
point 85	0.00565655	0.00020319	-0.00381225	0.00682431	0.308 (19)
point 87	-0.00203862	-0.0011523	-0.00188475	0.00300601	0.348 (19)
point 91	-0.00202015	0.0070864	0.00056993	0.00739073	0.268 (16)
point 94	0.0101355	-0.00649426	-0.00166419	0.0121521	0.303 (20)
point 95	0.00404373	-0.0067095	-0.00407575	0.00883068	0.305 (21)
point 97	-0.0132992	-0.00447953	0.00190551	0.0141621	0.266 (18)
point 98	-0.00517026	0.0103087	-0.00207673	0.0117181	0.285 (17)
point 100	0.0179933	-0.000478526	-0.00175876	0.0180854	0.373 (17)
point 101	-0.00635325	-0.00608178	0.00433091	0.0098035	0.442 (21)
point 102	-0.00817577	-0.00481925	0.00409318	0.0103355	0.709 (6)
point 105	0.00443546	0.00177305	-0.00391521	0.00617624	0.307 (21)
point 110	-0.00270505	0.00552876	0.00787349	0.00999382	0.356 (19)
point 115	-0.0184807	-0.00222362	0.00127717	0.0186577	0.433 (17)
point 116	-0.00454985	0.0172655	-0.00684916	0.0191236	0.421 (21)
point 117	0.00200124	0.00120862	-0.00764742	0.0079968	0.529 (19)
point 119	0.00280439	-0.00871774	0.00419382	0.0100723	0.488 (21)
point 122	0.015065	-0.00180113	-0.0151345	0.0214302	0.654 (15)
point 123	-0.00282902	-0.00234331	0.00480791	0.00605066	0.396 (18)
point 124	-0.00557945	-5.85832e-05	0.00836246	0.0100531	0.331 (23)
point 125	0.000461766	0.00266269	-0.00276009	0.0038628	0.445 (13)

Label	X error (m)	Y error (m)	Z error (m)	Total (m)	Image (pix)
point 127	-0.00601369	-0.00733336	0.00574964	0.0110906	0.389 (18)
point 128	0.00653116	-0.00761777	0.00834804	0.0130528	0.362 (17)
point 129	-0.00371711	0.00621984	-0.00129785	0.00736123	0.474 (18)
point 130	0.0135529	-0.00543136	-0.00242972	0.0148015	0.350 (18)
point 133	0.00485191	-0.00978791	-0.00538141	0.012178	0.524 (22)
point 136	-0.00262458	-0.00311871	0.00901087	0.00988992	0.670 (12)
point 139	0.00541337	-0.00383852	-0.00389212	0.00769334	0.410 (19)
point 142	0.00698923	-0.00478067	0.00468429	0.00967712	0.336 (17)
point 145	-0.00280386	0.0194939	-0.00555713	0.0204635	0.361 (18)
point 146	0.00642657	0.00215396	-0.00120897	0.00688491	0.491 (19)
point 147	0.000778342	0.000124504	0.00114338	0.00138876	0.400 (18)
point 151	0.00251748	0.00253815	0.00235965	0.00428344	0.356 (18)
point 154	0.00639777	0.00546816	-0.00364191	0.00917037	0.417 (18)
point 157	0.000264247	0.00051804	-0.00639288	0.00641927	0.440 (22)
point 158	-0.0111117	-0.000100406	-0.000650182	0.0111311	0.385 (11)
point 159	-0.00838809	0.00057408	0.00806242	0.0116487	0.393 (13)
point 162	-0.00896553	0.00164164	-0.0053253	0.0105563	0.424 (22)
point 164	-0.000151964	-0.0101826	0.0134878	0.0169005	0.581 (19)
point 167	-0.00809669	0.0132191	-0.00650301	0.0168104	0.347 (23)
point 168	0.000202909	-0.0035925	-0.000957067	0.00372333	0.307 (13)
point 170	0.00341964	0.000354266	-0.00311644	0.00464023	0.291 (15)
point 174	0.000195006	0.000245348	0.00264327	0.00266178	0.287 (20)
**Total**	**0.00658491**	**0.00790091**	**0.00490396**	**0.0113945**	**0.357**

Table 4. Control points.

X - Longitude, Y - Latitude, Z - Altitude.

Label	X error (m)	Y error (m)	Z error (m)	Total (m)	Image (pix)
point 2	-0.000897583	0.0312861	-0.00166154	0.031343	0.388 (25)
point 3	0.00907268	0.0235943	-0.0222492	0.0336754	0.307 (26)
point 4	-9.42011	-3.91828	0.145378	10.2036	0.355 (25)
point 6	0.00648885	0.0140723	-0.0176332	0.0234747	0.252 (27)
point 7	0.0053114	-0.00095781	-0.00787948	0.00955064	0.287 (24)

Label	X error (m)	Y error (m)	Z error (m)	Total (m)	Image (pix)
point 9	-0.026427	0.0290658	0.00870974	0.0402377	0.326 (24)
point 10	-0.0161916	-0.0407194	0.0712347	0.0836339	0.371 (17)
point 11	0.00293269	0.00106641	-0.000379981	0.00314361	0.229 (24)
point 15	0.0368801	0.0294832	0.010211	0.048308	0.353 (24)
point 21	0.0346427	0.033068	-0.034944	0.0592848	0.398 (28)
point 24	0.00345643	-0.00271563	-0.00204531	0.00484818	0.275 (28)
point 25	0.0179915	-0.00750475	-0.058161	0.061341	0.266 (10)
point 28	-0.00603611	-0.0117966	-0.0363879	0.0387256	0.321 (30)
point 32	-0.0141264	0.0294683	0.000214638	0.03268	0.274 (32)
point 33	0.0057496	-0.0105475	-0.00599527	0.0134258	0.368 (25)
point 34	0.00262267	-0.00916753	-0.0295321	0.0310333	0.293 (23)
point 36	-0.00587321	-0.0135966	0.0327071	0.0359043	0.198 (16)
point 37	0.0021875	-0.00548306	-0.00521472	0.0078767	0.312 (34)
point 42	-0.0136785	0.00553834	-0.0358661	0.0387834	0.305 (26)
point 43	0.00466703	-0.0089122	-0.0270446	0.0288551	0.256 (23)
point 46					0.310 (5)
point 48	-0.000665488	0.0140319	0.0296337	0.0327947	0.302 (23)
point 50	-0.0129144	0.0189177	0.040128	0.0462052	0.221 (25)
point 51	-0.0264076	-0.00824695	-0.00256628	0.0277841	0.217 (30)
point 55	0.0179549	-0.00126387	-0.0393812	0.0432996	0.195 (25)
point 57	0.0156937	-0.0408096	0.0198493	0.0480178	0.287 (34)
point 64	0.00736867	0.00405519	-0.0319126	0.0330024	0.266 (28)
point 67	0.00421548	0.0137656	-0.032598	0.0356356	0.359 (25)
point 68	-0.00353365	-0.0103954	-0.00126318	0.011052	0.231 (28)
point 70	-0.0122417	-0.00165513	-0.0399845	0.0418492	0.265 (29)
point 71	0.0103403	0.0202022	-0.0502432	0.0551311	0.216 (19)
point 72	-0.00189918	0.00949469	-0.0427167	0.0438004	0.254 (26)
point 75					0.101 (2)
point 76	0.00680472	0.0030508	0.0179478	0.0194354	0.362 (16)
point 77	-0.0106206	-0.00561043	-0.0292843	0.031652	0.256 (21)
point 78	-0.000523355	0.00161969	-0.000300541	0.00172847	0.327 (19)
point 79	-0.00801983	0.000899684	0.0448784	0.0455982	0.330 (16)

Label	X error (m)	Y error (m)	Z error (m)	Total (m)	Image (pix)
point 81	-0.000555807	-0.0202397	-0.0100421	0.0226009	0.406 (19)
point 82	-0.0003749	0.0113555	0.00761364	0.0136768	0.342 (21)
point 83	0.00841309	-0.00266547	0.000984666	0.00888	0.313 (15)
point 86	0.000783308	-0.00901283	0.00224658	0.00932158	0.319 (21)
point 88	0.00216935	-0.00868559	-0.0165895	0.0188509	0.221 (14)
point 89	-0.00324209	-0.0192993	-0.0327328	0.0381367	0.354 (20)
point 90	0.00816277	-0.0190909	-0.0345595	0.0403169	0.322 (19)
point 92	-0.00166648	-0.0151016	0.00759084	0.016984	0.207 (19)
point 93	-0.00963108	-0.00331462	0.0022703	0.0104355	0.369 (16)
point 96	0.00735266	0.0119666	-0.0145589	0.0202292	0.220 (24)
point 99	-0.0284524	0.00368808	-0.0357096	0.0458073	0.207 (21)
point 103	-0.00821928	0.00318183	-0.0223237	0.0240006	0.207 (15)
point 104	-0.00391083	0.00248208	-0.0286936	0.0290651	0.310 (17)
point 106	-0.00521396	0.00588026	-0.0336228	0.0345291	0.398 (33)
point 107	0.00126685	-0.00885005	0.026604	0.028066	0.271 (15)
point 108	-0.00244508	-0.00298407	-0.0279649	0.0282297	0.405 (22)
point 109	-0.00664244	-0.0219929	0.00623081	0.023804	0.288 (12)
point 111	0.00695195	-0.0362034	0.0246696	0.0443577	0.293 (16)
point 112	-0.00528029	-0.0301545	0.0223963	0.0379311	0.379 (10)
point 113	-0.00114039	-0.00420206	-0.00525145	0.0068217	0.340 (17)
point 114	-0.00129517	-0.00390582	0.0162182	0.0167321	0.439 (23)
point 118	0.0128615	0.00842141	0.016623	0.022642	0.296 (18)
point 120	0.0159563	-0.007385	0.0142334	0.0226215	0.194 (13)
point 121	0.00725722	-0.0116975	0.018396	0.0229763	0.416 (6)
point 126	0.0110119	0.00017289	0.0183605	0.0214102	0.228 (15)
point 131	0.00208999	-0.00540731	0.00957714	0.011195	0.215 (13)
point 132	0.00517093	-0.00198691	0.0139412	0.0150014	0.283 (18)
point 134	0.0165014	-0.00323381	-0.0308524	0.0351372	0.232 (21)
point 135	0.00116645	-0.00198731	0.00440296	0.00496951	0.335 (11)
point 137	0.0159274	0.00717956	-0.0204152	0.0268702	0.394 (14)
point 138	-0.0112273	0.0195258	-0.0579666	0.0621887	0.430 (21)
point 140	-0.010566	0.0117169	0.00240577	0.0159598	0.479 (19)

Label	X error (m)	Y error (m)	Z error (m)	Total (m)	Image (pix)
point 141	0.0107991	-0.00770949	-0.0323213	0.0349388	0.364 (15)
point 143	0.013205	-0.0115986	-0.018635	0.0256157	0.360 (20)
point 144	0.00796178	0.00438346	-0.0570042	0.0577242	0.304 (24)
point 148	0.00268287	0.00944559	-0.0380155	0.0392631	0.234 (21)
point 149	-0.0167077	0.00844002	-0.0282612	0.0338981	0.284 (18)
point 150	-0.00533569	0.0100811	-0.0044125	0.0122298	0.361 (20)
point 152	0.000406016	0.012624	0.00724399	0.0145604	0.400 (23)
point 153	0.00627311	0.0111786	-0.0234648	0.0267378	0.222 (16)
point 155	0.00720745	-0.00527242	-0.0256713	0.0271802	0.328 (18)
point 156	0.010326	0.00399772	-0.00644753	0.0128132	0.347 (7)
point 160	-0.0212388	-0.0161638	-0.0546757	0.0608423	0.293 (25)
point 161	0.00403267	0.0122008	-0.0355974	0.0378457	0.308 (20)
point 163	-0.0129135	-0.0131309	-0.042707	0.0465088	0.550 (20)
point 166	0.000744369	-0.0165384	0.00703069	0.0179862	0.403 (23)
point 171	-0.0024022	0.00645616	-0.0169692	0.0183141	0.314 (17)
point 172	-0.0197608	0.00918501	0.00154072	0.0218456	0.242 (16)
point 173	-0.00413324	-0.00129569	0.000767196	0.00439898	0.311 (16)
point 175	-0.00572773	0.0055568	-0.0271759	0.0283234	0.303 (17)
**Total**	**1.02182**	**0.425251**	**0.031451**	**1.10722**	**0.316**

Table 5. Check points.

X - Longitude, Y - Latitude, Z - Altitude.

Digital Elevation Model

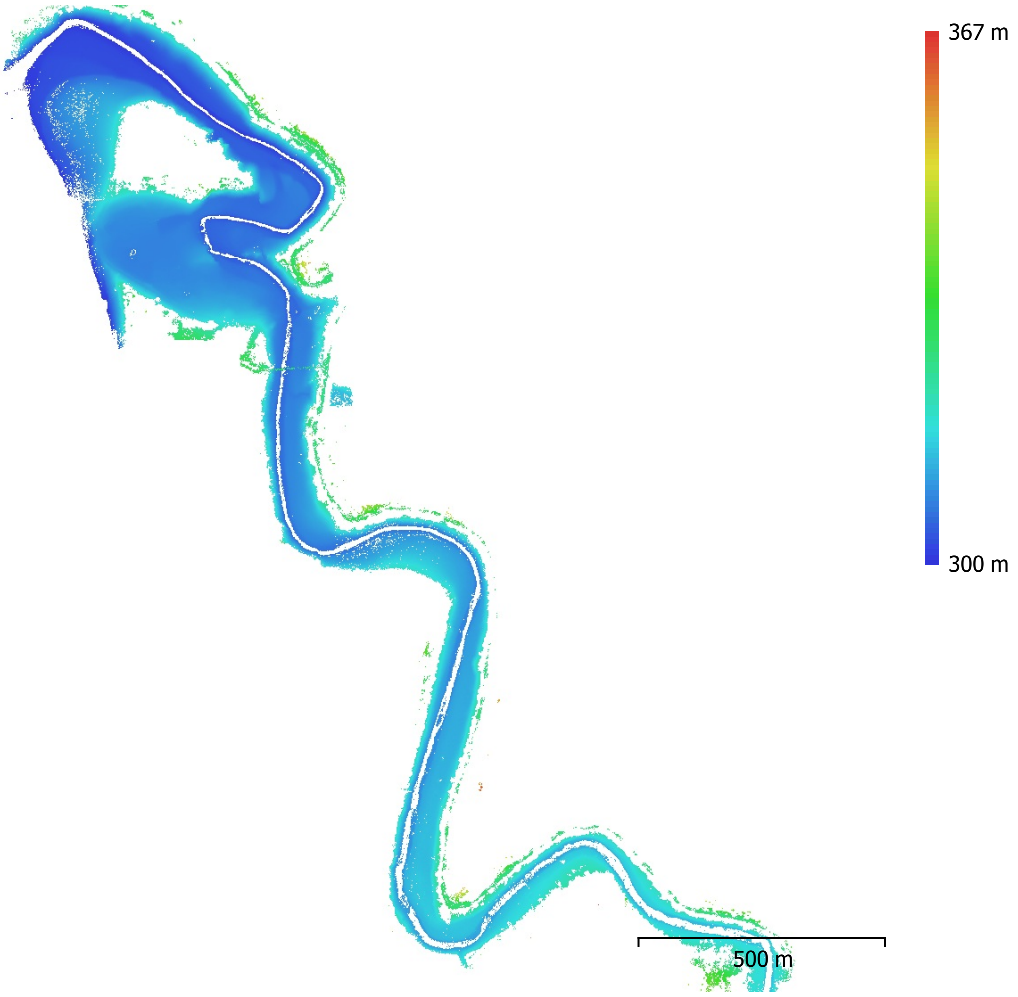

Fig. 4. Reconstructed digital elevation model.

Resolution:	unknown
Point density:	unknown

Processing Parameters

**General**	
Cameras	1527
Aligned cameras	1498
Markers	175
Coordinate system	WGS 84 (EPSG::4326)
Rotation angles	Yaw, Pitch, Roll
**Tie Points**	
Points	1,226,122 of 5,645,089
RMS reprojection error	0.131727 (0.279645 pix)
Max reprojection error	0.30369 (1.08802 pix)
Mean key point size	2.10773 pix
Point colors	3 bands, uint8
Key points	No
Average tie point multiplicity	2.99846
**Alignment parameters**	
Accuracy	High
Generic preselection	Yes
Reference preselection	Source
Key point limit	60,000
Key point limit per Mpx	1,000
Tie point limit	0
Exclude stationary tie points	Yes
Guided image matching	No
Adaptive camera model fitting	No
Matching time	53 minutes 32 seconds
Matching memory usage	1.52 GB
Alignment time	49 minutes 48 seconds
Alignment memory usage	1.61 GB
**Optimization parameters**	
Parameters	f, cx, cy, k1-k3, p1, p2
Adaptive camera model fitting	No
Optimization time	25 seconds
Date created	2023:10:20 15:19:02
Software version	2.0.0.15597
File size	302.69 MB
**System**	
Software name	Agisoft Metashape Professional
Software version	2.0.3 build 16960
OS	Windows 64 bit
RAM	63.90 GB
CPU	Intel(R) Core(TM) i7-7700 CPU @ 3.60GHz
GPU(s)	Quadro M4000
